# Enantioselective Synthesis in Continuous Flow: Polymer-Supported Isothiourea-Catalyzed Enantioselective Michael Addition–Cyclization with α-Azol-2-ylacetophenones

**DOI:** 10.1021/acs.oprd.4c00113

**Published:** 2024-05-02

**Authors:** Zhanyu Zhou, Kevin Kasten, Tengfei Kang, David B. Cordes, Andrew D. Smith

**Affiliations:** EaStCHEM, School of Chemistry, University of St. Andrews, North Haugh, St. Andrews KY16 9ST, U.K.

**Keywords:** packed bed reactor, supported isothiourea HyperBTM, continuous flow, α,β-unsaturated acyl-ammonium, enantioselective catalysis

## Abstract

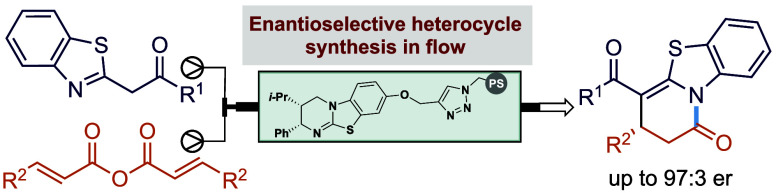

A packed reactor bed incorporating a polymer-supported isothiourea HyperBTM catalyst derivative has been used to promote the enantioselective synthesis of a range of heterocyclic products derived from α-azol-2-ylacetophenones and -acetamides combined with alkyl, aryl, and heterocyclic α,β-unsaturated homoanhydrides in continuous flow via an α,β-unsaturated acyl-ammonium intermediate. The products are generated in good to excellent yields and generally in excellent enantiopurity (up to 97:3 er). Scale-up is demonstrated on a 15 mmol scale, giving the heterocyclic product in 68% overall yield with 98:2 er after recrystallization.

## Introduction

**Scheme 1:**
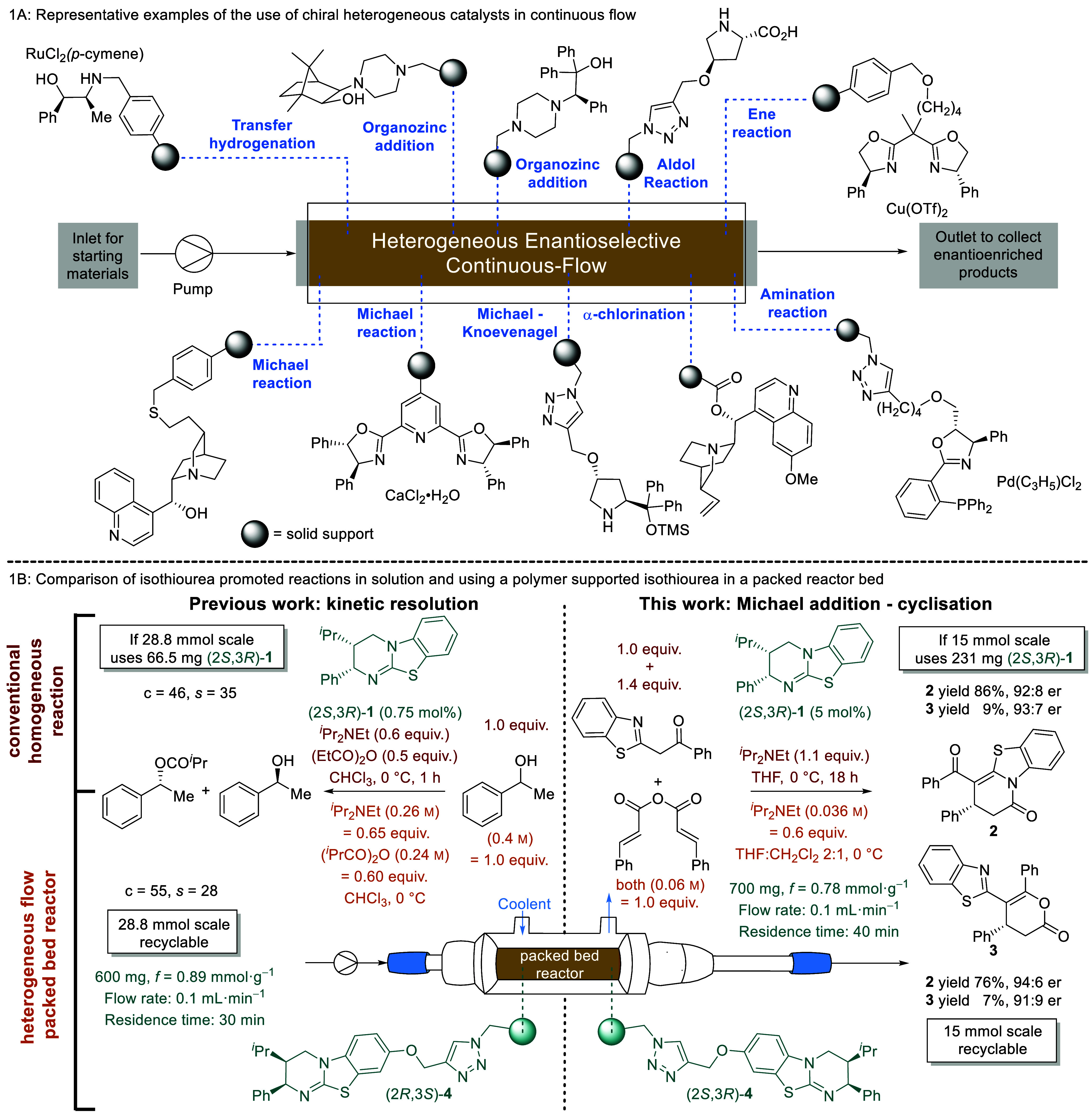
(A) The Application of Immobilised Asymmetric Organocatalysts in Flow and (B) Comparison of Isothiourea-Catalyzed Traditional Homogeneous Reactions and Comparison to Heterogeneous Packed Bed Reactor Processes in Flow

Enantioselective organocatalyzed reaction processes are now established as an effective alternative to metal and biocatalyzed transformations, allowing the formation of complex enantioenriched products from simple starting materials.^1^ Despite significant advances in this area, the most commonly recognized drawback to the use of organocatalysts is the relatively high catalyst loading (often 10–20 mol %) that is typically required for effective catalysis, combined with their generally poor recyclability. Consequently, the design and application of recyclable organocatalysts are of high interest, allowing a more sustainable and cost-effective approach. In this context, the heterogenization of homogeneous chiral catalysts is a promising approach that has been investigated through the attachment of chiral catalysts to organic polymers, dendrimers, membrane supports, or porous inorganic oxides.^2−8^ When coupled with advances in continuous-flow technology,^9−12^ a range of asymmetric reaction processes have been demonstrated, ranging from applications of transfer hydrogenation and organozinc addition to aldol and Michael addition reaction processes (Scheme 1A).^13−26^

As a representative example, in 2017, Pericàs and co-workers reported an asymmetric cycloaddition reaction promoted by an immobilized variant of the isothiourea catalyst benzotetramisole (BTM).^23^ The isothiourea catalyst was attached to a polymer to give a new class of immobilized Lewis base organocatalysts that afforded cycloaddition products with excellent yield and stereoselectivity. The immobilized catalysts could be recycled by filtration but showed mechanical degradation. However, incorporating this heterogeneous catalyst into a continuous-flow setup using a packed bed reactor allowed the enantioselective reaction to be performed and allowed separation of the supported catalyst simultaneously. Such packed bed reactors allow the reaction solution to pass through a polymer-supported catalyst embedded between two filters to achieve continuous product formation.^12^ Compared to conventional batch reactors, such a strategy has several advantages such as (i) continuous flow can avoid hot spots effectively, (ii) higher effective equivalents of the catalyst/reagent loading compared to substrate are offered, leading to improved efficiency, and (iii) no additional separation process is required to recycle the immobilized catalyst.^10^ We applied these principles to previous work on the acylative kinetic resolution of secondary and tertiary alcohols employing the isothiourea HyperBTM (2*S*,3*R*)-**1** as an organocatalyst.^27−31^ Consequently, HyperBTM was immobilized onto a Merrifield resin ((2*S*,3*R*)-**4**) and applied to the KR of a range of both secondary and tertiary alcohols in continuous flow, allowing an effective KR on a 28.8 mmol scale to be carried out with yields and selectivities comparable to those obtained from the batch process via an acyl-ammonium intermediate (Scheme 1B).^32^ Notably, all of these kinetic resolution processes were applied with the same packed bed of (2*R*,3*S*)-**4**, resulting in a total operation time in excess of 100 h in flow without significant degradation.

Nitrogen-containing heterocycles are privileged structural motifs commonly found in bioactive natural products, pharmaceuticals, and agrochemicals.^33^ For example, pyrazolone and thiazolone scaffolds have found broad applications as bioactive compounds in medicinal chemistry, including antiplatelet,^34^ anti-inflammatory,^35^ and anticancer^36^ activity. They are also utilized as ligands,^37^ organic semiconductors, and dyes.^38^ As a consequence, the development of effective methods to allow the preparation of nitrogen-containing heterocycles is a recognized challenge in the synthetic community. In previous work, we reported the isothiourea HyperBTM (2*S*,3*R*)-**1** catalyzed enantioselective formal cycloaddition reaction between 2-phenacylbenzothiazole and cinnamic anhydride (Scheme 1B), giving access to **2** and **3** in good yield and enantioselectivity.^39^ In this manuscript we demonstrate the efficiency of this protocol in continuous flow, allowing the first use of an immobilized isothiourea catalyst to exploit the formation of an α,β-unsaturated acyl-ammonium intermediate.^40,41^ The immobilized catalyst (2*S*,3*R*)-**4** is loaded in a jacketed Omnifit column and connected to a single-piston pump that is used to generate the reaction flow and form the product continuously. To showcase the potential industrial applicability of this process, a 15 mmol scale-up was performed to demonstrate the durability and recyclability of the immobilized HyperBTM catalyst (2*S*,3*R*)-**4**.

## Results and Discussion

Polymer-supported catalyst (2*S*,3*R*)-**4** was prepared by demethylation of (2*S*,3*R*)-8-methoxyHyperBTM and subsequent immoblization onto Merrifield resin via previously established methodology.^24,32^ Initial proof-of-concept and subsequent optimization were used, employing a model system consisting of the addition of 2-phenacylbenzothiazole to cinnamic anhydride. Polystyrene-supported catalyst (2*S*,3*R*)-**4** (700 mg, 0.55 mmol) was loaded in a size-adjustable, medium-pressure borosilicate glass column to create a vertical packed bed reactor (flow from bottom to top) fitted with a cooling jacket to control the reaction temperature using a recirculating chiller. Based upon our previous demonstration of kinetic resolutions in flow,^32^ solutions of 2-phenacylbenzothiazole (0.060 m = 1.0 equiv.) and base (0.066 m = 1.1 equiv.) in one syringe and cinnamic anhydride (0.084 m = 1.4 equiv.) in another syringe were delivered to the reactor bed via a mixing T-piece using a syringe pump with a flow rate of 0.1 mL·min^–1^ equating to a residence time of 40 min using PTFE tubing with a 1/32″ inner diameter. Optimization studies aimed to maximize product enantioselectivity and yield and began with the screening of reaction solvents (Table 1). The use of CHCl_3_ gave **2** in a good 89% yield but moderate 72:28 er with good regiocontrol (93:7 ratio of lactam **2**:lactone **3** arising from either *N*- or *O*-cyclization, respectively, Table 1, entry 1). Toluene or CH_2_Cl_2_ gave only poor conversion, but CH_2_Cl_2_ gave product **2** in high enantioselectivity (95:5 er, Table 1, entry 4). Performing the reactions in THF, in industrially preferred EtOAc, or CPME provided **2** in good yield (72–91%) but slightly reduced enantioselectivity (91:9 er), with THF giving the highest product yield (Table 1, entries 3, 5, and 6). Based on these findings, the use of a mixed solvent system consisting of CH_2_Cl_2_ (best enantioselectivity) and THF (highest yield) was trialled (Table 1, entries 3 and 7–10). Using different proportions of CH_2_Cl_2_ and THF, trends in reactivity indicated increased enantioselectivity but a decreasing yield with higher proportions of CH_2_Cl_2_. A 2:1 mixture of THF:CH_2_Cl_2_ (entry 8) was identified as optimal, leading to the best compromise between product yield and enantioselectivity. Another mixed solvent system involving ethyl acetate was also tested, resulting in a slightly decreased yield but otherwise unchanged er (Table 1, entry 11). The absolute configuration of the products was identified by comparison to that within the literature, consistent with the generally accepted stereochemical model for these types of processes.^28,39,42−60^

**Table 1:**
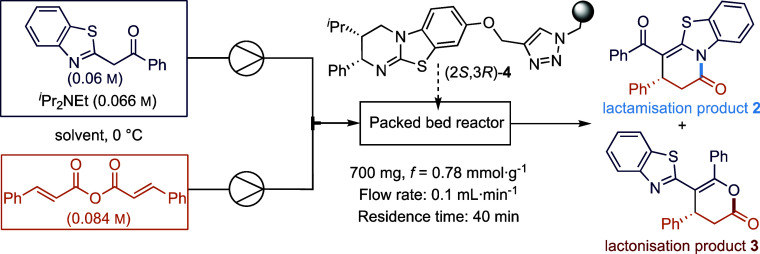
Screening of Reaction Solvents^a^

entry	solvent	**2** er^b^	**3** er^b^	**2** yield (%)^c^	**2**:**3**^d^
1	CHCl_3_	72:28	84:16	89	93:7
2	toluene	92:8	96:4	50	85:15
3	THF	90:10	88:12	91	92:8
4	CH_2_Cl_2_	95:5	94:6	59	92:8
5	EtOAc	91:9	92:8	84	89:11
6	CPME	91:9	92:8	72	90:10
7	THF:CH_2_Cl_2_ (1:1)	94:6	91:9	75	87:13
8	THF:CH_2_Cl_2_ (2:1)	93:7	92:8	86	86:14
9	THF:CH_2_Cl_2_ (4:1)	93:7	92:8	86	84:16
10	THF:CH_2_Cl_2_ (8:1)	92:7	91:9	91	84:16
11	EtOAc:CH_2_Cl_2_ (1:1)	93:7	91:9	71	85:15

a Reactions were carried out using the same catalyst bed of 700 mg polymer-supported (2*S*,3*R*)-**4** with 0.1 mL·min^–1^ flow rate, and the catalyst is regenerated using MeOH/chloroform (1:9) after each reaction.

b The er was determined by HPLC analysis on a chiral stationary phase.

c Isolated yield.

d The ratio of products was determined by ^1^H NMR of the crude reaction mixture.

To further increase product enantioselectivity, the effect of base and anhydride equivalents was investigated. A series of reactions using varying equivalents of ^*i*^Pr_2_NEt (from 1.1 to 0.0 equiv.) was performed, with relatively little variation in selectivity and product yield (Table 2, entries 1–6). Optimal product enantioselectivity was obtained using 0.6 equiv. of ^*i*^Pr_2_NEt, which also resulted in a good yield and enantioselectivity (86%, 95:5 er) (Table 2, entry 3). The effect of varying the equivalents of the α,β-unsaturated anhydride (from 1.4 to 1.0 equiv.) was investigated (entries 7–11). Although little variation in selectivity was observed overall, a general trend of increased formation of **2** over **3**, combined with decreasing isolated yield but increased enantioselectivity of major product **2** was observed with decreasing equivalents of the cinnamic anhydride. Choosing to maximize product er was prioritized, with 1.0 equiv. of cinnamic anhydride selected as the best conditions for this process as it minimized reagent excess while maintaining high yield (entry 10 prioritized over entry 3). Interestingly, performing the reactions without base and reduced anhydride concentrations resulted in a significantly decreased yield of **2** (entry 11). In an attempt to further increase the product yield, the residence time was doubled by decreasing the flow rate from 0.1 to 0.05 mL·min^–1^ (entry 12), resulting in similar product yields and enantioselectivities. Under conditions comparable to those for entry 10, use of the immobilized catalyst (2*S*,3*R*)-**4** in batch gave an 86:14 mixture of **2**:**3**, with product **2** isolated in 68% yield (91:9 er) and product **3** in 11% yield (92:8 er; see the SI for full details).

**Table 2:**
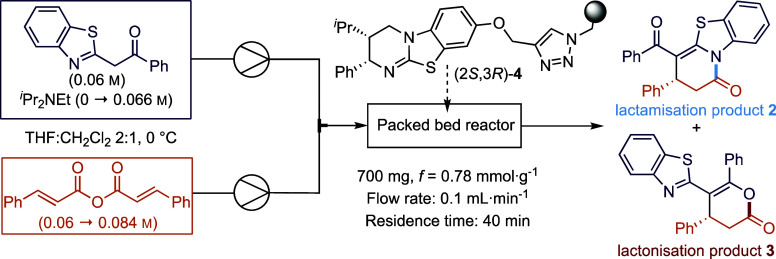
Screening of Base and Anhydride Equivalents^a^

entry	^*i*^Pr_2_Net (m)	anhydride (m)	**2** er^b^	**3** er^b^	**2** yield (%)^c^	**2**:**3**^d^
1	0.066	0.084	93:7	92:8	86	86:14
2	0.048	0.084	94:6	92:8	84	85:15
3	0.036	0.084	95:5	92:8	86	85:15
4	0.024	0.084	95:5	91:9	84	83:17
5	0.009	0.084	93:7	91:9	82	83:17
6	0.000	0.084	94:6	92:8	78	80:20
7	0.036	0.078	94:6	92:8	84	86:14
8	0.036	0.072	93:7	91:9	83	88:12
9	0.036	0.066	94:6	91:9	81	89:11
10	0.036	0.060	94:6	91:9	76	92:8
11	0.000	0.060	94:6	93:7	59	81:19
12^e^	0.036	0.060	93:7	92:8	78	93:7

a Reactions were carried out using the same catalyst bed of 700 mg polymer-supported (2*S*,3*R*)-**4** with 0.1 mL·min^–1^ flow rate in THF:CH_2_Cl_2_ = 2:1, and the catalyst is regenerated using MeOH/chloroform (1:9) after each reaction.

b The er was determined by HPLC analysis on a chiral stationary phase.

c Isolated yield.

d The ratio of products was determined by ^1^H NMR of the crude reaction mixture.

e Reaction was carried out with a flow rate of 0.05 mL·min^–1^.

### Scope and Limitations of the Enantioselective Annulation Process in Flow

**Scheme 2:**
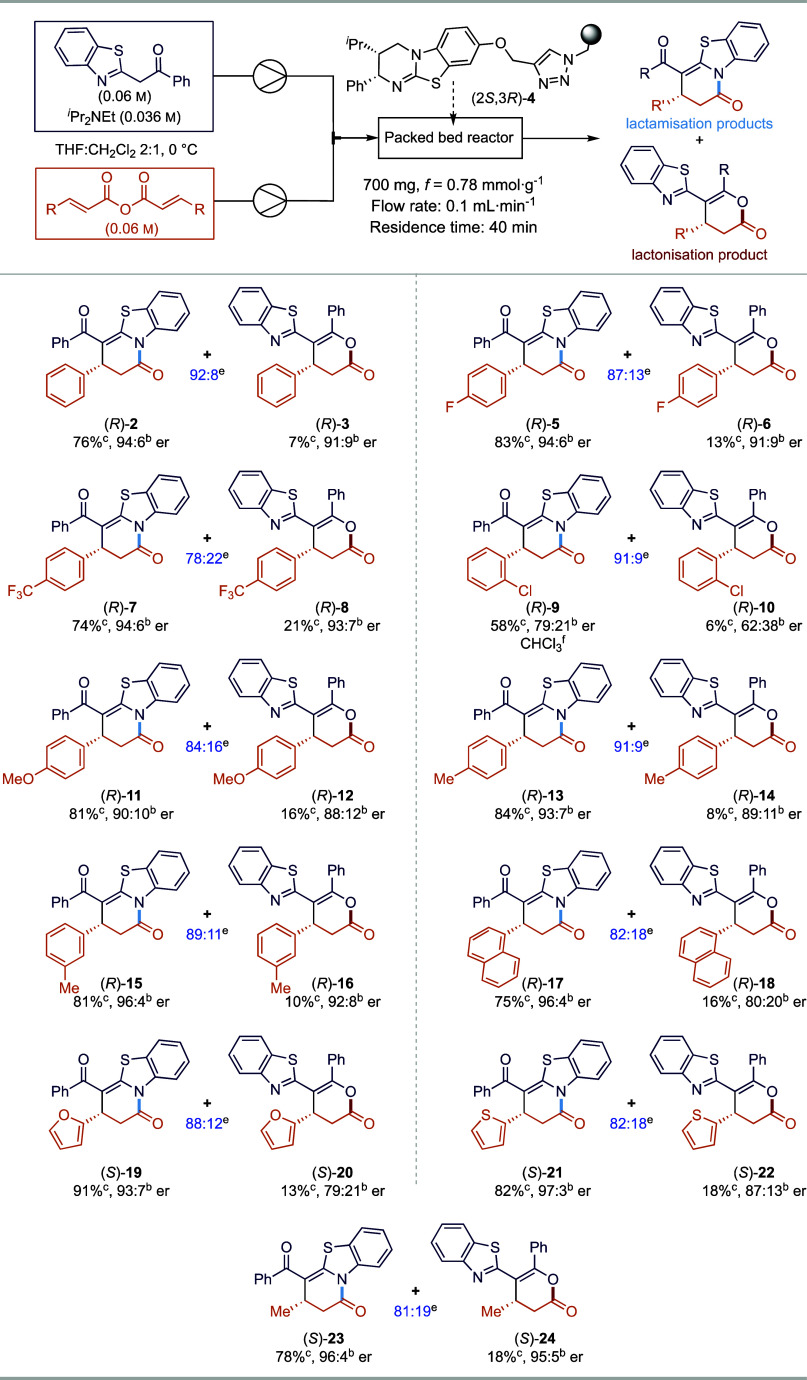
Scope of the Annulation Reaction Reactions were carried out using the same catalyst bed of 700 mg polymer-supported (2*S*,3*R*)-**4** with 0.1 mL·min^–1^ flow rate in THF:CH_2_Cl_2_ = 2:1, and the catalyst is regenerated using MeOH/chloroform (1:9) after each reaction. The er was determined by HPLC analysis on a chiral stationary phase. Isolated yield. ^1^H NMR yield. Ratio of regioselectivity by ^1^H NMR of the crude reaction mixture. Reactions were carried out in CHCl_3_ due to problems with solubility of the starting materials.

Having identified the optimal conditions for the model reaction process, the scope and limitations of this annulation were investigated. Initially, variation within a range of α,β-unsaturated anhydrides was probed (Scheme 2). In each case the constitutional isomeric products were separable, with the lactam product arising from *N*-cyclization being dominant. Incorporation of an electron-withdrawing 4-F_3_CC_6_H_4_ substituent and a halogenated 4-FC_6_H_4_ substituent led to a slight reduction in regioselectivity of the reaction, giving constitutional isomers **5**/**6** and **7**/**8** in good yield (83%/13%, 74%/21%, respectively) and excellent enantioselectivity (94:6/91:9, 94:6/93:7 er). 2-Chlorocinnamic anhydride led to products **9/10** in only moderate yield (58%/6% yield) and poor enantioselectivity (79:21/62:38 er) using CHCl_3_ as the solvent due to the poor solubility of 2-chlorocinnamic anhydride in THF:CH_2_Cl_2_ (2:1). 4-MeOC_6_H_4_ substitution was tolerated and gave **11**/**12** in good yield (81%/16% yield) but slightly decreased regioselectivity (90:10/88:12). 4-MeC_6_H_4_**13**/**14** and 3-MeC_6_H_4_ substitution **15/16** led to a slight improvement in the yield (84%/8% yield, 81%/10% yield, respectively) with excellent enantioselectivity in either case (93:7/89:11 er, 96:4/92:8 er, respectively). In addition, the reaction scope was extended to incorporate 1-naphthyl substitution, as well as heterocyclic 2-furyl and 2-thienyl substituents, which gave good yields of **17**/**18**, **19**/**20**, and **21**/**22**, respectively, with excellent enantioselectivity (96:4/80:20, 93:7/79:21, 97:3/87:13 er, respectively). Alkyl substitution within the anhydride was also tolerated, giving **23**/**24** in a good yield with high enantioselectivity (96:4/95:5 er).

**Scheme 3:**
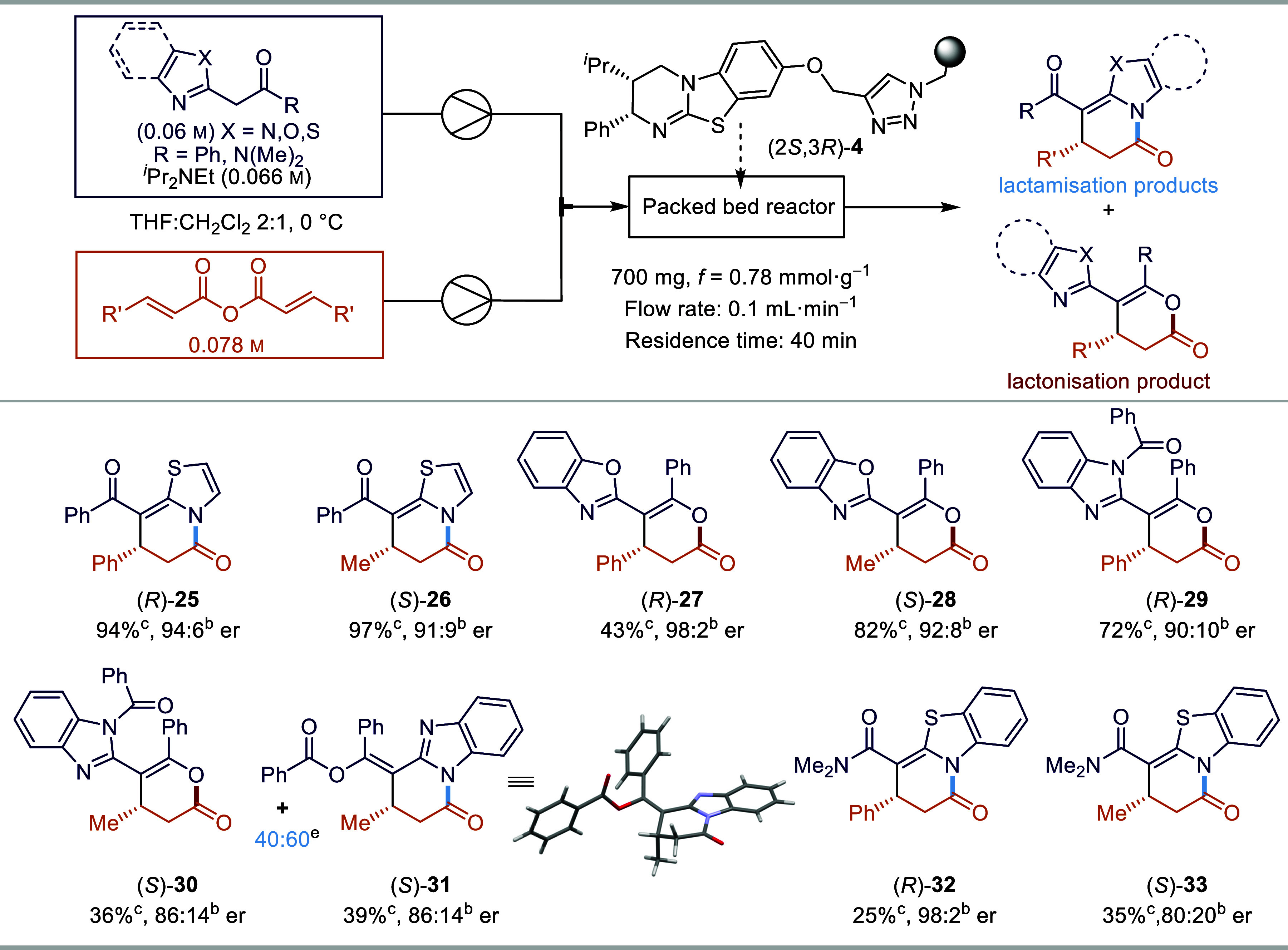
Scope of the Annulation Reaction with Variation of Heteroaromatic Enol Nucleophiles Reactions were carried out using the same catalyst bed of 700 mg polymer-supported (2*S*,3*R*)-**4** with 0.1 mL·min^–1^ flow rate in THF, and the catalyst is regenerated using MeOH/chloroform (1:9) after each reaction. The er was determined by HPLC analysis on a chiral stationary phase. Isolated yield.

To further probe reactivity and regioselectivity in these annulation processes, a range of α-azol-2-ylacetophenones was synthesized and used with both cinnamic anhydride and crotonic anhydride with slightly altered conditions to ensure optimal yields (THF was used as a single solvent, Scheme 3). The reaction with 2-phenacylthiazole gave exclusively the lactamization products for both cinnamic anhydride and crotonic anhydride, giving **25** and **26**, respectively, in excellent yield (94%, 97% yield) and with good enantioselectivity (94:6, 91:9 er). For the reaction of 2-phenacylbenzoxazole with cinnamic anhydride, a complete switch in regioselectivity compared with 2-phenacylbenzothiazole was observed, giving almost exclusively the lactonization product **27** in good enantioselectivity (98:2 er) albeit in moderate yield (43% yield). Changing to crotonic anhydride, **28** was obtained in an increased 82% yield but at the cost of decreased enantioselectivity (92:8 er). The use of 2-phenacylbenzimidazole with cinnamic anhydride showed good activity and regioselectivity but gave a 50:50 mixture of tautomeric products (combined yield of 92%) that could not be separated by HPLC analysis (see the SI for further information). To simplify the product mixture, acylation using benzoyl chloride was successfully attempted, giving **29** as the sole product in good yield (76%) and enantioselectivity (90:10 er). The use of crotonic anhydride in a similar procedure gave separable products *N*-acyl **30** and *O*-acyl **31** in a good overall combined yield (75%) with moderate enantioselectivity (86:14 er). Unambiguous confirmation of the constitution of *O*-acyl derivative **31** was achieved by single crystal X-ray analysis.^61^ Unfortunately, the use of 2-benzothiazol-2-yldimethylacetamide led to reduced reactivity with both cinnamic anhydride (giving **32**, 25% yield, 98:2 er) and crotonic anhydride (giving **33**, 35% yield, 80:20 er).

**Scheme 4:**
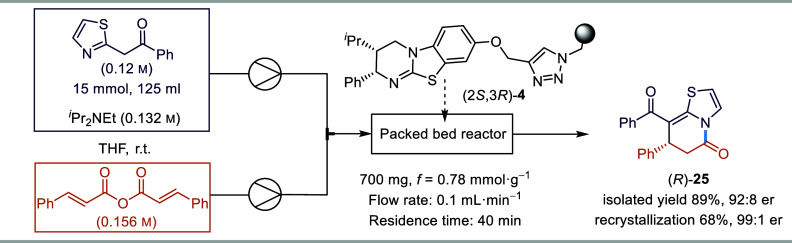
15 mmol Scale-Up under Optimized Reaction Conditions

Finally, the robustness of the same packed bed reactor was further probed by performing the annulation of 2-phenacylthiazole with cinnamic anhydride on a 15 mmol scale over a 42 h period (Scheme 4). An isolated yield for **25** of 89% (4.45 g) with a 92:8 er was obtained for the 15 mmol scale reaction, similar to the results observed on a 0.3 mmol scale consistent with no significant catalyst degradation or inactivation. Recrystallization of product (*R*)-**25** led to improved enantiopurity (99:1 er), resulting in an overall yield of 68% (3.4 g). As a control to monitor potential catalyst decomposition over this extended run, the reaction on a 0.3 mmol reaction scale was carried out directly before and after the scaled-up reaction for comparison, giving comparable results (see the SI for full details). Furthermore, the conversion to product was monitored at various time-points throughout the 42 h run, again indicating no significant deterioration of catalyst performance.

In conclusion, we have demonstrated the first use of a polymer-supported isothiourea in a packed bed reactor for enantioselective annulation reactions of α-azol-2-ylacetophenones and acetamides with α,β-unsaturated anhydrides via an α,β-unsaturated acyl-ammonium intermediate in continuous flow. In this protocol, the use of 2-phenacylbenzothiazole as a pronucleophile has been applied to generate a range of heterocyclic products, with good reactivity observed with alkyl, aryl, and heterocyclic β-substituted α,β-unsaturated homoanhydrides. Additionally, three alternative α-azol-2-ylacetophenones and one α-azol-2-ylacetamide were investigated, with their reactivity and regioselectivity in this annulation process explored, giving products in high yield (up to 96%) and excellent enantioselectivity (up to 99:1 er after recrystallization). All optimization, demonstration of the scope and limitations, and scale-up in this report used the same 700 mg batch of polymer-supported catalyst to generate the fixed reactor bed. This indicates that the polymer-supported isothiourea HyperBTM (2*S*,3*R*)-**4** exhibits a consistent and stable catalytic performance to promote the asymmetric annulation of α,β-unsaturated anhydrides with α-azol-2-ylacetophenones and -acetamides.

## Data Availability

All data (experimental procedures, characterization data including spectra) that support the findings of this study are available within the article and its Supporting Information. Crystallographic data for compound (*S*)-**31** have been deposited with the Cambridge Crystallographic Data Centre under deposition number 2339361. The research data supporting this publication can be accessed from the University of St. Andrews Research Portal Pure ID: 300191231 (“Enantioselective Synthesis in Continuous Flow: Polymer Supported Isothiourea Catalyzed Enantioselective Michael Addition-Lactamisation with Azaaryl Ketones”, 10.17630/9eec7d03-0634-4239-ab47-bdfe105813ec).

## Supporting Information Available

Experimental procedures, characterization data, NMR spectra, HPLC traces, as well as X-ray crystallographic data for **31** (PDF)
