# Supplementary material for: Enantioselective Synthesis in Continuous Flow: Polymer-Supported Isothiourea-Catalyzed Enantioselective Michael Addition–Cyclization with α-Azol-2-ylacetophenones
Source: Org Process Res Dev. 2024 May 2;28(5):2041–9. doi: 10.1021/acs.oprd.4c00113 (PMC11110067; doi:10.1021/acs.oprd.4c00113)
Supplement: Supplementary file 1 — op4c00113_si_001.pdf [file op4c00113_si_001.pdf]

Enantioselective Synthesis in Continuous Flow: Polymer Supported Isothiourea Catalyzed Enantioselective Michael Addition-Cyclisation with α-Azol-2-ylacetophenones

Zhanyu Zhou, Kevin Kasten, Tengfei Kang, David. B. Cordes, Andrew D. Smith*

EaStCHEM, School of Chemistry, University of St Andrews, North Haugh, St Andrews, KY16 9ST, UK.

e-mail: ads10@st-andrews.ac.uk

Contents

1 General Experimental 3

2 Syntheses 6

2.1 General Procedure A: Synthesis of Polymer Supported HyperBTM 6

Synthesis of *tert*-butyl (phenyl(phenylsulfonyl)methyl)carbamate S1 6

Synthesis of *tert*-butyl benzylidenecarbamate S2 6

Synthesis of *tert*-butyl ((1*S*,2*S*)-2-formyl-3-methyl-1-phenylbutyl)carbamate S3 7

Synthesis of *tert*-butyl ((1*S*,2*S*)-2-(hydroxymethyl)-3-methyl-1-phenylbutyl)carbamate S4 7

Synthesis of (S)-2-((S)-amino(phenyl)methyl)-3-methylbutan-1-ol hydrochloride S5 8

Synthesis of 2-chloro-6-methoxybenzo[*d*]thiazole S6 8

Synthesis of (2*S*,3*R*)-3-isopropyl-8-methoxy-2-phenyl-3,4-dihydro-2*H*-benzo[4,5]thiazolo [3,2-*a*]pyrimidine S8 9

Synthesis of (2*S*,3*R*)-8-hydroxy-3-isopropyl-2-phenyl-3,4-dihydro-2H-benzo[4,5] thiazolo[3,2-a]pyrimidin-1-ium bromide S9 10

Synthesis of (2*S*,3*R*)-3-isopropyl-2-phenyl-8-(prop-2-yn-1-yloxy)-3,4-dihydro-2*H* benzo[4,5]thiazolo[3,2-*a*]pyrimidine S10 10

Synthesis of azide-functionalised Merrifield resin S11 11

Synthesis of Merrifield resin-supported (2S,3R)-HyperBTM derivative Cat (2*S*,3*R*)-4 11

2.2 General Procedure B: Synthesis of azaaryl ketone 12

Synthesis of 2-phenacylbenzothiazole S12 12

Synthesis of 2-phenacylbenzoxazole S13 12

Synthesis of 2-phenacylthiazole S14 13

Synthesis of 2-phenacylbenzimidazole S15 13

Synthesis of 2- dimethylformylbenzothiazole S16 14

2.3 General Procedure C: Synthesis of α,β-unsaturated homoanhydrides 14

(*E*)-Cinnamic anhydride S17 15

(*E*)-3-(4-Fluorophenyl)acrylic anhydride S18 15

(*E*)-3-(4-(Trifluoromethyl)phenyl)acrylic anhydride S19 15

(*E*)-3-(4-Methoxyphenyl)acrylic 3-(4-methoxyphenyl)propanoic anhydride S20 15

(*E*)-3-(Furan-2-yl)acrylic anhydride S21 16

(*E*)-3-(Thien-2-yl)acrylic anhydride S22 16

(*E*)-3-(3-Methylphenyl)acrylic anhydride S23 16

(*E*)-3-(2-Chlorophenyl)acrylic anhydride S24 17

(*E*)-But-2-enoic anhydride S25 17

(*E*)-3-(4-Methylphenyl)acrylic anhydride S26 17

(*E*)-3-(naphthalen-1-yl)acrylic anhydride S27 18

2.4 General Procedure D, Batch Procedure E and Scale-up Procedure F: Asymmetric annulations of α,β-unsaturated homo-anhydrides and aza-aryls 18

Table S1. Conversion of product changed during scale-up process 19

2.4.1 Lactams and lactones products 19

3 Single crystal X-ray diffraction data 37

4 References 39

Appendix I: ^{1}H, ^{13}C{^{1}H}, 2D ^{1}H COSY, 2D ^{1}H–^{13}C HSQC and 2D ^{1}H–^{13}C HMBC NMR Spectra 40

Appendix II: HPLC traces of compounds 80

1 General Experimental

Reactions involving moisture sensitive reagents were carried out in flame-dried glassware under an inert atmosphere (Ar or N_2) using standard vacuum line techniques. Anhydrous solvents (Et_2O, CH_2Cl_2, THF and PhMe) were obtained after passing through an alumina column (Mbraun SPS-800) or purchased in a sealed bottled under inert atmosphere. Organometallic reagents were titrated before use according to literature procedures.[1] Room temperature (r.t.) refers to 18 ± 3 °C, Petrol refers to petroleum ether with the boiling range of 40 – 60 °C, brine refers to saturated aqueous sodium chloride solution, ether refers to diethylether (Et_2O). All chemicals and solvents used were purchased by pertinent brands (Sigma Aldrich, Alfa Aesar, Acros, Apollo Scientific, TCI, STREM) and used without further purification unless stated. For reactions conducted during the day following cooling baths were applied: 0 °C (ice/water), −10 °C (ice/acetone), −20 °C (ice/NaCl), −45 °C (CO_2(s) or N_2(l)/MeCN), −60 °C (CO_2(s) or N_2(l)/$CHCl_3$) and −78 °C (CO_2(s)/acetone). Temperatures of 0 °C to −78 °C for overnight reactions were obtained using an immersion cooler (HAAKE EK 90). Reactions involving heating were performed using DrySyn blocks or oil baths and a contact thermocouple. Under reduced pressure refers to the use of either a Büchi Rotavapor R-200 with a Büchi V491 heating Bath and Büchi V-800 vacuum controller, a Büchi Rotavapor R-210 with a Büchi V-491 heating bath and Büchi V-850 vacuum controller, a Heidolph Laborota 4001 with vacuum controller, an IKA RV10 rotary evaporator with an IKA HB10 heating bath and ILMVAC vacuum controller, or an IKA RV10 rotary evaporator with an IKA HB10 heating bath and Vacuubrand CVC3000 vacuum controller. Rotary evaporator condensers are fitted to Julabo FL601 Recirculating Coolers filled with ethylene glycol and set to −6 °C.

Analytical thin layer chromatography (TLC)[2] was performed on pre-coated aluminium plates (Kieselgel 60 F_{254} silica) plates purchased from Merck. Visualisation was achieved using ultraviolet light (254 nm) and staining with aqueous $KMnO_4$ or ethanolic vanillin solution followed by heating. Flash column chromatography was performed in glass columns fitted with porosity 3 sintered discs over Silica gel 60 (0.043 – 0.060 mm) using standard techniques as reported in literature with the solvent system stated.[3] Automated chromatography was performed on a Biotage® Selekt™ SEL-2SV with a 200 – 400 nm UV-detector using the method stated and Biotage® Sfär™ Silica HC D or Biotage® Sfär™ Silica D columns.

HPLC analyses were obtained on either a Shimadzu HPLC consisting of a DGU-20A5 degassing unit, LC-20AT liquid chromatography pump, SIL-20AHT autosampler, CMB-20A communications bus module, SPD-M20A diode array detector and a CTO-20A column oven or a Shimadzu HPLC consisting of a DGU-20A5R degassing unit, LC-20AD liquid chromatography pump, SIL-20AHT autosampler, SPD-20A UV/Vis detector and a CTO-20A column oven. Separation was achieved using either DAICEL CHIRALCEL OD-H and OJ-H

columns or DAICEL CHIRALPAK AD-H, AS-H, IA, IB, IC and ID columns using the method stated. HPLC traces of enantiomerically enriched compounds were compared with authentic racemic spectra. Racemic compounds were synthesised under analogous reaction conditions using achiral or racemic catalysts where necessary.

Optical rotations were determined using a Perkin Elmer Precisely/Model-341 Polarimeter with a Na/Hal lamp (Na D line, 589 nm) at 20 °C.

Infrared spectra were recorded on a Shimadzu IRAffinity-1 Fourier transform IR spectrophotometer fitted with a Specac Quest ATR accessory (diamond puck). Spectra were recorded of either thin films or solids, with characteristic absorption wavenumbers (ν_{max}) reported in cm^{-1}.

^{1}H, ^{13}C, ^{19}F and ^{32}P nuclear magnetic resonance (NMR) spectra were recorded with Bruker Avance™ 300 Cryomagnet with a BBFO probe, Bruker Avance II™ 400 Ultrashield with a BBFO probe, Bruker Avance™ 500 Ultrashield with a SmartProbe BBFO+ probe or Bruker Avance III™ 500 Ascend™ with a CryoProbe Prodigy BBO probe using deuterated solvents ($CDCl_3$, CD_2Cl_2, D_2O, CD_3OD, CD_3CN, $(CD_3)_2SO$, $(CD_3)_2CO$, $C_6D_5CD_3$) purchased from Sigma-Aldrich. Chemical shifts (δ) are quoted in ppm and referenced to residual solvent signals reported in literature.[4] $^{13}C\{^{1}H\}$ and $^{19}F\{^{1}H\}$ spectra were acquired using a proton broadband decoupling sequence. ^{13}C were recorded with DEPTQ or UDEFT sequences. Couplings were indicated by the use of conventional agreed abbreviations: s (singlet), d (doublet), t (triplet), q (quartet), m (multiplet), dd (doublet of doublets), td (triplet of doublets), etc. Coupling constants (*J*) are denoted with the number of bonds involved in the upper left and with the atoms coupling in the bottom right edge of the symbol, e.g. $^{3}J_{HH}$. The abbreviation *Ar* denotes aromatic and app denotes apparent.[5] NMR peak assignments were confirmed using 2D ^{1}H correlated spectroscopy (COSY), ^{1}D selective ^{1}H nuclear Overhauser effect spectroscopy (NOESY), 2D ^{1}H–^{13}C heteronuclear multiple-bond correlation spectroscopy (HMBC), and 2D ^{1}H–^{13}C heteronuclear single quantum coherence (HSQC) where necessary. For analysis of NMR-spectra MestReNova and tools therein were used.[6] For Karplus analysis transformed equation 2 was used.

Melting points were recorded on an Electrothermal 9100 melting point apparatus and are not corrected; (dec) refers to decomposition.

Mass spectrometry (*m*/*z*) data were acquired using ThermoFisher Exactive Orbitrap mass spectrometer or Micromass GCT (TOF) mass spectrometer with solids probe. Ionisation techniques used are indicated for each compound. Values are quoted as a ratio of mass to charge (*m*/*z*) in Daltons [Da].[7]

Common chemical abbreviations were used to indicate chemical groups or environments such as Ph (phenyl), *Ar* (aromatic, not confuse with Argon), Bn (benzyl), Et (ethyl), Me (methyl). To indicate atoms numbering

schemes are displayed with the spectrum and deviate from IUPAC numbering for clarity. For names and numbering concerning stereodiscriptors IUPAC nomenclature was applied.[8]

Authentic racemic samples were prepared in an analogous fashion using racemic HyperBTM.

2 Syntheses

2.1 General Procedure A: Synthesis of Polymer Supported HyperBTM

Synthesis of *tert*-butyl (phenyl(phenylsulfonyl)methyl)carbamate S1

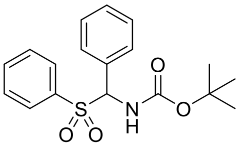

S1

Following the method described by Smith *et al.*,[1] to a 1 l round bottomed flask charged with THF (140 ml, 2.7 M) was added *tert*-butyl carbamate (38.00 g, 324.40 mmol), water (240 ml, 1.4 M), sodium benzenesulfinate (54.00 g, 328.89 mmol), benzaldehyde (35 ml, 344.30 mmol), and formic acid (70 ml, 1.90 mol) at room temperature. The mixture was stirred at this temperature overnight (15 h). The resulting precipitate was filtered and washed with water (50 ml) followed by hexane/CH_2Cl_2 (2 × 100 ml). The combined liquors were stirred for another 18 h. The resulting precipitated was filtered and washed (10 ml water, 9:1 Hexane:CH_2Cl_2, 10 ml). The combined solids were dried in an oven at 90 °C for 7 h to give the title compound **S1** as white solid (85.68 g, 246.60 mmol, 76%); mp 138 °C (hexane/CH_2Cl_2); ν_{max} (film)/cm^{-1} 2907 (C–H), 1718 (C=O), 1583 (C=C), 1468 (C–N); **^{1}H NMR** (400 MHz, $CDCl_3$) δ_H: 1.28 (9H, s, C(C*H*$_3$)$_3$), 5.78 (1H, d, *J* 11.0, N*H*), 5.95 (1H, d, *J* 10.8, C*H*), 7.39–7.51 (5H, m, ArC*H*), 7.51–7.71 (3H, m ArC*H*), 7.78–7.96 (2H, m, ArC*H*). The data is in agreement with the literature.[1]

Synthesis of *tert*-butyl benzylidenecarbamate S2

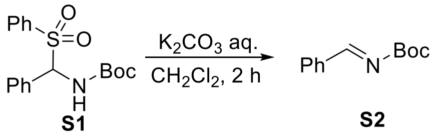

Following the method described by Smith *et al.*,[1] to a stirred solution of potassium carbonate (21.40 g, 53.90 mmol) in water (250 ml) in a 1 l three neck round bottom flask equipped with an overhead stirrer was added a slurry of sulfone (±)-**S1** in CH_2Cl_2 (155 ml). The biphasic reaction mixture was stirred at rt for 2.5 h. The reaction mixture was separated and the aqueous phase was extracted with CH_2Cl_2 (2× 200 ml). The combined organic layers were dried over $MgSO_4$, filtered, and concentrated *in vacuo* to provide imine **S2** as a colourless oil (13.10 g, 63.84 mmol, 97%); ν_{max} (film)/cm^{-1} 2978 (C–H), 1705 (C=O), 1636 (C=N), 1494 (C–N); **^{1}H NMR** (400 MHz, $CDCl_3$) δ_H: 1.54 (9H, s, C(C*H*$_3$)$_3$), 7.54-7.57 (2H, m, ArC(3,5)*H*), 7.62-7.64 (1H, m, ArC(4)*H*), 7.94-7.97 (2H, m, ArC(2,6)*H*), 8.80 (1H, s, C(N)*H*). The data is in agreement with the literature.[1]

Synthesis of *tert*-butyl ((1*S*,2*S*)-2-formyl-3-methyl-1-phenylbutyl)carbamate S3

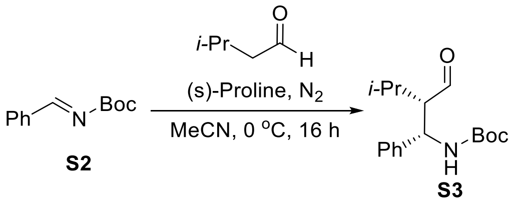

Following the method described by Smith *et al.*,[1] to a stirred solution of imine **S2** (58.00 g, 62.00 mmol) in CH_3CN (300 ml) in a flame dried 500 ml three neck round bottom flask equipped with an overhead stirrer under argon was added isovaleraldehyde (10.4 ml, 0.10 mol). The resulting solution was cooled to 0 °C in an ice bath. (*S*)-proline (1.50 g, 12.40 mmol) was added and the reaction mixture was stirred for 14 h at 0 °C before warming to rt. Water (200 ml), Et_2O (150 ml) and brine (50 ml) were added and the organic layer was separated. The aqueous layer was extracted with Et_2O (2 × 100 ml). The organic extracts were combined, dried ($MgSO_4$), filtered, and concentrated *in vacuo*. Trituration of the resulting solid with hexane (2 × 50 ml) gave aldehyde (1*S*,2*S*)-**S3** (>99:1 dr) as a white solid (11.10 g, 38.13 mmol, 61%); mp 142-145 °C; ν_{max} (film)/cm^{-1} 3385 (N-H), 2972 (C-H), 1717 (C=O),1682 (C=O), 1171 (C-O); **^{1}H NMR** (400 MHz, $CDCl_3$) δ_H: 1.03 (3H, d, *J* 6.9, CH(C*H*$_3$)$_A$(CH$_3$)$_B$), 1.14 (3H, d, *J* 6.9, CH(CH$_3$)$_A$(C*H*$_3$)$_B$), 1.41 (9H, s, C(C*H*$_3$)$_3$), 2.08-2.13 (1H, m, C*H*(C*H*$_3$)$_2$)), 2.47-2.51 (1H, m, C*Hi*-Pr), 4.91-5.11 (2H, m, C*H*Ph and N*H*), 7.20-7.25 (2H, m, ArC(2,6)*H*, 7.25-7.29 (1H, m, ArC(4)*H*), 7.29-7.35 (2H, m, ArC(3,5)*H*), 9.50 (1H, d, *J* 4.2, C(O)*H*). Data in agreement with the literature.[1]

Synthesis of *tert*-butyl ((1*S*,2*S*)-2-(hydroxymethyl)-3-methyl-1-phenylbutyl)carbamate S4

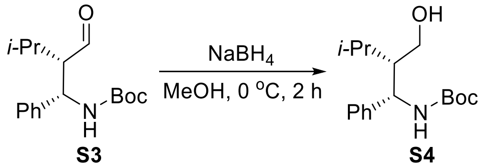

Following the method described by Smith *et al.*,[1] to a slurry of aldehyde (1*S*,2*S*)-**S3** (11.00 g, 37.80 mmol) in methanol (200 ml) at 2 °C in a 500 ml three neck round bottom flask equipped with an overhead stirrer was added $NaBH_4$ (2.20 g, 56.70 mmol) portion-wise over 0.5 h and the reaction mixture was left to stir for 1 h at rt. Saturated aqueous $NaHCO_3$ was added (50 ml) drop-wise over 10 mins forming a white precipitate. The methanol was removed *in vacuo* and water (300 ml) and CH_2Cl_2 (300 ml) was added. The layers were separated, and the aqueous layer was extracted with CH_2Cl_2 (2 × 100 ml). The combined organic extracts were dried over $MgSO_4$, filtered, and concentrated *in vacuo* to give alcohol (1*S*,2*S*)-**S4** as a white solid (11.11 g, 37.86 mmol, 100%); mp 110-114 °C; ν_{max} (film)/cm^{-1} 3395 (O-H, N-H), 2966 (C-H), 1682 (C=O), 1169 (C-O); **^{1}H NMR** (400 MHz, $CDCl_3$) δ_H: 0.84 (3H, d, *J* 6.9, CH(C*H*$_3$)$_A$(CH$_3$)$_B$), 1.02 (3H, d, *J* 6.9, CH(CH$_3$)$_A$(C*H*$_3$)$_B$), 1.43 (9H,

s, C(CH_3)$_3$), 1.72 (1H, s, C*H*(C*H*$_3$)$_2$)), 1.87 (1H, s, C*Hi*-Pr), 3.50 (1H, dd, *J* 11.4, 8.9, C*H*$_A$H$_B$), 3.67 (1H, dd, *J* 11.4, 2.8, CH$_A$*H*$_B$), 5.04 (1H, s, C*H*), 5.78 (1H, d, *J* 9.4, N*H*), 7.23-7.40 (5H, m, PhC(2,3,4,5,6)*H*). The data is in agreement with the literature.[1]

Synthesis of (S)-2-((S)-amino(phenyl)methyl)-3-methylbutan-1-ol hydrochloride S5

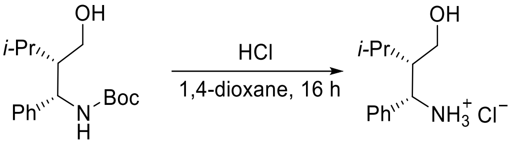

Following the method described by Smith *et al.*,[1] to a slurry of alcohol (1*S*,2*S*)-**S4** (11.50 g, 37.80 mmol) in dioxane (40 ml) was added 4 M HCl in dioxane (82 ml, 3.28 mol) drop-wise over 0.5 h and the reaction mixture was left to stir for 12 h at rt. 150 ml of dioxane were removed *in vacuo* and the solution was cooled to 15 °C to lead to precipitation of a white solid. Et_2O (50 ml) was added and the precipitate was filtered and washed with Et_2O (2 × 50 ml). The resulting white solid was dried (15 mbar, 45 °C) for 2 h to give amino alcohol (1*S*,2*S*)-**S5** as a white solid (6.83 g, 29.73 mmol, 76%); mp 159-163 °C; ν_{max} (film)/cm^{-1} 3310 (O-H, N-H), 2889 (C-H), 1456 (C-N), 1028 (C-O); **^{1}H NMR** (400 MHz, CD_3OD) δ_H: 0.83 (3H, d, *J* 6.8, CH(C*H*$_3$)$_A$(CH$_3$)$_B$), 1.14 (3H, d, *J* 6.7, CH(CH$_3$)$_A$(C*H*$_3$)$_B$, 1.54 (1H, app hept, *J* 6.8, C*H*(CH$_3$)$_A$(CH$_3$)$_B$), 2.00-2.09 (1H, m, C*H* CH(CH$_3$)$_2$), 3.48 (1H, dd, *J* 10.7, 9.7, C*H*$_A$H$_B$), 3.75 (1H, ddd, *J* 10.7, 4.6, 0.8, CH$_A$*H*$_B$), 4.58 (1H, d, *J* 4.2 C*H*Ph), 7.40-7.55 (5H, m, PhC(2,3,4,5,6)*H*). The data is in agreement with the literature.[1]

Synthesis of 2-chloro-6-methoxybenzo[*d*]thiazole S6

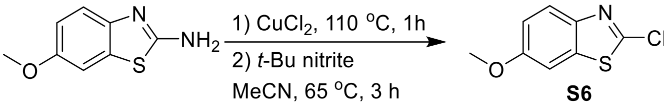

$CuCl_2$ (10.90 g, 80.00 mmol) was added to a round bottomed flask and dried under vacuum at 110 °C for 1 h. After cooling to 40-50 °C, MeCN (400 ml) and *tert*-butyl nitrite (12 ml, 100.80 mmol) were added. A suspension of 6- methoxybenzo[*d*]thiazol-2-amine (12.20 g, 67.20 mmol) in MeCN (12 ml) was added portion-wise using a pipette (N_2 gas evolution). Upon complete addition, the reaction was heated at 65 °C for 3 h. The reaction was cooled to room temperature, poured into 4 M HCl (200 ml), and extracted using Et_2O (2 × 200 ml). The combined organic layers were dried over $MgSO_4$, filtered, and concentrated *in vacuo.* Purification by column chromatography (3% EtOAc/Petrol ether) afforded 2-chloro-6-methoxybenzo[*d*]thiazole **S6** as a colourless solid (9.80 g, 49.09 mmol, 73%); mp 82-84 °C; ν_{max} (film)/cm^{-1} 3063 (C-H), 1599 (C=N), 1493 (C=C), 1009 (C-O); **^{1}H NMR** (400 MHz, $CDCl_3$) δ_H: 3.87 (3H, s, OC*H*$_3$), 7.07 (1H, dd, *J* 9.0, 2.6, ArC(5)*H*), 7.22 (1H, d, *J* 2.6, ArC(7)*H*), 7.82 (1H, d, *J* 9.0, ArC(4)*H*). The data is in agreement with the literature.[2]

Synthesis of (2*S*,3*R*)-3-isopropyl-8-methoxy-2-phenyl-3,4-dihydro-2*H*-benzo[4,5]thiazolo [3,2-*a*]pyrimidine S8

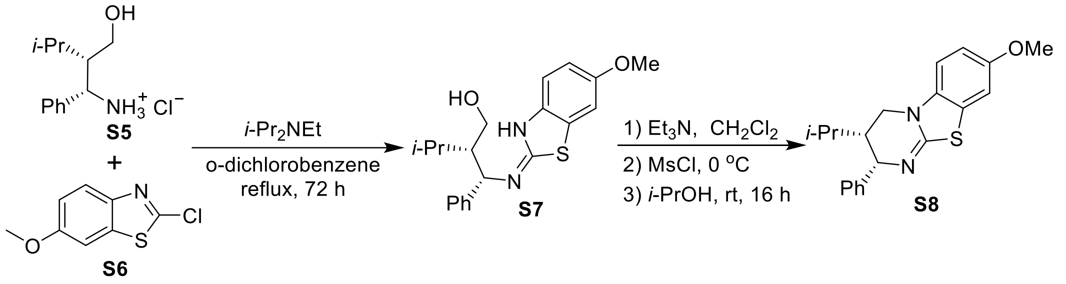

Following the method described by Smith *et al.*,[1] to a slurry of (1*S*,2*S*)-**S5** (4.80 g, 21.00 mmol) in chlorobenzene (7.5 ml) was added *i*-Pr_2NEt (10.5 ml, 60.00 mmol), 2-chloro-6-methoxybenzo[*d*]thiazole **S6** (4.00 g, 20.00 mmol) and the mixture heated to reflux for 70 h. The reaction mixture was cooled to rt, washed with water (2 × 100 ml), dried over $MgSO_4$, filtered, and concentrated *in vacuo*. The resulting solid was stirred in hexane (250 ml) for 15 mins and then filtered to provide 5.20 g alcohol (1*S*,2*S*)-**S7** as a white solid. To a slurry of 5.20 g crude amino alcohol **S7** in anhydrous CH_2Cl_2 (80 ml) was added Et_3N (8.5 ml, 60.80 mmol) and the reaction mixture was cooled to 0 °C. Methanesulfonyl chloride (1.5 ml, 19.80 mmol) was added and the reaction mixture was stirred at room temperature for 0.5 h. Once complete consumption of the amino alcohol was observed, *i*-PrOH (1 ml) was added and the reaction was heated at reflux for 16 h. The reaction was quenched with 1 M aq. NaOH (20 ml) and the biphasic mixture stirred vigorously for 0.5 h. The layers were separated and the aqueous layer was extracted with CH_2Cl_2 (3 × 20 ml). The combined organic fractions were washed with brine (50 ml), dried over $MgSO_4$, filtered, and concentrated *in vacuo* to afford the crude product. Further purification by column chromatography (15%-40% EtOAc/CH_2Cl_2) afforded an off-white solid that was recrystallised from 50% EtOAc in Hexane to give (2*S*,3*R*)-3-isopropyl-8-methoxy-2-phenyl-3,4-dihydro-2Hbenzo[4,5]thiazolo[3,2-a]pyrimidine **S8** as a colourless solid (3.43 g, 10.13 mmol, 51%); mp 127 °C; ν_{max} (film)/cm^{-1} 2934 (C-H), 1602 (C=N), 1467 (C=C), 1022 (C-O); $[\alpha]_D^{20}$ 309.3 (c 1.0 in $CHCl_3$); **1H NMR** (400 MHz, $CDCl_3$) δ_H: 0.84 (1H, d, *J* 6.6, $CH(CH_3)_A(CH_3)_B$), 1.13 (1H, d, *J* 6.6, $CH(CH_3)_A(CH_3)_B$), 1.23-1.35 (1H, m, C*H*$(CH_3)_2$), 1.89-2.00 (1H, m, C*Hi*-Pr), 3.33 (1H, app t, *J* 11.4, $CH_{ax}H_{eq}$), 3.77-3.88 (4H, m, OCH_3, $CH_{ax}H_{eq}$), 4.91 (1H, dd, *J* 4.4, 1.7, C*H*Ph), 6.72 (1H, d, *J* 8.7, ArC(6)*H*), 6.79 (1H, dd, *J* 8.7, 2.5, ArC(7)*H*), 6.95 (1H, d, *J* 2.5, ArC(9)*H*), 7.17-7.34 (5H, m, PhC(2,3,4,5,6)*H*). The data is in agreement with the literature.[3]

Synthesis of (2*S*,3*R*)-8-hydroxy-3-isopropyl-2-phenyl-3,4-dihydro-2H-benzo[4,5] thiazolo[3,2-a]pyrimidin-1-ium bromide S9

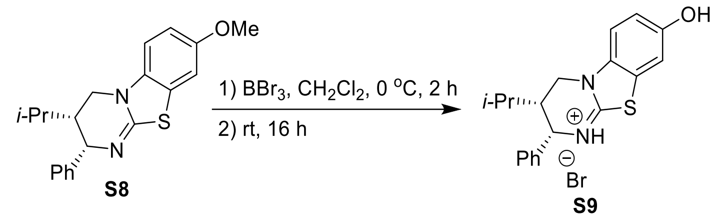

Following the method described by Smith *et al.*,[3] BBr_3 (18 ml, 18.00 mmol, 1 M in Hexane) was added dropwise to a solution of (2*S*,3*R*)-3-isopropyl-8-methoxy-2-phenyl-3,4-dihydro-2*H*-benzo[4,5]thiazolo[3,2-*a*]pyrimidine **S8** (0.60 g, 1.80 mmol) in CH_2Cl_2 (12 ml) at 0 °C. The solution was stirred at 0 °C for 2 h then warmed to room temperature and stirred for 16 h. The reaction was carefully quenched with MeOH (10 ml). MeOH was removed *in vacuo*, then CH_2Cl_2 (10 ml) was added and the aqueous phase was extracted with CH_2Cl_2 (3 × 20 ml). The organic layers were combined, dried over $MgSO_4$, filtered, and concentrated *in vacuo* to afford (2*S*,3*R*)-8-hydroxy-3-isopropyl-2-phenyl-3,4-dihydro-2*H*-benzo[4,5]thiazolo[3,2-*a*]pyrimidin-1-ium bromide **S9** as a colourless solid (0.69 g, 1.71 mmol, 95%); mp 198-201 °C; ν_{max} (film)/cm^{-1} 3159 (O-H), 2930 (C-H), 1599 (C=N), 1454 (C=C); **^{1}H NMR** (400 MHz, d_6-DMSO) δ_H: 0.79 (3H, d, *J* 6.7, CH(C*H*$_3$)$_A$(CH$_3$)$_B$), 1.07 (3H, d, *J* 6.5, CH(CH$_3$)$_A$(C*H*$_3$)$_B$), 1.14-1.31 (1H, m, C*H*(CH$_3$)$_2$), 2.25-2.38 (1H, m, C*Hi*-Pr), 3.68 (1H, m, C*H*$_{ax}$H$_{eq}$), 4.40 (1H, dd, *J* 13.3, 4.9, CH$_{ax}$*H*$_{eq}$), 5.17 (1H, d, *J* 4.8, C*H*Ph), 6.99 (1H, dd, *J* 8.8, 2.5, ArC(7)*H*), 7.26-7.34 (2H, m, PhC(2,6)*H*), 7.36-7.43 (3H, m, PhC(3,4,5)*H*), 7.46 (1H, d, *J* 2.5, ArC(9)*H*), 7.65 (1H, d, *J* 8.8, ArC(6)*H*), 9.98 (1H, s, O*H*), 11.14 (1H, s, N*H*). The data is in agreement with the literature.[3]

Synthesis of (2*S*,3*R*)-3-isopropyl-2-phenyl-8-(prop-2-yn-1-yloxy)-3,4-dihydro-2*H* benzo[4,5]thiazolo[3,2-*a*]pyrimidine S10

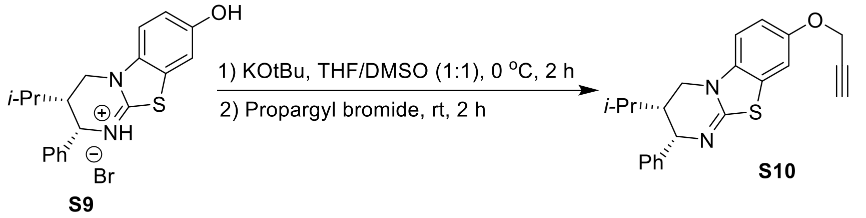

Following the method described by Smith *et al.*,[3] KO*t*-Bu (395.00 mg, 3.53 mmol) was added to a solution of (2*S*,3*R*)-8-hydroxy-3-isopropyl-2-phenyl-3,4-dihydro-2*H*-benzo[4,5]thiazolo[3,2-*a*]pyrimidin-1-ium bromide **S9** (0.55 g, 1.36 mmol) in THF/DMSO (1:1, 10 ml) at 0 °C, and the reaction mixture was stirred for 2 h. Propargyl bromide (227 µl, 2.04 mmol, 80% in toluene) was added and the reaction mixture was allowed to warm to r.t. The reaction was quenched with brine (10 ml) and the layers were separated. The aqueous layer was extracted with EtOAc (3 × 10 ml). The combined organic layers were washed with brine, dried over $MgSO_4$, filtered and concentrated *in vacuo.* The residue was purified by column chromatography (5%-20%

EtOAc/Petrol ether) to afford (2*S*,3*R*)-3-isopropyl-2-phenyl-8-(prop-2-yn-1-yloxy)-3,4-dihydro-2*H*-benzo[4,5]thiazolo[3,2-*a*]pyrimidine **S10** as a colourless solid (0.38 g, 1.05 mmol, 77%); mp 61-62 °C; ν_{max} (film)/cm^{-1} 2965 (C-H), 2862 (C-H), 1618 (C=N), 1483 (C=C), 1171 (C-O); $[\alpha]_D^{20} = +305\ (c\ 1.0\ CHCl_3)$); **^{1}H NMR** (400 MHz, $CDCl_3$) δ_H: 0.84 (3H, d, *J* 6.7, CH(C*H*$_3$)$_A$(CH$_3$)$_B$), 1.13 (3H, d, *J* 6.5, CH(CH$_3$)$_A$(C*H*$_3$)$_B$), 1.22–1.35 (1H, m, C*H*(CH$_3$)$_2$), 1.94 (1H, app ddt, *J* 11.3, 9.4, 4.9, C*Hi*-Pr), 2.54 (1H, app t, *J* 2.4, C≡C*H*), 3.33 (1H, app t, *J* 11.5, C*H*$_{ax}$H$_{eq}$), 3.83 (1H, ddd, *J* 11.5, 5.2, 1.8, CH$_{ax}$*H*$_{eq}$), 4.68 (2H, app d, *J* 2.4, C*H*$_2$O), 4.91 (1H, dd, *J* 4.5, 1.7, C*H*Ph), 6.73 (1H, d, *J* 8.7, ArC(6)*H*), 6.88 (2H, dd, *J* 8.7, 2.5, ArC(7)*H*), 7.04 (1H, d, *J* 2.5, ArC(9)*H*), 7.17-7.33 (5H, m, PhC(2,3,4,5,6)*H*). The data is in agreement with the literature.[3]

Synthesis of azide-functionalised Merrifield resin S11

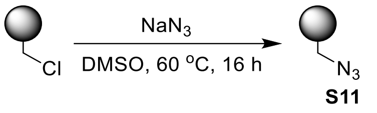

Following the method described by Smith *et al.*,[3] (chloromethyl)polystyrene resin (3.00 g, *f* = 1.30 mmol·g^{-1}) was added to NaN_3 (0.78 g, 51.00 mmol) in DMSO (30 ml). The mixture was heated at 60 °C (without stirring) for 16 h and then cooled to r.t. The suspension was filtered and washed sequentially with H_2O (500 ml), THF-MeOH 1:1 (250 ml), MeOH (250 ml) and THF (250 ml). The resulting solid was dried *in vacuo* for 24 h at 40 °C to afford (azidomethyl)polystyrene **S11** (12.60 g); IR ν_{max} (solid, cm^{-1}) 2094 (N_3); Elemental analysis (%) C 85.05 H 7.54, N 5.30; *f* = 1.26 mmol·g^{-1}.

Synthesis of Merrifield resin-supported (2S,3R)-HyperBTM derivative Cat (2*S*,3*R*)-4

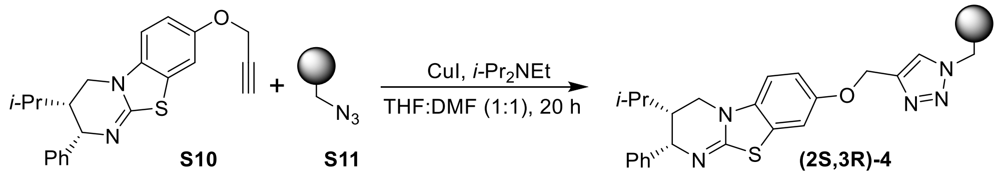

Following the method described by Smith *et al.*,[3] (2*S*,3*R*)-3-isopropyl-2-phenyl-8-(prop-2-yn-1-yloxy)-3,4-dihydro-2*H*-benzo[4,5]thiazolo[3,2-*a*]pyrimidine **S10** (0.41 g, 1.13 mmol), *i*-Pr$_2$NEt (626 µl, 3.78 mmol) and CuI (9.84 mg, 0.06 mmol, 5 mol%) were added to a suspension of (azidomethyl)polystyrene **S11** (0.86 g, 1.08 mmol, *f* = 1.26 mmol·g^{-1}) in THF:DMF 1:1 (52 ml) with slow stirring (200 rpm). The reaction mixture was stirred until disappearance of the azide band (~2094 cm^{-1}) was confirmed by IR. The suspension was filtered and washed sequentially with THF (1:1, 200 ml), H_2O (200 ml), H_2O-MeOH (1:1, 200 ml), MeOH (200 ml), MeOH-THF (1:1, 200 ml), THF (200 ml) and CH_2Cl_2 (200 ml) and the resin was dried *in vacuo* at 40 °C for 24 h to afford a pale brown resin (2*S*,3*R*)-8-((1-ethyl-1*H*-1,2,3-triazol-4-yl)methoxy)-3-isopropyl-2-phenyl-3,4-dihydro-2*H*-benzo[4,5]thiazolo[3,2-*a*] pyrimidine on polymer support (2*R*,3*S*)-**4** (0.90 g, 62%); Elemental analysis (%) C 82.33, H 6.86, N 5.49; *f* = 0.78 mmol·g^{-1}.

2.2 General Procedure B: Synthesis of azaaryl ketone

Synthesis of 2-phenacylbenzothiazole S12

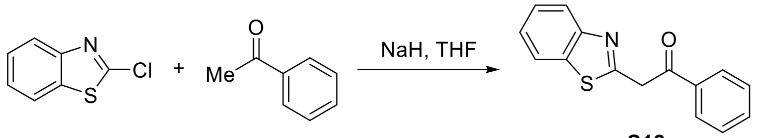

Following the method described by Smith *et al.*,[4] acetophenone (9.3 ml, 80.00 mmol) was added slowly to a suspension of sodium hydride (60% in mineral oil, 4.80 g, 120.00 mmol) in dry THF (60 ml) under argon and the flask stirred for 10 minutes. 2-Chlorobenzothiazole (12.5 ml, 96.00 mmol) was added dropwise and the reaction heated at reflux for 15 hours. The reaction was quenched by dropwise addition of water at 0 °C, the acidified to pH 1-2 using a solution of 1 M HCl. The mixture was diluted with ethyl acetate (150 ml) and then washed sequentially with water (2 × 100 ml) and saturated $NaHCO_3$ solution (100 ml). The organic layer was dried over anhydrous $MgSO_4$, filtered, and concentrated *in vacuo*. The crude solid was recrystallized from toluene to give azaaryl ketone **S12** as a yellow solid (11.10 g, 43.82 mmol, 54%); mp 111-113 °C; ν_{max} (film)/cm^{-1} 3046 (C-H), 1616 (C=N), 1450 (C=C), 1020 (C-O); analysed as a 55:45 mixture of tautormers: **^{1}H NMR** (400 MHz, $CDCl_3$) δ_H: 4.86 (1.1H, s, *keto*-C*H*$_2$-CO), 6.40 (0.9H, s, *enol*-C*H*=COH), 7.33 (0.93H, t, *J* 7.6, *enol*-benzothiazoleC(6)*H*), 7.41 (0.6H, t, *J* 7.6, *enol*-benzothiazoleC(6)*H*), 7.42–7.52 (4.1H, m, Ar*H*), 7.53 (1H, m, *enol*-benzothiazoleC(4)*H*), 7.64 (0.6H, app t, *J* 7.4, *keto*-benzothiazoleC(5)*H*), 7.81 (0.9H, d, *J* 8.0, *enol*-benzothiazoleC(4)*H*), 7.85 (0.9H, d, *J* 8.0, *enol*-benzothiazoleC(7)*H*)), 7.91 (2.4H, m, *keto*-benzothiazoleC(4)*H*, *enol*-phenacylC(2′)*H*), 8.04 (0.6H, d, *J* 8.0, *keto*-benzothiazoleC(7)*H*), 8.09-8.14 (1.1H, m, *J* 7.5 *keto*-phenacylC(2′)*H*). The data is in agreement with the literature.[4]

Synthesis of 2-phenacylbenzoxazole S13

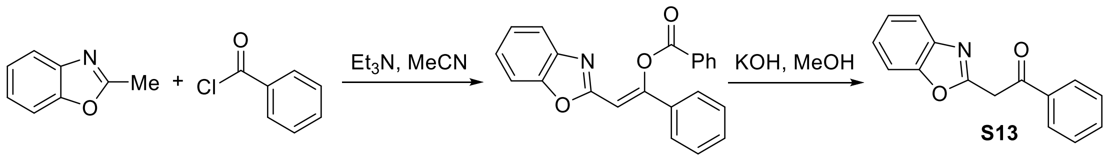

Following the method described by Smith *et al.*,[5] to a solution of 2-methylbenzoxazole (4.8 ml, 40.00 mmol) in MeCN (200 ml) was added benzoyl chloride (20.4 ml, 120 mmol) and Et_3N (20 ml, 144.00 mmol). The solvent was removed *in vacuo*. The residue was dissolved in CH_2Cl_2 (100 ml) and the organic layer was washed with saturated aqueous $NaHCO_3$ solution, dried over $MgSO_4$, filtered, and concentrated *in vacuo* to give the ester as a brown oil that was used without further purification. The crude ester was dissolved in methanol (100 ml), solid KOH (5.60 g, 0.10 mol) was added portionwise and the flask was stirred at room temperature for 20 h. The solvent was removed *in vacuo* and the residue was dissolved in CH_2Cl_2. The mixture was acidified using 2 M HCl. The aqueous layer was separated and the organic layer washed with saturated

aqueous $NaHCO_3$, dried over $MgSO_4$, filtered and concentrated *in vacuo*. The crude was purified by column chromatography (5% EtOAc/Petrol ether) followed by recrystallisation from ethanol to give the azaaryl ketone **S13** as an off-white solid (6.90 g, 29.08 mmol, 73%); mp 95-98 °C; ν_{max} (film)/cm^{-1} 1624 (C=O), 1614 (C=N), 1450 (C=C), 1067 (C-O); analysed as 50:50 mixture of tautormers: **^{1}H NMR** (400 MHz, $CDCl_3$) δ_H: 4.66 (1H, s, *keto*-C*H*$_2$-CO), 6.22 (0.5H, s, *enol*-C*H*=COH), 7.27–7.39 (2H, m, Ar*H*), 7.41–7.56 (3.5H, m, Ar*H*), 7.62 (2H, m, Ar*H*), 7.72 (0.5H, m, Ar*H*), 7.89 (1H, m, Ar*H*), 8.01–8.09 (1H, m, PhC(2)*H*). Data in agreement with the literature.[5]

Synthesis of 2-phenacylthiazole S14

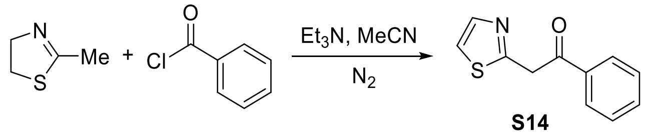

Following the method described by Smith *et al.*,[6] triethylamine (5.0 ml, 36.00 mmol) was added to a solution of a 2-methylthiazole (0.9 ml, 10.00 mmol) and benzoyl chloride (3.5 ml, 30.00 mmol) in MeCN (0.3 M) at r.t. under a nitrogen atmosphere. The reaction was heated at reflux overnight. The reaction was cooled to r.t. and MeCN was removed *in vacuo*. The residue was dissolved in CH_2Cl_2 and washed with saturated aqueous $NaHCO_3$ and brine, dried over $MgSO_4$, filtered, and concentrated *in vacuo*. The residue was dissolved in the prepared KOH (25.00 mmol)/MeOH (40 ml) solvent and the reaction stirred overnight. The solvent was removed, the residue dissolved in EtOAc and washed sequentially with 1 M HCl twice, saturated aqueous $NaHCO_3$ and brine, dried over $MgSO_4$, filtered, and concentrated *in vacuo*. Further purification by column chromatography (5% EtOAc/Petrol ether) gave the azaaryl ketone **S14** as a green oil (1.82 g, 8.95 mmol, 89%); ν_{max} (film)/cm^{-1} 2922 (C-H), 1682 (C=O), 1595 (C=N), 1449 (C=C), 1354 (C-S), 1201 (C-O); analysed as a 80:20 mixture of tautomers: **^{1}H NMR** (400 MHz, $CDCl_3$) δ_H: 4.77 (1.6H, s, *keto*-C*H*$_2$-CO), 6.37 (0.4H, s, *enol*-C*H*=COH), 7.08 (0.4H, d, *J* 3.4, *enol*-thiazoleC*H*), 7.34 (0.8H, d, *J* 3.4, *keto*-thiazoleC*H*), 7.38–7.46 (1.2H, m, PhC(4)*H*), 7.47-7.55 (1.6H, m, *keto*-PhC(3,5)*H*), 7.58-7.64 (0.8H, m, *enol*-PhC(3,5)*H*), 7.69 (0.4H, d, *J* 3.4, *enol*-thiazoleC*H*), 7.79 (0.8H, d, *J* 3.4, *keto*-thiazoleC*H*), 7.82-7.87 (0.8H, d, *J* 8.0, 0.8H, m, *enol*-PhC(2,6)*H*), 8.05-8.12 (1.6H, d, *J* 8.0, 0.8H, m, *keto*-PhC(2,6)*H*).The data is in agreement with the literature.[6]

Synthesis of 2-phenacylbenzimidazole S15

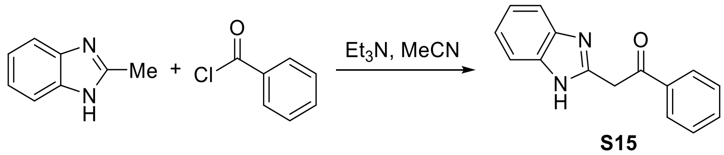

Following the method described by Smith *et al.*,[7] to a solution of 2- methylbenzimidazole (2.64 g, 20.00 mmol) in MeCN (20 ml) was added benzoyl chloride (7.0 ml, 60.00 mmol)) and Et_3N (8.4 ml, 60.00 mmol) to give the

ester as a yellow solid which was used immediately. Heating the ester at reflux in isopropylalcohol (10 ml) for 3 h gave the azaaryl ketone **S15** after filtration as a yellow solid (2.78 g, 11.77 mmol, 59%); mp 177-180 °C; ν_{max} (film)/cm^{-1} 3053 (C-H), 1614 (C=O), 1593 (C=N), 1452 (C=C), 1076 (C-O); analysed as a 50:50 mixture of tautomers: **^{1}H NMR** (400 MHz, DMSO-d_6) δ_H: 4.69 (1H, s, *keto*-CH_2-CO), 6.11 (0.5H, s, *enol*-C*H*=COH), 7.12–7.21 (2H, m, benzimidazoleC(5)*H* and benzimidazoleC(6)*H*), 7.38–7.60 (4.5H, m, ArC*H*), 7.62–7.72 (0.5H, m, *keto*-phenylC(4)*H*), 7.83–7.92 (1H, m, 2 *enamino*-phenylC(2)*H*), 8.04–8.13 (1H, m, 2×*keto*-phenylC(2)*H*), 12.31 (0.5H, s, N*H*). The data is in agreement with the literature.[7]

Synthesis of 2- dimethylformylbenzothiazole S16

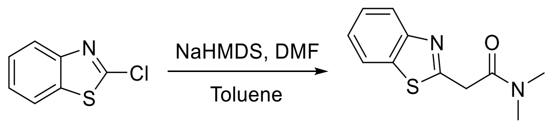

S16

Following the method described by Smith *et al.*,[7] To a degassed solution of 2-Chlorobenzothiazole (1.0 ml, 8.00 mmol) and DMF (2.3 ml, 24.00 mmol) in dry toluene (30 ml) was added NaHMDS (12.6 ml, 1.9 M solution in THF) dropwise at 0 °C, and the solution stirred for 5 h at 0 °C followed by room temperature for 16 h. Excess NaHMDS was quenched by dropwise addition of saturated aqueous NH_4Cl (50 mL) at 0 °C. The organic layer was separated, then the aqueous layer extracted with EtOAc (2 × 50 ml). The organic layer was dried over anhydrous $MgSO_4$, filtered, and concentrated *in vacuo* to give azaaryl ketone **S16** as a white solid (0.95 g, 4.32 mmol, 54%); mp 94-95 °C; ν_{max} (film)/cm^{-1} 1643 (C=O), 1524 (C=N), 1124 (C-N); **^{1}H NMR** (400 MHz, DMSO-d_6) δ_H: 3.03 (3H, s, NCH_3CH$_3$), 3.16 (3H, s, NCH$_3$CH_3), 4.26 (2H, s, CH_2-CO), 7.39 (1H, ddd, *J* 8.3, 7.3, 1.2, benzothiazoleC(6)*H*), 7.47 (1H, ddd, *J* 8.3, 7.2, 1.2, benzothiazoleC(5)*H*), 7.88 (1H, ddd, *J* 8.0, 1.3, 0.6, benzothiazoleC(4)*H*), 7.99 (1H, ddd, *J* 8.1, 1.3, 0.7, benzothiazoleC(7)*H*).. The data is in agreement with the literature.[7]

2.3 General Procedure C: Synthesis of α,β-unsaturated homoanhydrides

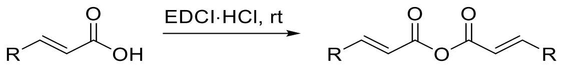

To a solution of carboxylic acid (1.4 equiv.) in CH_2Cl_2 or THF as specified (0.8 M) was added 1-ethyl-3-(3-dimethylaminopropyl)carbodiimide·HCl (EDCI·HCl) (1.0 equiv.) and the solution was stirred for 1-2 h at room temperature. The solution was diluted with CH_2Cl_2 (50 ml) and then washed sequentially with water (2 × 50 ml) and saturated aqueous $NaHCO_3$ solution (50 ml). The organic layer was dried over anhydrous $MgSO_4$, filtered, and concentrated *in vacuo* to afford the homoanhydride.

(*E*)-Cinnamic anhydride S17

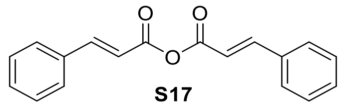

S17

The title compound was prepared according to *General Procedure B* from (*E*)-cinnamic acid (10.00 g, 67.50 mmol) and EDCI·HCl (12.94 g, 67.50 mmol) in CH_2Cl_2 (80 ml) to give the *homoanhydride* **S17** as a white solid (5.73 g, 20.59 mmol, 61%); mp 118-119 °C; ν_{max} (film)/cm^{-1} 1697 (C=O), 1450 (C=C), 1067 (C-O); **^{1}H NMR** (400 MHz, $CDCl_3$) δ_H: 6.54 (2H, d, *J* 16.0, ArCH=C*H*CO), 7.40–7.47 (6H, m, PhC*H*), 7.54–7.63 (4H, m, PhC*H*), 7.86 (2H, d, *J* 16.0, ArC*H*=CHCO). The data is in agreement with the literature.[8]

(*E*)-3-(4-Fluorophenyl)acrylic anhydride S18

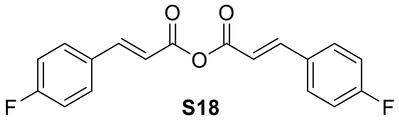

S18

The title compound was prepared according to *General Procedure B* from fluorophenyl)acrylic acid (1.66 g, 10.00 mmol) and EDCI·HCl (0.96 g, 10.00 mmol) in CH_2Cl_2 (4 ml) to give the *homoanhydride* **S18** as a white solid (1.07 g, 3.40 mmol, 68%); mp 88-91 °C; ν_{max} (film)/cm^{-1} 1699 (C=O), 1595 (C=C), 1128 (C-O); **^{1}H NMR** (400 MHz, $CDCl_3$) δ_H: 6.45 (2H, d, *J* 16.1, ArCH=C*H*CO), 7.07-7.16 (4H, m, ArC*H*), 7.54-7.61 (4H, m, ArC*H*), 7.82 (2H, d, *J* 16.1, ArC*H*=CHCO). The data is in agreement with the literature.[8]

(*E*)-3-(4-(Trifluoromethyl)phenyl)acrylic anhydride S19

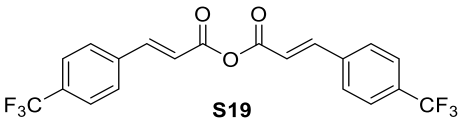

S19

The title compound was prepared according to *General Procedure B* from 4- trifluoromethylcinnamic acid (2.16 g, 10.00 mmol) and EDCI·HCl (1.92 g, 10.00 mmol) in CH_2Cl_2 (10 ml) to give the *homoanhydride* **S19** as a white solid (1.21 g, 2.92 mmol, 58%); mp 127-130 °C; ν_{max} (film)/cm^{-1} 1705 (C=O), 1418 (C=C), 1321 (CF_3), 1109 (C-O); **^{1}H NMR** (400 MHz, $CDCl_3$) δ_H: 6.60 (2H, d, *J* 16.0, ArCH=C*H*CO), 7.69 (8H, s, ArC*H*), 7.88 (2H, d, *J* 16.0, ArC*H*=CHCO). The data is in agreement with the literature.[8]

(*E*)-3-(4-Methoxyphenyl)acrylic 3-(4-methoxyphenyl)propanoic anhydride S20

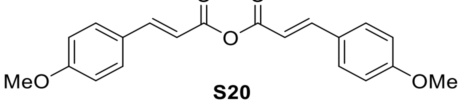

S20

The title compound was prepared according to *General Procedure B* from 4-methoxycinnamic acid (1.78 g, 10.00 mmol) and EDCI·HCl (1.92 g, 10.00 mmol) in THF (20 ml) to give the *homoanydride* **S20** as a white solid (0.89 g, 2.63 mmol, 53%); mp 118-122 °C; ν_{max} (film)/cm^{-1} 1717 (C=O), 1597 (C=C), 1254 (C-O); **^{1}H NMR** (400 MHz, $CDCl_3$) δ_H: 3.85 (6H, s, ArOC*H*$_3$), 6.38 (2H, d, *J* 15.8, ArCH=C*H*CO), 6.93 (4H, d, *J* 8.7, ArC*H*), 7.53 (4H, d, *J* 8.7, ArC*H*), 7.80 (2H, d, *J* 15.8, ArC*H*=CHCO). The data is in agreement with the literature.[8]

(*E*)-3-(Furan-2-yl)acrylic anhydride S21

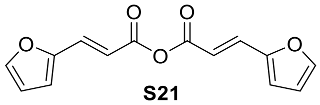

S21

The title compound was prepared according to *General Procedure B* from furylacrylic acid (1.38 g, 10.00 mmol) and EDCI·HCl (1.15 g, 10.00 mmol) in THF (10 ml) to give the *homoanhydride* **S21** as a brown solid (1.04 g, 4.03 mmol, 81%); mp 69-72 °C; ν_{max} (film)/cm^{-1} 1773 (C=O), 1625 (C=O), 1473 (C=C); **^{1}H NMR** (400 MHz, $CDCl_3$) δ_H: 6.36 (2H, d, *J* 15.7, furanylCH=C*H*CO), 6.51 (2H, dd, *J* 3.5, 1.7, furanylC(4)*H*), 6.73 (2H, d, *J* 3.5, furanylC(3)*H*), 7.55-7.60 (4H, m, furanylC(5)*H*, furanylC*H*=CHCO). The data is in agreement with the literature.[8]

(*E*)-3-(Thien-2-yl)acrylic anhydride S22

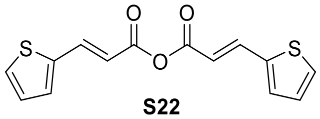

S22

The title compound was prepared according to *General Procedure B* from thienylacrylic acid (1.54 g, 10.00 mmol) and EDCI·HCl (1.15 g, 10.00 mmol) in THF (10 ml) to give the *homoanhydride* **S22** as a brown solid (1.12 g, 3.86 mmol, 77%); mp 84-87 °C; ν_{max} (film)/cm^{-1} 1676 (C=O), 1612 (C=O), 1410 (C=C), 1238 (C-S-C), 1043 (C-O); **^{1}H NMR** (400 MHz, $CDCl_3$) δ_H: 6.30 (2H, d, *J* 15.6, thienylCH=C*H*CO), 7.10 (2H, dd, *J* 5.1, 3.7, thienylC(4)*H*), 7.36 (2H, d, *J* 3.7, thienylC(3)*H*), 7.48 (2H, d, *J* 5.0, 1.0, thienylC(5)*H*), 7.94 (2H, d, *J* 15.6, thienylC*H*=CHCO). Data in agreement with the literature.[8]

(*E*)-3-(3-Methylphenyl)acrylic anhydride S23

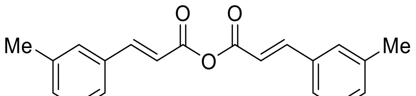

S23

The title compound was prepared according to *General Procedure B* from 3-methylcinnamic acid (0.81 g, 5.00 mmol) and EDCI·HCl (576.00 mg, 3.00 mmol) in CH_2Cl_2 (10 ml) to give the *homoanhydride* **S23** as a white solid (0.5 g, 1.63 mmol, 65%); mp 58-59 °C; ν_{max} (film)/cm^{-1} 2916 (C-H), 1697 (C=O), 1628 (C=O), 1584 (C=C), 1128

(C-O); **^{1}H NMR** (400 MHz, $CDCl_3$) δH: 2.39 (6H, s, C*H*$_3$), 6.52 (2H, d, *J* 15.9, ArCH=C*H*CO), 7.16-7.42 (8H, m, ArC*H*), 7.83 (2H, d, *J* 15.9, ArC*H*=CHCO). The data is in agreement with the literature.[8]

(*E*)-3-(2-Chlorophenyl)acrylic anhydride S24

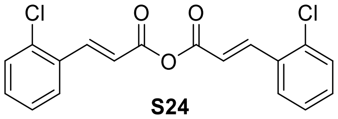

S24

The title compound was prepared according to *General Procedure B* from 2-chlorocinnamic acid (1.83 g, 10.00 mmol) and EDCI·HCl (1.15 g, 10.00 mmol) in CH_2Cl_2 (20 ml) to give the *homoanhydride* **S24** as a white solid (0.95 g, 2.74 mmol, 55%); mp 142-145 °C; ν_{max} (film)/cm^{-1} 1705 (C=O), 1440 (C=C), 1136 (C-O), 1117 (C-Cl); **^{1}H NMR** (400 MHz, $CDCl_3$) δ_H: 6.54 (2H, d, *J* 16.0, ArCH=C*H*CO), 7.30-7.40 (4H, m, ArC*H*), 7.44-7.47 (2H, m, ArC(5)*H*), 7.69 (2H, dd, *J* 7.6, 1.8, ArC(6)*H*), 8.30 (2H, d, *J* 16.0, ArC*H*=CHCO). The data is in agreement with the literature.[8]

(*E*)-But-2-enoic anhydride S25

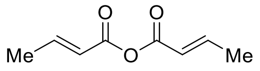

S25

The title compound was prepared according to *General Procedure B* from crotonic acid (0.86 g, 10.00 mmol) and EDCI·HCl (1.15 g, 10.00 mmol) in CH_2Cl_2 (20 ml) to give the *homoanhydride* **S25** as a colourless oil (0.53 g, 3.44 mmol, 69%); ν_{max} (film)/cm^{-1} 1776 (C=O), 1647 (C=O), 1442 (C=C), 1062 (C-O); **1H NMR** (400 MHz, $CDCl_3$) δ_H: 1.95 (6H, dd, *J* 7.0, 1.7, C*H*$_3$), 5.91 (2H, dq, *J* 15.5, 1.7, CH_3CH=C*H*CO), 7.14 (2H, dq, *J* 15.5, 6.9, CH_3C*H*=CHCO). The data is in agreement with the literature.[8]

(*E*)-3-(4-Methylphenyl)acrylic anhydride S26

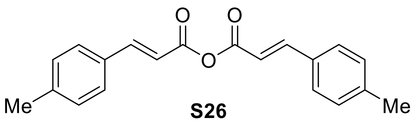

S26

The title compound was prepared according to *General Procedure B* from 4-methylcinnamic acid (1.62 g, 10.00 mmol) and EDCI·HCl (1.15 g, 10.00 mmol in THF (20 ml) to give *homoanydride* **S26** as a white solid (0.99 g, 3.23 mmol, 65%); mp 124-126 °C; ν_{max} (film)/cm^{-1} 3075 (C-H), 1699 (C=O), 1512 (C=C), 1126 (C-O); **^{1}H NMR** (400 MHz, $CDCl_3$) δH: 2.42 (6H, s, C*H*$_3$), 6.51 (2H, d, *J* 15.9, ArCH=C*H*CO), 7.23-7.31 (4H, m, ArC*H*), 7.47-7.54 (4H, m, ArC*H*), 7.85 (2H, d, *J* 15.9, ArC*H*=CHCO). The data is in agreement with the literature.[9]

(*E*)-3-(naphthalen-1-yl)acrylic anhydride S27

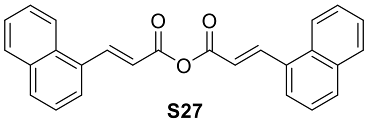

S27

The title compound was prepared according to *General Procedure B* from naphthalen-1-ylcinnamic acid (1.98 g, 10.00 mmol) and EDCI·HCl (1.15 g, 10.00 mmol) in THF (20 mL) to give the *homoanydride* **S27** as a white solid (1.10 g, 2.91 mmol, 58%); mp 92-96 °C; ν_{max} (film)/cm^{-1} 1708 (C=O), 1510 (C=C), 1464 (C=C), 1167 (C-O); **1H NMR** (400 MHz, $CDCl_3$) δH: 6.70-6.69 (2H, d, *J* 15.6, ArCH=C*H*CO), 7.53-7.58 (4H, m, Ar*H*), 7.61-7.64 (2H, m, Ar*H*), 7.87 (2H, d, *J* 8.1, Ar*H*), 7.91 (2H, d, *J* 8.1, Ar*H*), 7.98 (2H, d, *J* 8.1, Ar*H*), 8.23 (2H, d, *J* 8.2, Ar*H*), 8.77 (2H, d, *J* 15.7, ArC*H*=CHCO). Data in agreement with the literature.[10]

2.4 General Procedure D, Batch Procedure E and Scale-up Procedure F: Asymmetric annulations of α,β-unsaturated homo-anhydrides and aza-aryls.

General Procedure D: For the details of the reaction set in continuous flow, a packed bed reactor consisting of a vertically-mounted Omnifit glass chromatography column (10 mm pore size and up to maximal 70 mm of adjustable bed height), with a glass cooling jacket was loaded with PS-HyperBTM (2*S*,3*R*)-**4** (700 mg; *f* = 0.78 mmol·g^{-1}). Reaction solvent (THF : CH_2Cl_2 = 2:1 mixture) was pumped through the packed bed reactor at 0.5 ml·min^{-1} by 305 HPLC pump developed by Gilson for 30 min. And the column was then cooled by circulting ethylene glycol (0 °C) using a Julabo F250 over 10 min. Two syringes (10.0 ml 1010LT type of syringe developed by Hamilton) were used to inject reagents using a SPLG200 series syringe pump developed by WPI. Solutions of 2-phenacylbenzothiazole (0.060 M = 1.0 equiv.) and *i*-Pr_2NEt (0.036 M = 0.6 equiv.) in one syringe and cinnamic anhydride (0.060 M = 1.0 equiv.) in another syringe were delivered to the column *via* a mixing T-piece using a syringe pump with a combined flowrate of 0.1 mL·min^{-1} (total flow time, 40 min), the flow passed column from the bottom to the top. After complete addition of the reagents from the syringes, the HPLC pump was connected, and pumped reaction solvent (THF : CH_2Cl_2 = 2:1 mixture) at 0.1 mL·min^{-1} for 45 min to ensure elution of the products. When different reactions were carried out, an additional solution of 10% MeOH in $CHCl_3$ was pumped at 0.5 ml·min^{-1} for 30 min to regenerate catalyst.

Batch Procedure E: **S12** (0.10 mmol, 1.0 equiv.) and anhydride **S17** (0.10 mmol, 1.0 equiv.) was dissolved in THF : CH_2Cl_2 = 2:1 mixture (0.5ml) and the solution cooled to 0 °C. PS-HyperBTM (2*S*,3*R*)-**4** (35 mol%, 45 mg) and *i*-Pr_2NEt (0.06 mmol, 0.6 equiv.) were added and the solution stirred at 0 °C for 16 h. After reaction, PS-HyperBTM (2*S*,3*R*)-**4** was recovered through filtration, followed by washing sequentially with $CHCl_3$, MeOH and THF (10 mL each) and finally drying under high vacuum for 2 h. Purification of the reaction mixture by

silica gel chromatography column using 10:2 CH_2Cl_2/hexane as eluent gave the product **2** (major) (yield 68%, 91:9 er) and **3** (minor) (yield 11%, 92:8 er).

Scale-up Procedure F: For the details of the Scale-up Procedure, using the same packed bed reactor as in general procedure D. Reaction solvent THF was pumped through the packed bed reactor at 0.5 ml·min^{-1} by HPLC pump for 30 min. Two 10.0 ml syringes were used to inject reagents using a syringe pump. 10 ml solutions of 2-phenacylbenzothiazole (0.120 M = 1.0 equiv.) and *i*-Pr_2NEt (0.132 M = 1.1 equiv.) in one syringe and 10 ml cinnamic anhydride (0.156 M = 1.3 equiv.) in another syringe were delivered to the column *via* a mixing T-piece using a syringe pump with a combined flowrate of 0.1 mL·min^{-1}. After complete addition of the 20 ml reagents, repeat above syringe pump procedure without elution of product and regeneration of catalyst (total flow time: 2500 min, total flow volume: 250 ml). After complete addition of all the reagents, the HPLC pump was connected, and pumped reaction solvent (THF) at 0.1 mL·min^{-1} for 45 min to ensure elution of the products. To analyse the change in yield during the scale-up process, four samples were taken at different time (554 min, 1314 min, 2272 min and 2500 min), and the results were shown in Table S1. For the recrystallization procedure, the product (*R*)-**25** was suspended in Et_2O then recrystallised from EtOAc, the racemic form crystallised preferentially, filtration and the liquors were concentrated to get product with improved enantiopurity.

Table S1. Conversion of product changed during scale-up process.

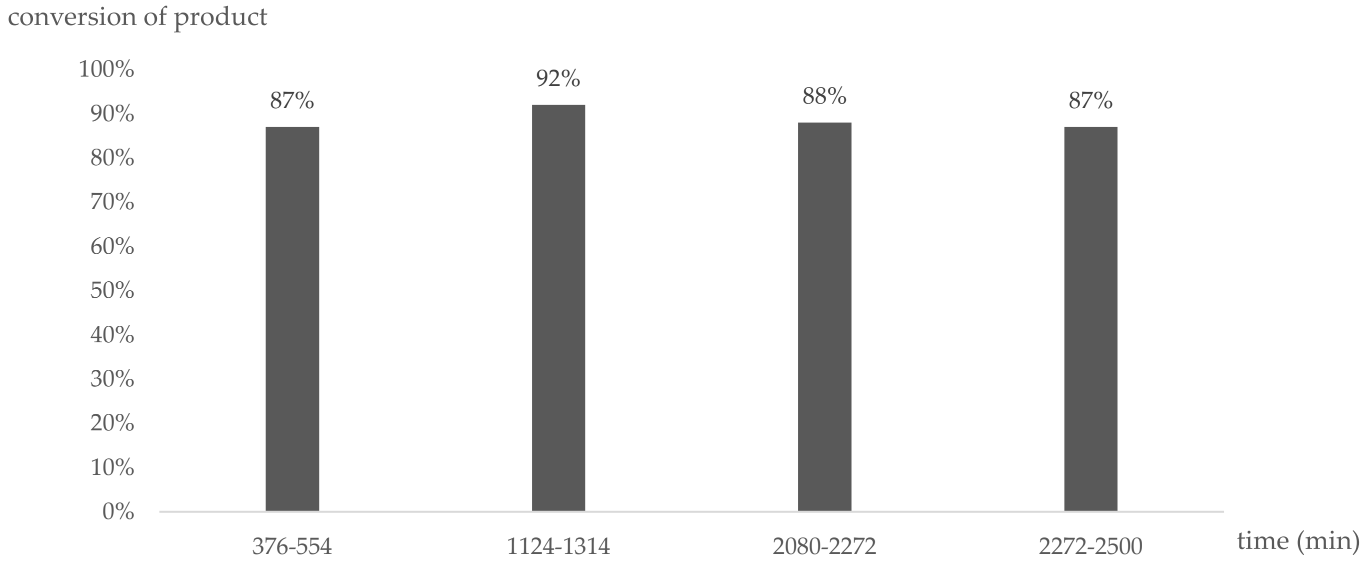

2.4.1 Lactams and lactones products

(3*R*)-4-benzoyl-3-phenyl-2,3-dihydro-1*H*-benzo[4,5]thiazolo[3,2-*a*]pyridin-1-one 2

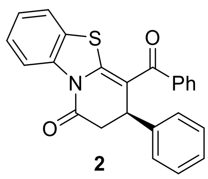

2

2 (major): Purification of the residue by silica gel chromatography column using 10:2 CH_2Cl_2/hexane as eluent gave the product **2** (major) (yield 76%, 94:6 er) and **3** (minor); (yield 76%, 94:6 er); $[\alpha]_D^{20}$ −139.7 (*c* 1.0 in $CHCl_3$); HPLC analysis, ChiralPak AD-H (20% ^{i}PrOH : hexane, flow rate 1 ml·min^{-1}, 254 nm, 30 °C), t_R (*S*)-**2**: 12.9 min, t_R (*R*)-**2**: 21.8 min, 94:6 er; ^{1}**H NMR** (400 MHz, $CDCl_3$) δ_H: 3.05 (1H, dd, *J* 15.9, 2.2, C(2)H_AH_B), 3.29 (1H, dd, *J* 15.9, 6.9, C(2)H_AH_B), 4.38 (1H, dd, *J* 6.9, 2.2, C(3)*H*-Ph), 7.11 (2H, d, *J* 7.2, C(3)H-PhC(2,6)*H*), 7.21-7.44 (10H, m, ArC*H*), 7.62 (1H, m, C(6)*H*), 8.47 (1H, d, *J* 7.8, C(9)*H*); ^{13}**C{^{1}H} NMR** (100 MHz, $CDCl_3$) δ_C: 38.6 (*C*(3)H), 41.4 (*C*(2)H_2), 107.7 (*C*(4)), 117.5 (*C*(9)H), 122.0 (*C*(6)H), 125.9 (*C*(7)H), 126.8 (C(3)H-Ar*C*(2,6)H), 127.0 (C(4)-Ar*C*(3,5)H, *C*(8)H), 127.6 (C(3)H-Ar*C*(4)H), 127.8 (*C*(5a)), 128.1 (C(4)-Ar*C*(2.6)H), 129.3 (C(3)H-Ar*C*(3,5)H), 130.3 (C(4)-Ar*C*(4)H), 136.0 (*C*(9a)), 139.4 (C(4)-Ar*C*(1)), 140.8 (C(3)H-Ar*C*(1)), 156.2 (*C*(4a)), 167.9 (*C*(1)=O), 191.2 (C(4)*C*=O). The data is in agreement with the literature.[11]

(4*R*)-5-(benzo[d]thiazol-2-yl)-4,6-diphenyl-3,4-dihydro-2*H*-pyran-2-one 3

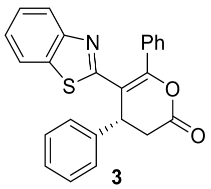

3

3 (minor): (yield 7%, 91:9 er); $[\alpha]_D^{20}$ −8.9 (*c* 1.0 in $CHCl_3$); HPLC analysis, ChiralPak AD-H (20% ^{i}PrOH : hexane, flow rate 1 ml·min^{-1}, 254 nm, 30 °C), t_R (*S*)-**3**: 7.7 min, t_R (*R*)-**3**: 10.7 min, 91:9 er; ^{1}**H NMR** (400 MHz, $CDCl_3$) δ_H: 3.08 (1H, dd, *J* 15.8, 1.6, C(3)H_AH_B), 3.32 (1H, dd, *J* 15.8, 7.6, C(3)H_AH_B), 5.03 (1H, dd, *J* 7.6, 1.6, C(4)*H*-Ph), 7.23-7.36 (6H, m, ArC*H*), 7.38-7.52 (3H, m, ArC*H*), 7.55-7.63 (4H, m, ArC*H*), 7.93 (1H, d, *J* 7.8, ArC*H*); ^{13}**C{^{1}H} NMR** (100 MHz, $CDCl_3$) δ_C: 36.9 (*C*(3)H_2), 41.3 (*C*(4)H), 115.1 (*C*(5)), 121.3 (C(5)-HetArCH), 123.1 (C(5)-HetArCH), 125.5 (C(5)-HetArCH), 126.1 (C(5)-HetArCH), 127.0 (C(4)H-Ph*C*(2,6)H), 127.8 (C(4)H-Ph*C*(4)H), 128.9 (C(6)-Ph*C*(3.5)H), 129.3 (C(4)Ph*C*(3,5)H), 130.1 (C(6)-Ph*C*(2,6)H), 130.8 (C(6)-Ph*C*(4)H), 131.9 (C(6)-PhC(1)), 135.7 (C(5)-HetArC), 139.6 (C(4)-Ph*C*(1)), 152.4 (C(5)-HetAr*C*), 154.4 (*C*(6)), 164.2 (C(5)-HetAr*C*=N), 166.6 (*C*(2)). Data in agreement with the literature.[11]

(3*R*)-4-benzoyl-3-(4-fluorophenyl)-2,3-dihydro-1*H*-benzo[4,5]thiazolo[3,2-*a*]pyridin-1-one 5

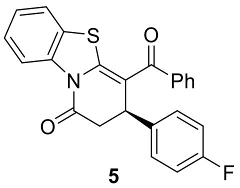

5

5 (major): Purification of the residue by silica gel chromatography column using 10:2 CH_2Cl_2/hexane as eluent gave the product **5** (major) (yield 83%, 94:6 er) and **6** (minor); mp 120–123 °C; $[\alpha]_D^{20}$ −112.5 (c 1.0 in $CHCl_3$); HPLC analysis, ChiralPak AD-H (20% ^{i}PrOH : hexane, flow rate 1 ml·min^{-1}, 254 nm, 30 °C), t_R (*S*)-**5**: 14.2 min, t_R (*R*)-**5**: 22.2 min, 94:6 er; ν_{max} (film)/cm^{-1} 1724 (C=O), 1627 (C=C), 1604 (C=O); **^{1}H NMR** (400 MHz, $CDCl_3$) δ_H: 3.00 (1H, dd, *J* 15.9, 2.4, C(2)H_AH_B), 3.27 (1H, dd, *J* 15.9, 6.8 C(2)H_AH_B), 4.33 (1H, dd, *J* 6.8, 2.4, C(3)*H*-Ar), 6.91-7.01 (2H, m, C(3)H-ArC(3,5)*H*), 7.01-7.11 (2H, m, C(3)H-ArC(2,6)*H*), 7.2-7.32 (4H, m, ArC*H*), 7.32- 7.45 (3H, m, ArC*H*), 7.58-7.65 (1H, m, C(6)*H*), 8.43-8.50 (1H, m, C(9)*H*); **^{13}C{^{1}H} NMR** (100 MHz, $CDCl_3$) δ_C: 38.0 (*C*(3)H), 41.5 (*C*(2)H_2), 107.5 (*C*(4)), 116.3 (d, $^2J_{CF}$ 21.4, C(3)H-Ar*C*(3,5)H), 117.6 (*C*(9)H), 122.0 (*C*(6)H), 126.0 (*C*(7)H), 126.9 (C(4)-Ar*C*(3,5)H), 127.1 (*C*(8)H), 127.7 (*C*(5a)), 128.2 (C(4)-Ar*C*(2,6)H), 128.5 (d, $^3J_{CF}$ 8.1, C(3)H-Ar*C*(2,6)), 130.4 (C(4)-Ar*C*(4)H), 136.0 (*C*(9a)), 136.5 (d, $^4J_{CF}$ 3.1, C(3)H-Ar*C*(1)), 139.3 (C(4)-Ar*C*(1)), 156.3 (*C*(4a)), 162.1 (d, $^1J_{CF}$ 246.5, C(3)H-Ar*C*(4)-F), 167.7 (*C*(1)=O), 191.3 (C(4)*C*=O); HRMS (APCI$^+$) $C_{24}H_{16}FNO_2S$ ([M+H]$^+$) requires 402.0959, found 402.0953 (−1.5 ppm).

(4*R*)-5-(benzo[d]thiazol-2-yl)-4-(4-fluorophenyl)-6-phenyl-3,4-dihydro-2H-pyran-2-one 6

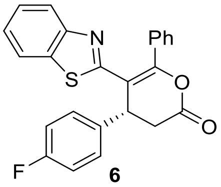

6

6 (minor): (yield 13%, 91:9 er); mp 140–144 °C; $[\alpha]_D^{20}$ −13.3 (c 1.0 in $CHCl_3$); chiral HPLC analysis, ChiralPak AD-H (20% *i*-PrOH : hexane, flow rate 1 mL·min^{-1}, 254 nm, 30 °C), t_R (*S*)-**6**: 7.8 min, t_R (*R*)-**6**: 9.3 min, 91:9 er; ν_{max} (film)/cm^{-1} 1770 (C=O), 1645 (C=N), 1597 (C=C), 1506 (C=O), 1342 (C–S) , 1273 (C–O); **^{1}H NMR** (400 MHz, $CDCl_3$) δ_H: 3.07 (1H, dd, *J* 15.8, 1.8, C(3)H_AH_B), 3.33 (1H, dd, *J* 15.8, 7.6, C(3)H_AH_B), 5.05 (1H, dd, *J* 7.5, 1.8, C(4)*H*-Ar), 6.97-7.06 (2H, m, C(4)H-ArC(3,5)*H*), 7.26-7.37 (4H, m, C(4)H-ArC(2,6)*H*, C(5)-HetArC(5)*H*), 7.40-7.50 (3H, m, C(5)-HetArC(6)*H*, C(6)-PhC(3,5)*H*), 7.50-7.60 (3H, m, C(6)-PhC(4)*H*, C(6)-PhC(2,6)*H*), 7.64 (1H, d, *J* 8.1, C(5)-HetArC(4)*H*), 7.95 (1H, d, *J* 8.2, C(5)-HetArC(7)*H*); **^{13}C{1H} NMR** (100 MHz, $CDCl_3$) δ_C: 36.9 (*C*(3)H_2), 40.4 (*C*(4)H), 115.2 (*C*(5)), 116.1 (d, $^2J_{CF}$ 21.6, C(4)H-Ar*C*(3,5)H), 121.2 (C(5)-HetAr*C*(7)H), 123.1 (C(5)-HetAr*C*(4)H), 125.5 (C(5)-HetAr*C*(6)H), 126.1 (C(5)-HetAr*C*(5)H), 128.6 (d, $^3J_{CF}$ 8.1, C(4)H-Ar*C*(2,6)H), 128.9 (C(6)-Ph*C*(2,6)H), 130.0 (C(6)-Ph*C*(3,5)H), 130.9 (C(6)-Ph*C*(4)H), 131.7 (C(6)-Ph*C*(1)), 135.4 (d, $^4J_{CF}$ 3.1, C(4)H-Ar*C*(1)), 135.7 (C(5)-HetAr*C*), 152.4 (C(5) -HetAr*C*), 154.4 (*C*(6)), 162.2 (d, $^1J_{CF}$ 246.3, C(4)H-Ar*C*(4)-F),

163.8 (C(5)-HetAr*C*=N), 166.4 (*C*(2)); HRMS (APCI$^+$) $C_{24}H_{16}FNO_2S$ ($[M+H]^+$) requires 402.0959, found 402.0955 (−1.0 ppm).

(11*R*)-4-benzoyl-3-(4-(trifluoromethyl)phenyl)-2,3-dihydro-1H-benzo[4,5]thiazolo [3,2-a]pyridin-1-one 7

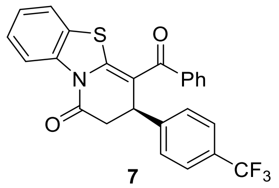

7

7 (major): Purification of the residue by silica gel chromatography column using 10:2 CH_2Cl_2/hexane as eluent gave the product **7** (major) (yield 74%, 94:6 er) and **8** (minor); $[\alpha]_D^{20}$ −109.4 (c 1.0 in $CHCl_3$); HPLC analysis, ChiralPak AD-H (20% *i*-PrOH : hexane, flow rate 1 mL·min^{-1}, 254 nm, 30 °C), t_R (*S*)-**7**: 10.8 min, t_R (*R*)-**7**: 17.4 min, 94:6 er; **^{1}H NMR** (400 MHz, $CDCl_3$) δ_H: 3.05 (1H, dd, *J* 16.0, 2.4, C(2)H_AH_B), 3.35 (1H, dd, *J* 16.0, 7.0, C(2)H_AH_B), 4.44 (1H, dd, *J* 7.1, 2.4, C(3)*H*-Ar), 7.17–7.25 (4H, m, Ar*H*), 7.26–7.33 (2H, m, Ar*H*), 7.33–7.44 (3H, m, Ar*H*), 7.52–7.58 (2H, m, Ar*H*), 7.59–7.65 (1H, m, C(6)*H*), 8.48 (1H, dd, *J* 8.0, 1.4, C(9)*H*); **^{13}C{1H} NMR** (100 MHz, $CDCl_3$) δ_C: 38.5 (*C*(3)H), 41.1 (*C*(2)H_2), 106.8 (*C*(4)), 117.6 (*C*(9)H), 122.1 (*C*(6)H), 124.0 (q, $^1J_{CF}$ 272.1, CF_3), 126.2 (*C*(7)H), 126.4 (q, $^3J_{CF}$ 3.9, C(3)H-Ar*C*(3,5)H), 126.8 (C(4)-ArC(2,6)H), 127.2 (*C*(8)H), 127.3 (C(4)-Ar*C*(3,5)H), 127.6 (*C*(5a)), 128.3 (*C*(3)H-ArC(2,6)H), 129.8 (q, $^2J_{CF}$ 32.7, *C*(3)H-ArC(4)-CF_3), 130.4 (*C*(4)-ArC(4)H), 135.9 (*C*(9a)), 139.3 (C(4)-Ar*C*(1)), 145.0 (C(3)H-Ar*C*(1)), 156.6 (*C*(4a)), 167.3 (*C*(1)=O), 191.1 (C(4)*C*=O). Data in agreement with the literature.[11]

(4*R*)-5-(benzo[d]thiazol-2-yl)-6-phenyl-4-(4-(trifluoromethyl)phenyl)-3,4-dihydro-2*H*-pyran-2-one 8

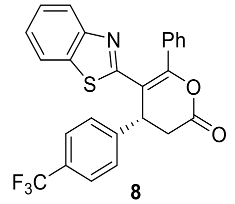

8

8 (minor): (yield 21%, 93:7 er); $[\alpha]_D^{20}$ +8.4 (c 1.0 in $CHCl_3$); HPLC analysis, ChiralPak AD-H (20% *i*-PrOH : hexane, flow rate 1 mL·min^{-1}, 254 nm, 30 °C), t_R (*S*)-**8**: 6.2 min, t_R (*R*)-**8**: 7.5 min, 93:7 er; **^{1}H NMR** (400 MHz, $CDCl_3$) δ_H: 3.08 (1H, dd, *J* 15.8, 1.7, C(3) H_AH_B), 3.35 (1H, dd, *J* 15.9, 7.7, C(3)H_AH_B), 5.14 (1H, dd, *J* 7.7, 1.7, C(4)*H*-Ar), 7.27–7.33 (1H, m, C(5)-HetArC(6)*H*), 7.36–7.43 (1H, m, C(5)-HetArC(5)*H*), 7.43–7.50 (4H, m, ArC*H*), 7.49–7.65 (6H, m, ArC*H*), 7.91 (1H, d, *J* 8.3, C(5)-HetArC(7)*H*); **^{13}C{1H} NMR** (100 MHz, $CDCl_3$) δ_C: 36.4 (*C*(3)H_2), 40.7 (*C*(4)H), 114.7 (*C*(5)), 121.2 (C(5)-HetArC(4)H), 123.1 (C(5)-HetAr*C*(7)H), 125.6 (C(5)-HetArC(6)H), 126.1 (C(5)-HetArC(5)H), 126.2 (q, $^3J_{CF}$ 4.0, C(4)H-Ar*C*(3,5)H), 127.5 (C(6)-Ph*C*(2,6)H), 129.0 (C(4)H-Ar*C*(2,6)H), 130.0 (C(6)-Ph*C*(3,5)H), 131.1 (C(6)-Ph*C*(4)H), 131.6 (C(6)-Ph*C*(1)), 135.6 (C(5)-HetAr*C*),

143.8 (C(4)H-ArC(1)), 152.3 (C(5)-HetArC), 154.9 (C(6)), 163.5 (C(5)-HetArC=N), 166.0 (C(2)), [C(4)H-ArC(4)-CF_3 and CF_3 not seen in 13C NMR due to low sample quantity]. Data in agreement with the literature.[11]

(11*S*)-4-benzoyl-3-(2-chlorophenyl)-2,3-dihydro-1H-benzo[4,5]thiazolo[3,2-a]pyridin-1-one 9

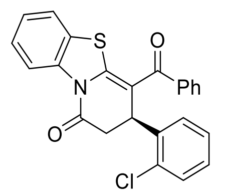

9

9 (major): Purification of the residue by silica gel chromatography column using 10:2 CH_2Cl_2/hexane as eluent gave the product **9** (major) (yield 58%, 79:21 er) and **10** (minor); mp 154–160 °C; $[\alpha]_D^{20}$ −108.4 (c 1.0, $CHCl_3$); chiral HPLC analysis, ChiralPak AD-H (15% *i*-PrOH : hexane, flow rate 1 mL·min^{-1}, 254 nm, 30 °C), t_R (*S*)-**9**: 12.9 min, t_R (*R*)-**9**: 18.0 min, 79:21 er; ν_{max} (film)/cm^{-1} 3069 (C–H), 2907 (C–H), 1716 (C=O), 1595 (C=C), 1460 (C–N), 1354 (C–S); **^{1}H NMR** (400 MHz, $CDCl_3$) δ_H: 3.11 (1H, dd, *J* 16.1, 2.3, C(2)H_AH_B), 3.24 (1H, dd, *J* 16.1, 7.2, C(2)H_AH_B), 4.33 (1H, dd, *J* 7.2, 2.3, C(3)*H*-Ar), 7.11-7.16 (1H, m, ArC*H*), 7.17-7.33 (6H, m, ArC*H*), 7.34-7.48 (4H, m, ArC*H*), 7.62-7.68 (1H, m, ArC*H*), 8.46-8.52 (1H, m, ArC*H*); **^{13}C{1H} NMR** (100 MHz, $CDCl_3$) δ_C: 35.9 (*C*(3)H), 39.0 (*C*(2)H_2), 107.0 (*C*4), 117.6 (*C*(6)H), 122.1 (*C*(7)H), 126.0 (*C*(9)H), 126.6 (C(4)-Ar*C*(2,6)H), 127.2 (C(3)H-Ar*C*(5)H), 127.8 (*C*(8)H), 128.0 (C(3)H-Ar*C*(6)H), 128.2 (C(4)-Ar*C*(3,5)H), 129.1 (C(3)H-Ar*C*(4)H), 130.4 (C(4)-Ar*C*(4)H), 130.6 (C(3)H-Ar*C*(3)H), 133.1 (*C*(5a)), 136.0 (C(3)H-Ar*C*(2)), 137.5 (*C*(9a)), 137.5 (C(3)H-Ar*C*(1)), 139.1 (C(4)-Ar*C*(1)), 157.0 (*C*(4a)), 167.7 (*C*(1)=O), 191.0 (C(4)*C*=O); HRMS (APCI$^+$) $C_{24}H_{16}ClNO_2S$ ([M+H]$^+$) requires 418.0663, found 418.0653 (−2.4 ppm).

(4*S*)-5-(benzo[d]thiazol-2-yl)-4-(2-chlorophenyl)-6-phenyl-3,4-dihydro-2H-pyran-2-one 10

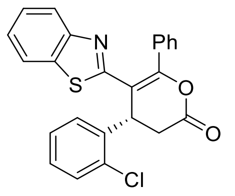

10

10 (major): (yield 6%, 62:38 er); mp 137–142 °C; $[\alpha]_D^{20}$ −13.7 (c 1.0, $CHCl_3$); chiral HPLC analysis, ChiralPak AD-H (15% *i*-PrOH : hexane, flow rate 1 mL·min^{-1}, 254 nm, 30 °C), t_R (*S*)-**10**: 7.9 min, t_R (*R*)-**10**: 8.6 min, 62:38 er; ν_{max} (film)/cm^{-1} 2957 (C–H), 2922 (C–H), 2850 (C–H), 1775 (C=O), 1651 (C=N), 1595 (C=C), 1346 (C–S), 1273 (C–O); **^{1}H NMR** (400 MHz, $CDCl_3$) δ_H: 3.18 (1H, dd, *J* 16.1, 1.8, C(3)H_AH_B), 3.30 (1H, dd, *J* 15.9, 7.8, C(3)H_AH_B), 5.47 (1H, dd, *J* 7.8, 1.7, C(4)*H*-Ar), 7.93 (1H, d, *J* 8.4, C(5)-HetAr(7)*H*); **^{13}C{1H} NMR** (100 MHz, $CDCl_3$) δ_C: 35.3 (*C*(3)H_2), 41.1 (*C*(4)H), 114.0 (*C*(5)), 121.2 (ArCH), 123.3 (ArCH), 125.5 (ArCH), 126.1 (ArCH), 127.4 (ArCH),

128.9 (C(6)-PhC(3,5)H), 129.5 (ArCH), 129.9 (C(6)-PhC(2,6)H), 130.9 (C(6)-PhC(4)H), 131.7 (C(6)-PhC(1)), 133.9 (ArCH), 135.6 (C(5)-HetArC), 137.7 (C4)H-ArC(2)), 152.3 (C(5)-HetArC), 155.3 (C(6)), 163.6 (C(5)-HetArC=N), 166.1 (C(2)); HRMS (APCI$^+$) $C_{24}H_{16}ClNO_2S$ ([M+H]$^+$) requires 418.0663, found 418.0654 (−2.1 ppm).

(3*R*)-4-benzoyl-3-(4-methoxyphenyl)-2,3-dihydro-1*H*-benzo[4,5]thiazolo[3,2-*a*]pyridin-1-one 11

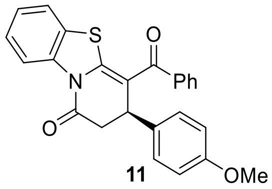

11

11 (major): Purification of the residue by silica gel chromatography column using 10:2 CH_2Cl_2/hexane as eluent gave the product **11** (major) (yield 81%, 90:10 er) and **12** (minor); $[\alpha]_D^{20}$ −119.7 (c 1.0 in $CHCl_3$); chiral HPLC analysis, ChiralPak AD-H (20% *i*-PrOH : hexane, flow rate 1 mL·min^{-1}, 254 nm, 30 °C), t_R (*S*)-**11**: 17.9 min, t_R (*R*)-**11**: 29.9 min, 90:10 er; **^{1}H NMR** (400 MHz, $CDCl_3$) δ_H: 3.03 (1H, dd, *J* 15.8, 2.3, C(12)H_AH_B), 3.26 (1H, dd, *J* 15.8, 6.7 C(12)H_AH_B), 3.79 (3H, s, OC*H*3), 4.31 (1H, dd, *J* 6.7, 2.3, C(3)*H*-Ar), 6.81-6.86 (2H, m, C(3)H-ArC(3)*H*), 7.00-7.06 (2H, m, C(3)H-ArC(2)*H*), 7.28-7.34 (4H, m, Ar*H*), 7.32-7.44 (3H, m, Ar*H*), 7.56-7.65 (1H, m, C(6)*H*), 8.43-8.51 (1H, m, C(9)*H*); **^{13}C{1H} NMR** (100 MHz, $CDCl_3$) δ_C: 37.8 (*C*(3)H), 41.7 (*C*(2)H_2), 55.3 (OCH_3), 108.1 (*C*(4)), 114.7 (C(3)H-Ar*C*(3,5)H), 117.5 (*C*(9)H), 122.0 (*C*(6)H), 125.9 (*C*(7)H), 127.0 (*C*(8)H), 127.1 (C(4)-Ar*C*(2,6)H), 127.8 (*C*(5a)), 127.9 (C(3)H-Ar*C*(2,6)H), 128.1 (C(4)-Ar*C*(3,5)H), 130.3 (C(4)-Ar*C*(4)H), 132.6 (C(3)H-Ar*C*(1)), 136.1 (*C*(9a)), 139.4 (C(4)-Ar*C*(1)), 156.0 (*C*(4a)), 158.9 (C(3)H-Ar*C*(4)), 168.1 (*C*(1)=O), 191.3 (C(4)*C*=O). Data in agreement with the literature.[11]

(4*R*)-5-(benzo[d]thiazol-2-yl)-4-(4-methoxyphenyl)-6-phenyl-3,4-dihydro-2H-pyran-2-one 12

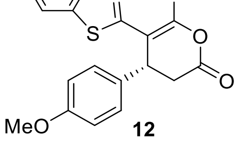

12

12 (minor): (yield 16%, 88:12 er); $[\alpha]_D^{20}$ +15.3 (c 1.0 in $CHCl_3$); chiral HPLC analysis, ChiralPak AD-H (20% *i*-PrOH : hexane, flow rate 1 mL·min^{-1}, 254 nm, 30 °C), t_R (*S*)-**12**: 9.6 min, t_R (*R*)-**12**: 14.1 min, 88:12 er; **^{1}H NMR** (400 MHz, $CDCl_3$) δ_H: 3.07 (1H, dd, *J* 15.7, 1.8, C(3)H_AH_B), 3.31 (1H, dd, *J* 15.7, 7.5, C(3)H_AH_B), 3.77 (3H, s, OC*H*3), 4.97 (1H, dd, *J* 7.5, 1.8, C(4)*H*-Ar), 6.84 (2H, d, *J* 8.7, C(4)H-ArC(3)*H*), 7.28 (3H, dd, *J* 16.1, 8.3, C(4)H-ArC(2)*H*, C(5)-HetArC(5)*H*), 7.38-7.46 (3H, m, C(5)-HetArC(6)*H*, C(6)-PhC(3)*H*), 7.50 (1H, m, C(6)-PhC(4)*H*), 7.55-7.59 (2H, m, C(6)-PhC(2)*H*), 7.62 (1H, d, *J* 8.0, C(5)-HetArC(4)*H*), 7.95 (1H, d, *J* 8.2, C(5)-HetArC(7)*H*); **^{13}C{1H} NMR** (100 MHz, $CDCl_3$) δ_C: (100 MHz, $CDCl_3$) 37.1 (*C*(3)H_2), 40.5 (*C*(4)H), 55.2 (OCH_3), 114.6

(C(4)HArC(3,5)H), 115.4 (*C*(5)), 121.2 (C(5)-HetAr*C*(7)H), 123.1 (C(5)-HetAr*C*(4)H), 125.3 (C(5)-HetAr*C*(6)H), 126.0 (C(5)-HetAr*C*(5)H), 128.1 (C(4)H-Ar*C*(2,6)H), 128.8 (C(6)-Ph*C*(2,6)H), 130.0 (C(6)-Ph*C*(3,5)H), 130.7 (C(6)-Ph*C*(4)H), 131.5 (C(4)H-Ar*C*(1)), 131.9 (C(6)-Ph*C*(1)), 148.2 (C(5)-HetAr*C*), 152.5 (C(5)-HetAr*C*), 154.0 (*C*(6)), 159.0 (C(4)H-Ar*C*(4)), 164.2 (C(5)-HetAr*C*=N)), 166.7 (*C*(2)). Data in agreement with the literature.[11]

(3*R*)-4-benzoyl-3-(*p*-tolyl)-2,3-dihydro-1*H*-benzo[4,5]thiazolo[3,2-*a*]pyridin-1-one 13

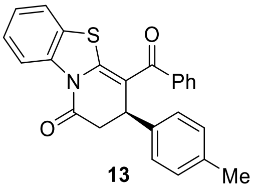

13

13 (major): Purification of the residue by silica gel chromatography column using 10:2 CH_2Cl_2/hexane as eluent gave the product **13** (major) (yield 84%, 93:7 er) and **14** (minor); mp 128–132 °C; $[\alpha]_D^{20}$ −129.2 (c 1.0, $CHCl_3$); chiral HPLC analysis, ChiralPak AD-H (20% *i*-PrOH : hexane, flow rate 1 mL·min^{-1}, 254 nm, 30 °C), t_R (*S*)-**13**: 11.7 min, t_R (*R*)-**13**: 23.1 min, 93:7 er; ν_{max} (film)/cm^{-1} 1719 (C=O), 1626 (C=C), 1601 (C=C), 1489 (C–N); **^{1}H NMR** (400 MHz, $CDCl_3$) δ_H: 2.33 (3H, s, ArC*H*$_3$), 3.04 (1H, dd, *J* 15.9, 2.3, C(2)H_AH_B), 3.27 (1H, dd, *J* 15.9, 6.8, C(2)H_AH_B), 4.33 (1H, dd, *J* 6.8, 2.3, C(3)*H*-Ar), 7.00 (2H, m, Ar*H*), 7.08 (2H, d, *J* 7.9, Ar*H*), 7.27-7.44 (7H, m, Ar*H*), 7.62-7.64 (1H, m, Ar*H*), 8.47-8.49 (1H, m, Ar*H*); **^{13}C{1H} NMR** (100 MHz, $CDCl_3$) δ_C: 21.1 (C(3)H-ArCH$_3$), 38.2 (*C*(3)H), 41.6 (*C*(2)H$_2$), 107.9 (*C*4), 117.6 (*C*(6)H), 122.0 (*C*(7)H), 125.9 (*C*(8)H), 126.7 (C(3)H-Ar*C*(2,6)H), 127.0 (*C*(9)H), 127.1 (C(4)-Ar*C*(2,6)H), 127.8 (*C*(5a)), 128.1 (C(4)-Ar*C*(3,5)H), 130.0 (C(3)H-Ar*C*(3,5)H), 130.3 (C(4)-Ar*C*(4)H), 136.1 (C(3)H-Ar*C*(4)), 137.2 (C(4)-Ar*C*(1)), 137.6 (C(3)H-Ar*C*(1)), 139.4 (*C*(9a)), 156.1 (*C*(4a)), 168.0 (*C*(1)=O), 191.3 (C(4)*C*=O); HRMS (APCI$^+$) $C_{25}H_{19}NO_2S$ ([M+H]$^+$) requires 398.1209, found 398.1204 (−1.2 ppm).

(4*R*)-5-(benzo[d]thiazol-2-yl)-6-phenyl-4-(*p*-tolyl)-3,4-dihydro-2*H*-pyran-2-one 14

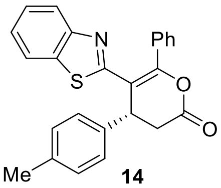

14

14 (minor): (yield 8%, 89:11 er); mp 140–145 °C; $[\alpha]_D^{20}$ +26.0 (c 1.0, $CHCl_3$); HPLC analysis, ChiralPak AD-H (20% *i*-PrOH : hexane, flow rate 1 mL·min^{-1}, 254 nm, 30 °C), t_R (*S*)-**14**: 7.7 min, t_R (*R*)-**14**: 10.5 min, 89:11 er; ν_{max} (film)/cm^{-1} 2920 (C–H), 2852 (C–H), 1782 (C=O), 1645 (C=N), 1597 (C=C), 1348 (C–S), 1273 (C–O); **^{1}H NMR** (400 MHz, $CDCl_3$) δ_H: 2.30 (3H, s, ArC*H*$_3$), 3.07 (1H, dd, *J* 15.7, 1.7, C(3)H_AH_B), 3.32 (1H, dd, *J* 15.7, 7.5, C(3)H_AH_B), 4.99 (1H, dd, *J* 7.5, 1.7, C(4)*H*-Ar), 7.05 (1H, d, *J* 7.8, Ar*H*), 7.13-7.14 (2H, m, Ar*H*), 7.19-7.23 (1H,

m, Ar*H*), 7.28-7.32 (2H, m, ArC*H*), 7.40-7.47 (3H, m, Ar*H*), 7.50-7.52 (1H, m, Ar*H*), 7.54-7.60 (2H, m, Ar*H*), 7.63 (1H, d, *J* 8.0, C(5)-HetArC*H*), 7.95 (1H, d, *J* 8.0, C(5)-HetArC*H*); **^{13}C{^{1}H} NMR** (100 MHz, $CDCl_3$) δ_C: 21.0 (C(4)H-ArCH_3), 37.0 (*C*(3)H_2), 40.9 (*C*(4)H), 115.2 (*C*(6)), 121.2 (C(5)-HetArCH), 123.1 (C(5)-HetArCH), 125.3 (C(5)-HetArCH), 126.0 (C(5)-HetArCH), 126.8 (C(4)H-Ar*C*(2,6)H), 128.8 (C(6)-Ph*C*(2,6)H), 129.9 (C(6)-Ph*C*(3,5)H), 130.0 (C(4)HAr*C*(3,5)H), 130.7 (C(6)-Ph*C*(4)H), 131.9 (C(6)-Ph*C*(1)), 135.7 (C(5)-HetAr*C*H), 136.5 (C(4)H-Ar*C*(4)), 137.4 (C(4)H-Ar*C*(1)), 152.5 (*C*(5)), 154.1 (C(5)-HetAr*C*), 164.2 (C(5)-HetAr*C*=N), 166.7 (*C*(2)); HRMS (APCI$^+$) $C_{25}H_{19}NO_2S$ ([M+H]$^+$) requires 398.1209, found 398.1206 (−0.7 ppm).

(3*R*)-4-benzoyl-3-(*m*-tolyl)-2,3-dihydro-1*H*-benzo[4,5]thiazolo[3,2-*a*]pyridin-1-one 15

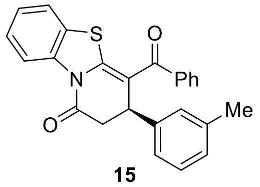

15

15 (major): Purification of the residue by silica gel chromatography column using 10:2 CH_2Cl_2/hexane as eluent gave the product **15** (major) (yield 81%, 96:4 er) and **16** (minor); $[\alpha]_D^{20}$ −113.2 (c 1.0, $CHCl_3$); chiral HPLC analysis, ChiralPak AD-H (20% *i*-PrOH : hexane, flow rate 1 mL·min^{-1}, 254 nm, 30 °C), t_R (*S*)-**15**: 10.7 min, t_R (*R*)-**15**: 15.8 min, 96:4 er; **^{1}H NMR** (400 MHz, $CDCl_3$) δ_H: 2.30 (3H, s, ArC*H*$_3$), 3.07 (1H, dd, *J* 15.9, 2.4, C(2)*H*$_A$H$_B$), 3.28 (1H, dd, *J* 15.9, 6.9, C(2)H$_A$*H*$_B$), 4.33 (1H, dd, *J* 6.9, 2.4, C(3)*H*-Ar), 6.90 (2H, m, Ar*H*), 7.08 (1H, d, *J* 7.5, Ar*H*), 7.19 (1H, t, *J* 7.5, Ar*H*), 7.28-7.42 (7H, m, Ar*H*), 7.62-7.64 (1H, m, Ar*H*), 8.48-8.50 (1H, m, Ar*H*); **^{13}C{1H} NMR** (100 MHz, $CDCl_3$) δ_C: 21.6 (C(3)H-ArCH_3), 38.5 (*C*(3)H), 41.4 (*C*(2)H_2), 107.7 (*C*(4)), 117.6 (*C*(6)H), 122.0 (*C*(7)H), 123.8 (C(3)H-Ar*C*(6)H), 125.9 (*C*(8)H), 127.0 (C(3)H-Ar*C*(4)H), 127.1 (C(4)-Ar*C*(2,6)H), 127.5 (*C*(9)H), 127.8 (*C*(5a)), 128.1 (C(4)-Ar*C*(3,5)H), 128.4 (C(3)H-Ar*C*(5)H), 129.2 (C(3)H-Ar*C*(2)H), 130.3 (C(4)-Ar*C*(4)H), 136.1 (C(3)H-Ar*C*(3)), 139.0 (C(4)-Ar*C*(1)), 139.4 (C(9a)Ar*C*), 140.8 (C(3)H-Ar*C*(1)), 156.2 (*C*(4a)), 168.0 (*C*(1)=O), 191.3 (C(4)*C*=O). Data in agreement with the literature.[11]

(4*R*)-5-(benzo[d]thiazol-2-yl)-6-phenyl-4-(*m*-tolyl)-3,4-dihydro-2*H*-pyran-2-one 16

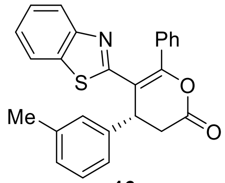

16

16 (minor): (yield 10%, 92:8 er); $[\alpha]_D^{20}$ −11.3 (c 1.0, $CHCl_3$); HPLC analysis, ChiralPak AD-H (20% *i*-PrOH : hexane, flow rate 1 mL·min^{-1}, 254 nm, 30 °C), t_R (*S*)-**16**: 6.8 min, t_R (*R*)-**16**: 9.4 min, 92:8 er; **^{1}H NMR** (400 MHz, $CDCl_3$) δ_H: 2.33 (3H, s, C(4)HArC*H*$_3$), 3.08 (1H, dd, *J* 15.7, 0.7, C(3)*H*$_A$H$_B$), 3.32 (1H, dd, *J* 15.7, 7.6, C(3)H$_A$*H*$_B$),

4.99 (1H, dd, *J* 7.7, 1.7, C(4)*H*-Ar), 7.05 (1H, d, *J* 7.8, C(4)H-ArC(4)*H*), 7.13-7.14 (2H, m, Ar*H*), 7.19-7.23 (1H, m, C(4)H-ArC(2)*H*), 7.28-7.32 (2H, m, C(5)-HetArC*H*), 7.40-7.47 (3H, m, Ar*H*), 7.50-7.52 (1H, m, C(4)H-ArC(5)*H*), 7.54-7.60 (2H, m, Ar*H*), 7.63 (1H, d, *J* 8.0, C(5)-HetArC*H*), 7.95 (1H, d, *J* 8.2, C(5)-HetArC*H*). **^{13}C{1H} NMR** (100 MHz, $CDCl_3$) δ_C: 21.6 (C(4)H-ArCH$_3$), 37.0 (*C*(3)H$_2$), 41.3 (*C*(4)H), 115.0 (*C*(6)), 121.3 (C(5)-HetArCH), 123.1 (C(5)-HetArCH), 123.9 (C(5)-HetArCH), 125.3 (C(4)H-Ar*C*(6)H), 126.0 (C(5)-HetAr*C*H), 127.8 (C(4)H-ArC(4)H), 128.6 (C(6)-PhC(4)H), 128.8 (C(6)-PhC(2,6)H), 129.1 (C(4)H-ArC(5)H), 123.0 (C(6)-PhC(3,5)H), 130.8 (C(4)H-Ar*C*(2)H, 132.1 (C(6)-Ph*C*(1)), 135.8 (C(5)-HetAr*C*), 138.9 (C(4)H-Ar*C*(3)), 139.5 (C(4)H-Ar*C*(1)), 152.5 (*C*(5)), 154.2 (C(5)-HetArC), 164.2 (C(5)-HetAr*C*=N), 166.6 (*C*(2)). Data in agreement with the literature.[11]

(3*R*)-4-benzoyl-3-(naphth-1-yl)-2,3-dihydro-1*H*-benzo[4,5]thiazolo[3,2-*a*]pyridin-1-one 17

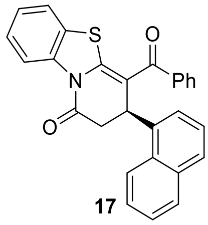

17

17 (major): Purification of the residue by silica gel chromatography column using 10:2 CH_2Cl_2/hexane as eluent gave the product **17** (major) (yield 75%, 96:4 er) and **18** (minor); mp 178–183 °C; $[\alpha]_D^{20}$ −99.5 (c 1.0 in $CHCl_3$); chiral HPLC analysis, ChiralPak AD-H (20% *i*-PrOH : hexane, flow rate 1 mL·min^{-1}, 254 nm, 30 °C), t_R (*S*)-**17**: 6.8 min 16.2 min, t_R (*R*)-**17**: 25.6 min, 96:4 er; ν_{max} (film)/cm^{-1} 3051 (C–H), 1717 (C=O), 1610(C=O), 1570 (C=C), 1493 (C–N); **^{1}H NMR** (400 MHz, $CDCl_3$) δ_H: 3.19 (1H, dd, *J* 15.8, 2.0, C(2)H_AH_B), 3.38 (1H, dd, *J* 15.8, 7.2, C(2)H_AH_B), 5.18 (1H, dd, *J* 7.1, 2.0, C(3)*H*-Ar), 7.06-7.12 (2H, m, Ar*H*), 7.19-7.27 (2H, m, Ar*H*), 7.28-7.43 (5H, m, Ar*H*), 7.50-7.61 (2H, m, Ar*H*), 7.63-7.71 (1H, m, Ar*H*), 7.79-7.99 (3H, m, Ar*H*), 8.42-8.48 (1H, m, Ar*H*); **^{13}C{1H} NMR** (100 MHz, $CDCl_3$) δ_C: 34.9 (*C*(3)H), 40.1 (*C*(2)H$_2$), 107.7 (*C*(4)), 117.5 (*C*(9)H), 122.0 (*C*(6)H), 122.1 (C(3)H-ArCH), 124.3 (C(3)H-ArCH), 125.7 (C(3)H-ArCH), 125.9 (*C*(7)H), 126.7 (C(3)H-ArCH), 126.9 (C(4)-ArC(3,5)H), 127.1 (*C*(8)H), 127.9 (*C*(5a)), 128.1 (C(4)-Ar*C*(2,6)H)), 128.7 (C(3)H-ArCH), 129.5 (C(3)H-ArCH), 130.0 (C(3)H-Ar*C*), 130.3 (C(4)-Ar*C*(4)H), 134.7 (C(3)H-Ar*C*), 135.2 (C(3)H-Ar*C*), 136.1 (C(9a)), 139.0 (C(4)-ArC(1)), 157.0 (*C*(4a)), 167.8 (*C*(1)=O), 190.8 (C(4)*C*=O); HRMS (APCI$^+$) $C_{28}H_{19}NO_2S$ ([M+H]$^+$) requires 434.1209, found 434.1199 (−2.3 ppm).

(4*R*)-5-(benzo[d]thiazol-2-yl)-4-(naphth-1-yl)-6-phenyl-3,4-dihydro-2*H*-pyran-2-one 18

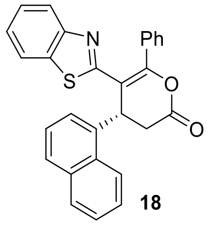

18

18 (minor): (yield 16%, 80:20 er); mp 158–162 °C; $[\alpha]_D^{20}$ +8.9 (c 1.0 in $CHCl_3$); chiral HPLC analysis, ChiralPak AD-H (20% i-PrOH : hexane, flow rate 1 mL min^{-1}, 254 nm, 30 °C), t_R (*R*)-**18**: 8.5 min, t_R (*S*)-**18**: 11.1 min, 80:20 er; ν_{max} (film)/cm^{-1} 3117 (C–H), 3063 (C–H), 2924 (C–H), 1778 (C=O), 1651 (C=N), 1597 (C=C), 1277 (C–O); **^{1}H NMR** (400 MHz, $CDCl_3$) δ_H: 3.25 (1H, dd, *J* 15.5, 1.5, C(3)H_AH_B), 3.46 (1H, dd, *J* 15.8, 7.7, C(3)H_AH_B), 5.88 (1H, dd, *J* 7.7, 1.5, C(4)*H*-Ar), 7.22-7.30 (2H, m, Ar*H*), 7.32-7.71 (10H, m, Ar*H*), 7.74-7.92 (3H, m, Ar*H*), 8.21 (1H, d, *J* 8.5, Ar*H*); **^{13}C{1H} NMR** (100 MHz, $CDCl_3$) δ_C: 36.2 (C(3)H_2), 37.5 (C(4)H), 114.5 (C(5)), 121.2 (C(5)-HetArCH), 122.7 (C(4)H-ArCH), 123.1 (C(5)-HetArCH), 123.6 (C(4)H-ArCH), 125.6 (C(5)-HetArCH), 125.9 (C(4)H-ArCH), 126.0 (C(5)-HetArCH),126.7 (C(4)H-ArCH), 128.7 (C(4)H-ArCH), 128.9 (C(6)-PhC(3,5)H), 129.3 (C(4)H-ArCH), 130.1 (C(6)-PhC(2,6)H), 130.7 (C(4)H-ArC), 130.8 (C(6)-PhC(4)H), 131.9 (C(6)-PhC(1)), 133.8 (C(4)H-ArC), 134.5 (C(4)H-ArC), 135.7 (C(5)-HetArC), 152.4 (C(5)-HetArC), 155.1 (C(6)), 164.1 (C(5)-HetArC=N), 166.3 (C(2)); HRMS (APCI$^+$) $C_{28}H_{19}NO_2S$ ($[M+H]^+$) requires 434.1209, found 434.1200 (−2.0 ppm).

(3*S*)-4-benzoyl-3-(furan-2-yl)-2,3-dihydro-1*H*-benzo[4,5]thiazolo[3,2-*a*]pyridin-1-one 19

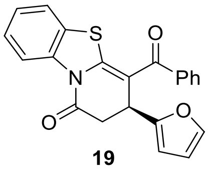

19

19 (major): Purification of the residue by silica gel chromatography column using 10:2 CH_2Cl_2/hexane as eluent gave the product **19** (major) (yield 91%, 93:7 er) and **20** (minor); $[\alpha]_D^{20}$ −42.1 (c 1.0, $CHCl_3$); HPLC analysis, ChiralPak AD-H (20% *i*-PrOH : hexane, flow rate 1 mL·min^{-1}, 254 nm, 30 °C), t_R (*R*)-**19**: 14.9 min, t_R (*S*)-**19**: 19.7 min, 93:7 er; **^{1}H NMR** (400 MHz, $CDCl_3$) δ_H: 3.17 (1H, dd, *J* 16.1, 6.1, C(2)H_AH_B), 3.25 (1H, dd, *J* 16.1 2.5, C(2)H_AH_B), 4.45 (1H, ddd, *J* 6.1, 2.6, 1.0, C(3)*H*-Ar), 5.99 (1H, m, Ar*H*), 6.25 (1H, dd, *J* 3.3, 1.9, Ar*H*), 7.29-7.51 (8H, m, Ar*H*), 7.54-7.61 (1H, m, Ar*H*), 8.46-8.53 (1H, m, Ar*H*); **^{13}C{1H} NMR** (100 MHz, $CDCl_3$) δ_C: 33.0 (*C*(3)H), 38.0 (*C*(2)H_2), 105.7 (*C*(4)), 106.9 (furanyl*C*(3)H), 110.4 (furanyl*C*(4)H), 117.7 (*C*(9)H), 121.9 (*C*(6)H), 125.9 (*C*(7)H), 127.0 (C(4)-Ar*C*(2,6)H), 127.1 (*C*(8)H), 127.6 (*C*(5a)), 128.3 (C(4)-Ar*C*(3,5)H), 130.3 (C(4)-ArC(4)H), 136.1 (C(4)-Ar*C*(1)), 139.4 (*C*(9a)), 142.8 (furanyl*C*(5)H), 153.6 (furanyl*C*(2)), 156.7 (*C*(4a)), 167.8 (*C*(1)=O), 190.9 (C(4)*C*=O). Data in agreement with the literature.[11]

(4*S*)-5-(benzo[d]thiazol-2-yl)-4-(furan-2-yl)-6-phenyl-3,4-dihydro-2*H*-pyran-2-one 20

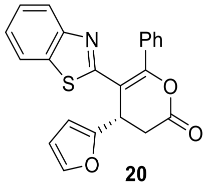

20

20 (minor): (yield 13%, 79:21 er); $[\alpha]_D^{20}$ −2.7 (c 1.0 in $CHCl_3$); chiral HPLC analysis, ChiralPak AD-H (20% *i*-PrOH : hexane, flow rate 1 mL·min^{-1}, 254 nm, 30 °C), t_R (*R*)-**20**: 7.6 min, t_R (*S*)-**20**: 14.8 min, 79:21 er; **^{1}H NMR** (400 MHz, $CDCl_3$) δ_H: 3.21 (1H, dd, *J* 15.9, 6.7, C(3)H_AH_B), 3.28 (1H, dd, *J* 15.9, 1.9, C(3)H_AH_B), 5.14 (1H, dd, *J* 6.8, 1.6, C(4)*H*-Ar), 6.19 (1H, d, *J* 3.3, ArH), 6.26 (1H, dd, *J* 3.3, 1.9, ArH), 7.31 (1H, t, *J* 7.6, ArH), 7.34 (1H, d, *J* 1.8, ArH), 7.44 (3H, td, *J* 7.3, 1.5, ArH), 7.49-7.57 (3H, m, ArH), 7.61-7.68 (1H, m, ArH), 7.99 (1H, d, *J* 8.2, ArH); **^{3}C{1H} NMR** (100 MHz, $CDCl_3$) δ_C: 36.0 (*C*(3)H_2), 36.8 (*C*(4)H), 106.8 (furanylC(3)H), 110.3 (furanylC(4)H), 113.3 (*C*(5)), 121.2 (C(5)-HetAr*C*(7)H), 123.1 (C(5)-HetAr*C*(4)H), 125.4 (C(5)-HetAr*C*(6)H), 126.1 (C(5)-HetAr*C*(5)H), 128.9 (C(6)-Ph*C*(3,5)H), 130.0 (C(6)-Ph*C*(2,6)H), 130.9 (C(6)-Ph*C*(4)H), 131.7 (C(6)- Ph*C*(1)), 135.7 (C(5)-HetAr*C*), 142.8 (furanyl*C*(5)H), 152.3 (furanyl*C*(2)), 152.4 (C(5)-HetAr*C*), 154.6 (*C*(6)), 163.8 (C(5)-HetAr*C*=N), 166.3 (*C*(2)). Data in agreement with the literature.[11]

(3*S*)-4-benzoyl-3-(thien-2-yl)-2,3-dihydro-1*H*-benzo[4,5]thiazolo[3,2-*a*]pyridin-1-one 21

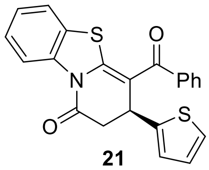

21

21 (major): Purification of the residue by silica gel chromatography column using 10:2 CH_2Cl_2/hexane as eluent gave the product **21** (major) (yield 82%, 97:3 er) and **22** (minor); mp 150–153 °C; $[\alpha]_D^{20}$ −51.1 (c 1.0, $CHCl_3$); HPLC analysis, ChiralPak AD-H (20% *i*-PrOH : hexane, flow rate 1 mL·min^{-1}, 254 nm, 30 °C), t_R (*R*)-**21**: 18.0 min, t_R (*S*)-**21**: 28.2 min, 97:3 er; ν_{max} (film)/cm^{-1} 3071 (C–H), 1719 (C=O), 1595 (C=C), 1477 (C–N), 1362 (C–S); **^{1}H NMR** (400 MHz, $CDCl_3$) δ_H: 3.17 (1H, dd, *J* 16.1, 6.1, C(2)H_AH_B), 3.25 (1H, dd, *J* 16.1 2.5, C(2)H_AH_B), 4.45 (1H, ddd, *J* 6.1, 2.6, 1.0, C(3)*H*-Ar), 5.99 (1H, m, Ar*H*), 6.25 (1H, dd, *J* 3.3, 1.9, Ar*H*), 7.29-7.51 (8H, m, Ar*H*), 7.54-7.61 (1H, m, Ar*H*), 8.46-8.53 (1H, m, Ar*H*); **^{13}C{1H} NMR** (100 MHz, $CDCl_3$) δ_C: 34.3 (*C*(3)H), 41.6 (*C*(2)H_2), 108.4 (*C*(4)), 117.7 (*C*(9)H), 122.0 (*C*(6)H), 124.8 (thiophene*C*(3)H), 125.1 (thiophene*C*(4)H), 126.0 (*C*(7)H), 127.1 (C(4)-Ar*C*(2,6)H), 127.1 (thiopheneC(5)H), 127.4 (*C*(8)H), 127.7 (*C*(5a)), 128.3 (C(4)-Ar*C*(3,5)H), 130.4 (C(4)-Ar*C*(4)H), 136.0 (C(4)-Ar*C*(1)), 139.3 (*C*(9a)), 144.9 (thiophene*C*(2)), 156.5 (*C*(4a)), 167.6 (*C*(1)=O), 190.7 (C(4)*C*=O); HRMS (APCI$^+$) $C_{22}H_{16}NO_2S_2$ ($[M+H]^+$) requires 390.0617, found 390.0611 (−1.5 ppm).

(4*S*)-5-(benzo[d]thiazol-2-yl)-6-phenyl-4-(thien-2-yl)-3,4-dihydro-2*H*-pyran-2-one 22

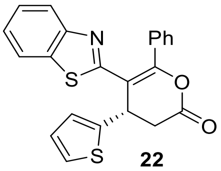

22

22 (minor): (yield 18%, 87:13 er); mp 135–138 °C; $[\alpha]_D^{20}$ +3.1 (c 1.0 in $CHCl_3$); chiral HPLC analysis, ChiralPak AD-H (20% *i*-PrOH : hexane, flow rate 1 mL·min^{-1}, 254 nm, 30 °C), t_R (*R*)-**22**: 8.6 min, t_R (*S*)-**22**: 17.9 min, 87:13 er; ν_{max} (film)/cm^{-1} 3067 (C–H), 2953 (C–H), 2922 (C–H), 1782 (C=O), 1647 (C=N), 1595 (C=C), 1343 (C–S); **^{1}H NMR** (400 MHz, $CDCl_3$) δ_H: 3.21 (1H, dd, *J* 15.9, 6.7, C(3)*H*$_A$H$_B$), 3.28 (1H, dd, *J* 15.9, 1.9, C(3)H$_A$*H*$_B$), 5.14 (1H, dd, *J* 6.8, 1.6, C(4)*H*-Ar), 6.19 (1H, d, *J* 3.3, Ar*H*), 6.26 (1H, dd, *J* 3.3, 1.9, Ar*H*), 7.31 (1H, t, *J* 7.6, Ar*H*), 7.34 (1H, d, *J* 1.8, Ar*H*), 7.44 (3H, td, *J* 7.3, 1.5, Ar*H*), 7.49-7.57 (3H, m, Ar*H*), 7.61-7.68 (1H, m, Ar*H*), 7.99 (1H, d, *J* 8.2, Ar*H*); **^{3}C{1H} NMR** (100 MHz, $CDCl_3$) δ_C: 36.2 (*C*(4)H), 36.8 (*C*(3)H$_2$), 115.8 (*C*(5)), 121.2 (C(5)-HetAr*C*(7)H), 123.1 (C(5)-HetAr*C*(4)H), 124.6 (thiophene*C*(3)H), 124.9 (thiophene*C*(4)H), 125.5 (C(5)-HetAr*C*(6)H), 126.1 (C(5)-HetAr*C*(5)H), 127.1 (thiophene*C*(5)H), 129.0 (C(6)-PhC(3,5)*H*), 130.0 (C(6)-Ph*C*(2,6)H), 130.9 (C(6)-Ph*C*(4)H), 131.7 (C(6)-Ph*C*(1)), 135.8 (C(5)-HetArC), 142.8 (thiophene*C*(2)), 152.3 (C(5)-HetArC), 154.2 (*C*(6)), 163.6 (C(5)-HetAr*C*=N), 166.3 (*C*(2)); HRMS (APCI$^+$) $C_{22}H_{16}NO_2S_2$ ([M+H]$^+$) requires 390.0617, found 390.0615 (−0.5 ppm).

(3*S*)-4-benzoyl-3-methyl-2,3-dihydro-1*H*-benzo[4,5]thiazolo[3,2-*a*]pyridin-1-one 23

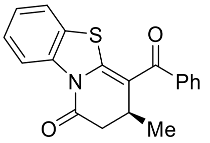

23

23 (major): Purification of the residue by silica gel chromatography column using 10:2 CH_2Cl_2/hexane as eluent gave the product **23** (major) (yield 78%, 96:4 er) and **24** (minor); $[\alpha]_D^{20}$ +97.4 (c 1.0 in $CHCl_3$); chiral HPLC analysis, ChiralPak AD-H (20% *i*-PrOH : hexane, flow rate 1 mL·min^{-1}, 254 nm, 30 °C), t_R (*R*)-**23**: 11.5 min, t_R (*S*)-**23**: 14.4 min, 96:4 er; **^{1}H NMR** (400 MHz, $CDCl_3$) δ_H: 1.11 (3H, d, *J* 7.0, C*H*$_3$), 2.72 (1H, dd, *J* 16.0, 2.2, C(2)*H*$_A$H$_B$), 3.08 (1H, dd, *J* 16.0, 6.3, C(2)H$_A$*H*$_B$), 3.31 (1H, pd, *J* 6.9, 2.2, C(3)*H*-CH$_3$), 7.2–7.40 (2H, m, Ar*H*), 7.42–7.38 (6H, m, Ar*H*), 8.48–8.54 (1H, m, Ar*H*); **^{3}C{1H} NMR** (100 MHz, $CDCl_3$) δ_C: 19.6 (C(3)H-CH$_3$), 27.8 (*C*(2)H), 40.0 (*C*(2)H$_2$), 110.5 (*C*(4)), 117.5 (*C*(9)H), 121.9 (*C*(6)H), 125.7 (*C*(7)H), 126.8 (C(4)-ArC(3,5)H), 126.9 (*C*(8)H), 127.7 (*C*(5a)), 128.4 (C(4)-Ar*C*(2,6)H), 130.0 (C(4)-Ar*C*(4)H), 136.1 (*C*(9a)), 140.0 (C(4)-Ar*C*(1)), 154.2 (*C*(4a)), 168.9 (*C*(1)=O), 191.4 (C(4)*C*=O). Data in agreement with the literature.[11]

(4*S*)-5-(benzo[d]thiazol-2-yl)-4-methyl-6-phenyl-3,4-dihydro-2*H*-pyran-2-one 24

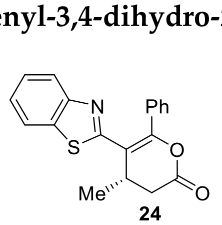

24

24 (minor): (yield 18%, 95:5 er); $[\alpha]_D^{20}$ +18.7 (c 1.0 in $CHCl_3$); chiral HPLC analysis, ChiralPak AD-H (20% *i*-PrOH : hexane, flow rate 1 mL·min^{-1}, 254 nm, 30 °C), t_R (*R*)-**24**: 5.9 min, t_R (*S*)-**24**: 9.5 min, 95:5 er; **^{1}H NMR**

(400 MHz, $CDCl_3$) δ_H: 1.33 (3H, d, *J* 7.1, C*H*$_3$), 2.81 (1H, dd, *J* 15.7, 2.0, C(3)*H*$_A$H$_B$), 3.04 (1H, dd, *J* 15.7, 6.7, C(3)H$_A$*H*$_B$), 3.84 (1H, pd, *J* 7.1, 2.0, C(4)*H*-CH_3), 7.30–7.37 (1H, m, Ar*H*), 7.37–7.52 (6H, m, Ar*H*), 7.63 – 7.68 (1H, m, Ar*H*), 7.95 – 8.01 (1H, m, Ar*H*); ^{3}**C{1H} NMR** (100 MHz, $CDCl_3$) δ_C: 18.9 (*C*(4)H-CH_3), 30.7 (*C*(4)H), 36.0 (*C*(3)H_2), 117.8 (*C*(5)), 121.2 (C(5)-HetAr*C*(4)H), 123.0 (C(5)-HetAr*C*(7)H), 125.4 (C(5)-HetAr*C*(4)H), 126.1 (C(5)-HetAr*C*(5)H), 129.0 (C(6)- Ph*C*(3,5)H), 130.0 (C(6)-Ph*C*(2,6)H), 130.6 (C(6)-Ph*C*(4)H), 132.0 (C(6)-Ph*C*(1)), 135.6 (C(5)-HetAr*C*), 152.6 (C(5)-HetAr*C*), 153.1 (*C*(6)), 164.1 (C(5)-HetAr*C*=N), 167.5 (C(2)). Data in agreement with the literature.[11]

(7*R*)-8-benzoyl-7-phenyl-6,7-dihydro-5*H*-thiazolo[3,2-a]pyridin-5-one 25

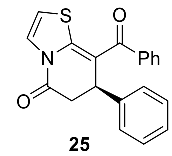

25

25: Purification of the residue by silica gel chromatography column using 10:1 hexane/ethyl acetate as eluent gave the product **25** (yield 94%, 94:6 er); mp 152–158 °C; $[\alpha]_D^{20}$ +6.25 (c 1.0 in $CHCl_3$); chiral HPLC analysis, ChiralPak AD-H (5% *i*-PrOH : hexane, flow rate 1 mL·min^{-1}, 254 nm, 30 °C), t_R (*S*)-**25**: 31.9 min, t_R (*R*)-**25**: 37.5 min, 94:6 er; ν_{max} (film)/cm^{-1} 3113 (C–H), 3061 (C–H), 2955 (C–H), 2922 (C–H), 1722 (C=O), 1630 (C=C), 1599 (C=C), 1474 (C–N), 1343 (C–S); ^{1}**H NMR** (400 MHz, $CDCl_3$) δ_H: 3.02 (1H, dd, *J* 16.3, 2.2, C(2)*H*$_A$H$_B$), 3.29 (1H, dd, *J* 16.3, 7.0, C(2)H$_A$*H*$_B$), 4.40 (1H, dd, *J* 7.0, 2.2, C(3)*H*-Ph), 6.54 (1H, d, *J* 4.7, C(7)*H*), 7.03-7.09 (2H, m, Ar*H*), 7.23-7.28 (2H, m, Ar*H*), 7.29-7.44 (6H, m, Ar*H*), 7.56 (1H, d, *J* 4.7, C(6)*H*); ^{13}**C{1H} NMR** (100 MHz, $CDCl_3$) δ_C: 38.8 (*C*(3)H), 39.9 (*C*(2)H_2), 105.7 (*C*(4)), 111.1 (*C*(7)H), 121.5 (*C*(6)H), 126.7 (C(3)H-Ph*C*(2,6)H), 127.0 (C(4)-Ar*C*(3,5)H), 127.5 (C(3)H-Ph*C*(4)H), 128.1 (C(4)-Ar*C*(2,6)H), 129.3 (C(3)H-Ph*C*(3,5)H), 130.0 (C(4)-Ar*C*(4)H), 139.6 (C(4)-Ar*C*(1)), 141.2 (C(3)-Ph*C*(1)), 156.8 (*C*(4a)), 166.1 (*C*(1)=O), 189.5 (C(4)*C*=O); HRMS (NSI$^+$) $C_{20}H_{15}NO_2S$ ([M+H]$^+$) requires 334.0896, found 334.0894 (−0.6 ppm).

(7*S*)-8-benzoyl-7-methyl-6,7-dihydro-5*H*-thiazolo[3,2-a]pyridin-5-one 26

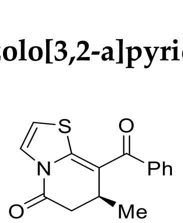

26

26: Purification of the residue by silica gel chromatography column using 10:1 hexane/ethyl acetate as eluent gave the product **26** (yield 97%, 91:9 er); mp 115–120 °C; $[\alpha]_D^{20}$ +149.87 (c 1.0 in $CHCl_3$); chiral HPLC analysis, ChiralPak AD-H (5% *i*-PrOH : hexane, flow rate 1 mL·min^{-1}, 254 nm, 30 °C), t_R (*R*)-**26**: 24.1 min, t_R (*S*)-**26**: 28.7 min, 91:9 er; ν_{max} (film)/cm^{-1} 3115 (C–H), 3098 (C–H), 2963 (C–H), 1703 (C=O), 1595 (C=C), 1468 (C–N), 1356 (C–S); ^{1}**H NMR** (400 MHz, $CDCl_3$) δ_H: 1.06 (3H, d, *J* 7.0, C*H*$_3$), 2.70 (1H, dd, *J* 16.5, 2.1, C(2)*H*$_A$H$_B$), 2.97 (1H,

dd, *J* 16.5, 6.43, C(2)H_A*H_B*), 3.36 (1H, pd, *J* 7.0, 2.1, C(3)*H*-CH_3), 6.49 (1H, d, *J* 4.8, C(7)*H*), 7.42-7.49 (3H, m, Ar*H*), 7.49-7.55 (2H, m, Ar*H*), 7.59 (1H, d, *J* 4.8, C(6)*H*); **^{13}C{1H} NMR** (100 MHz, $CDCl_3$) δ_C: 19.8 (C(3)H-CH_3), 28.2 (*C*(3)H), 38.6 (*C*(2)H_2), 108.6 (*C*(4)), 111.0 (*C*(7)H), 121.4 (*C*(6)H), 126.9 (C(4)-Ar*C*(3,5)H), 128.3 (C(4)-Ar*C*(2,6)H), 129.8 (C(4)-Ar*C*(4)H), 140.0 (C(4)-Ar*C*(1)), 154.8 (*C*(4a)), 167.0 (*C*(1)=O), 189.5 (C(4)*C*=O); HRMS (NSI^+) $C_{15}H_{14}NO_2S$ ($[M+H]^+$) requires 272.0740, found 272.0739 (−0.4 ppm).

(4*R*)-5-(benzo[d]oxazol-2-yl)-4,6-diphenyl-3,4-dihydro-2*H*-pyran-2-one 27

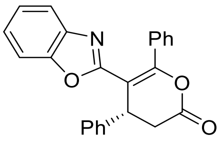

27

27: Purification of the residue by silica gel chromatography column using 10:1 hexane/ethyl acetate as eluent gave the product **27** (yield 43%, 98:2 er); $[\alpha]_D^{20}$ +32.1 (c 1.0 in $CHCl_3$); chiral HPLC analysis, ChiralPak OJ-H (10% *i*-PrOH : hexane, flow rate 1 mL·min^{-1}, 254 nm, 30 °C), t_R (*R*)-**27**: 19.6 min, t_R (*S*)-**27**: 25.2 min, 98:2 er; **^{1}H NMR** (400 MHz, $CDCl_3$) δ_H: 3.11 (1H, dd, *J* 15.7, 1.8, C(3)*H_A*H_B), 3.30 (1H, dd, *J* 15.7, 7.5, C(3)H_A*H_B*), 4.90 (1H, dd, *J* 7.5, 1.8, C(4)*H*-Ph), 7.18-7.45 (10H, m, Ar*H*), 7.46-7.60 (3H, m, Ar*H*), 7.63-7.69 (1H, m, Ar*H*); **^{13}C{1H} NMR** (100 MHz, $CDCl_3$) δ_C: 36.6 (*C*(3)H_2), 40.3 (*C*(4)H), 107.5 (*C*(5)), 110.5 (C(5)-HetAr*C*(7)H), 119.9 (C(5)-HetAr*C*(4)H), 124.5 (C(5)-HetA*rC*(6)H), 125.3 (C(5)-HetAr*C*(5)H), 126.9 (2×PhCH), 127.9 (C(4)H-Ph*C*(4)H), 128.2 (2×PhCH), 129.0 (2×PhCH), 129.3 (2×PhCH), 130.5 (C(6)- Ph*C*(4)H), 132.6 (C(6)- Ph*C*(1)), 139.4 (C(4)-Ph*C*(1)), 141.5 (C(5)-HetAr*C*), 150.2 (C(5)-HetAr*C*), 156.0 (*C*(6)), 160.9 (C(5)-HetAr*C*=N), 166.2 (*C*(2)). Data in agreement with the literature.[11]

(4*S*)-5-(benzo[d]oxazol-2-yl)-4-methyl-6-phenyl-3,4-dihydro-2*H*-pyran-2-one 28

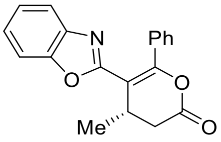

28

28: Purification of the residue by silica gel chromatography column using 10:1 hexane/ethyl acetate as eluent gave the product **28** (yield 82%, 92:8 er); $[\alpha]_D^{20}$ +76.3 (c 1.0 in $CHCl_3$); chiral HPLC analysis, ChiralPak AD-H (5% *i*-PrOH : hexane, flow rate 1 mL·min^{-1}, 254 nm, 30 °C), t_R (*R*)-**28**: 9.1 min, t_R (*S*)-**28**: 15.3 min, 92:8 er; **^{1}H NMR** (400 MHz, $CDCl_3$) δ_H: 1.36 (3H, d, *J* 7.1, C*H_3*), 2.81 (1H, dd, *J* 15.7, 1.8, C(3)H_A*H_B*), 3.01 (1H, dd, *J* 15.7, 7.5, C(3)H_A*H_B*), 3.67 (1H, dd, *J* 7.5, 1.8, C(4)*H*-C*H_3*), 7.22-7.34 (3H, m, Ar*H*), 7.34-7.42 (2H, m, Ar*H*), 7.43-7.49 (3H, m, Ar*H*), 7.70-7.74 (1H, m, Ar*H*); **^{13}C{1H} NMR** (100 MHz, $CDCl_3$) δ_C: 19.1 (C(4)H-CH_3), 29.8 (*C*(4)H), 35.6 (*C*(3)H_2), 109.9 (*C*(5)), 110.5 (C(5)-HetAr*C*(7)H), 119.8 (C(5)-HetAr*C*(4)H), 124.6 (C(5)-HetAr*C*(6)H), 125.3

(C(5)-HetArC(5)H), 128.1 (C(6)-PhC(3,5)H), 129.0 (C(6)-PhC(2,6)), 130.2 (C(6)-PhC(4)H), 132.8 (C(6)-PhC(1)), 141.5 (C(5)-HetArC), 150.2 (C(5)-HetArC), 154.9 (C(6)), 161.1 (C(5)-HetArC=N), 167.1 (C(2)). Data in agreement with the literature.[11]

4-benzoyl-3-phenyl-3,5-dihydrobenzo[4,5]imidazo[1,2-*a*]pyridin-1(2*H*)-one S27 and 4-benzoyl-3-phenyl-3,4-dihydrobenzo[4,5]imidazo[1,2-*a*]pyridin-1(2*H*)-one S28

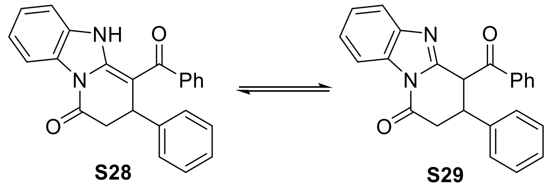

S28 and **S29**: Purification of the residue by silica gel chromatography column using 10:1 hexane/ethyl acetate as eluent gave the product **S28** and **S29** (yield 95%); **S28**: **^{1}H NMR** (400 MHz, $CDCl_3$) δ_H: 3.13 (1H, dd, *J* 16.3, 2.5, C(12)*H*H), 3.29 (1H, dd, *J* 16.3, 6.2, C(12)H*H*), 4.40 (1H, dd, *J* 6.2, 2.5, C(11)*H*), 7.12-7.18 (2H, m, Ar*H*), 7.21-7.44 (8H, m, Ar*H*), 7.49-7.58 (1H, m, Ar*H*), 7.60-7.73 (2H, m, Ar*H*), 8.18 (1H, m, Ar*H*); **S29**: **^{1}H NMR** (400 MHz, $CDCl_3$) δ_H: 3.20 (1H, dd, *J* 17.5, 8.0, C(12)*H*H), 3.43 (1H, dd, *J* 17.5, 5.0, C(12)H*H*), 4.11 (1H, ddd, *J* 8.0, *J* 6.8, *J* 5.0, C(11)H), 5.50 (1H, d, *J* 6.8, C(10)H), 7.19-7.33 (4H, m, ArH), 7.36-7.46 (3H, m, ArH), 7.49-7.56 (2H, m, ArH), 7.61-7.73 (2H, m, ArH), 8.03-8.09 (2H, m, ArH), 8.29-8.34 (1H, m, ArH).

(4*R*)-2-(2-oxo-4,6-diphenyl-3,4-dihydro-2*H*-pyran-5-yl)-1*H*-benzo[d]imidazole-1-carboxylic acid 29

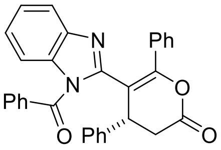

29: Purification of the residue by silica gel chromatography column using 10:1 hexane/ethyl acetate as eluent gave the product **29** (yield 72%, 90:10 er); mp 85–90 °C; $[\alpha]_D^{20}$ +267.3 (c 1.0 in $CHCl_3$); chiral HPLC analysis, ChiralPak AD-H (20% *i*-PrOH : hexane, flow rate 1 mL·min^{-1}, 254 nm, 30 °C), t_R (*S*)-**29**: 6.0 min, t_R (*R*)-**29**: 34.1 min, 90:10 er; ν_{max} (film)/cm^{-1} 2959 (C–H), 2922 (C–H), 1732 (C=O), 1599 (C=C), 1526 (C=C); **^{1}H NMR** (400 MHz, $CDCl_3$) δ_H: 3.39 (1H, dd, *J* 16.8, 2.9, C(3)H_AH_B), 3.45 (1H, dd, *J* 16.8, 5.0, C(3)H_AH_B), 4.66 (1H, dd, *J* 5.0, 3.0, C(4)*H*-Ph), 7.21-7.30 (3H, m, Ar*H*), 7.31-7.46 (9H, m, Ar*H*), 7.50-7.58 (4H, m, Ar*H*), 7.64-7.71 (1H, m, Ar*H*), 8.12-8.18 (1H, m, Ar*H*), 8.23-8.29 (2H, m, Ar*H*); **^{13}C{1H} NMR** (100 MHz, $CDCl_3$) δ_C: 40.8 (C(3)H_2), 41.0 (*C*(4)H), 115.3 (C(5)-HetAr*C*(7)H), 115.4 (*C*(5)), 120.2 (C(5)-HetAr*C*(4)H), 125.3 (C(5)-HetAr*C*(6)H), 125.7 (C(5)-HetAr*C*(5)H), 127.1 (C(4)H-Ph*C*(2,6)H), 127.6 (C(4)H-Ph*C*(4)H), 128.1 (C(6)-Ph*C*(3,5)H), 128.4 (C(4)H-Ph*C*(3,5)H), 128.7 (CO-Ph*C*(3,5)H), 129.4 (C(6)-Ph*C*(2,6)H), 130.0 (C(6)-Ph*C*(1)), 130.1 (C(6)-Ph*C*(4)H), 130.5 (CO-Ph*C*(2,6)H), 133.4 (CO-Ph*C*(4)H), 134.4 (C(5)-HetArC)), 139.9 (CO-Ph*C*(1)), 143.8 (C(4)H-Ph*C*(1)), 148.5

(C(5)-HetArC), 151.0 (*C*(6)), 165.4 (C(5)-Het*C*=O), 166.8 (*C*(2)); HRMS (NSI$^+$) $C_{31}H_{22}N_2O_3$ ([M+H]$^+$) requires 471.1703, found 471.1697 (−1.3 ppm).

(4*S*)-5-(1-benzoyl-1*H*-benzo[d]imidazol-2-yl)-4-methyl-6-phenyl-3,4-dihydro-2*H*-pyran-2-one 30

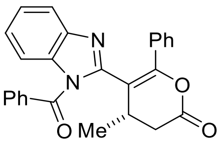

30

30: Purification of the residue by silica gel chromatography column using 10:1 hexane/ethyl acetate as eluent gave the product **30** (yield 36%, 86:14 er) and **31**; mp 136–140 °C; $[\alpha]_D^{20}$ +246.22 (c 1.0 in $CHCl_3$); chiral HPLC analysis, ChiralPak AD-H (20% *i*-PrOH : hexane, flow rate 1 mL·min^{-1}, 254 nm, 30 °C), t_R (*R*)-**30**: 7.9 min, t_R (*S*)-**30**: 46.8 min, 86:14 er; ν_{max} (film)/cm^{-1} 2955 (C–H), 2922 (C–H), 2853 (C–H), 1726 (C=O), 1635 (C=C), 1599 (C=C), 1265 (C–O); **^{1}H NMR** (400 MHz, $CDCl_3$) δ_H: 1.40 (3H, d, *J* 7.0, C*H*$_3$), 2.77 (1H, dd, *J* 16.8, 2.3, C(3)H_AH_B), 3.15 (1H, dd, *J* 16.9, 5.0, C(3)H_AH_B), 3.44 (1H, dqt, *J* 7.1, 5.1 C(4)*H*-C*H*$_3$), 7.22-7.29 (1H, m, Ar*H*), 7.30-7.39 (2H, m, Ar*H*), 7.41-7.54 (5H, m, Ar*H*), 7.56-7.69 (3H, m, Ar*H*), 8.12-8.22 (2H, m, Ar*H*), 8.21-8.29 (1H, m, Ar*H*); **^{13}C{1H} NMR** (100 MHz, $CDCl_3$) δ_C: 20.58 (C(4)-CH_3), 31.17 (*C*(4)H), 40.9 (*C*(3)H_2), 115.3 (C(5)-HetAr*C*(7)H), 118.6 (*C*(5)), 120.1 (C(5)-HetAr*C*(4)H), 125.3 (C(5)-HetAr*C*(6)H), 125.7 (C(5)-HetAr*C*(5)H), 128.4 (4×Ar*C*H), 128.8 (2×Ar*C*H), 129.9 (C(6)-Ar*C*(4)H), 130.1 (C(6)-Ar*C*(1)), 130.4 (2×Ar*C*H), 130.5 (C(5)-HetAr*C*), 133.3 (CO-Ph*C*(4)H), 134.7 (CO-Ph*C*(1)), 143.7(C(5)-HetAr*C*), 147.9(*C*(6)), 149.2(C(5)-HetAr*C*), 165.3 (C(5)-Het*C*=O), 167.7 (*C*(2)); HRMS (NSI$^+$) $C_{26}H_{20}N_2O_3$ ([M+H]$^+$) requires 409.1547, found 409.1541 (−1.5 ppm).

(3*S*,4*E*)-(3-methyl-1-oxo-2,3-dihydrobenzo[4,5]imidazo[1,2-*a*]pyridin-4(1*H*)-ylidene)(phenyl)methyl benzoate 31

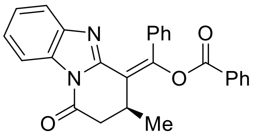

31

31: (yield 39%, 86:14 er); mp 148–154 °C; $[\alpha]_D^{20}$ +246.48 (c 1.0 in $CHCl_3$); chiral HPLC analysis, ChiralPak AD-H (20% *i*-PrOH : hexane, flow rate 1 mL·min^{-1}, 254 nm, 30 °C), t_R (*R*)-**31**: 5.1 min, t_R (*S*)-**31**: 8.5 min, 86:14 er; ν_{max} (film)/cm^{-1} 2959 (C–H), 2924 (C–H), 1734 (C=O), 1635 (C=C), 1599 (C=C); **^{1}H NMR** (400 MHz, $CDCl_3$) δ_H: 1.30 (3H, d, *J* 7.0, C*H*$_3$), 2.86 (1H, dd, *J* 17.0, 2.2, C(2)H_AH_B), 3.29 (1H, dd, *J* 17.0, 5.3, C(2)H_AH_B), 3.53 (1H, dqt, *J* 7.1, 5.2, 2.6 C(3)*H*-Ph), 7.32-7.44 (5H, m, Ar*H*), 7.53-7.59 (3H, m, Ar*H*), 7.61-7.66 (2H, m, Ar*H*), 7.66-7.72 (1H, m, Ar*H*), 8.16-8.22 (2H, m, Ar*H*), 8.29-8.34 (1H, m, Ar*H*); **^{13}C{1H} NMR** (100 MHz, $CDCl_3$) δ_C: 19.1 (C(3)H-

CH_3), 30.8 (*C*(3)H), 40.6 (*C*(2)H_2), 115.3 (*C*(9)H), 119.1 (*C*(4)), 120.2 (*C*(6)H), 125.4 (*C*(7)H), 125.6 (*C*(8)H), 128.1 ((C(4)=C-C-*C*(3,5)H), 128.8 (*C*(9a)), 128.9 (C(4)=C-OCOPh*C*(3,5)H), 129.4 (C(4)=C-C-Ph*C*(2,6)H), 129.8 ((C(4)=C-C-Ph*C*(4)H), 130.3 (C(4)=C-OCOPh*C*(2,6)H), 130.8 (*C*(5a)), 134.1 (C(4)=C-OCOPh*C*(4)H), 134.4 ((C(4)=C-C-Ph*C*(1)), 143.2 (C(4)=C-OCOPh*C*(1)), 148.3 (*C*(4a)), 150.6 (C(4)=*C*-O), 164.3 (C(4)*C*=O), 168.1 (*C*(1)=O); HRMS (NSI$^+$) $C_{26}H_{20}N_2O_3$ ([M+H]$^+$) requires 409.1547, found 409.1542 (−1.2 ppm).

(3*R*)-*N*,*N*-dimethyl-1-oxo-3-phenyl-2,3-dihydro-1*H*-benzo[4,5]thiazolo[3,2-*a*]pyridine-4-carboxamide 32

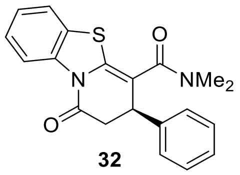

32

32: Purification of the residue by silica gel chromatography column using 10:1 hexane/ethyl acetate as eluent gave the product **32** (yield 25%, 98:2 er); $[\alpha]_D^{20}$ −73.24 (c 1.0 in $CHCl_3$); chiral HPLC analysis, ChiralPak AD-H (20% *i*-PrOH : hexane, flow rate 1 mL·min^{-1}, 254 nm, 30 °C), t_R (*R*)-**32**: 16.8 min, t_R (*S*)-**32**: 26.9 min, 98:2 er; **^{1}H NMR** (400 MHz, $CDCl_3$) δ_H: 2.85 (6H, s, N(C*H*$_3$)$_2$), 2.97 (1H, dd, *J* 16.2, 5.9, C(2)H_AH_B), 3.18 (1H, dd, *J* 16.2, 7.1, C(2)H_AH_B), 4.20 (1H, dd, *J* 7.1, 5.9, C(3)*H*-Ph), 7.14-7.18 (1H, m, Ar*H*), 7.20-7.26 (4H, m, Ar*H*), 7.27-7.33 (3H, m, Ar*H*), 8.33-8.38 (1H, m, Ar*H*); **^{13}C{^{1}H} NMR** (100 MHz, $CDCl_3$) δ_C: 36.9 (2×N-(*C*H$_3$)$_2$), 40.1 (*C*(3)H), 40.7 (*C*(2)H_2), 106.3 (*C*(4)), 117.5 (*C*(9)H), 121.3 (*C*(6)H), 125.3 (*C*(7)H), 126.0 (*C*(5a)), 126.3 (*C*(8)H), 126.8 (C(3)H-Ph*C*(2,6)H), 127.5 (C(3)H-Ph*C*(4)H), 129.1 (C(3)H-Ph*C*(3,5)H), 137.2 (*C*(9a)), 140.8 (C(3)H-Ph*C*(1)), 142.2 (*C*(4a)), 167.7 (*C*(1)=O), 169.3 (*C*ON-(CH$_3$)$_2$). Data in agreement with the literature.[11]

(3*S*)-*N*,*N*,3-trimethyl-1-oxo-2,3-dihydro-1*H*-benzo[4,5]thiazolo[3,2-*a*]pyridine-4-carboxamide 33

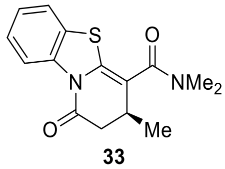

33

33: Purification of the residue by silica gel chromatography column using 10:1 hexane/ethyl acetate as eluent gave the product **33** (yield 35%, 80:20 er); $[\alpha]_D^{20}$ +7.3 (c 1.0 in $CHCl_3$); chiral HPLC analysis, ChiralPak AD-H (20% *i*-PrOH : hexane, flow rate 1 mL·min^{-1}, 254 nm, 30 °C), t_R (*S*)-**33**: 9.6 min, t_R (*R*)-**33**: 13.6 min, 80:20 er; **^{1}H NMR** (400 MHz, $CDCl_3$) δ_H: 1.19 (3H, d, *J* 6.9, C(3)H-C*H*$_3$), 2.59 (1H, dd, *J* 16.1, 6.9, C(2)H_AH_B), 2.89 (1H, dd, *J* 16.1, 6.3, C(2)H_AH_B), 3.00 (1H, dq, *J* 13.7, 6.9, C(3)*H*-Ph), 3.06 (6H, s, N-(C*H*$_3$)$_2$), 7.11 (1H, td, *J* 7.6, 1.3, C(4)*H*), 7.15-7.24 (2H, m, Ar*H*), 8.32-8.36 (1H, m, Ar*H*); **^{13}C{1H} NMR** (100 MHz, $CDCl_3$) δ_C: 18.7 (C(3)H-*C*H$_3$), 29.6 (*C*(3)H), 36.7 (2×N-(*C*H$_3$)$_2$), 40.4 (*C*(2)H_2), 108.5 (*C*(4)), 117.5 (*C*(9)H), 121.2 (*C*(6)H), 125.2 (*C*(7)H), 125.4 (*C*(5a)),

126.2 (*C*(8)H), 137.3 (*C*(9a)), 138.3 (*C*(4a)), 168.3 (*C*(1)=O), 169.5 (*C*ON-(CH_3)$_2$). Data in agreement with the literature.[11]

3 Single crystal X-ray diffraction data

X-ray diffraction data for compound **31** were collected at 173 K using a Rigaku MM-007HF High Brilliance RA generator/confocal optics [Cu Kα radiation (λ = 1.54187 Å)] with XtaLAB P100 diffractometer. Intensity data were collected (using a calculated strategy) and processed (including correction for Lorentz, polarization and absorption) using CrysAlisPro.[1] The structure was solved by dual-space methods (SHELXT[2]) and refined by full-matrix least-squares against F^2 (SHELXL-2019/3[3]). Non-hydrogen atoms were refined anisotropically, and hydrogen atoms were refined using a riding model. All calculations were performed using the Olex2[4] interface. Selected crystallographic data are presented in Table S2.

CCDC 2339361 contains the supplementary crystallographic data for this paper. These data can be obtained free of charge from The Cambridge Crystallographic Data Centre via www.ccdc.cam.ac.uk/structures.

Table S2. Selected crystallographic data.

	(*S*)-31
formula	$C_{26}H_{20}N_2O_3$
fw	408.44
crystal description	Colourless prism
crystal size [mm^3]	0.16×0.13×0.04
space group	$P2_12_12_1$
a [Å]	8.04718(9)
b [Å]	10.61221(14)
c [Å]	23.8179(3)
vol [Å]^3	2034.01(4)
Z	4
ρ (calc) [g/cm^3]	1.334
μ [mm^{-1}]	0.710
F(000)	856
reflections collected	37028
independent reflections (R_{int})	3599 (0.0307)
parameters, restraints	281, 0
GoF on F^2	1.077
R_1 [$I > 2\sigma(I)$]	0.0235
wR_2 (all data)	0.0628
largest diff. peak/hole [e/Å^3]	0.168, -0.027
Flack parameter	0.11(4)

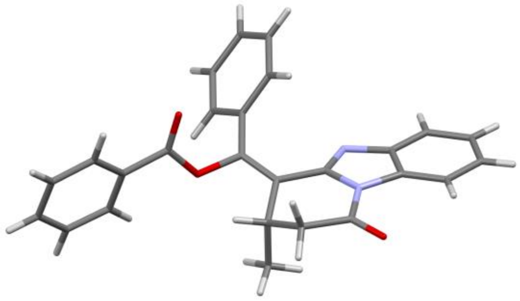

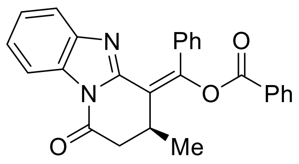

(*S*)-31

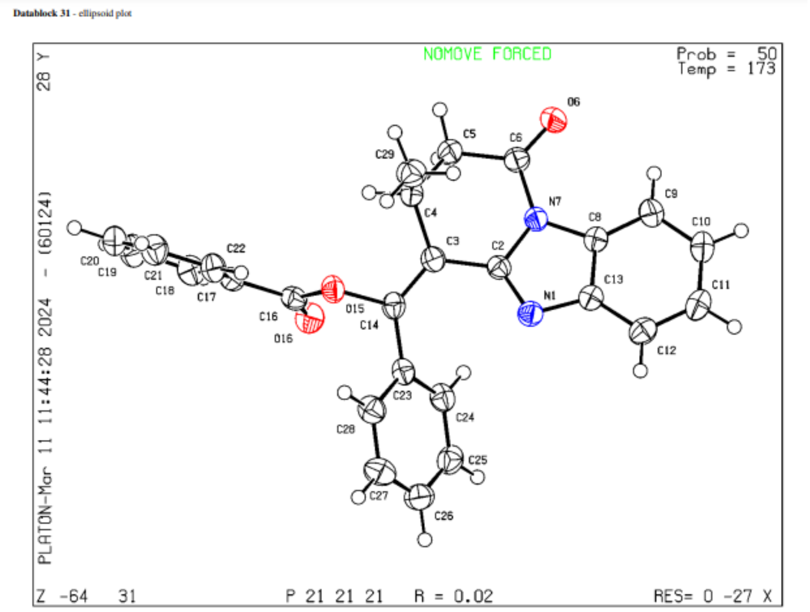

4 References

1. L. C. Morrill, J. Douglas, T. Lebl, A. M. Slawin, D. J. Fox and A. D. Smith, *Chemical Science*, 2013, **4**, 4146-4155.
2. D. S. Daniels, S. R. Smith, T. Lebl, P. Shapland and A. D. Smith, *Synthesis*, 2015, **47**, 34-41.
3. R. M. Neyyappadath, R. Chisholm, M. D. Greenhalgh, C. Rodríguez-Escrich, M. A. Pericàs, G. Hahner and A. D. Smith, *ACS Catalysis*, 2018, **8**, 1067-1075.
4. Y. Kubota, S. Tanaka, K. Funabiki and M. Matsui, *Organic letters*, 2012, **14**, 4682-4685.
5. H. I. De Silva, S. Chatterjee, W. P. Henry and C. U. Pittman Jr, *Synthesis*, 2012, **44**, 3453-3464.
6. M. D. Greenhalgh, S. Qu, A. M. Slawin and A. D. Smith, *Chemical science*, 2018, **9**, 4909-4918.
7. I. Dzvinchuk, A. Nesterenko, V. Polovinko, A. Ryabitskii and M. Lozinskii, *Chemistry of Heterocyclic Compounds*, 2011, **47**, 953-963.
8. E. R. Robinson, C. Fallan, C. Simal, A. M. Slawin and A. D. Smith, *Chemical Science*, 2013, **4**, 2193-2200.
9. W. C. Hartley, F. Schiel, E. Ermini and P. Melchiorre, *Angewandte Chemie*, 2022, e202204735.
10. Z. Yang, S. Chen, F. Yang, C. Zhang, Y. Dou, Q. Zhou, Y. Yan and L. Tang, *European Journal of Organic Chemistry*, 2019, **2019**, 5998-6002.
11. E. R. Robinson, D. M. Walden, C. Fallan, M. D. Greenhalgh, P. H.-Y. Cheong and A. D. Smith, *Chemical science*, 2016, **7**, 6919-6927.

Appendix I: ^{1}H, $^{13}C\{^{1}H\}$, 2D ^{1}H COSY, 2D ^{1}H–^{13}C HSQC and 2D ^{1}H–^{13}C HMBC NMR Spectra

(3*R*)-4-benzoyl-3-phenyl-2,3-dihydro-1*H*-benzo[4,5]thiazolo[3,2-*a*]pyridin-1-one 2

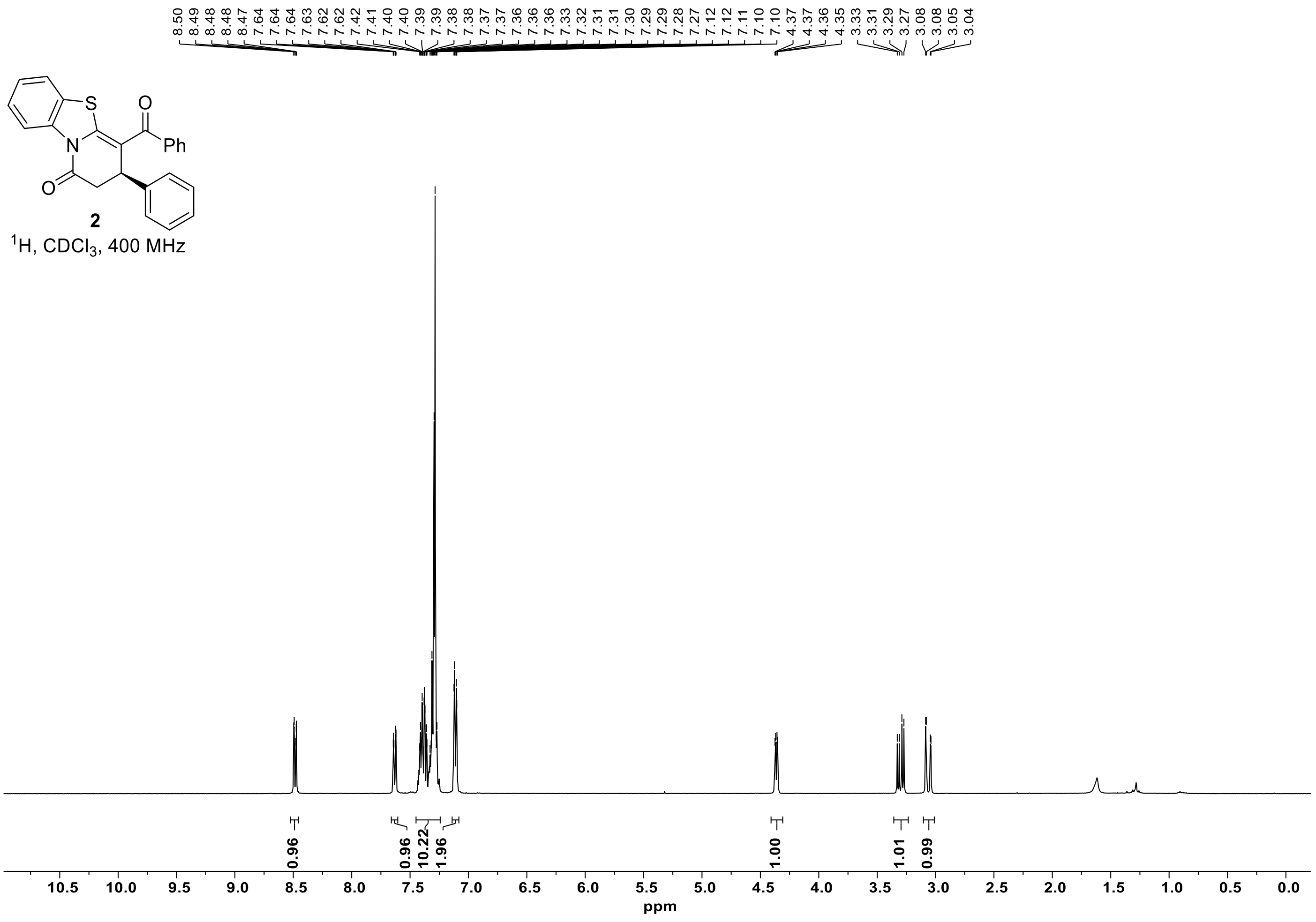

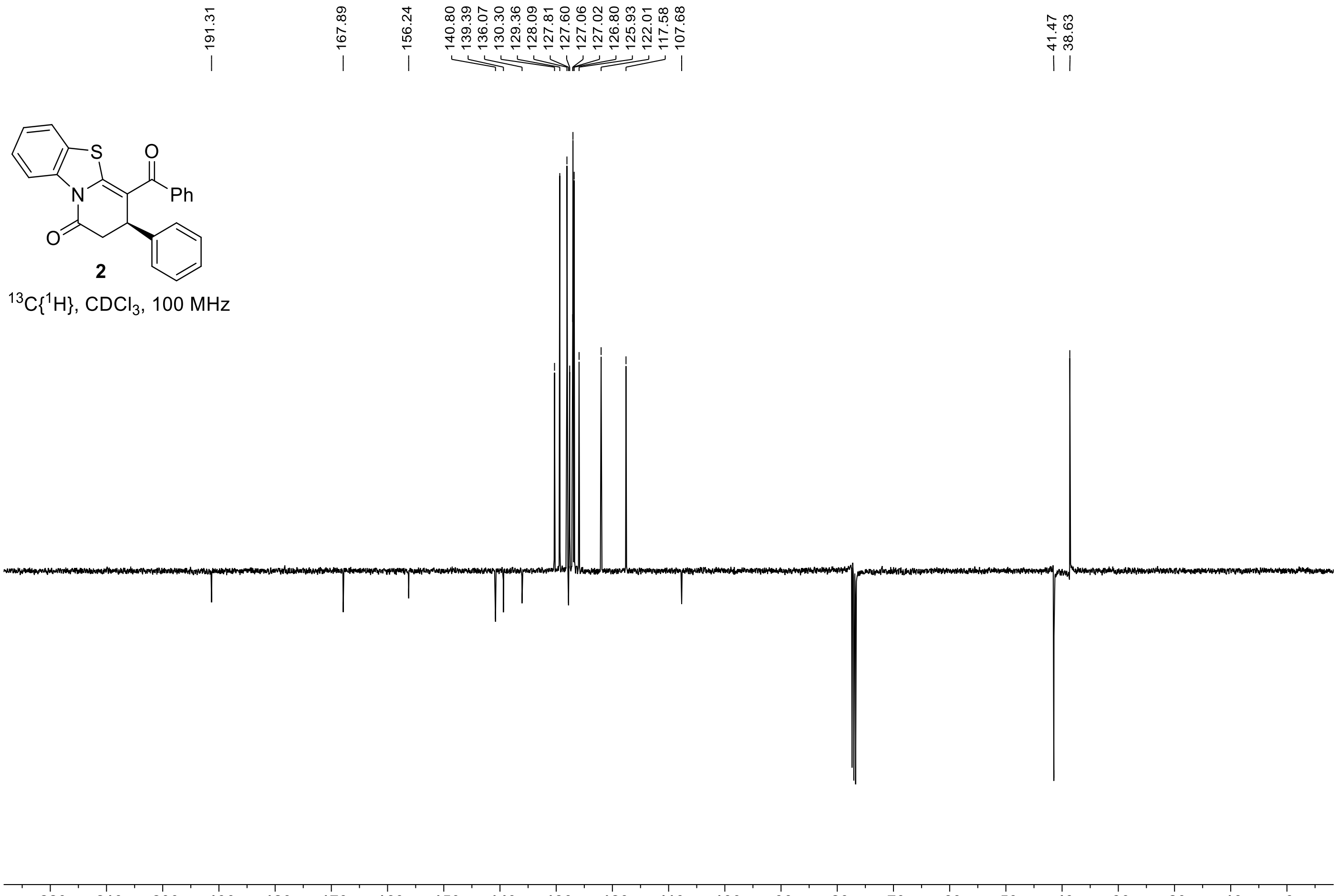

(4*R*)-5-(benzo[d]thiazol-2-yl)-4,6-diphenyl-3,4-dihydro-2*H*-pyran-2-one 3

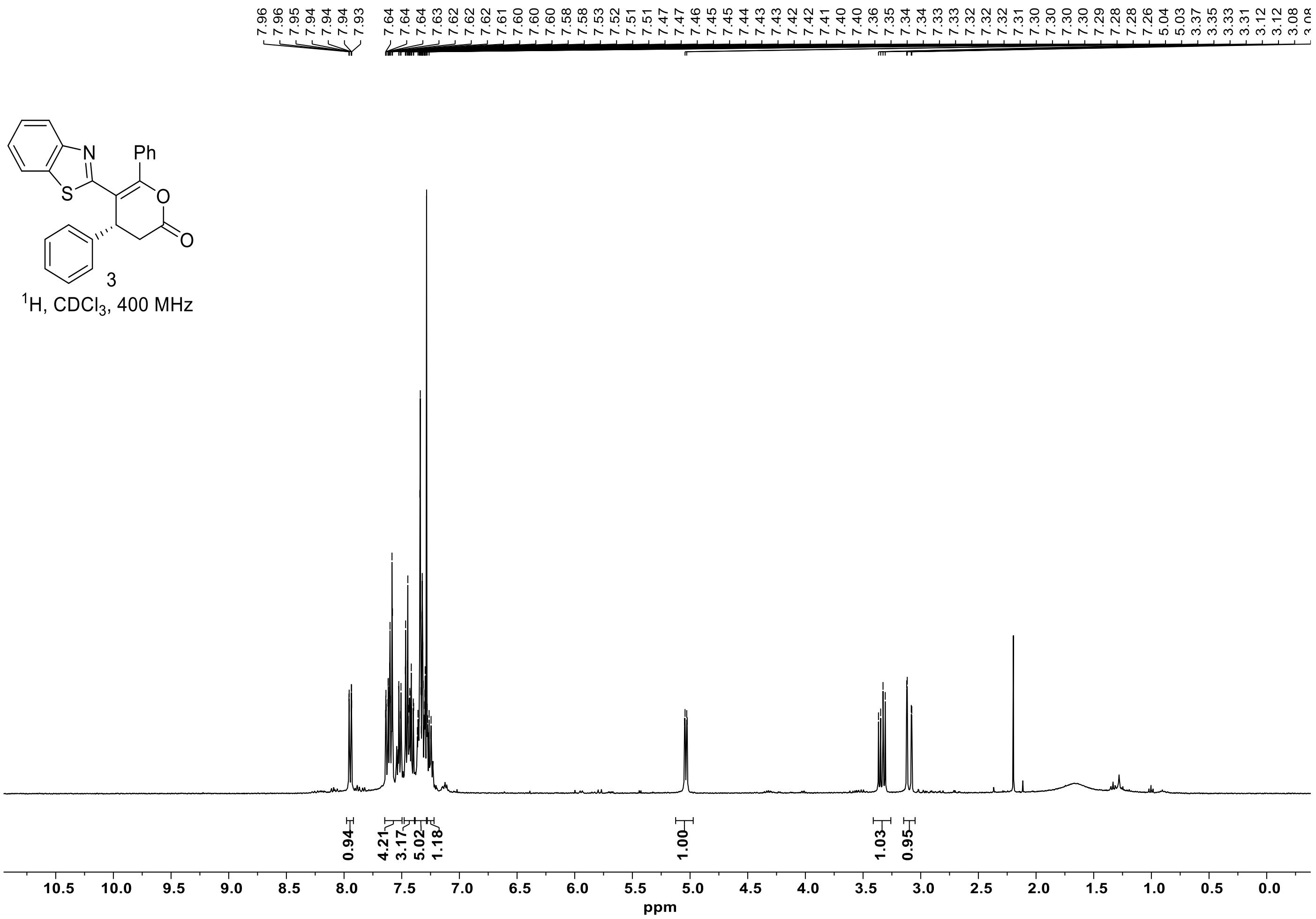

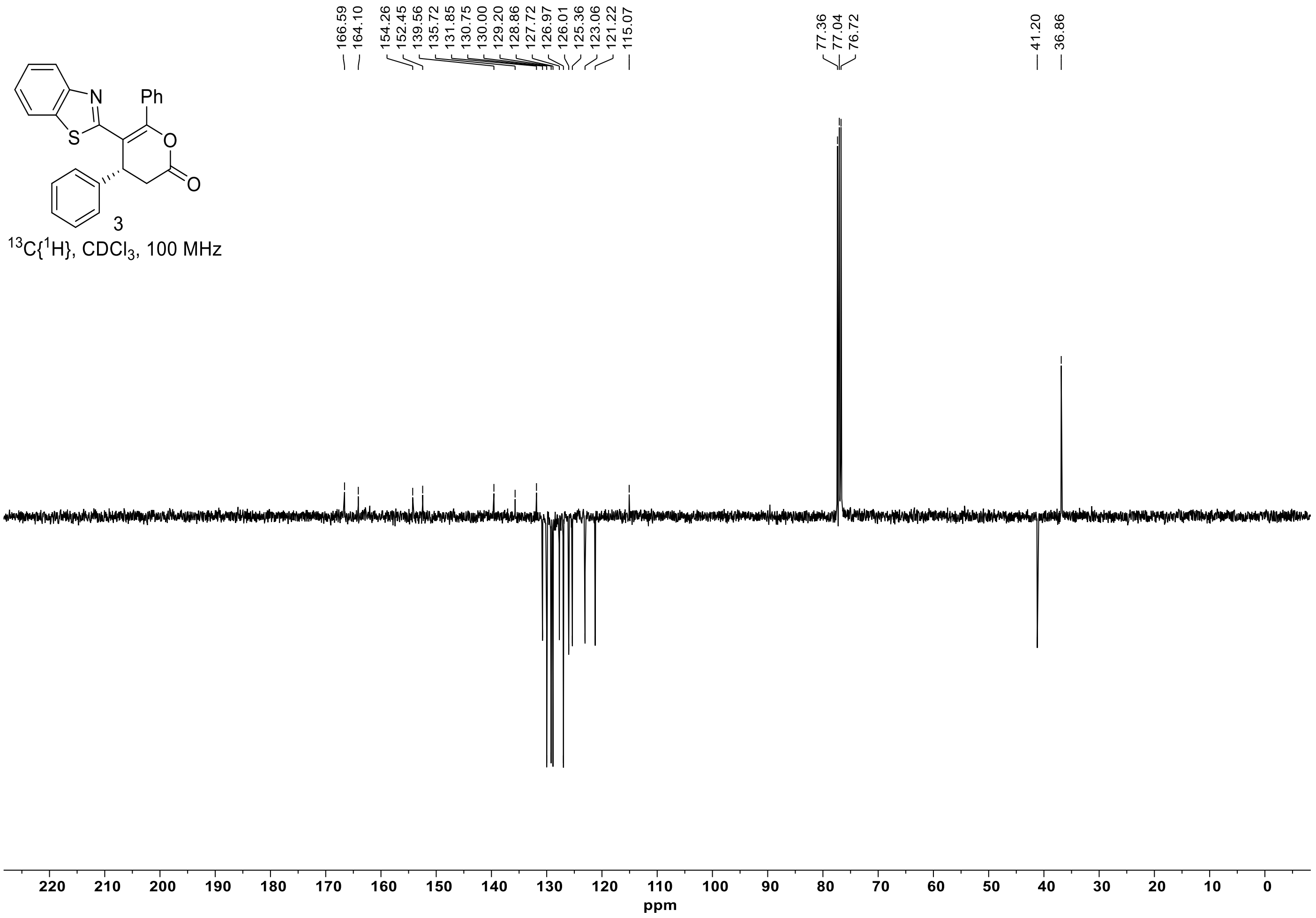

(3*R*)-4-benzoyl-3-(4-fluorophenyl)-2,3-dihydro-1*H*-benzo[4,5]thiazolo[3,2-*a*]pyridin-1-one 5

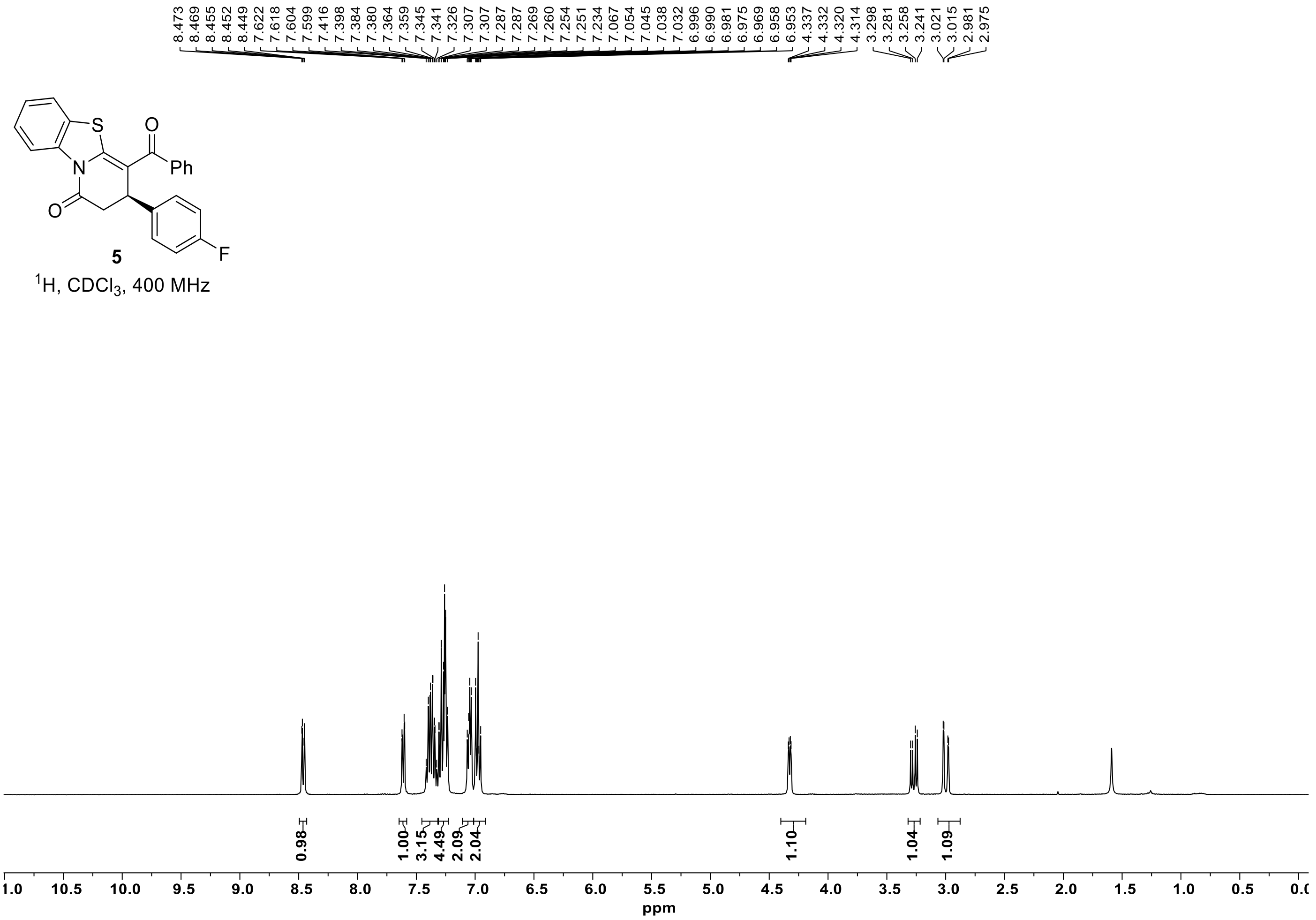

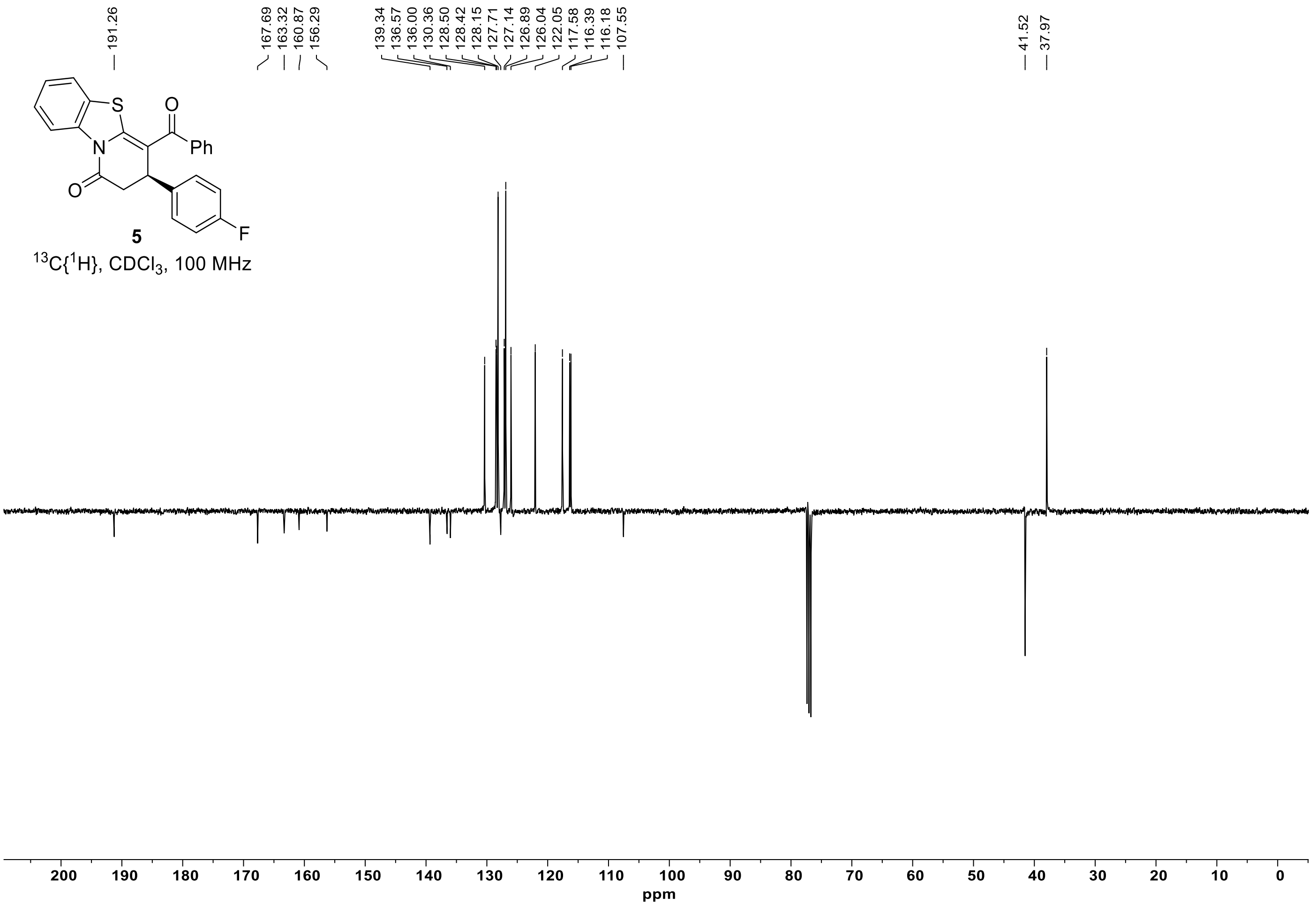

(4*R*)-5-(benzo[d]thiazol-2-yl)-4-(4-fluorophenyl)-6-phenyl-3,4-dihydro-2H-pyran-2-one 6

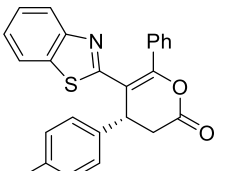

6

^{1}H, $CDCl_3$, 400 MHz

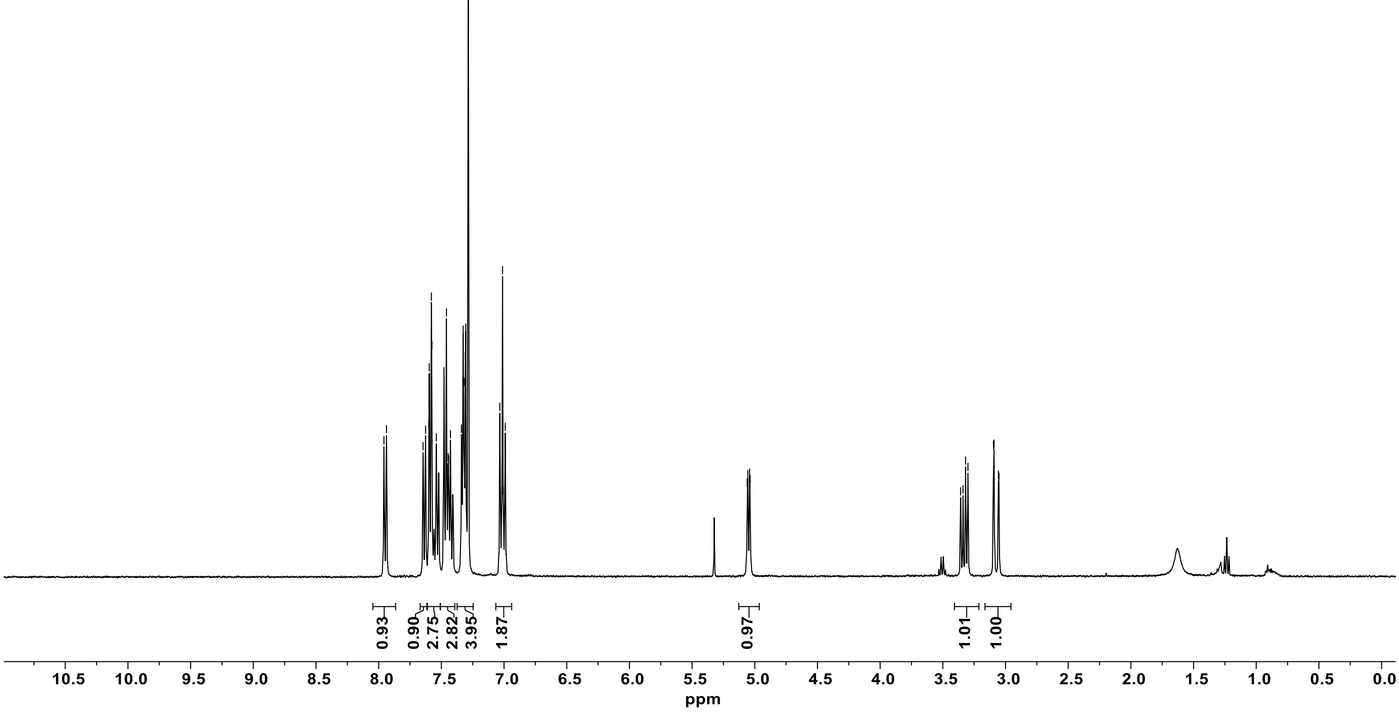

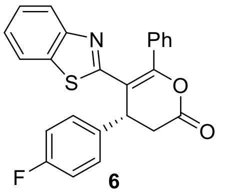

6

^{13}C{^{1}H}, $CDCl_3$, 100 MHz

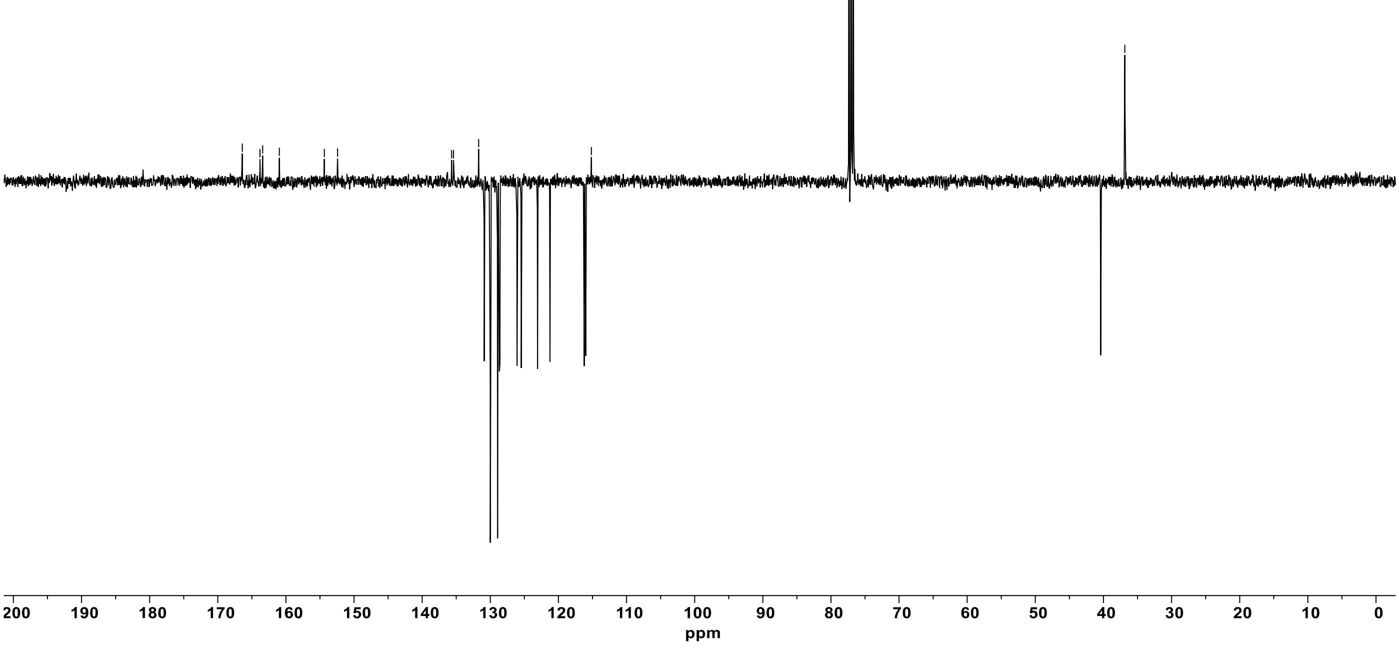

(11*R*)-4-benzoyl-3-(4-(trifluoromethyl)phenyl)-2,3-dihydro-1H-benzo[4,5]thiazolo [3,2-a]pyridin-1-one 7

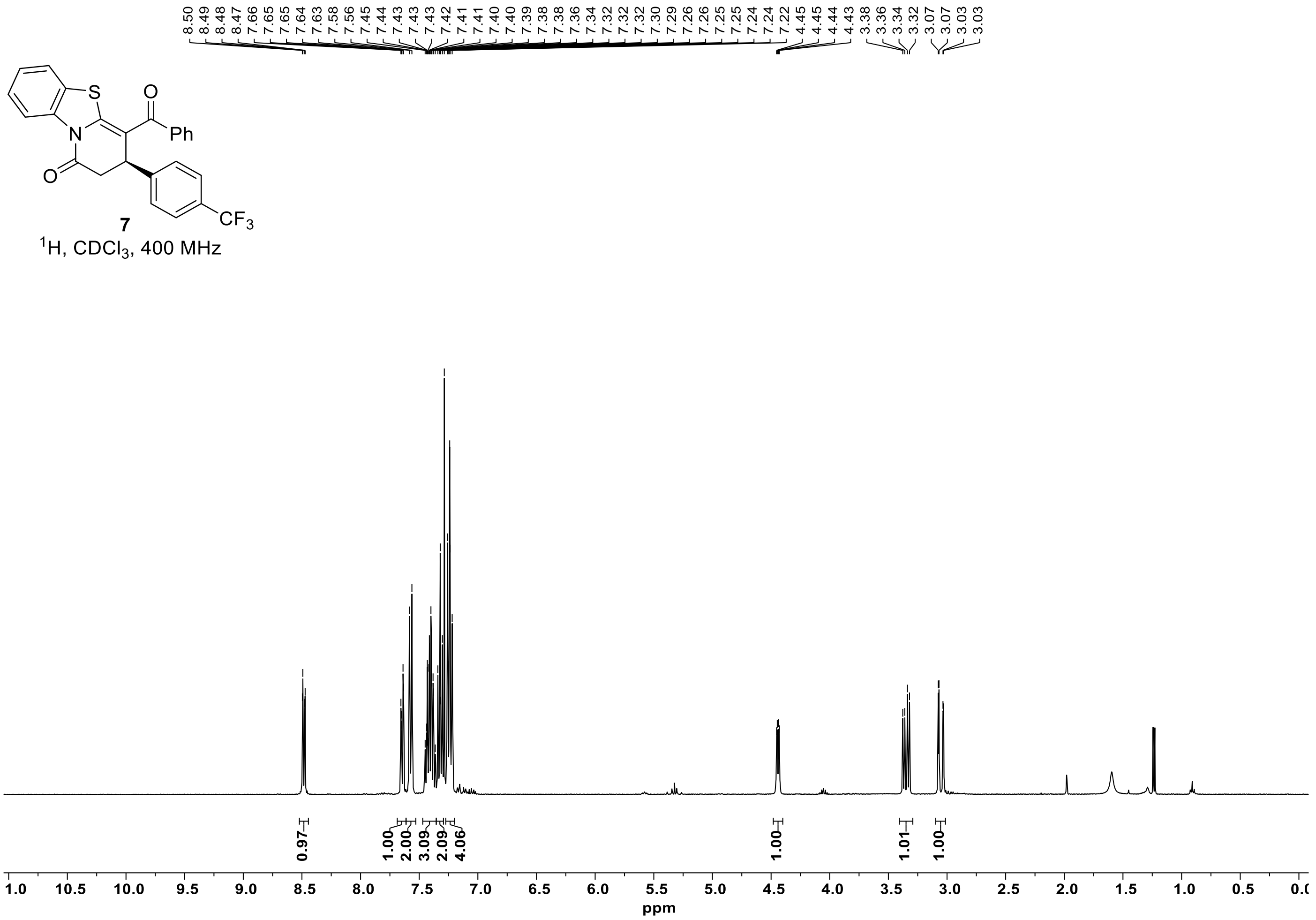

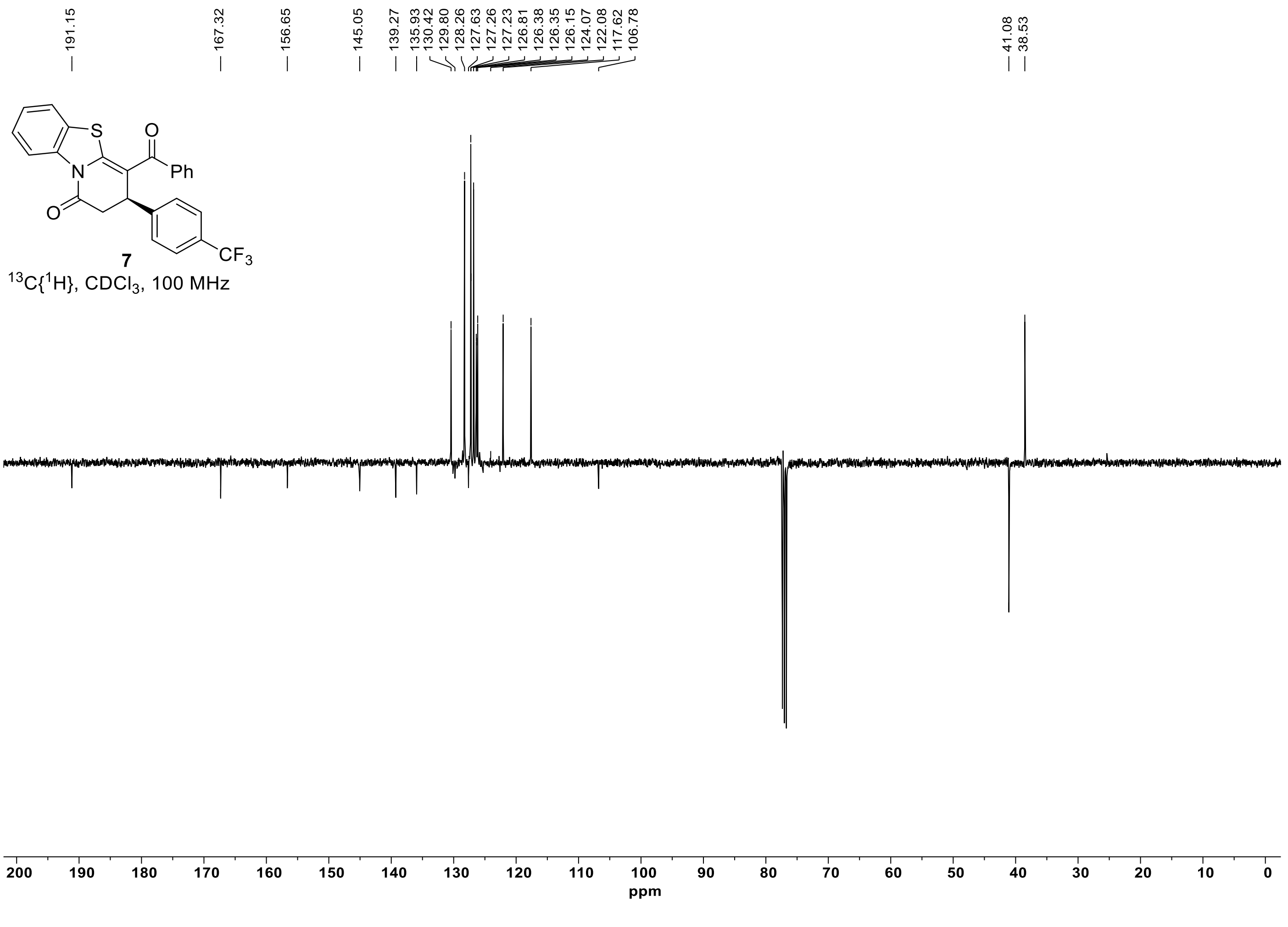

(4*R*)-5-(benzo[d]thiazol-2-yl)-6-phenyl-4-(4-(trifluoromethyl)phenyl)-3,4-dihydro-2*H*-pyran-2-one 8

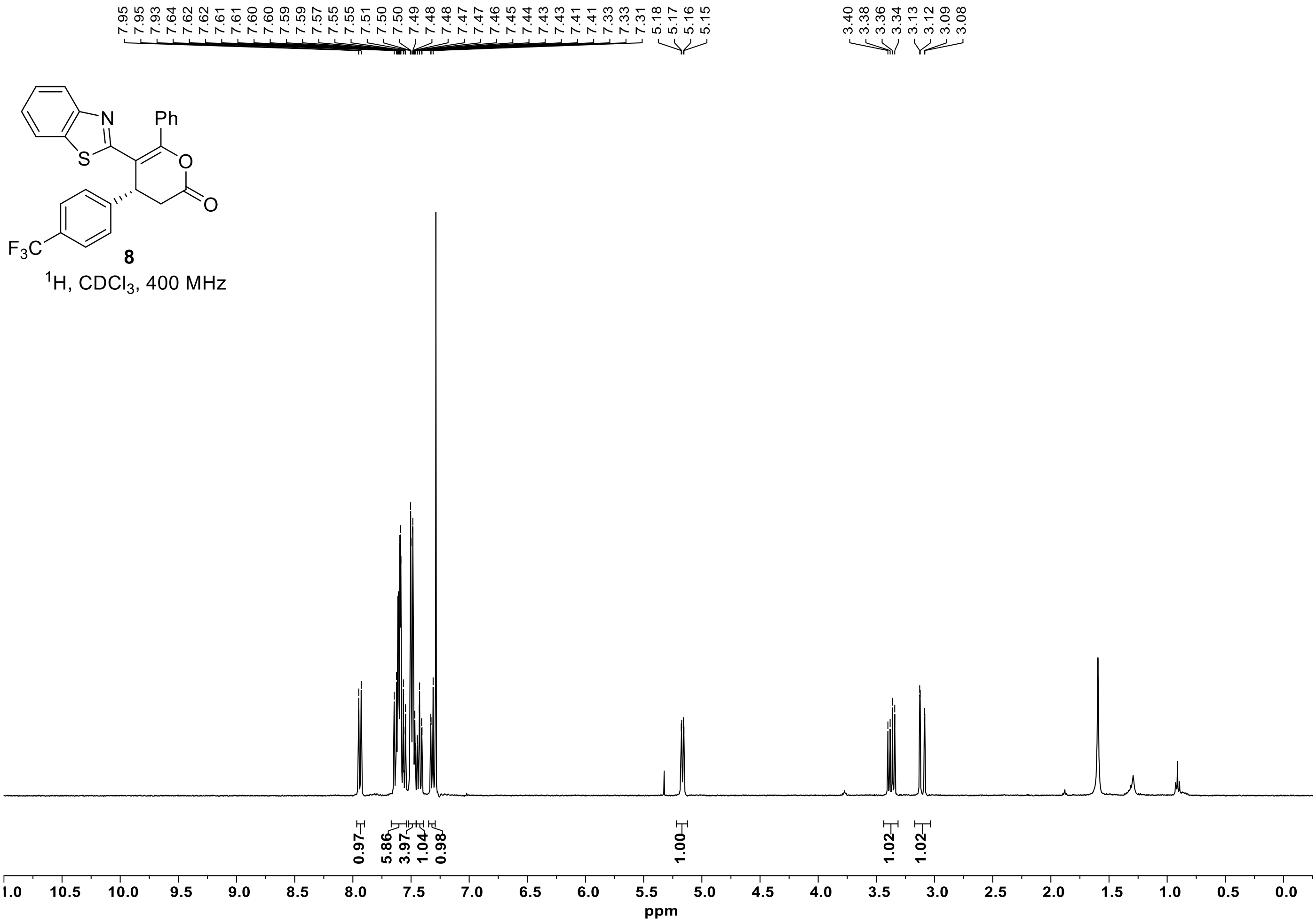

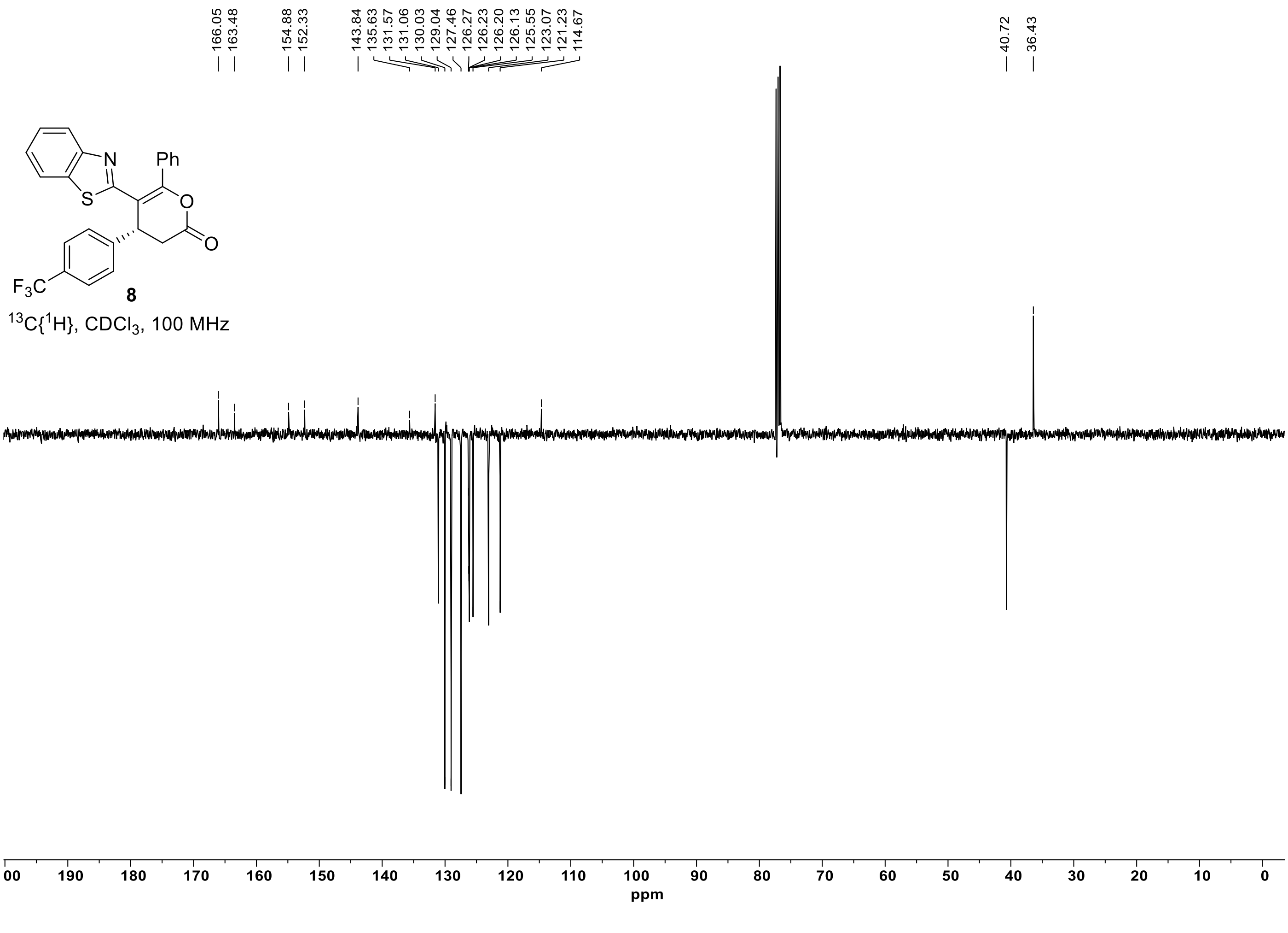

(11*S*)-4-benzoyl-3-(2-chlorophenyl)-2,3-dihydro-1H-benzo[4,5]thiazolo[3,2-a]pyridin-1-one 9

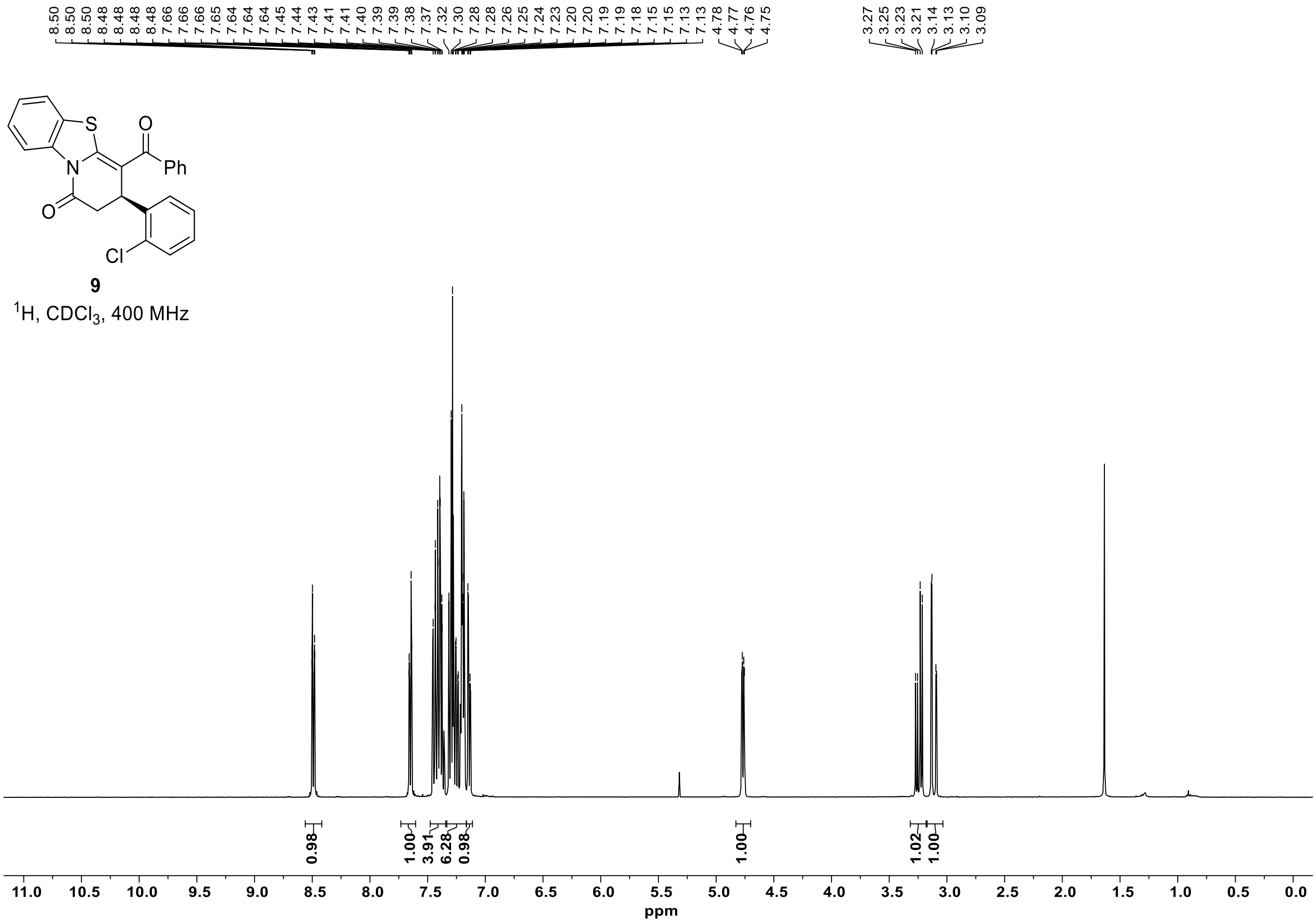

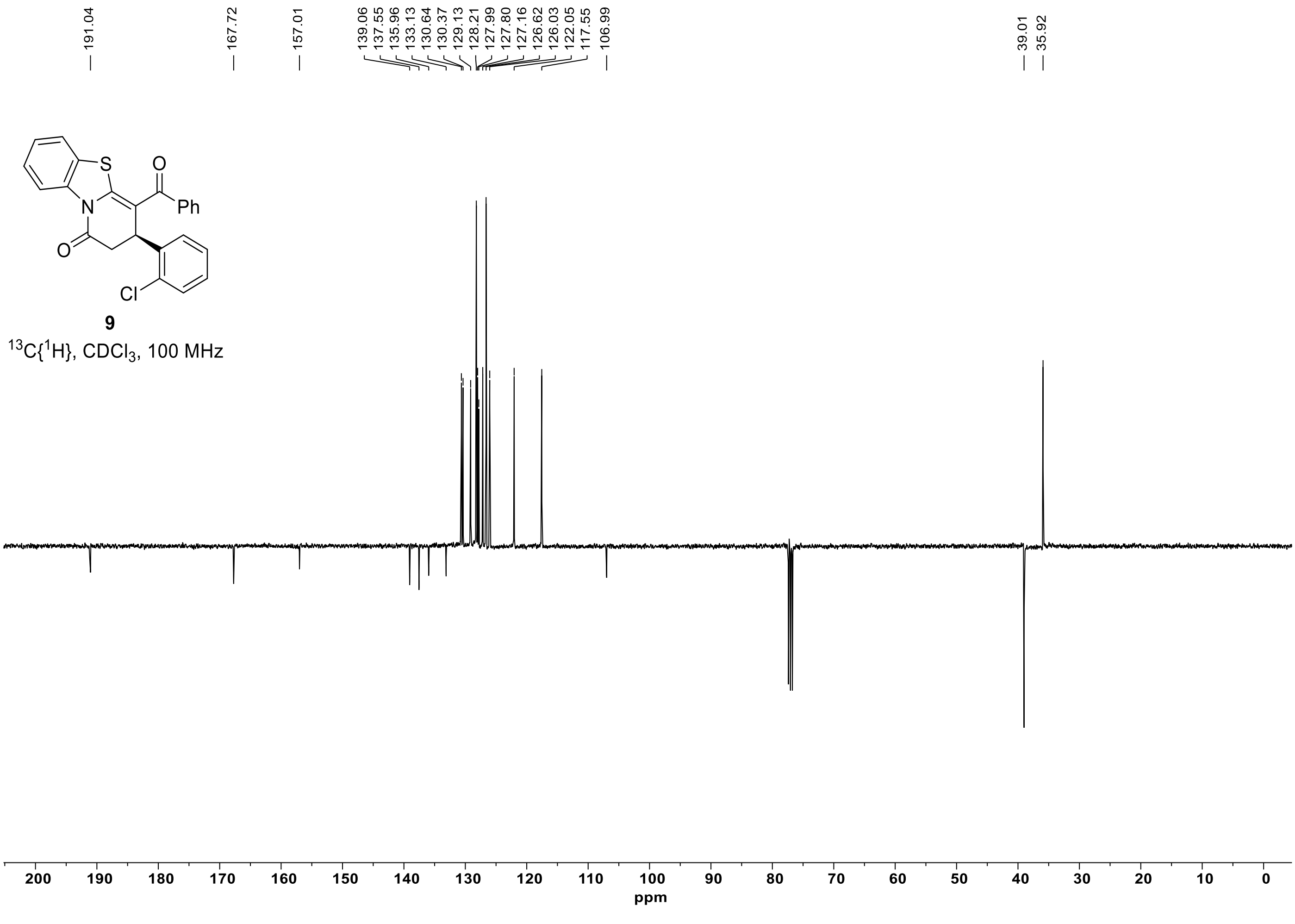

(4*S*)-5-(benzo[d]thiazol-2-yl)-4-(2-chlorophenyl)-6-phenyl-3,4-dihydro-2H-pyran-2-one 10

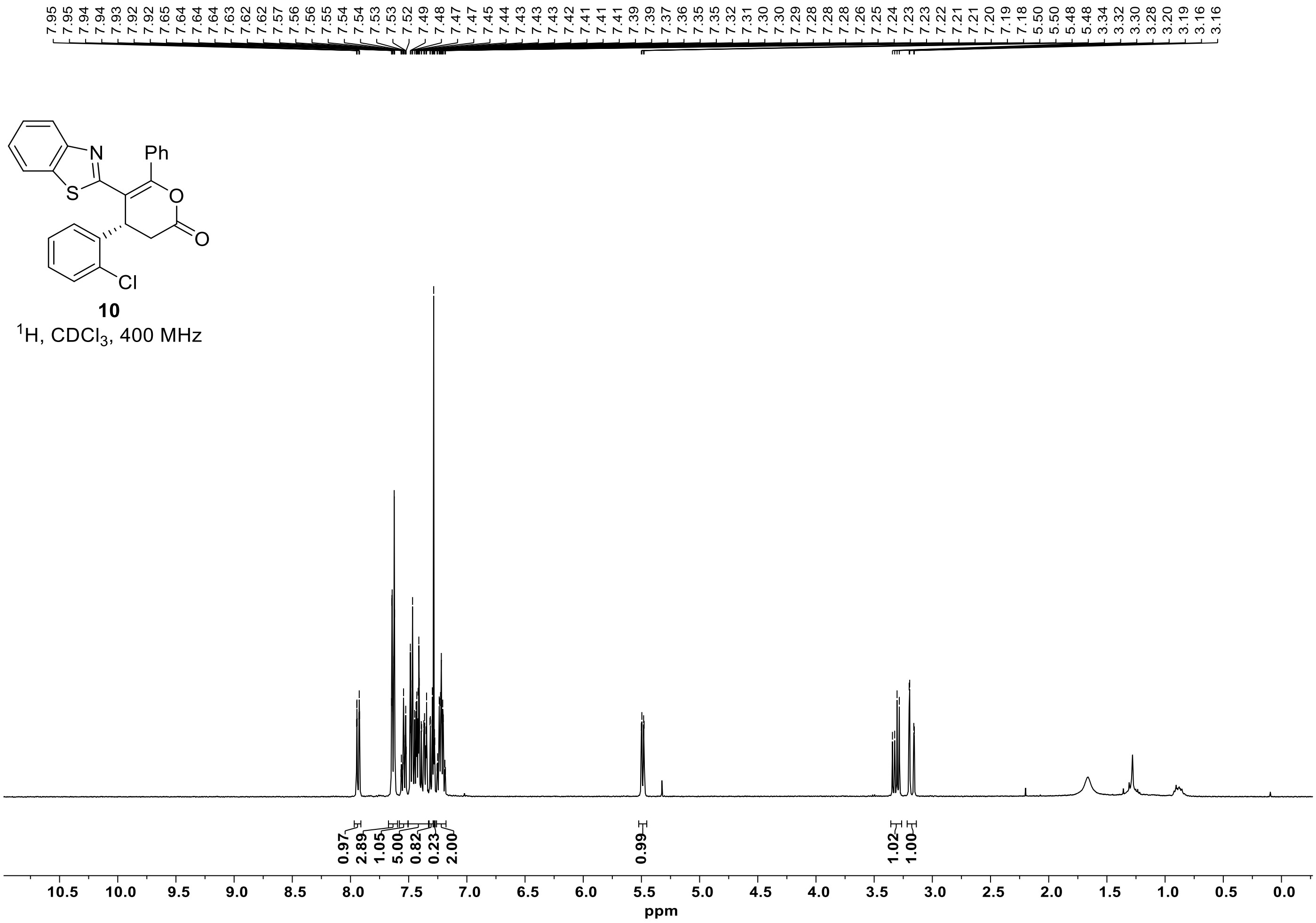

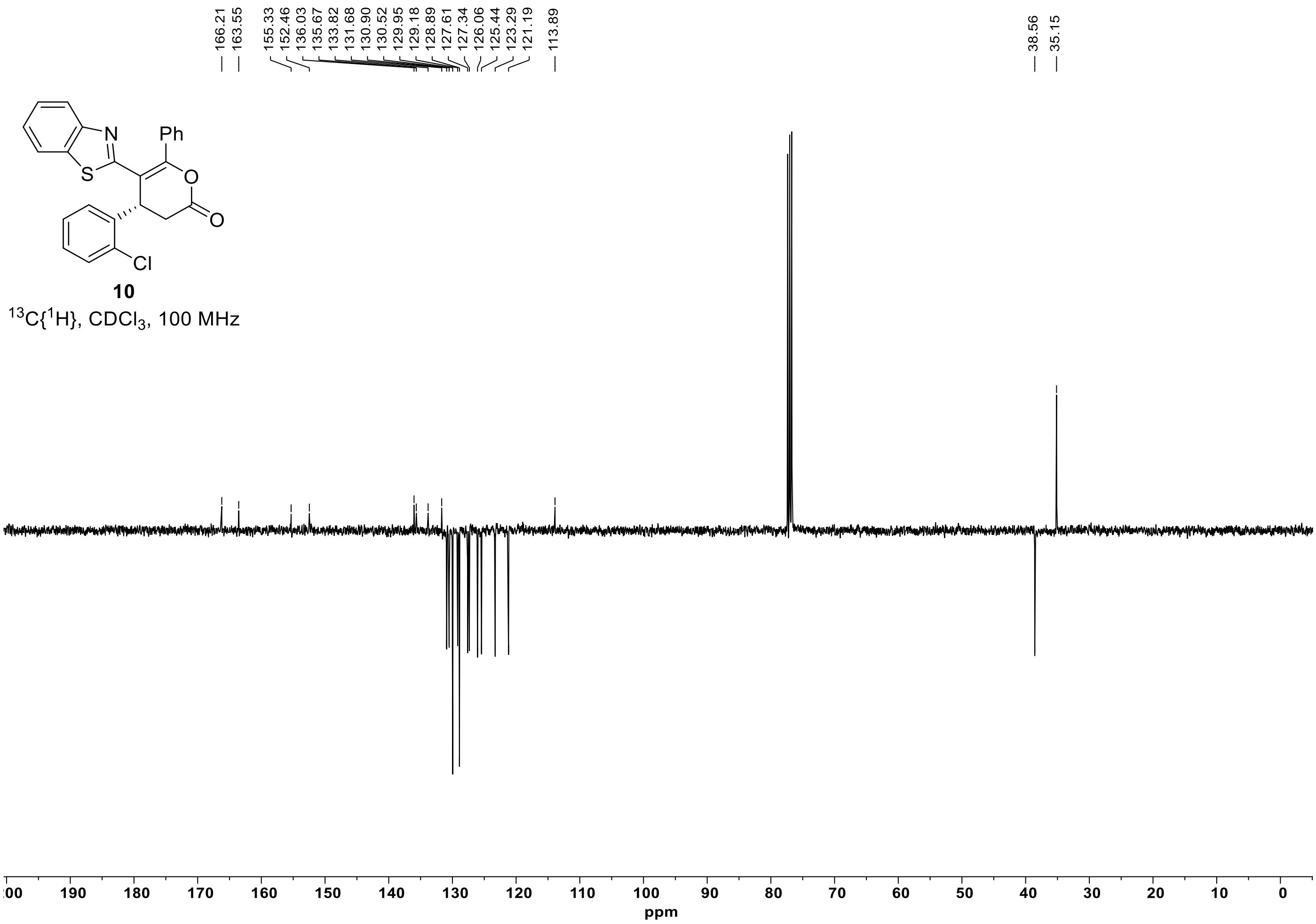

(3*R*)-4-benzoyl-3-(4-methoxyphenyl)-2,3-dihydro-1*H*-benzo[4,5]thiazolo[3,2-*a*]pyridin-1-one 11

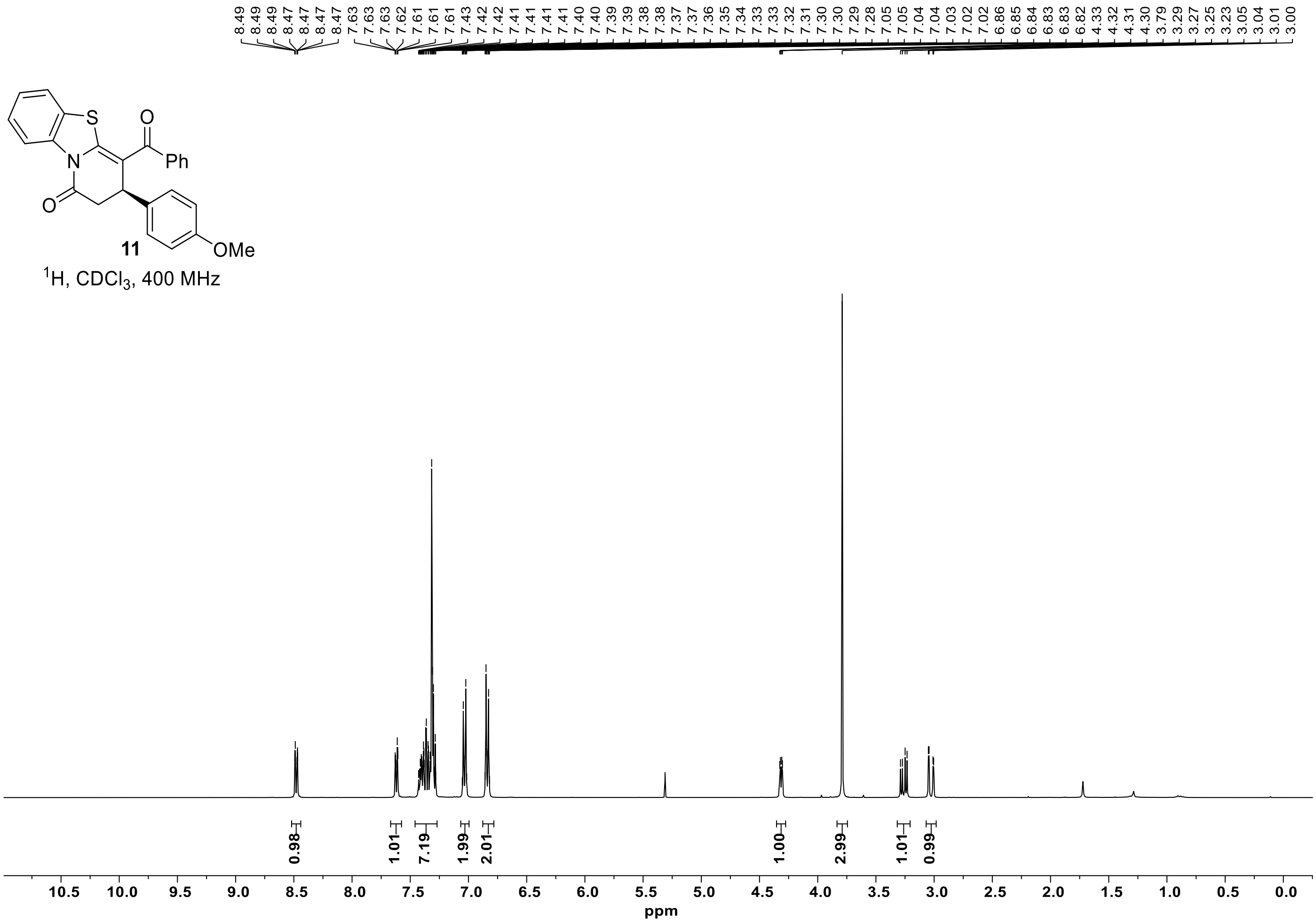

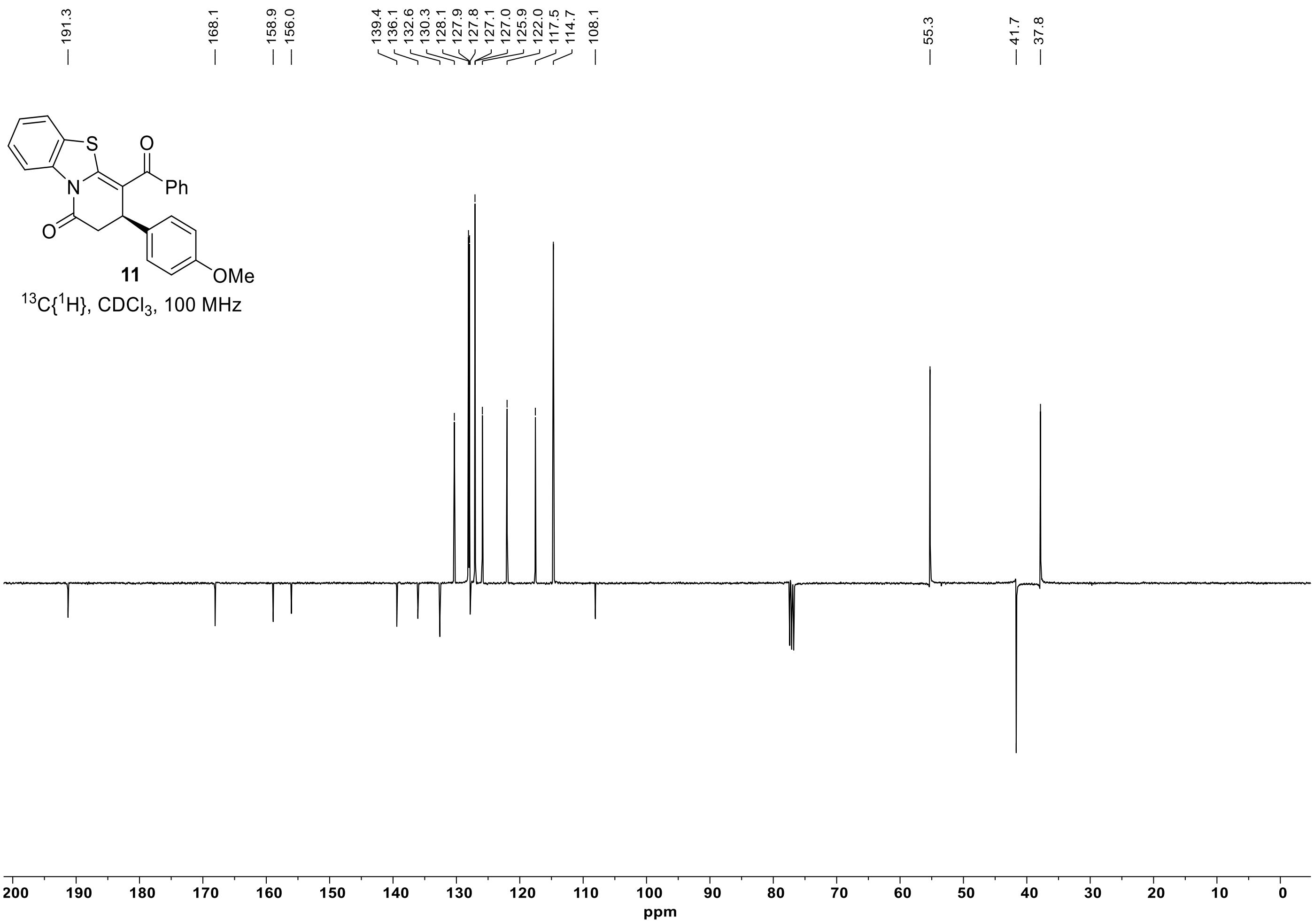

(4*R*)-5-(benzo[d]thiazol-2-yl)-4-(4-methoxyphenyl)-6-phenyl-3,4-dihydro-2H-pyran-2-one 12

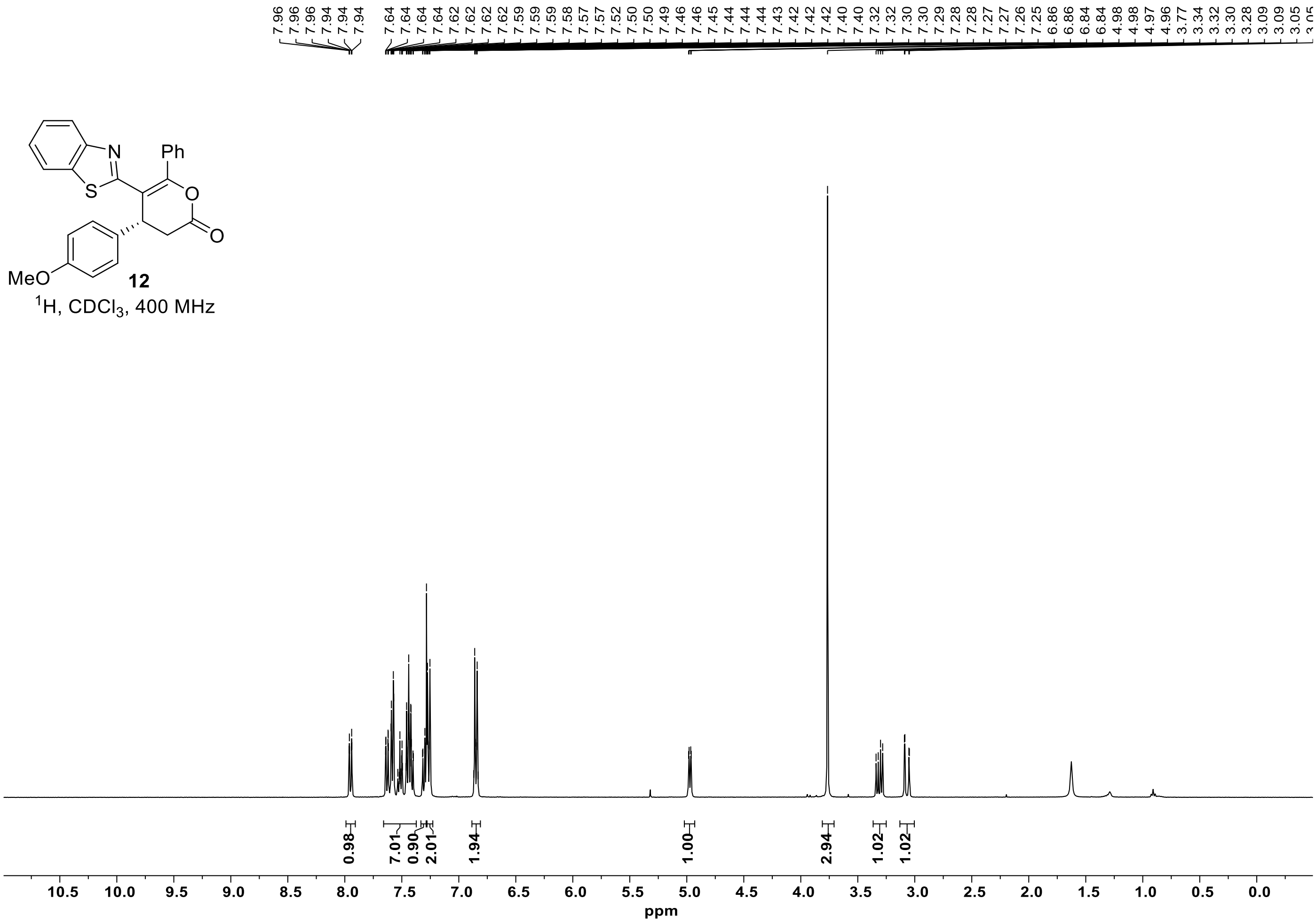

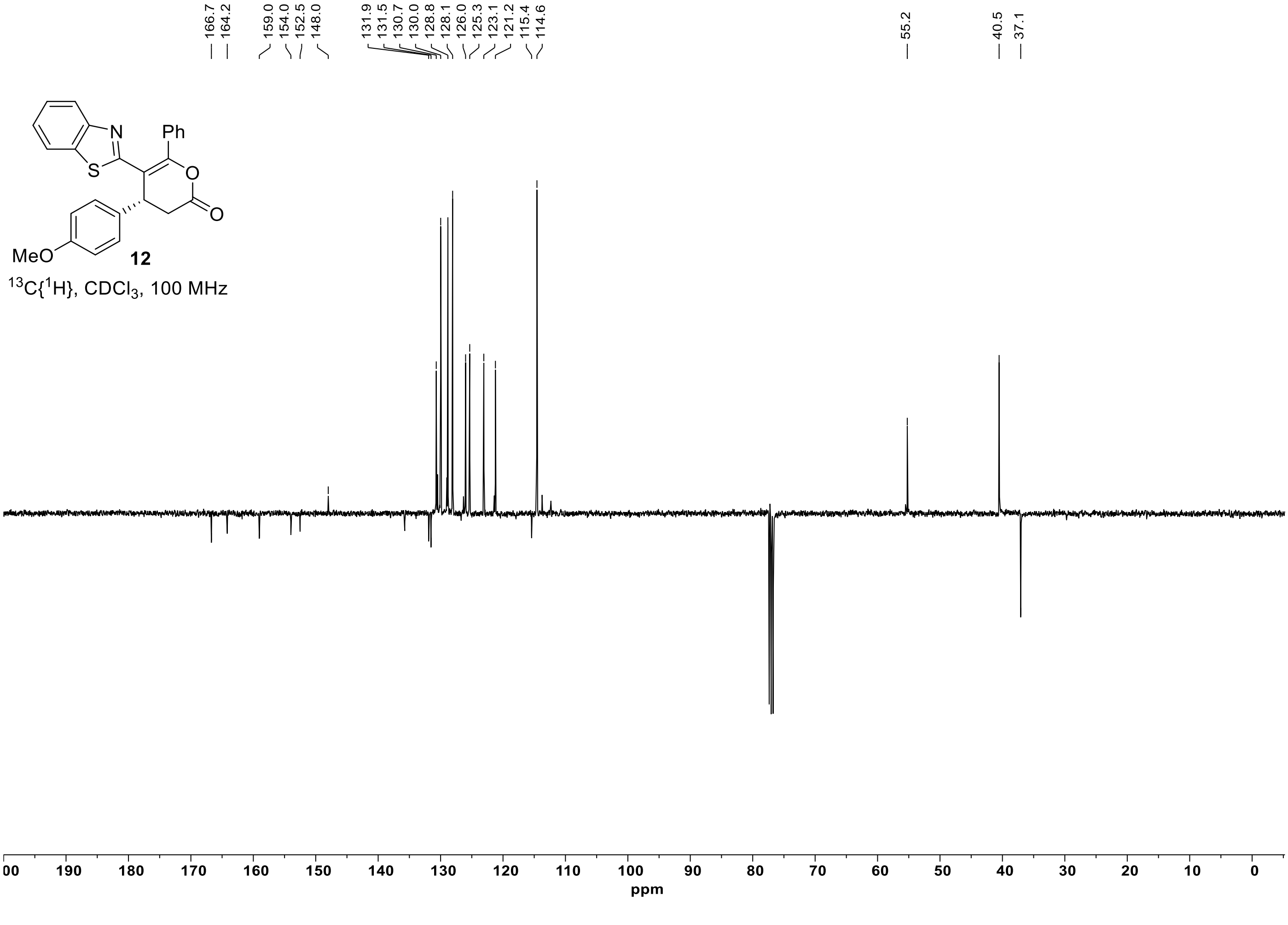

(3*R*)-4-benzoyl-3-(*p*-tolyl)-2,3-dihydro-1*H*-benzo[4,5]thiazolo[3,2-*a*]pyridin-1-one 13

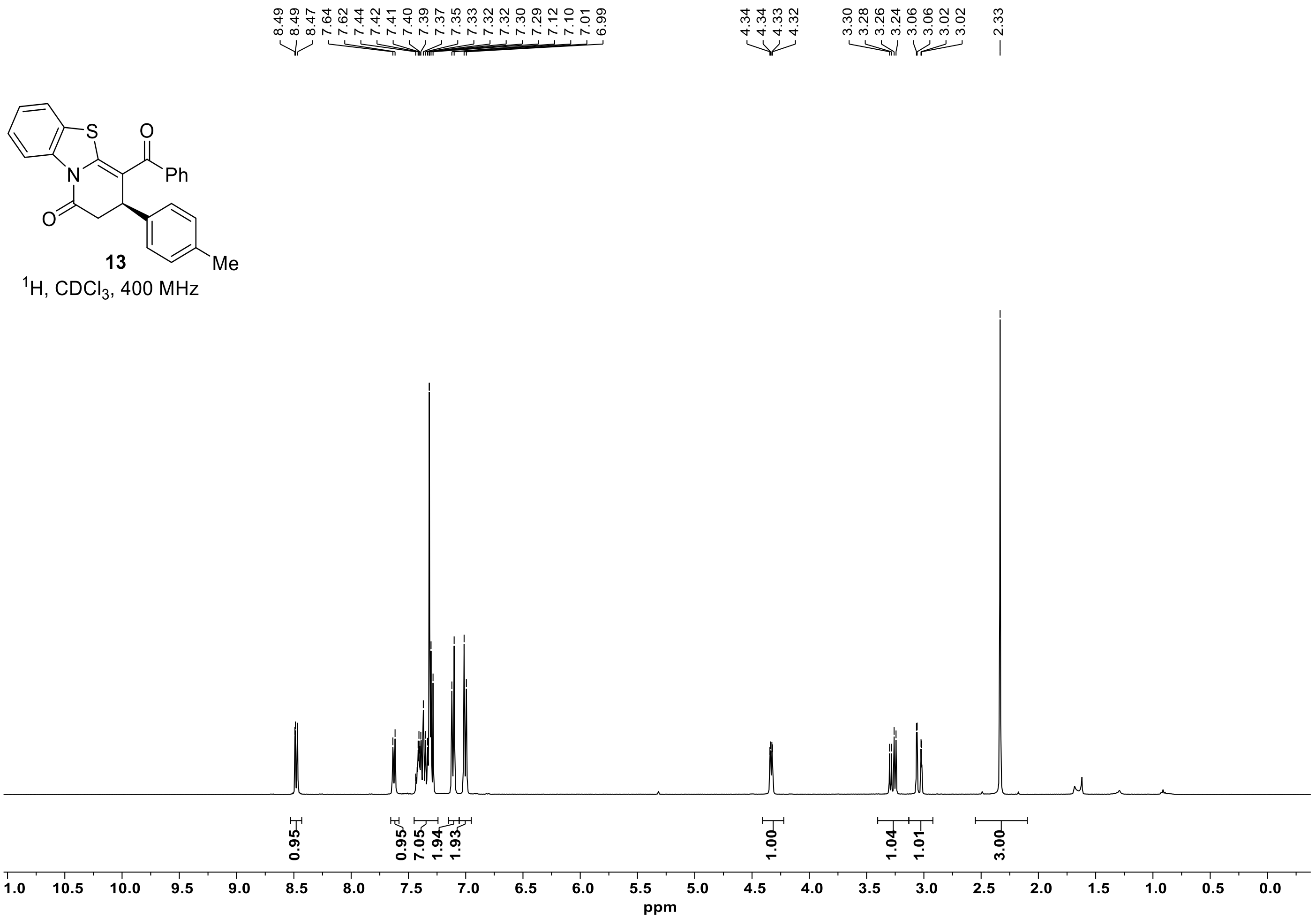

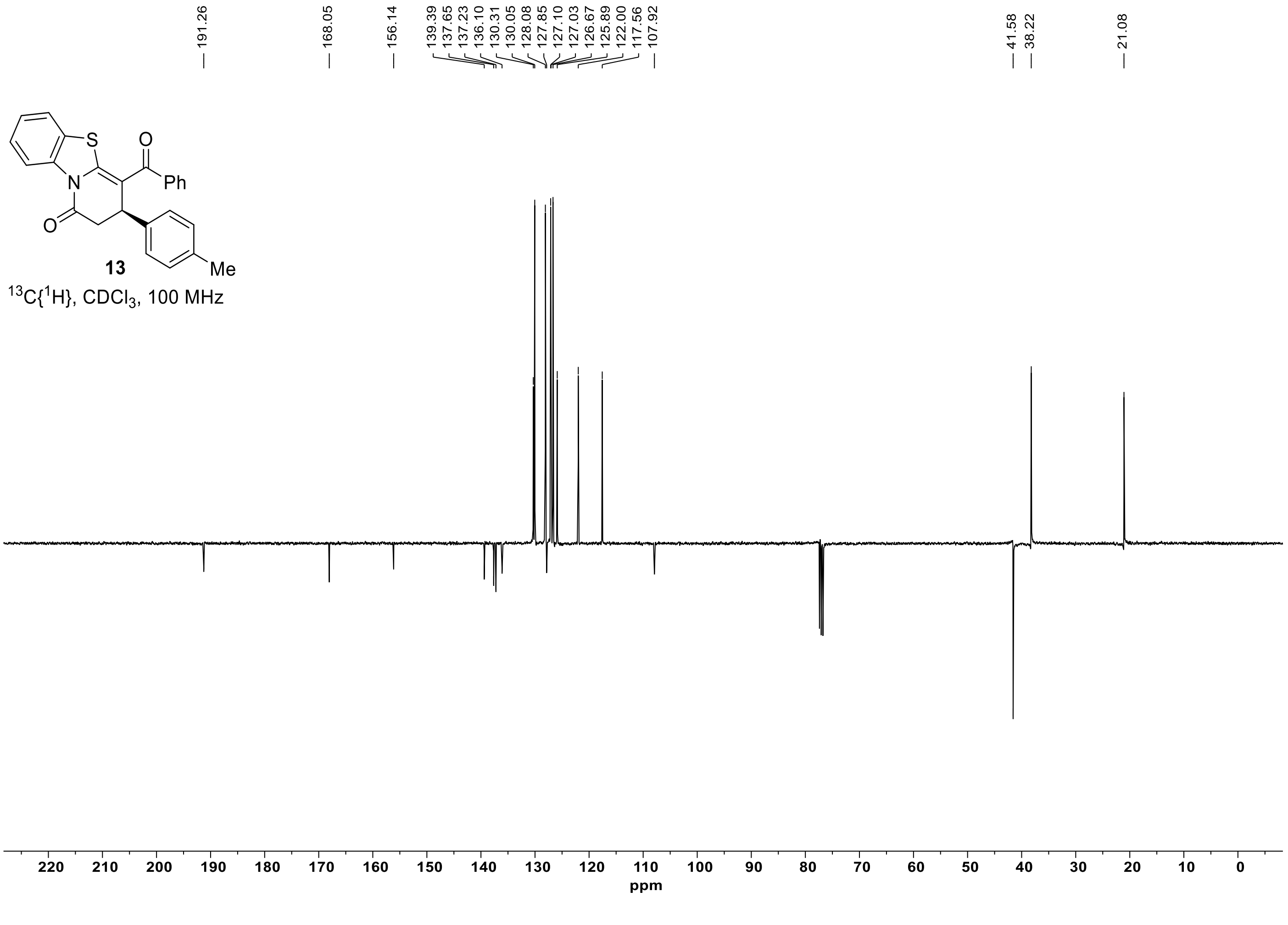

(4*R*)-5-(benzo[d]thiazol-2-yl)-6-phenyl-4-(*p*-tolyl)-3,4-dihydro-2*H*-pyran-2-one 14

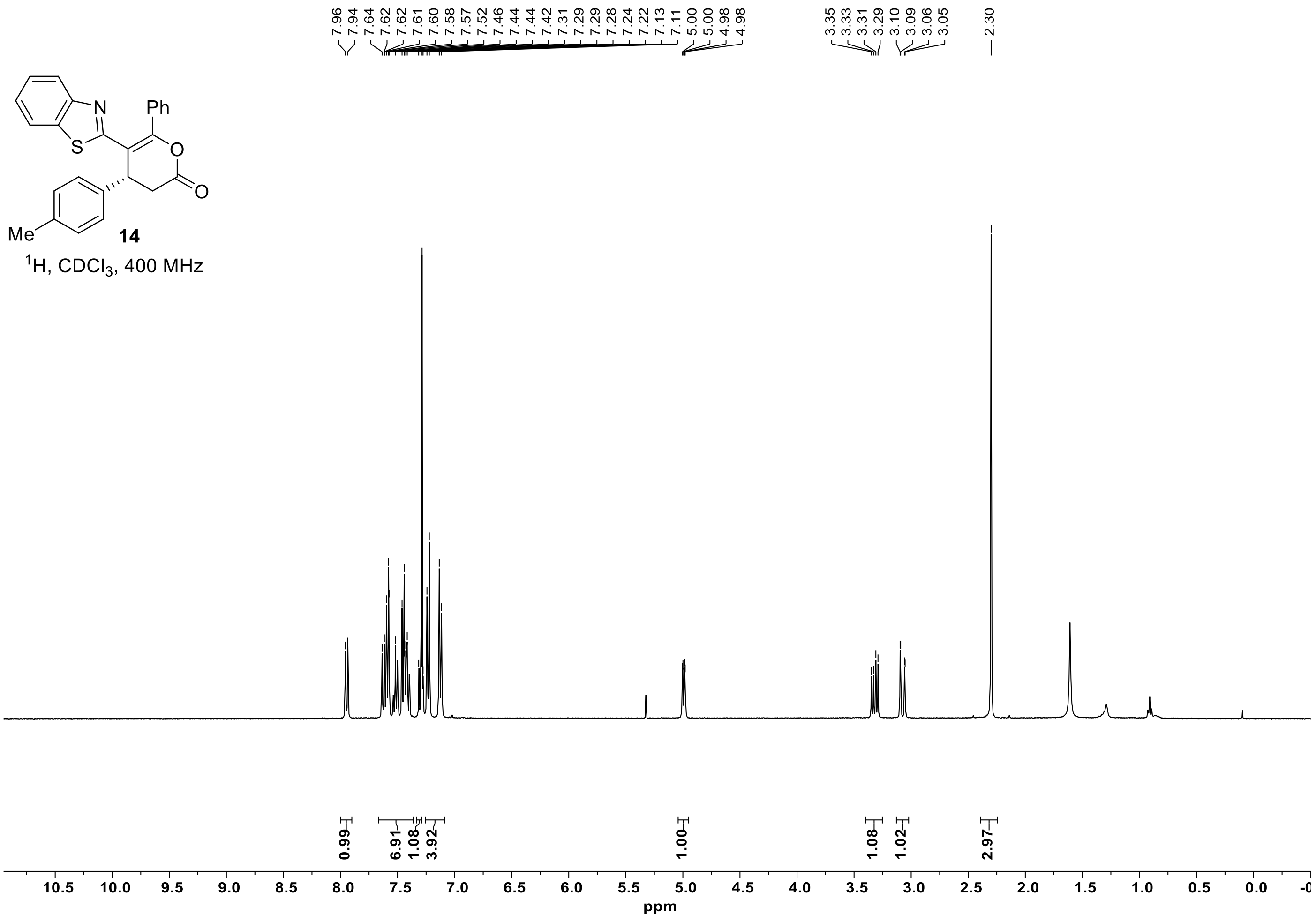

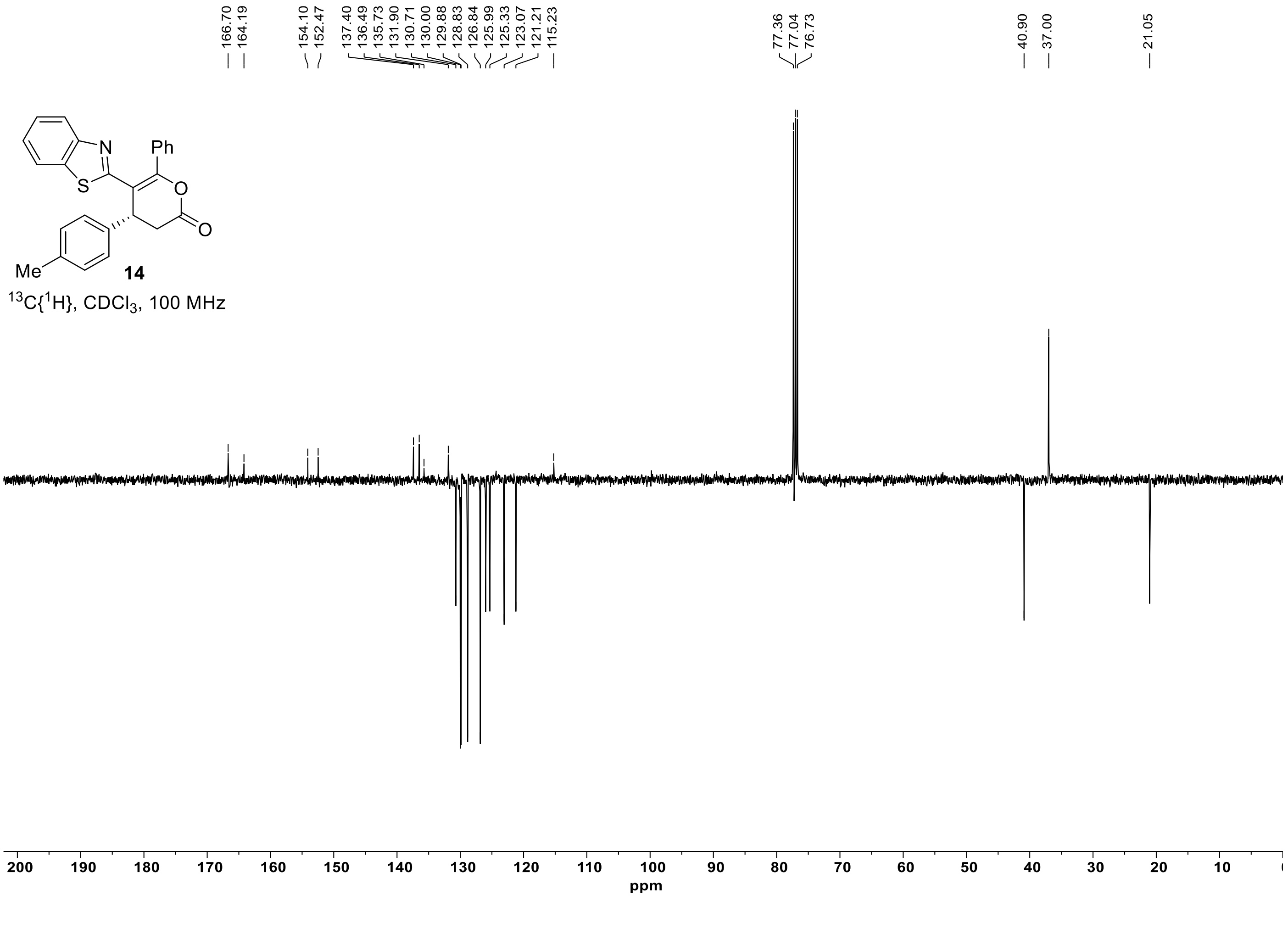

(3*R*)-4-benzoyl-3-(*m*-tolyl)-2,3-dihydro-1*H*-benzo[4,5]thiazolo[3,2-*a*]pyridin-1-one 15

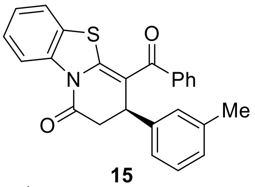

15
^1H, $CDCl_3$, 400 MHz

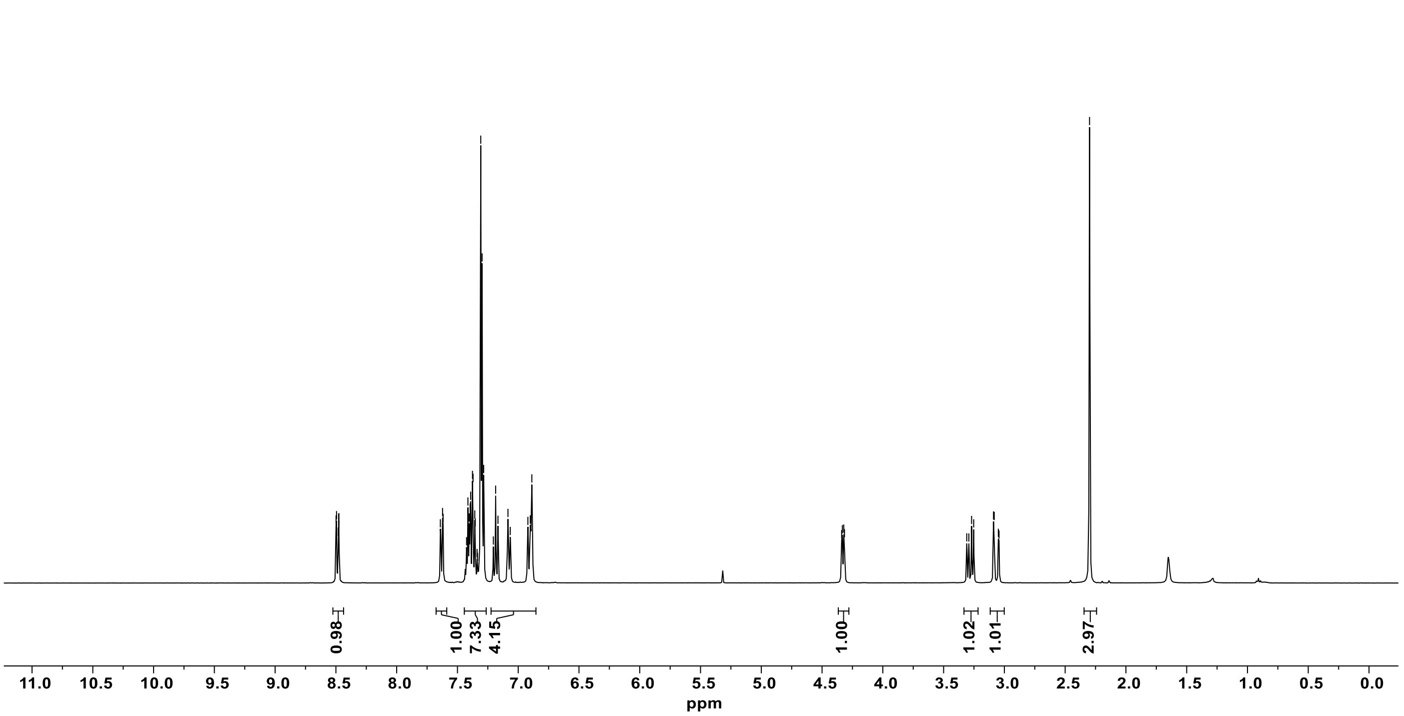

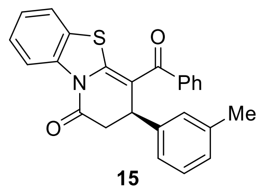

15
$^{13}C\{^1H\}$, $CDCl_3$, 100 MHz

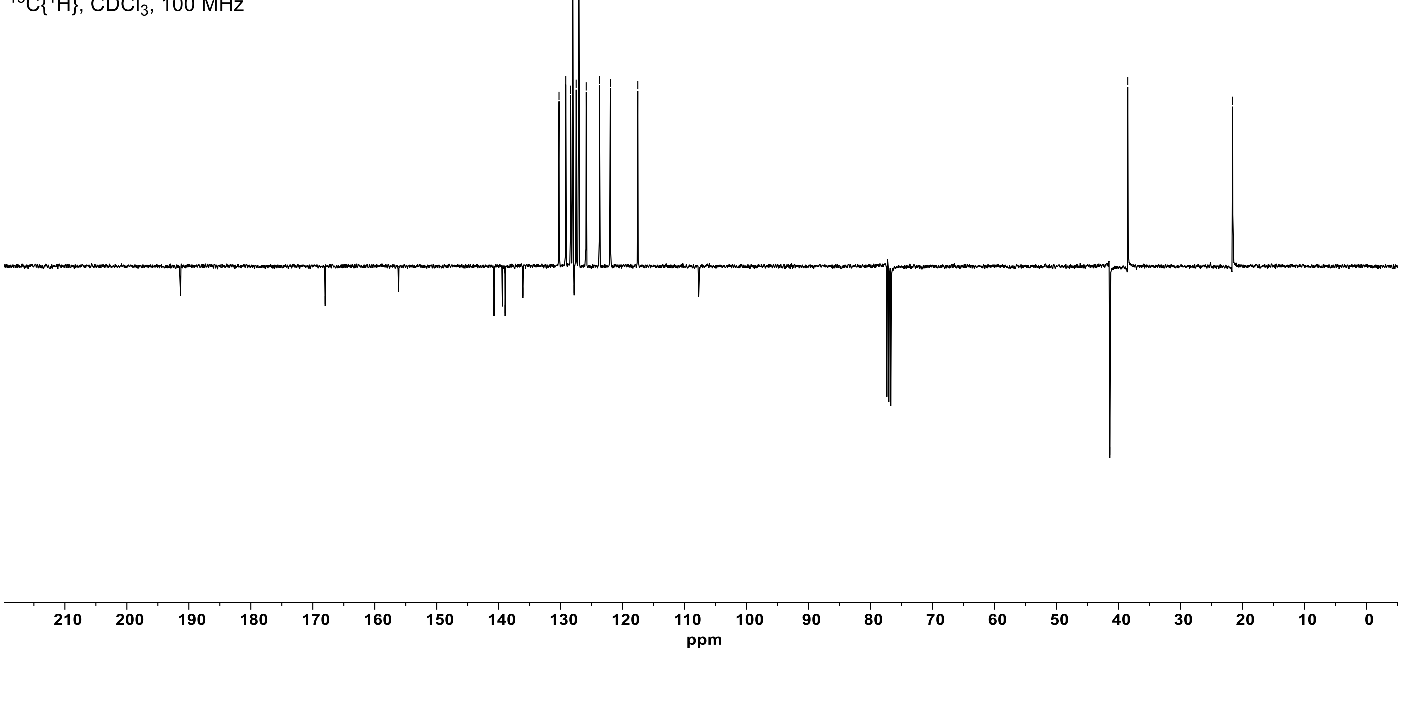

(4*R*)-5-(benzo[d]thiazol-2-yl)-6-phenyl-4-(*m*-tolyl)-3,4-dihydro-2*H*-pyran-2-one 16

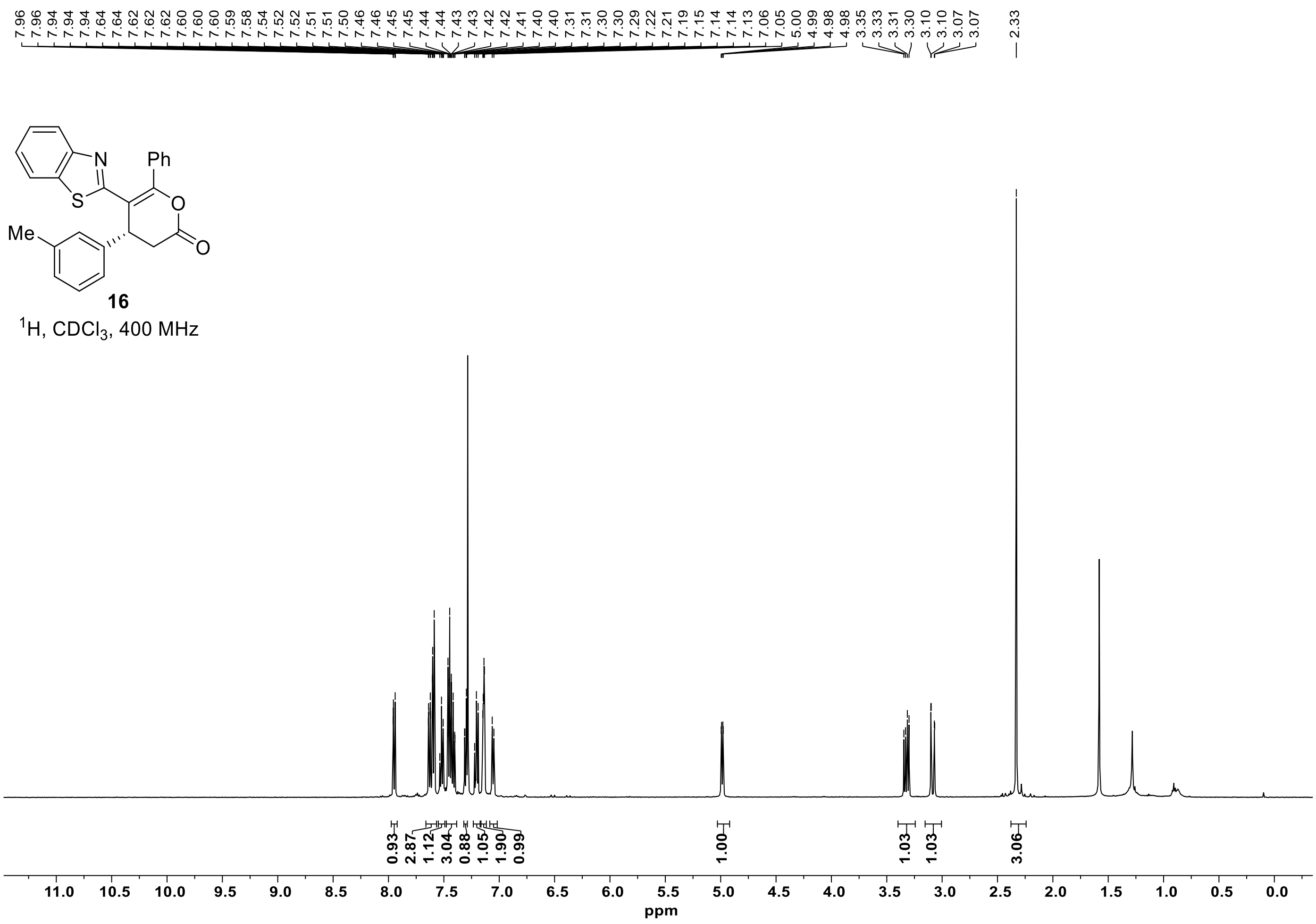

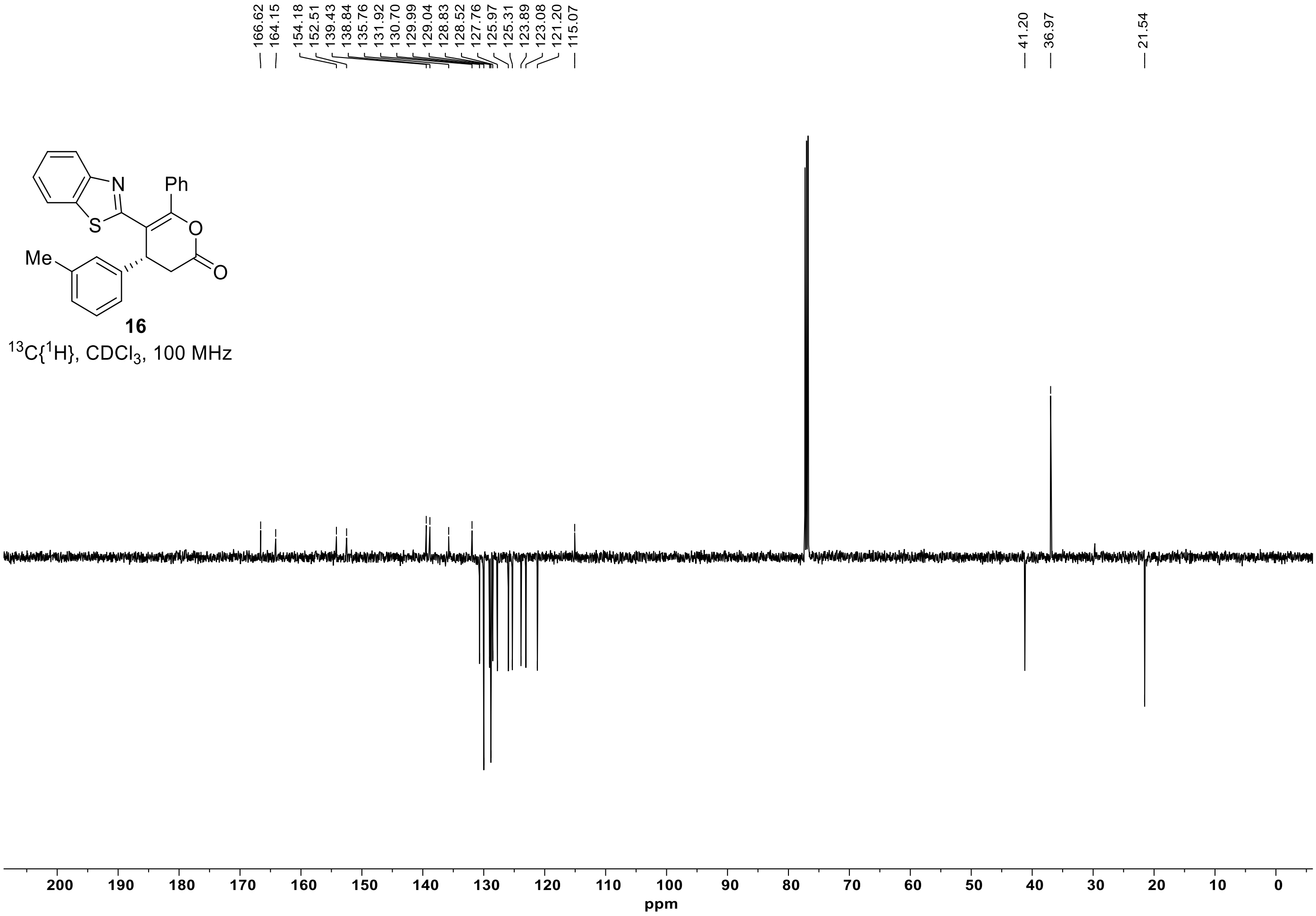

(3*R*)-4-benzoyl-3-(naphth-1-yl)-2,3-dihydro-1*H*-benzo[4,5]thiazolo[3,2-*a*]pyridin-1-one 17

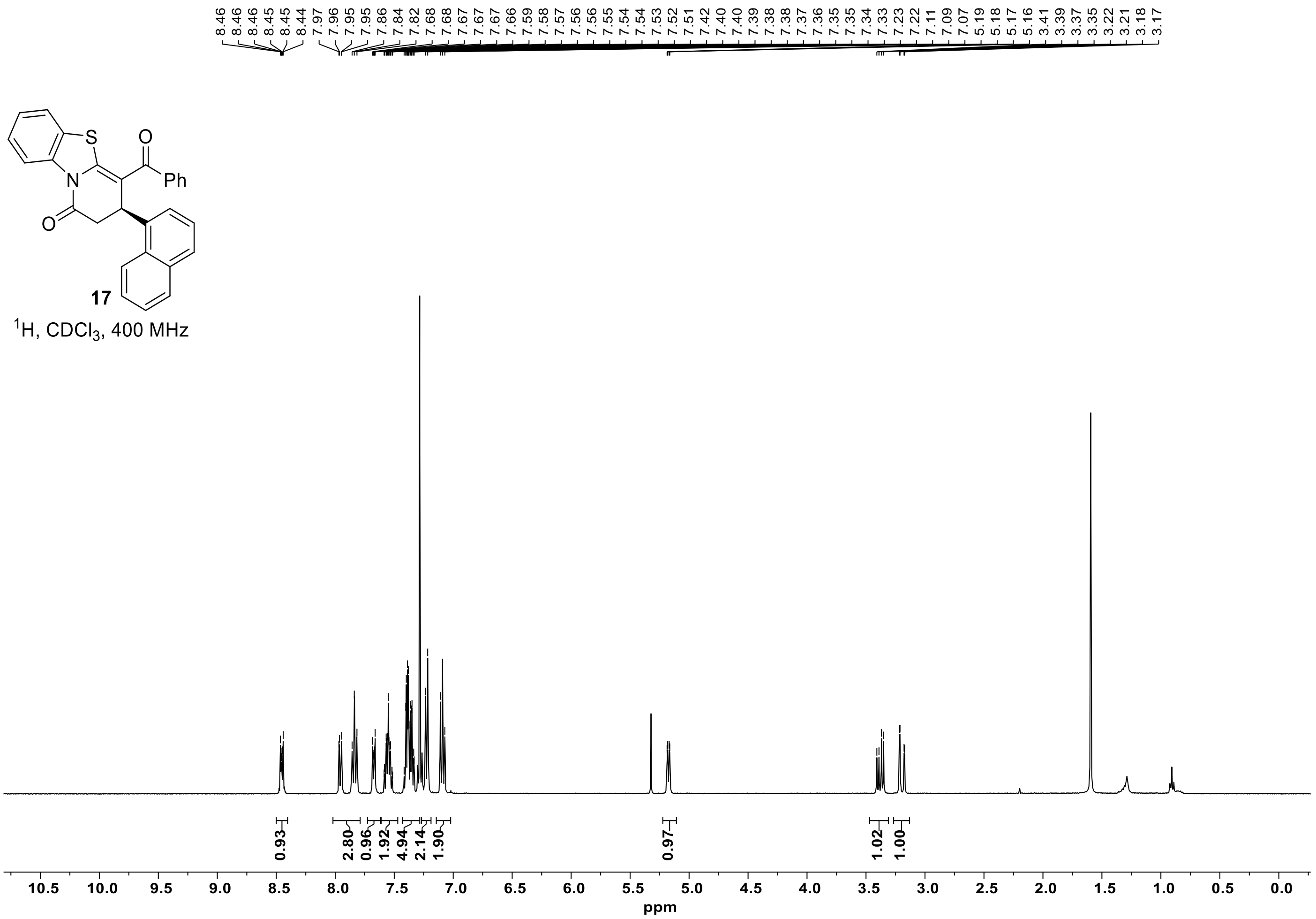

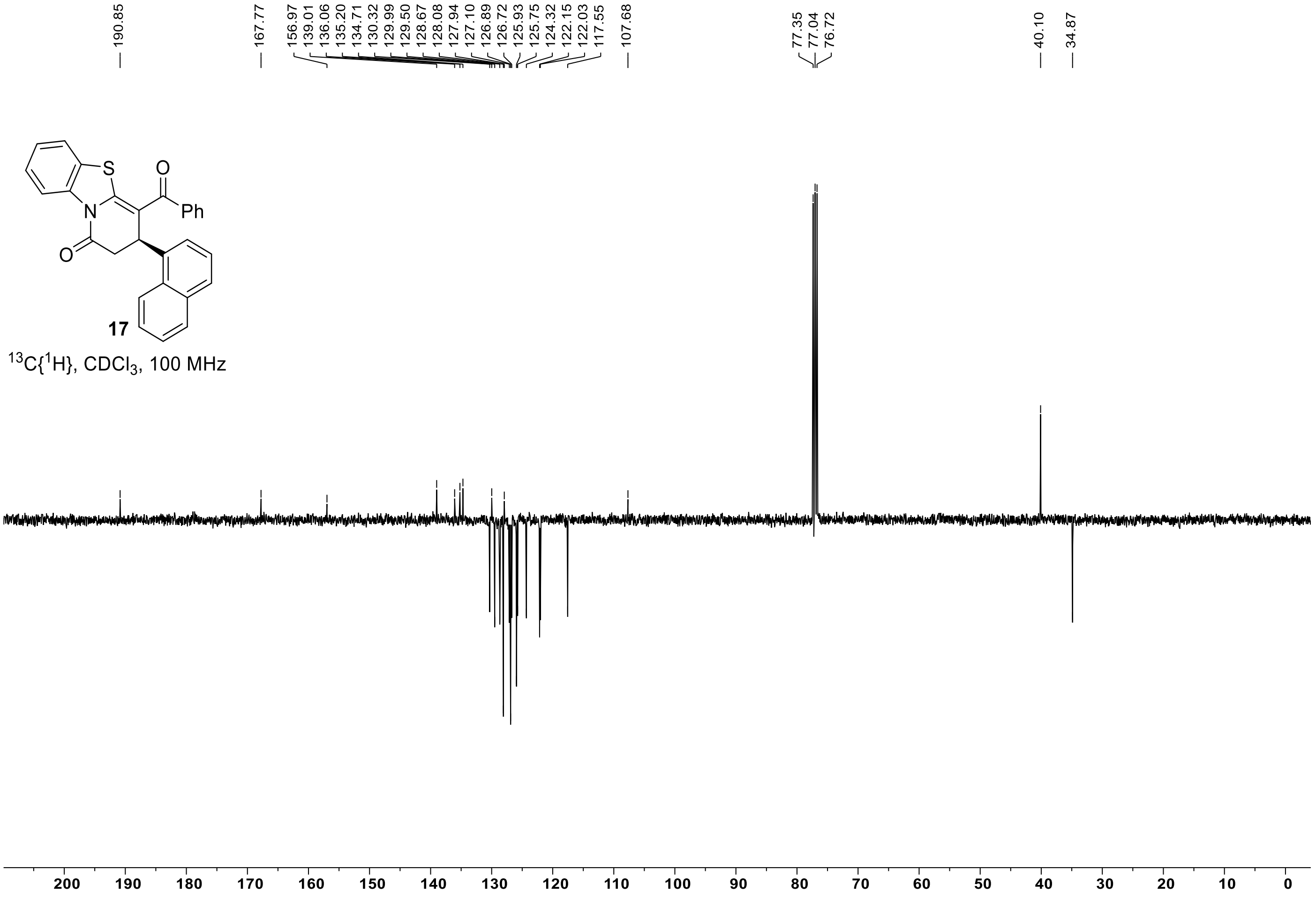

(4*R*)-5-(benzo[d]thiazol-2-yl)-4-(naphth-1-yl)-6-phenyl-3,4-dihydro-2*H*-pyran-2-one 18

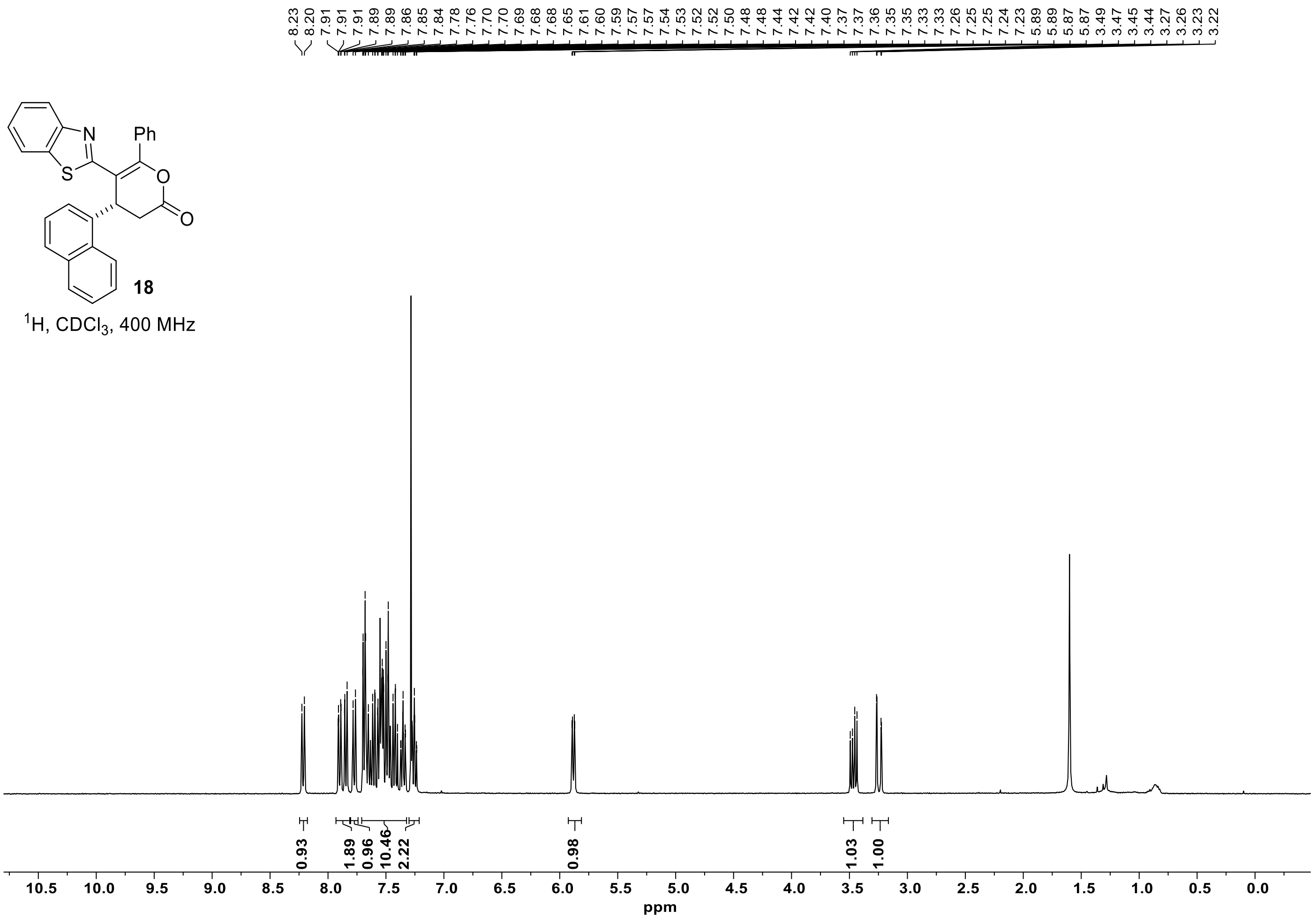

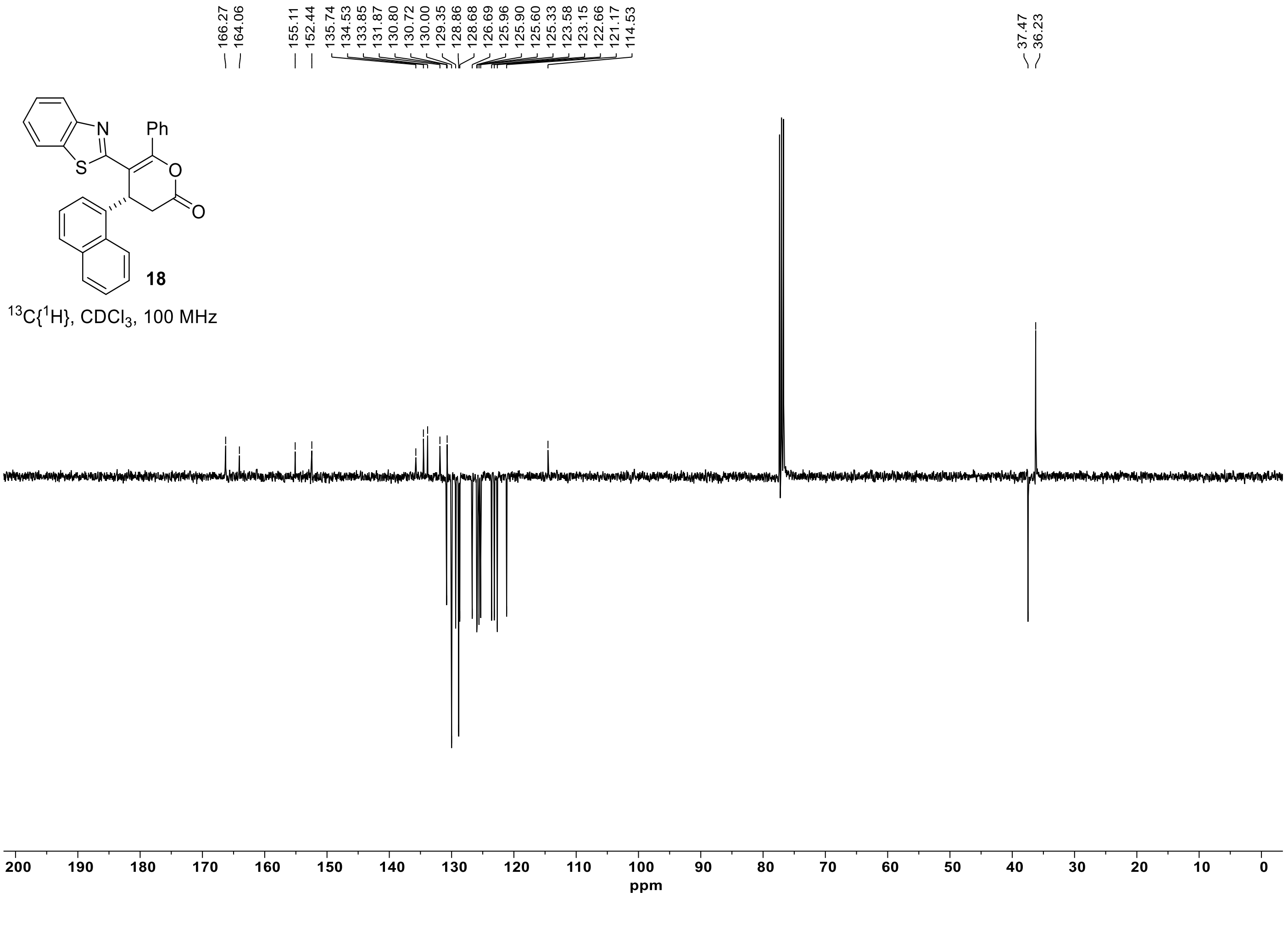

(3*S*)-4-benzoyl-3-(furan-2-yl)-2,3-dihydro-1*H*-benzo[4,5]thiazolo[3,2-*a*]pyridin-1-one 19

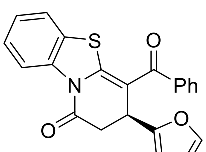

19

^{1}H, $CDCl_3$, 400 MHz

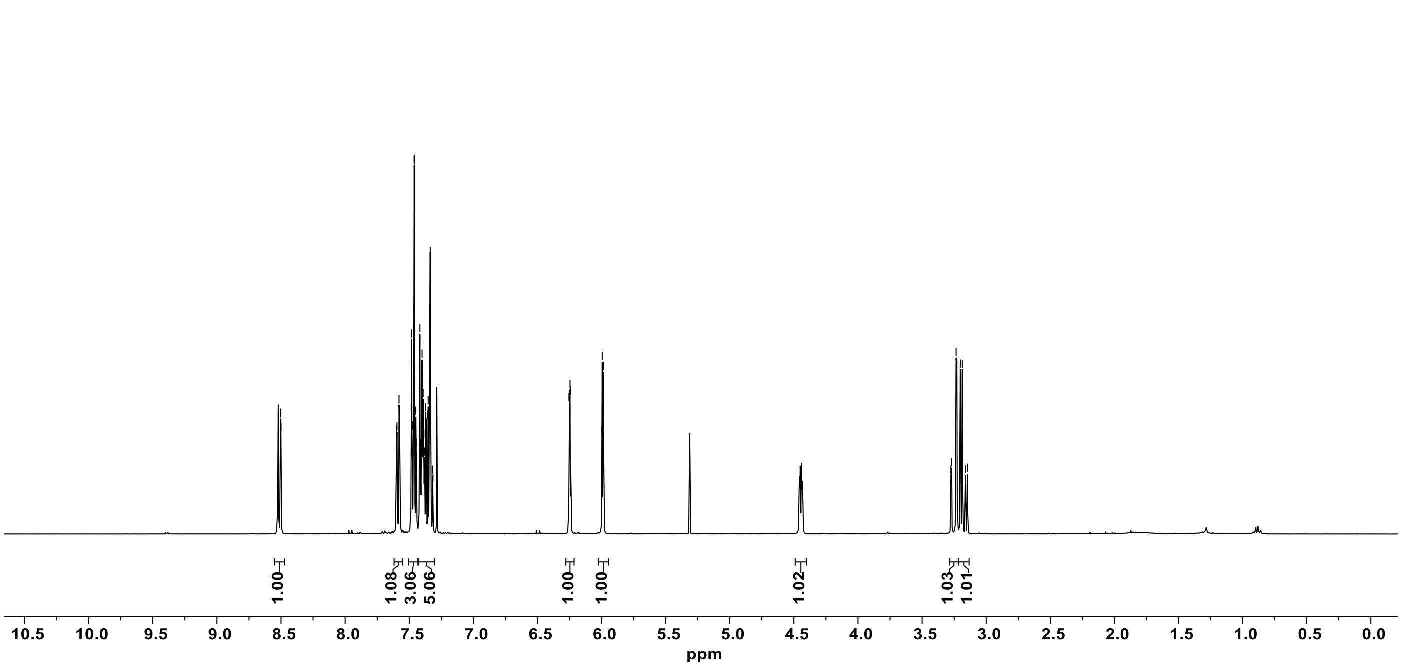

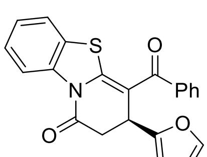

19

$^{13}C\{^1H\}$, $CDCl_3$, 100 MHz

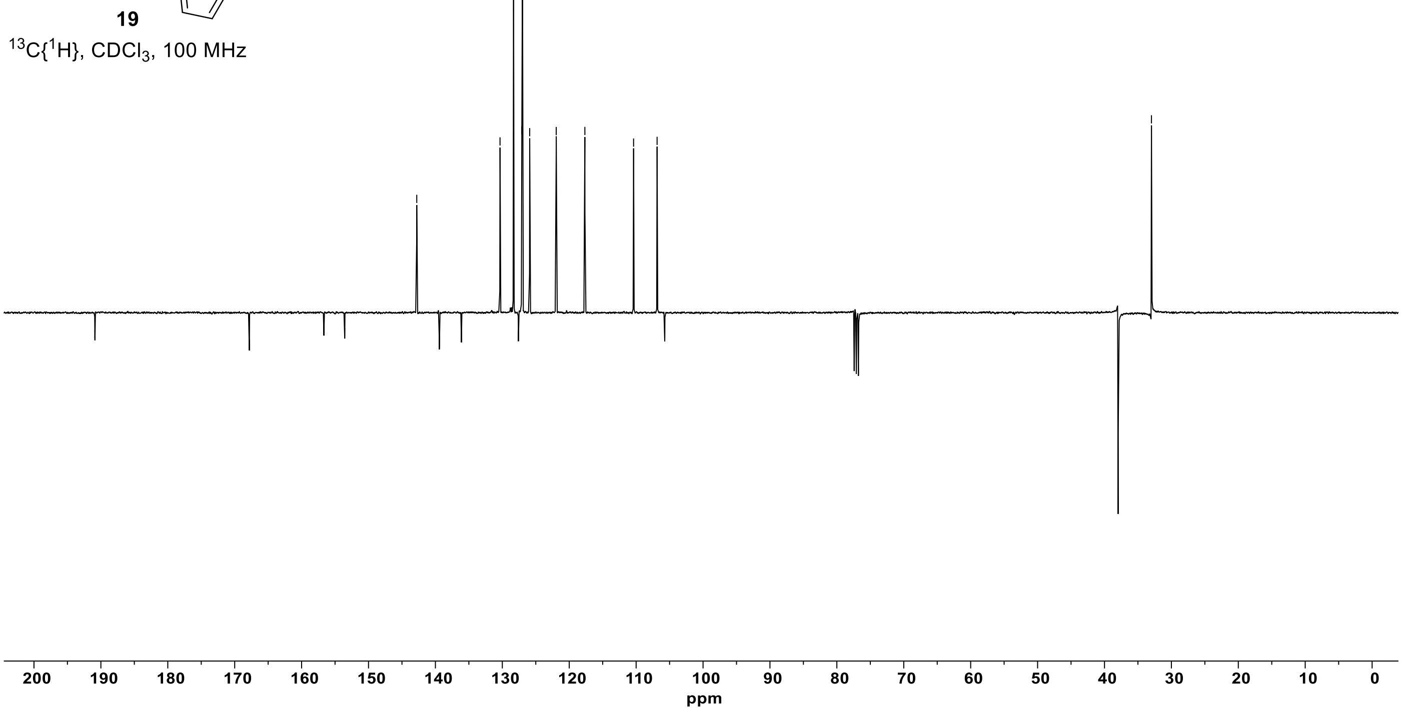

(4*S*)-5-(benzo[d]thiazol-2-yl)-4-(furan-2-yl)-6-phenyl-3,4-dihydro-2*H*-pyran-2-one 20

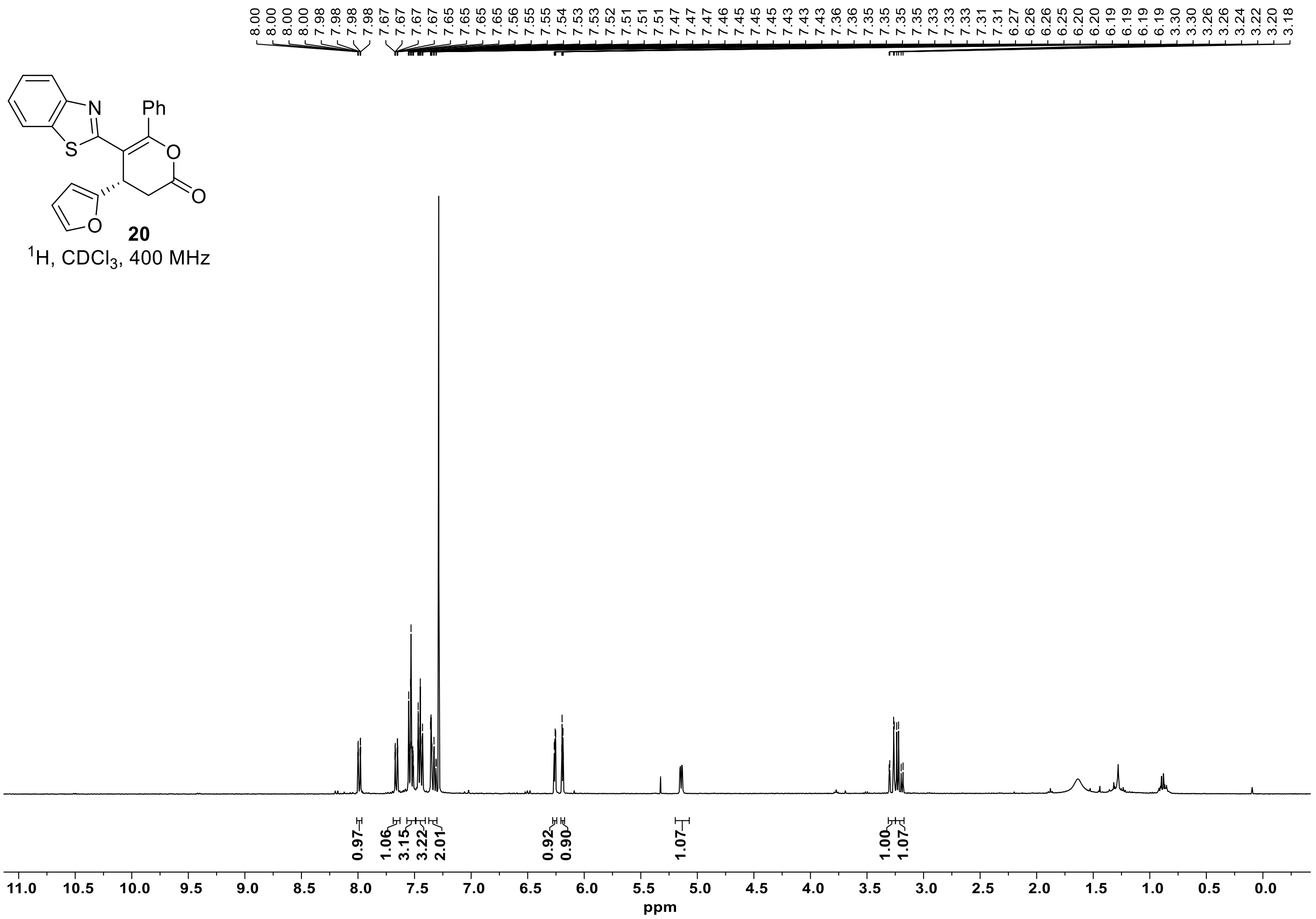

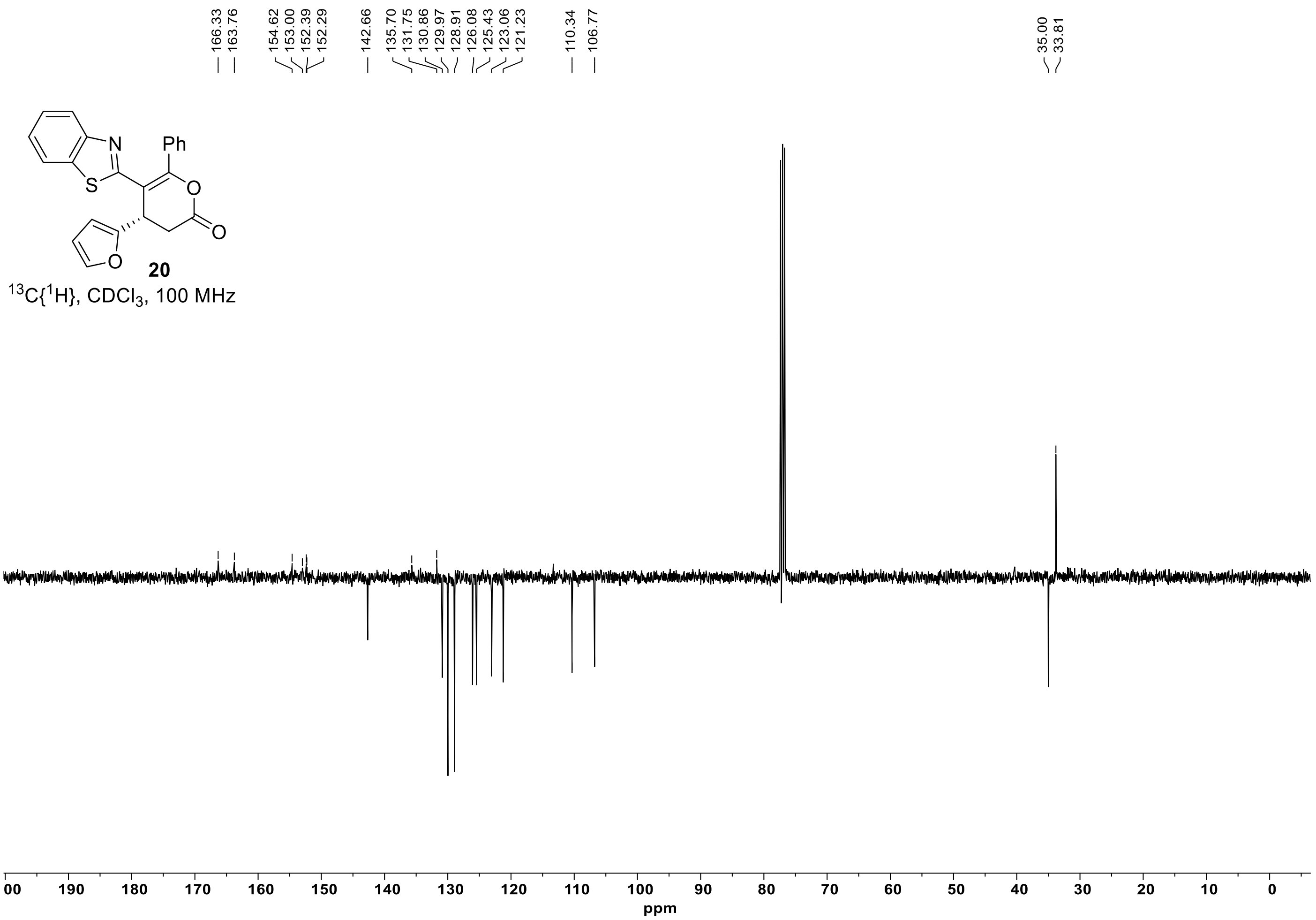

(3*S*)-4-benzoyl-3-(thien-2-yl)-2,3-dihydro-1*H*-benzo[4,5]thiazolo[3,2-*a*]pyridin-1-one 21

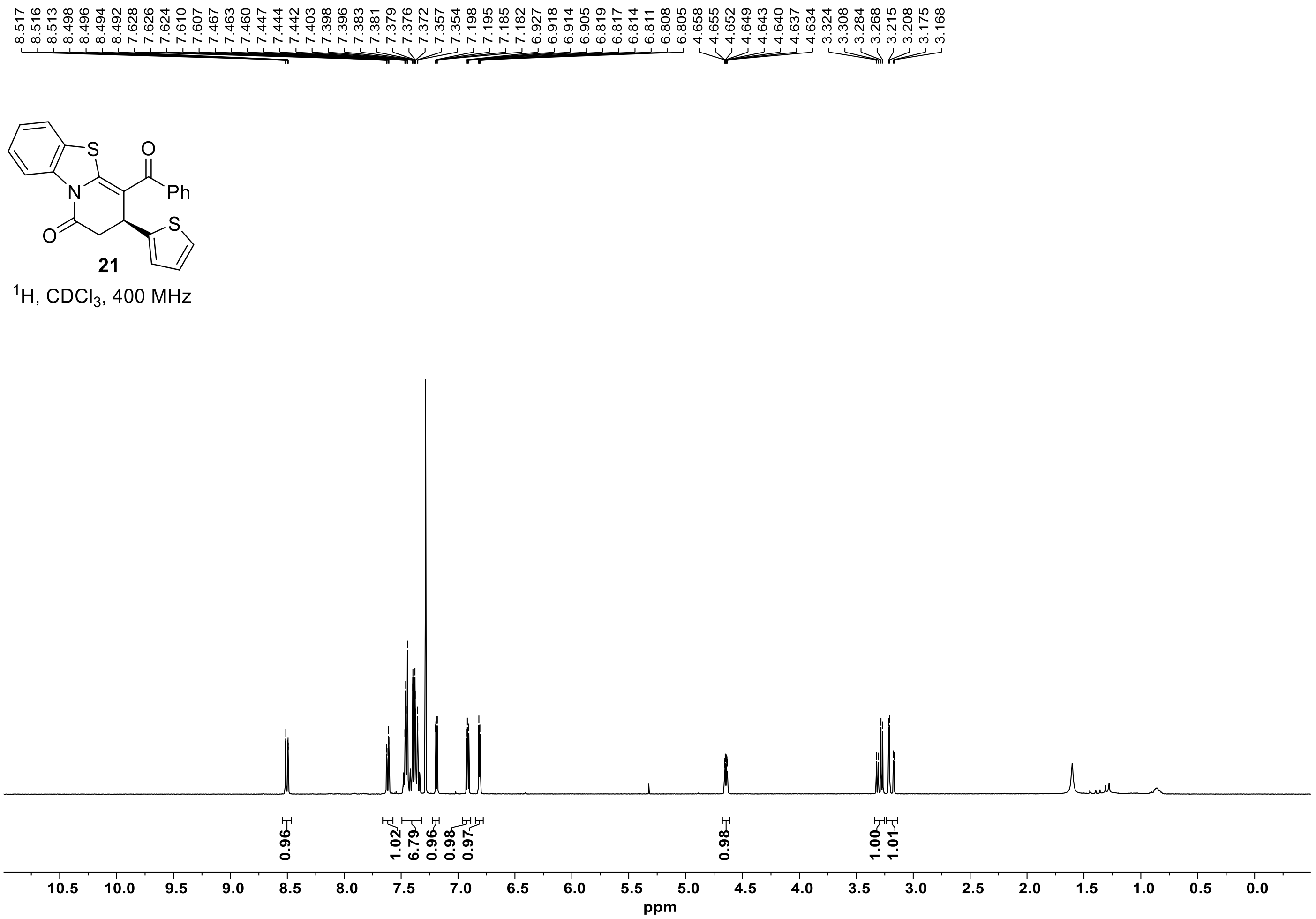

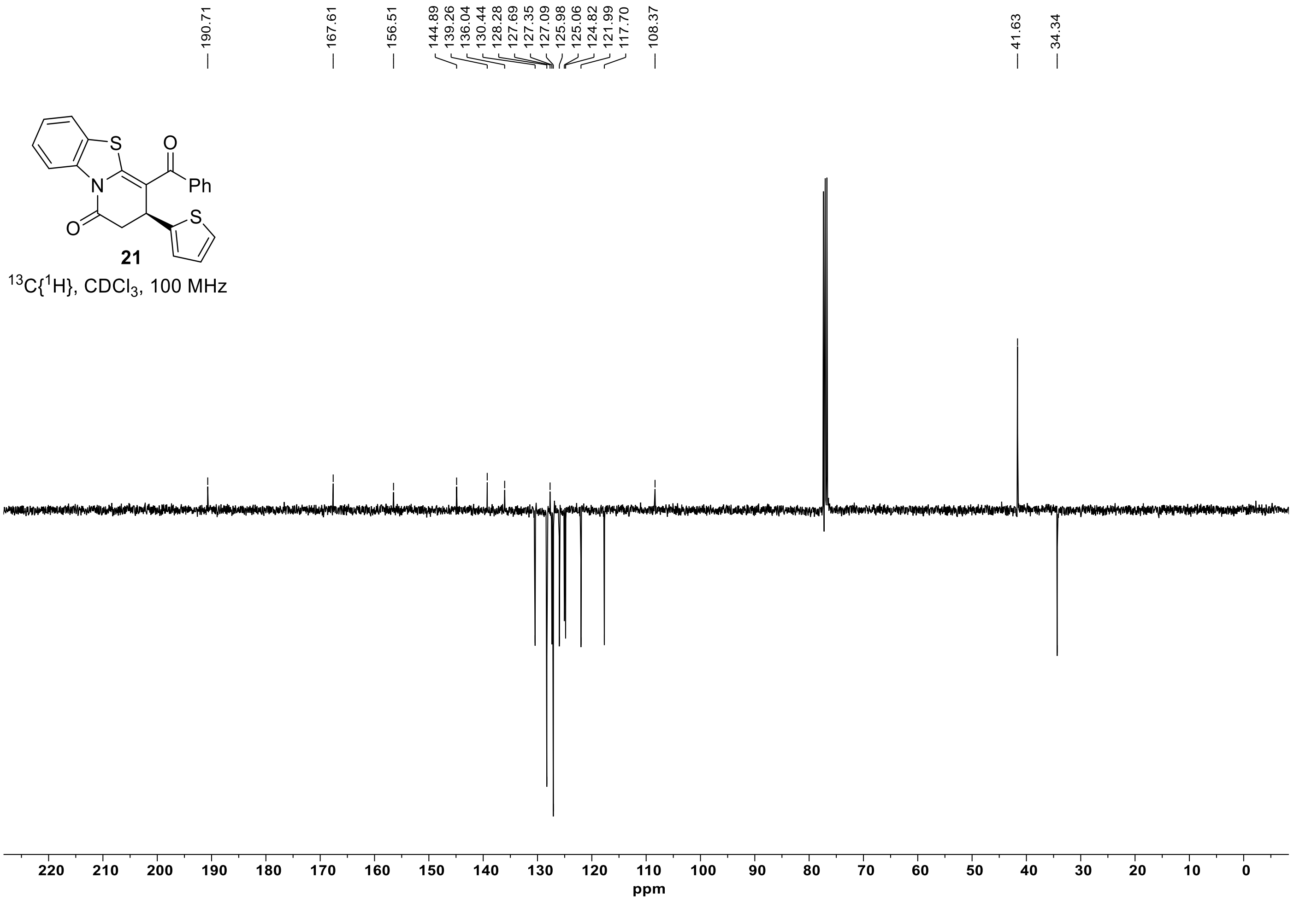

(4*S*)-5-(benzo[d]thiazol-2-yl)-6-phenyl-4-(thien-2-yl)-3,4-dihydro-2*H*-pyran-2-one 22

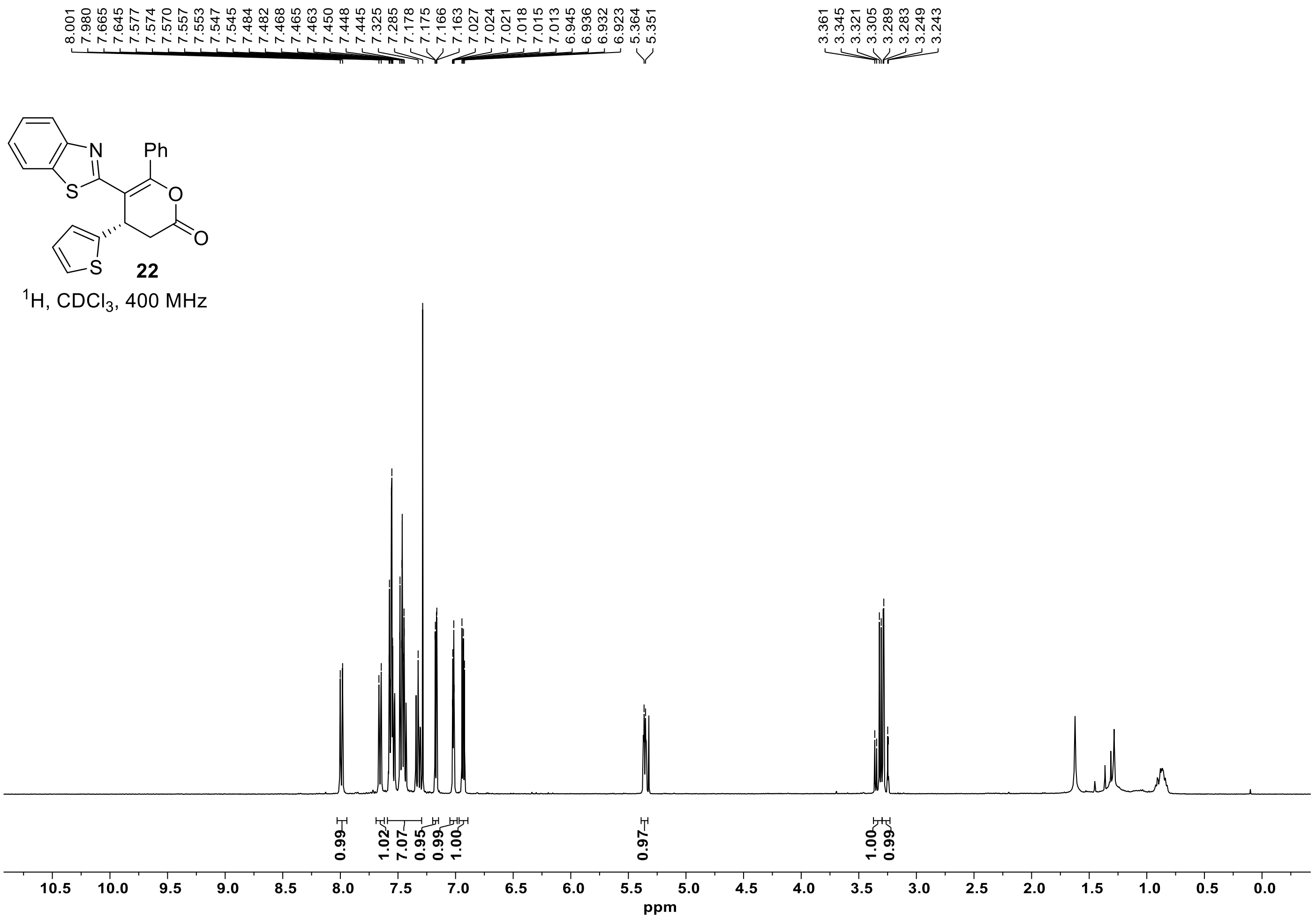

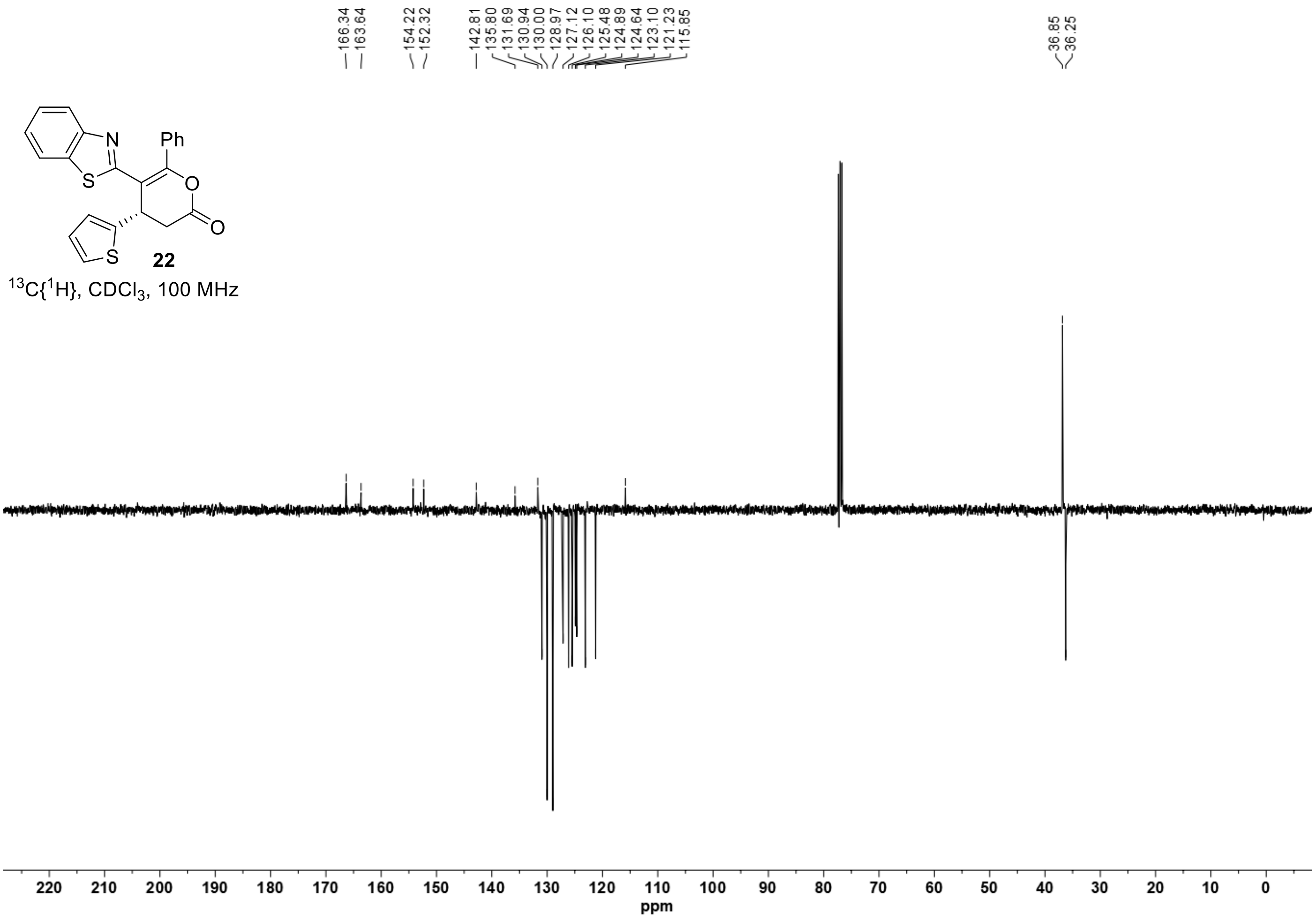

(3*S*)-4-benzoyl-3-methyl-2,3-dihydro-1*H*-benzo[4,5]thiazolo[3,2-*a*]pyridin-1-one 23

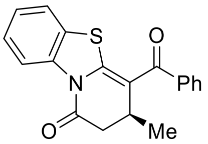

23

^{1}H, CDCl$_3$, 400 MHz

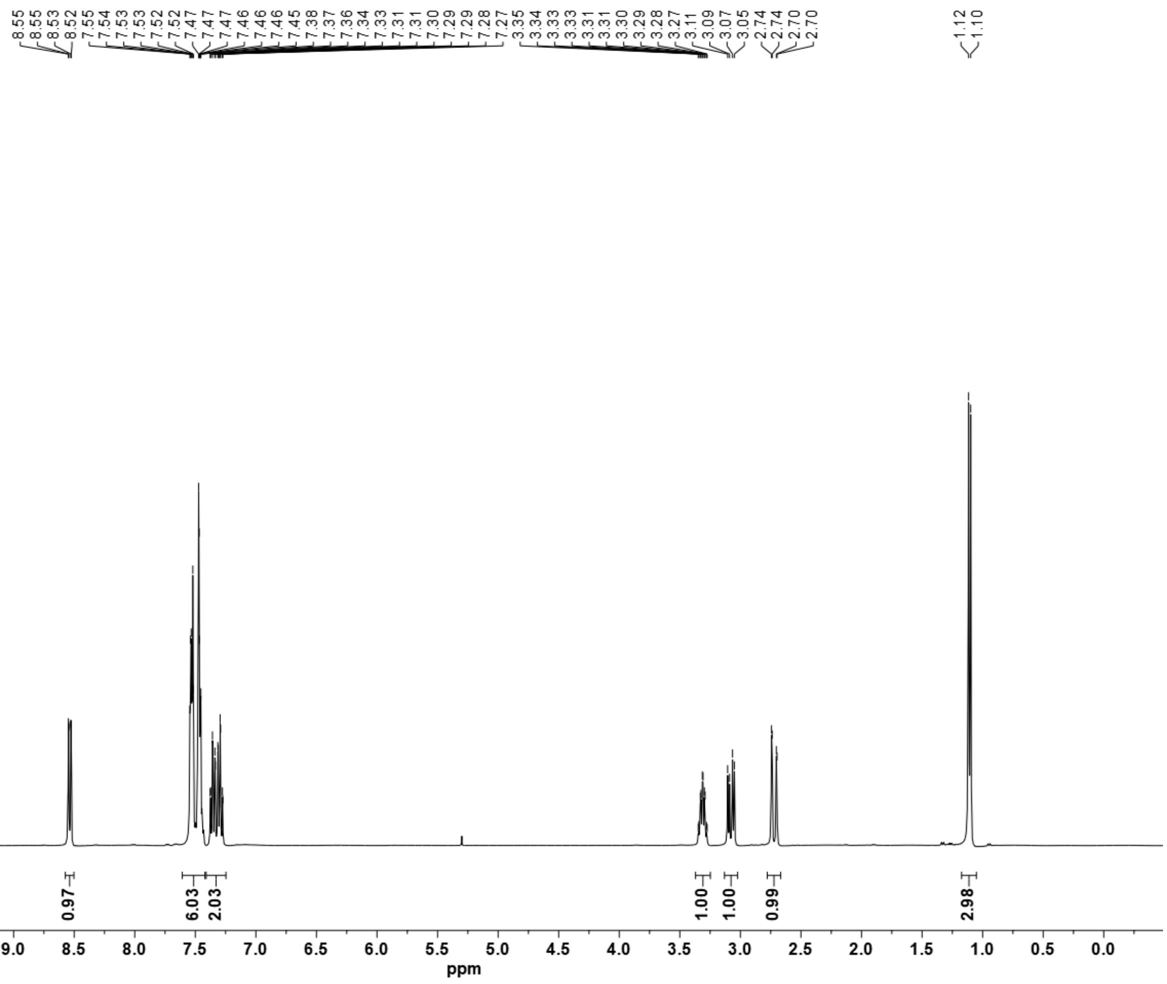

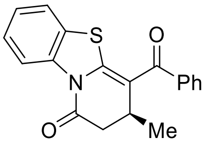

23

^{13}C{^{1}H}, CDCl$_3$, 100 MHz

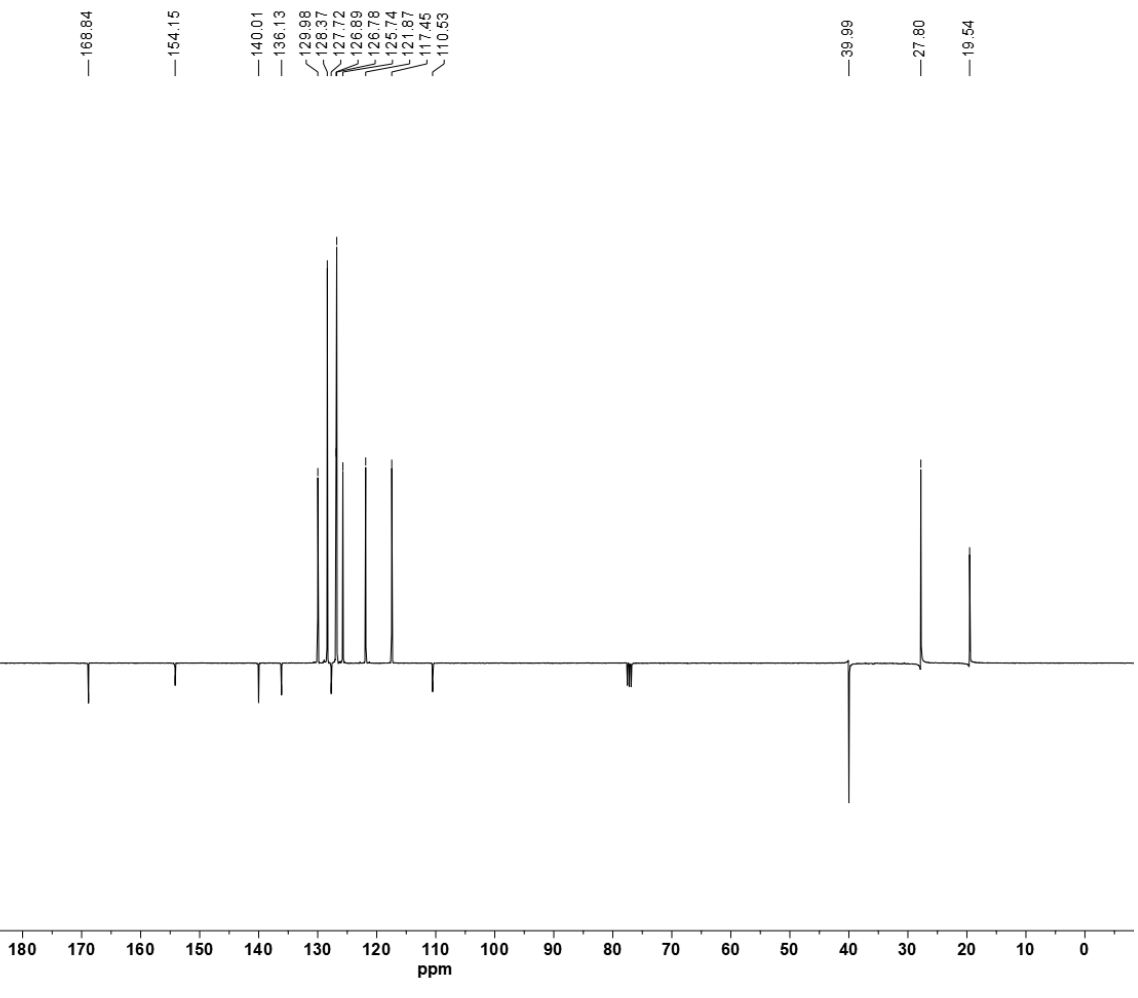

(4*S*)-5-(benzo[d]thiazol-2-yl)-4-methyl-6-phenyl-3,4-dihydro-2*H*-pyran-2-one 24

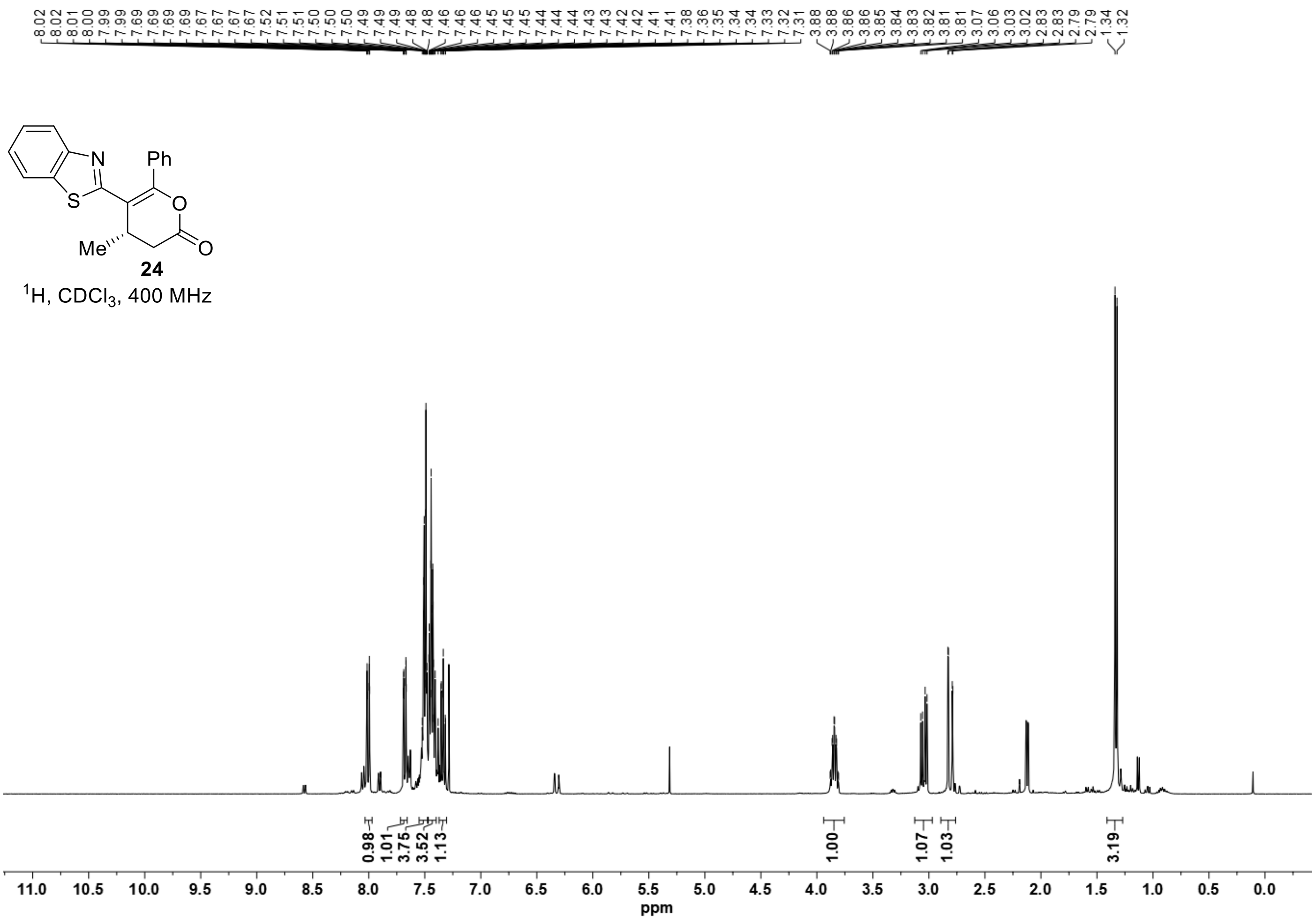

24

^{1}H, $CDCl_3$, 400 MHz

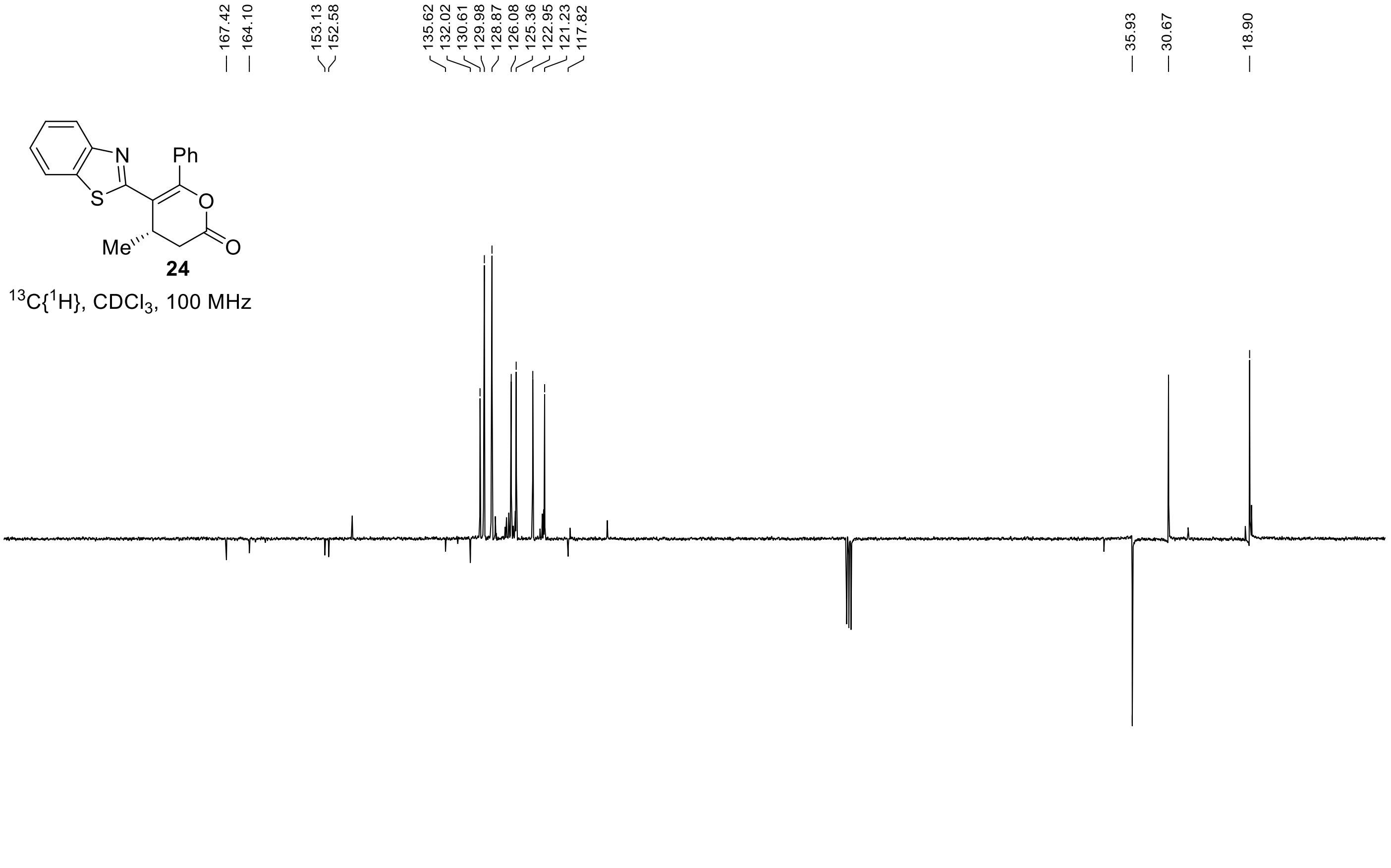

24

$^{13}C\{^{1}H\}$, $CDCl_3$, 100 MHz

(7*R*)-8-benzoyl-7-phenyl-6,7-dihydro-5*H*-thiazolo[3,2-a]pyridin-5-one 25

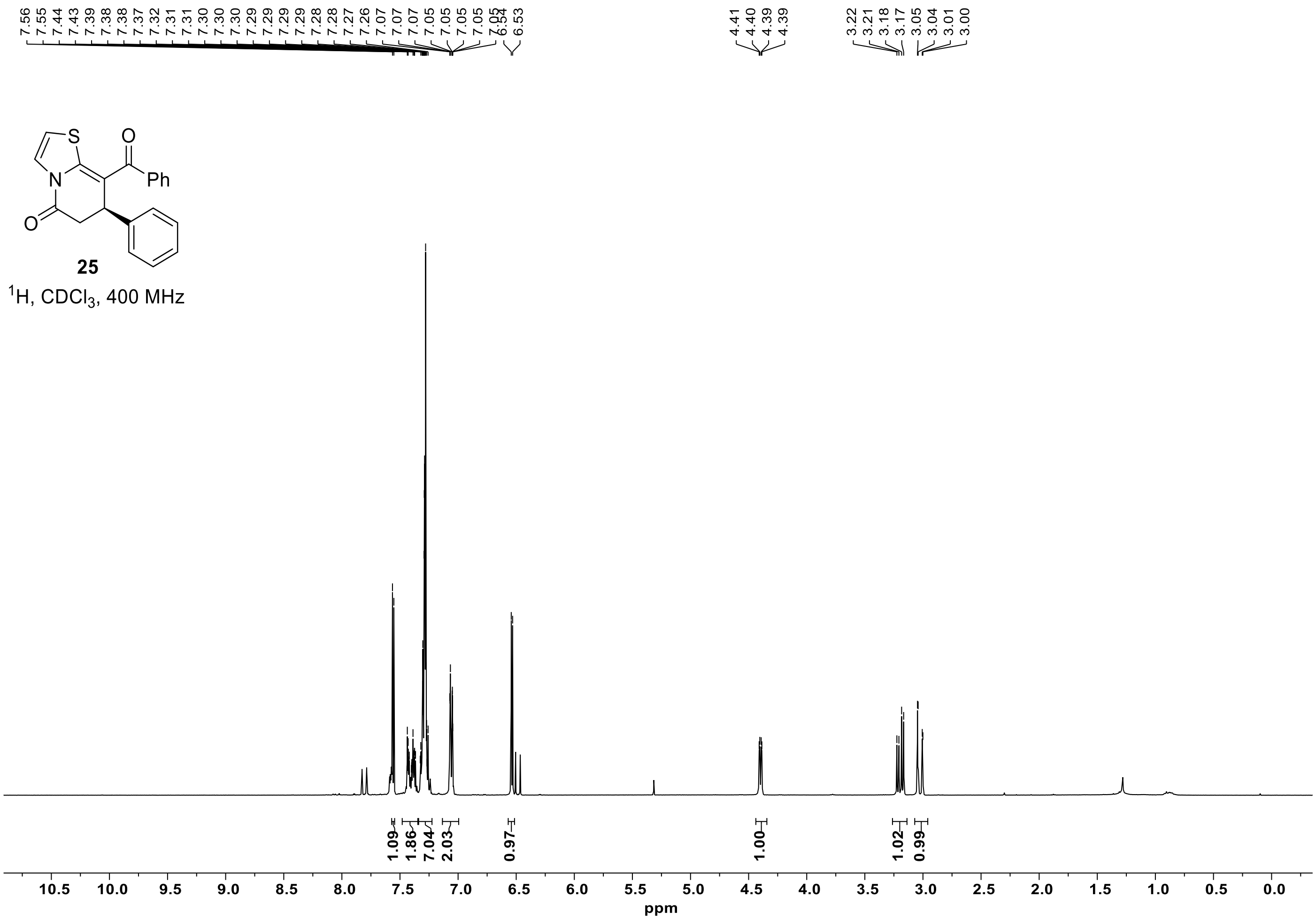

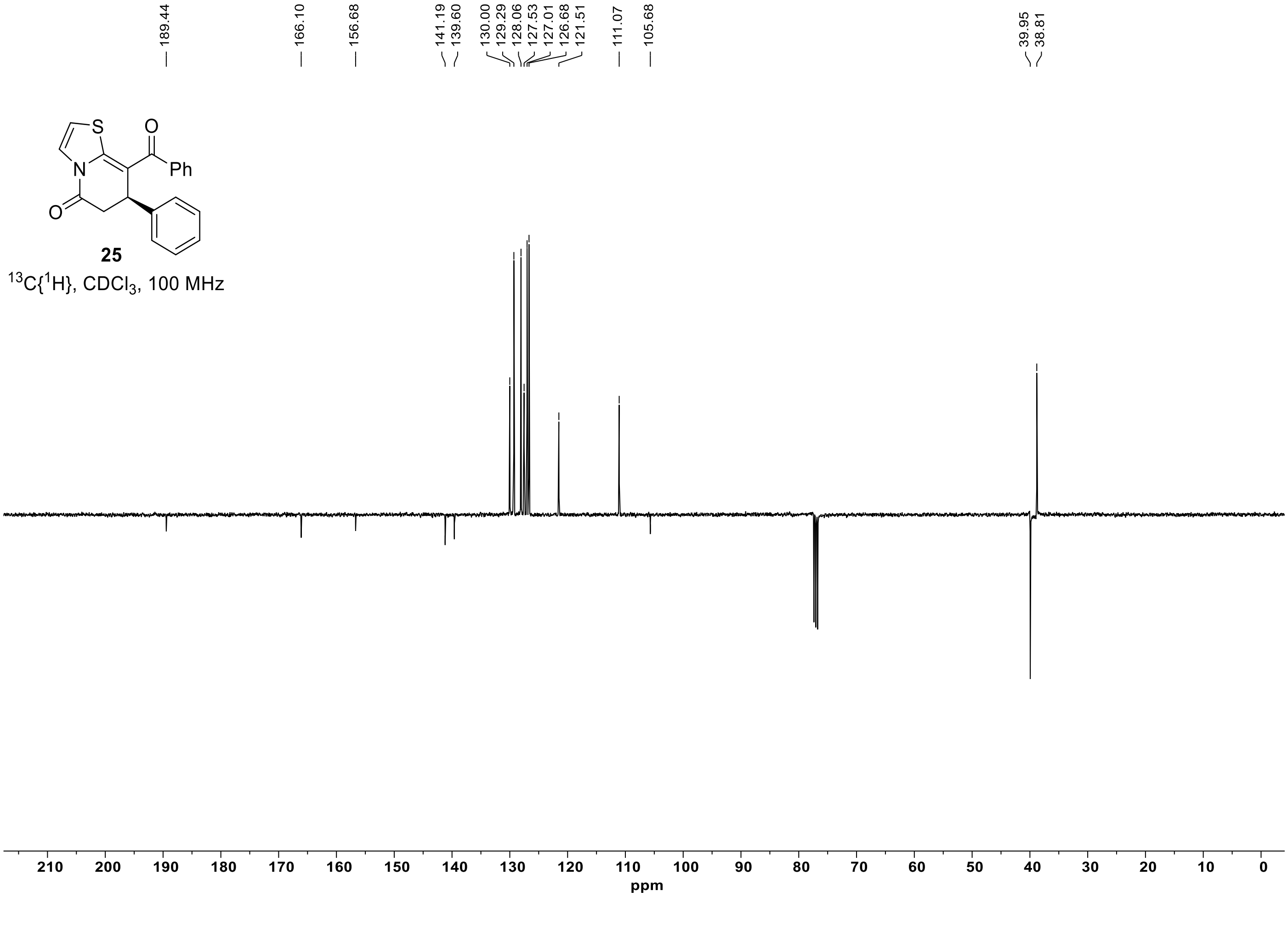

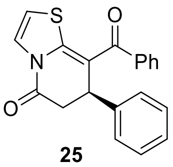

25

2D, ^{1}H-^{1}H, COSY, $CDCl_3$

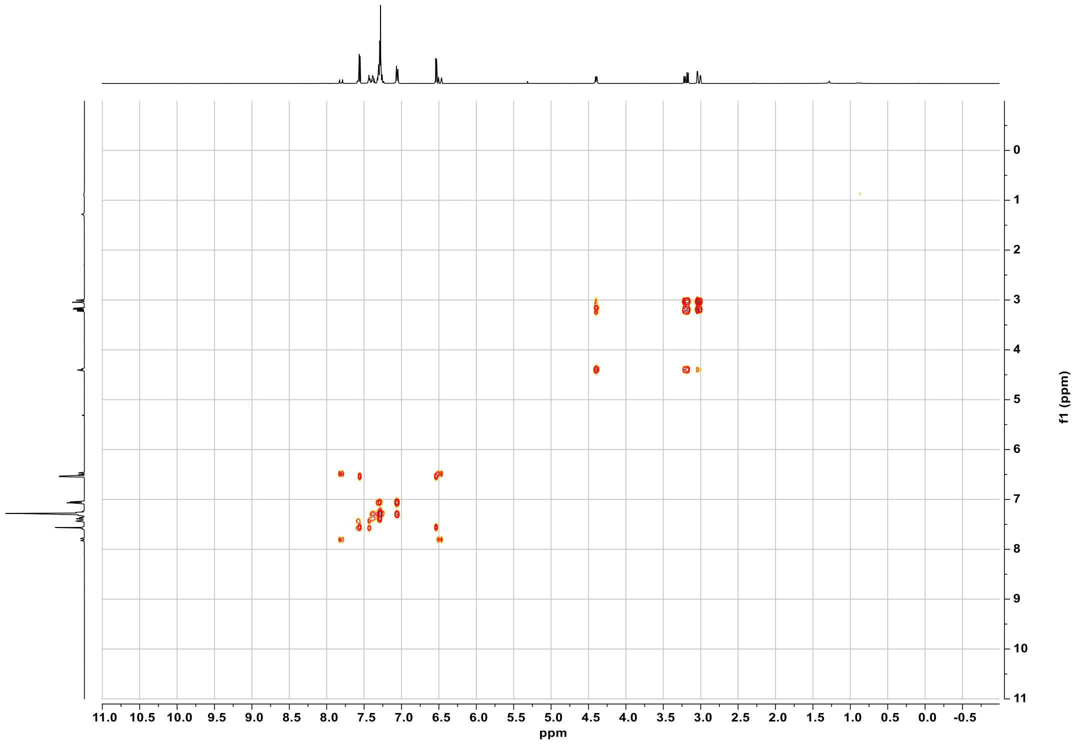

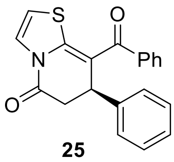

25

2D, ^{1}H-^{13}C, HSQC, $CDCl_3$

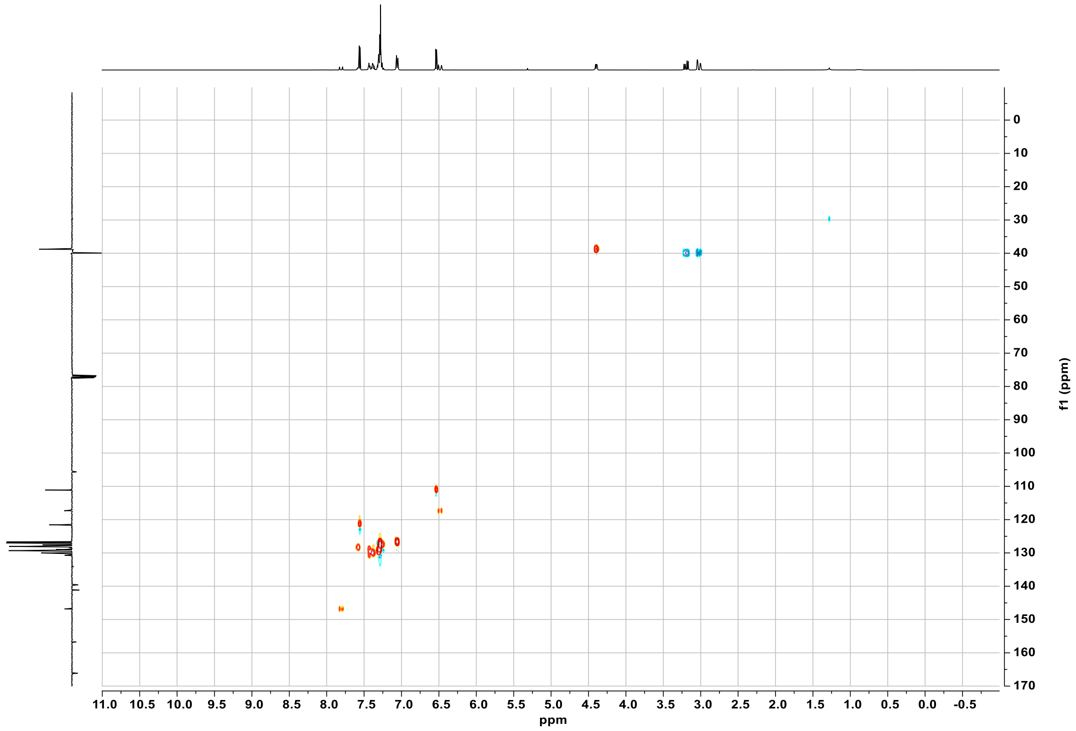

(7*S*)-8-benzoyl-7-methyl-6,7-dihydro-5*H*-thiazolo[3,2-a]pyridin-5-one 26

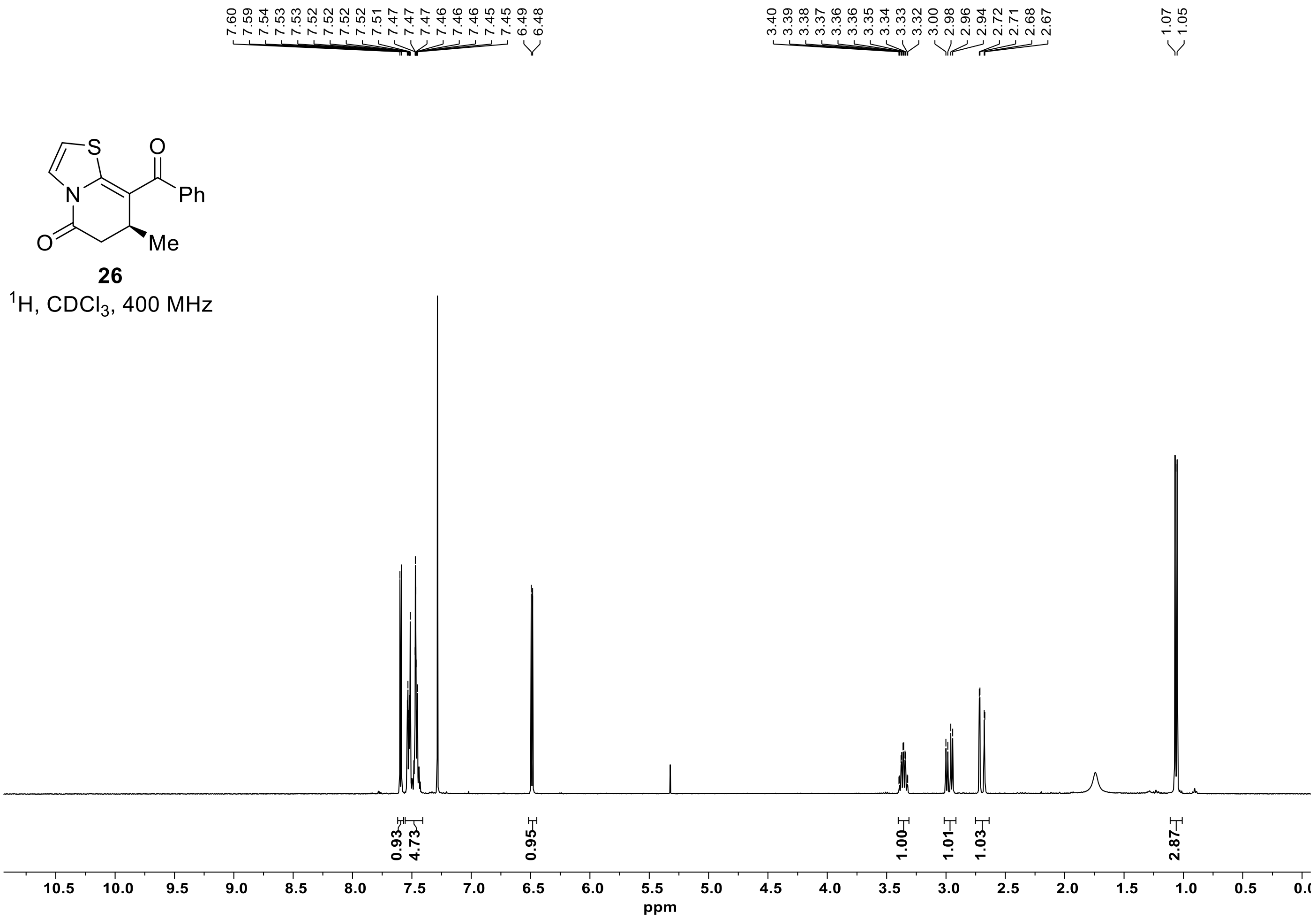

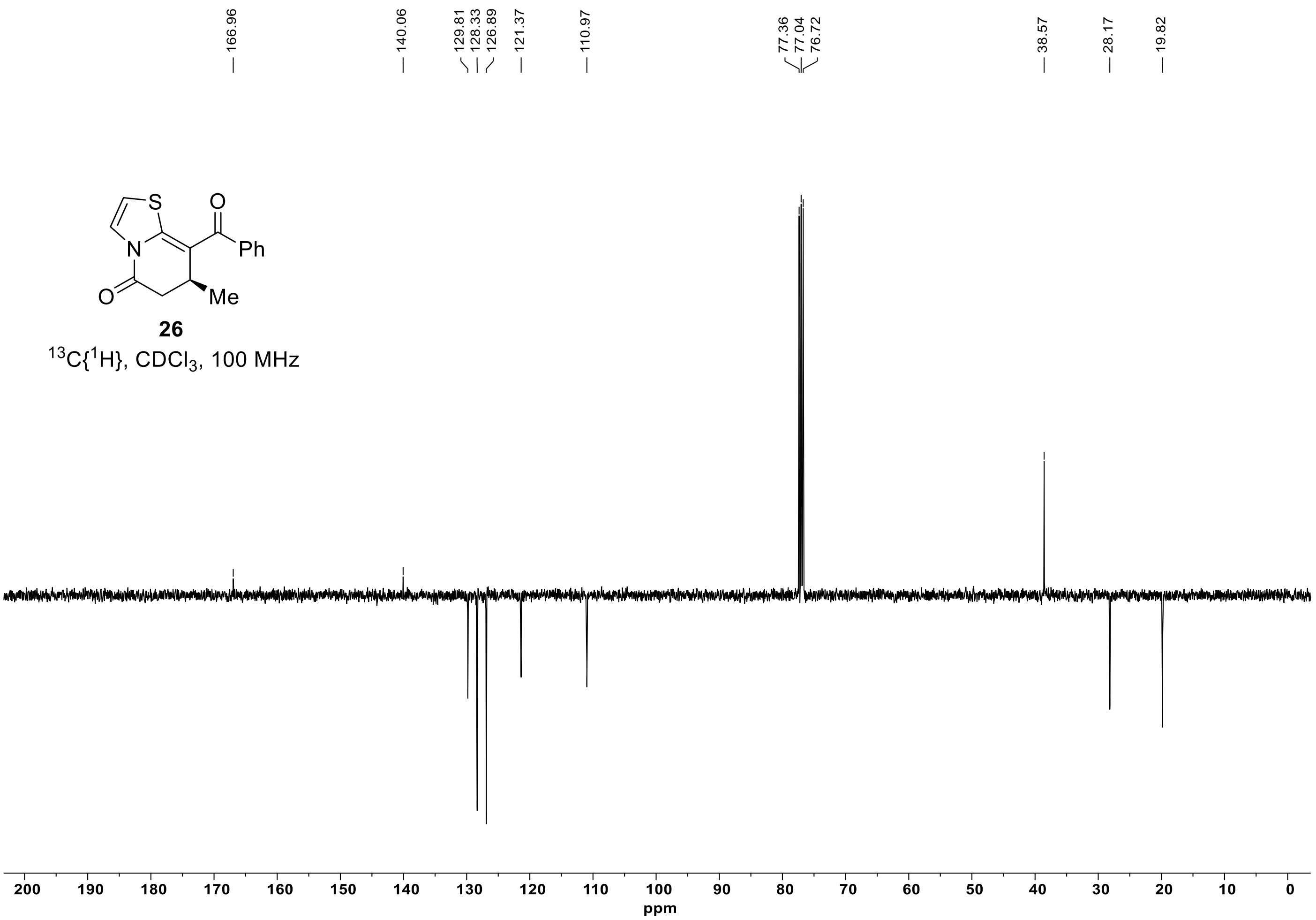

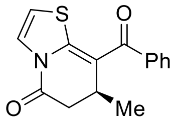

26

2D, ^{1}H-^{1}H, COSY, $CDCl_3$

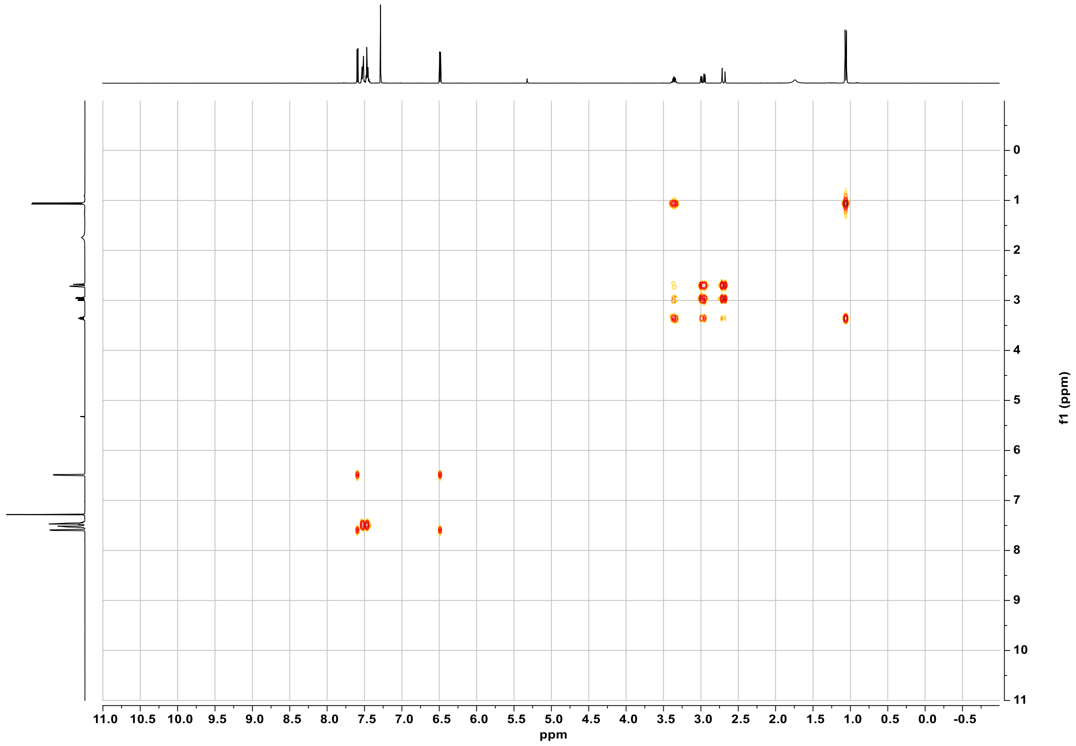

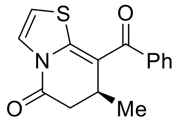

26

2D, ^{1}H-^{13}C, HSQC, $CDCl_3$

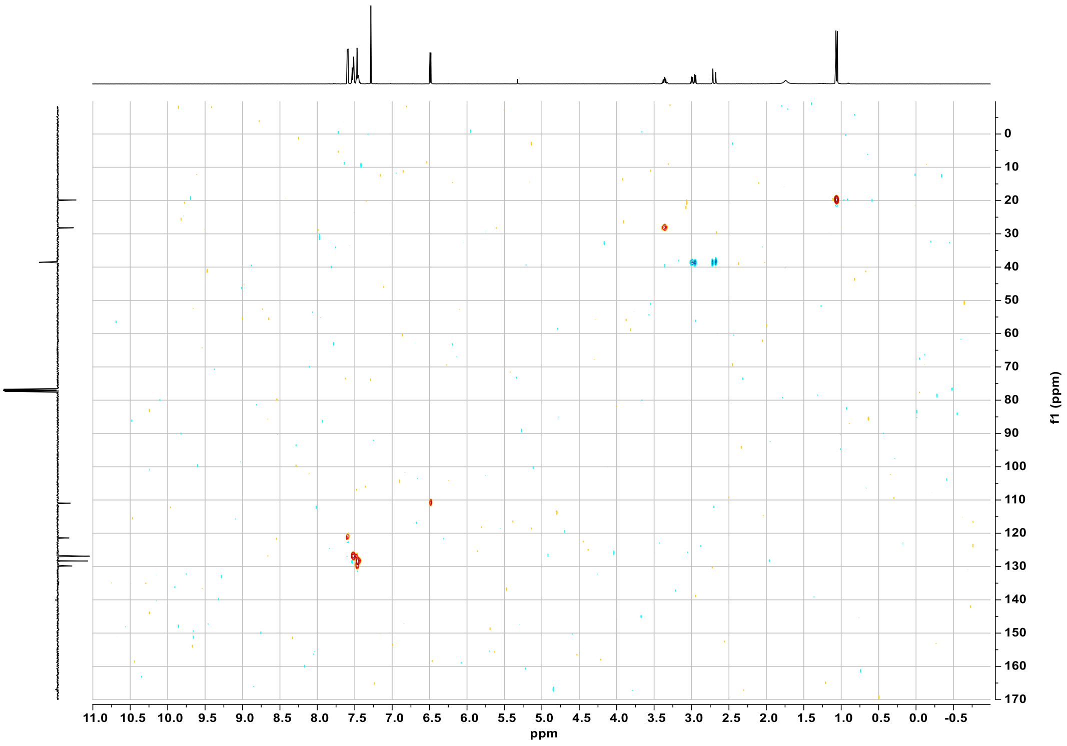

(4*R*)-5-(benzo[d]oxazol-2-yl)-4,6-diphenyl-3,4-dihydro-2*H*-pyran-2-one 27

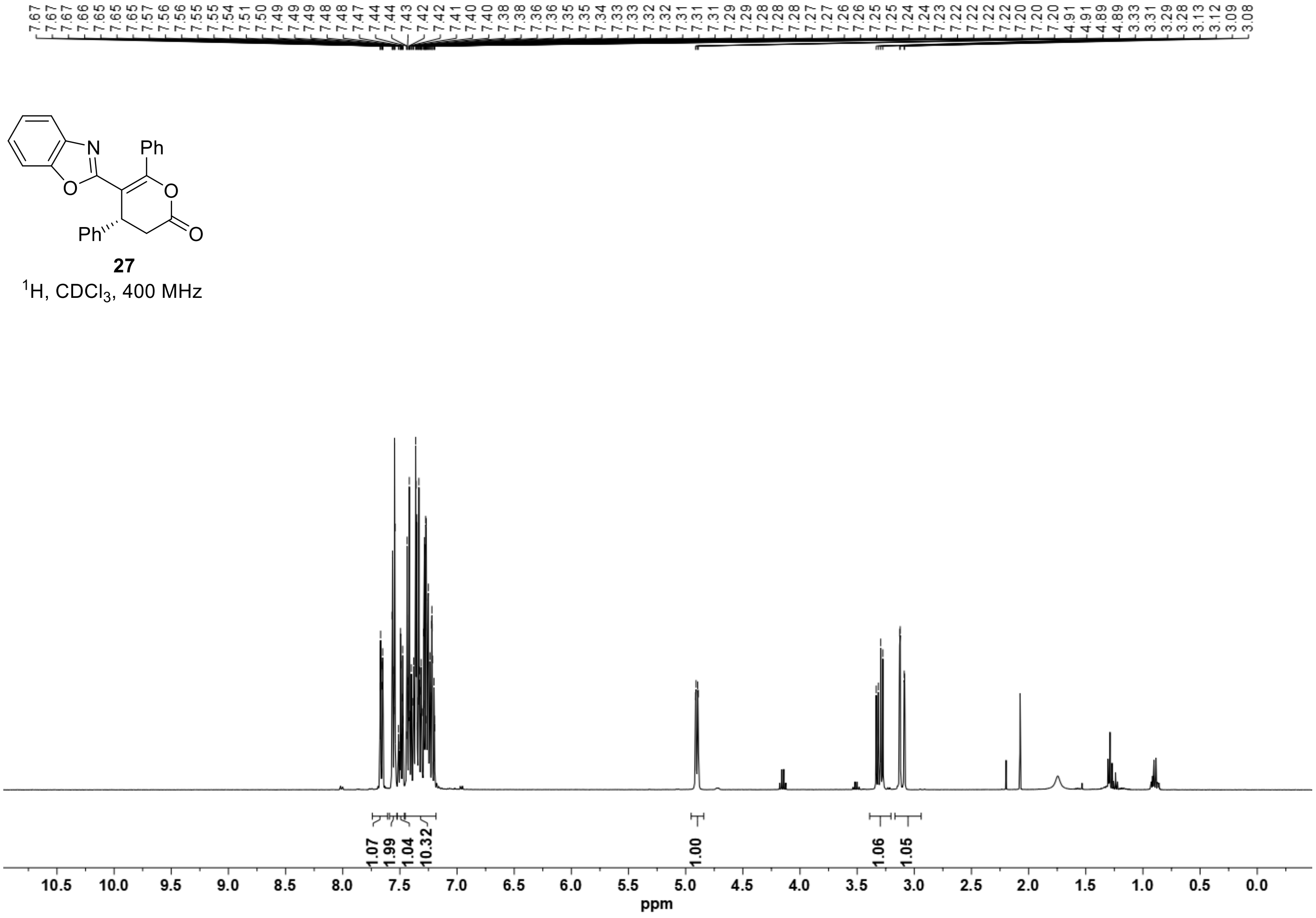

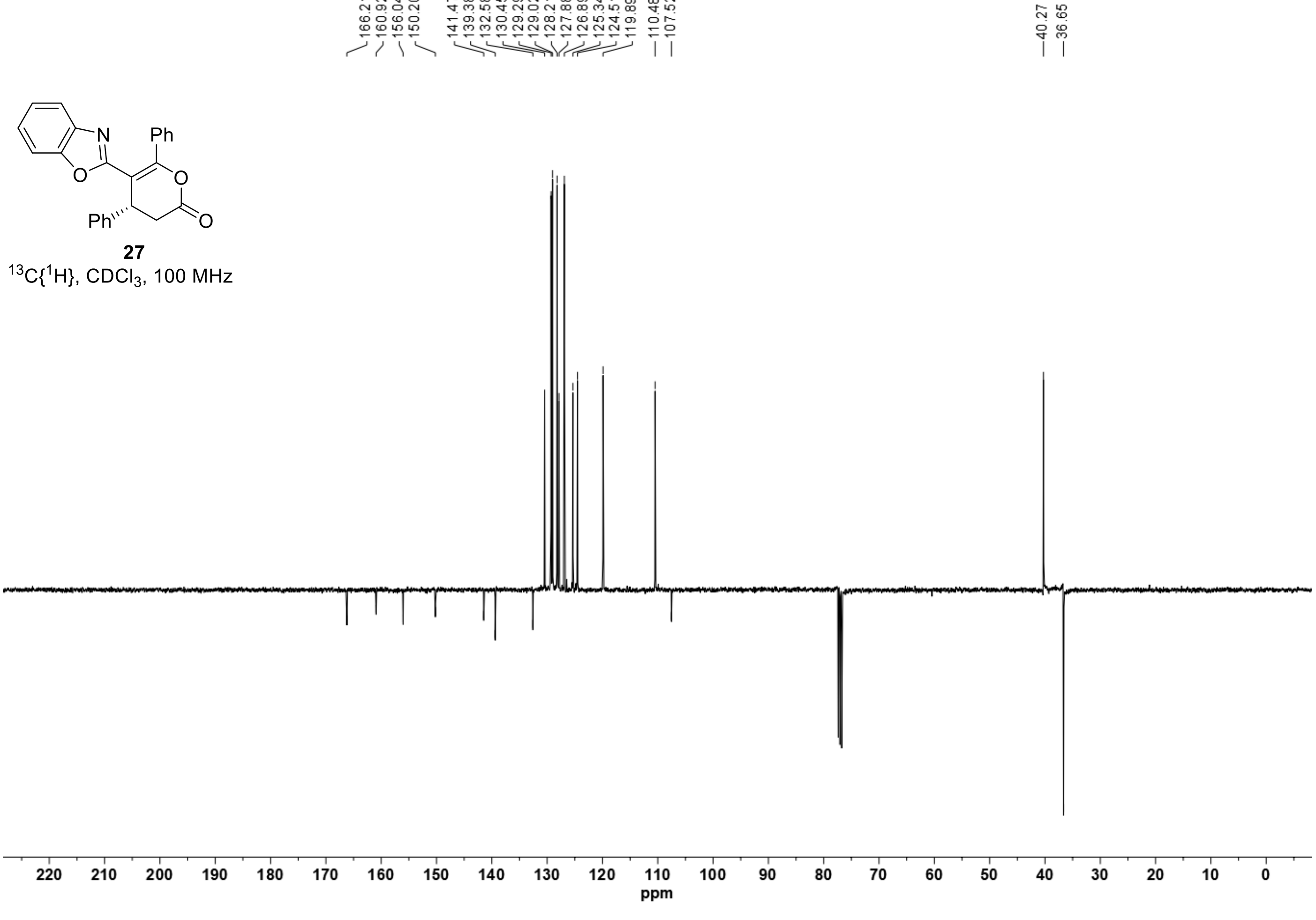

(4*S*)-5-(benzo[d]oxazol-2-yl)-4-methyl-6-phenyl-3,4-dihydro-2*H*-pyran-2-one 28

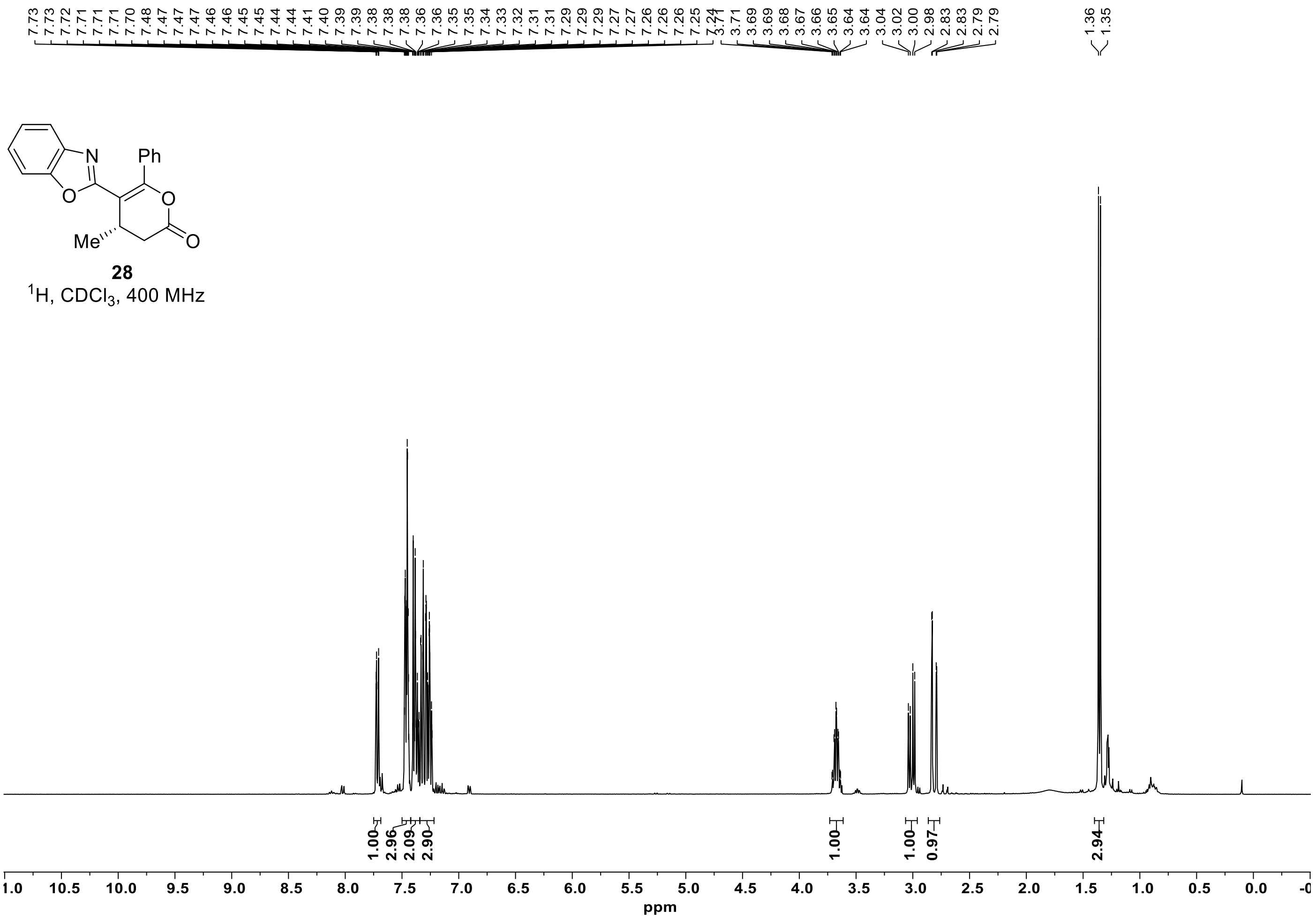

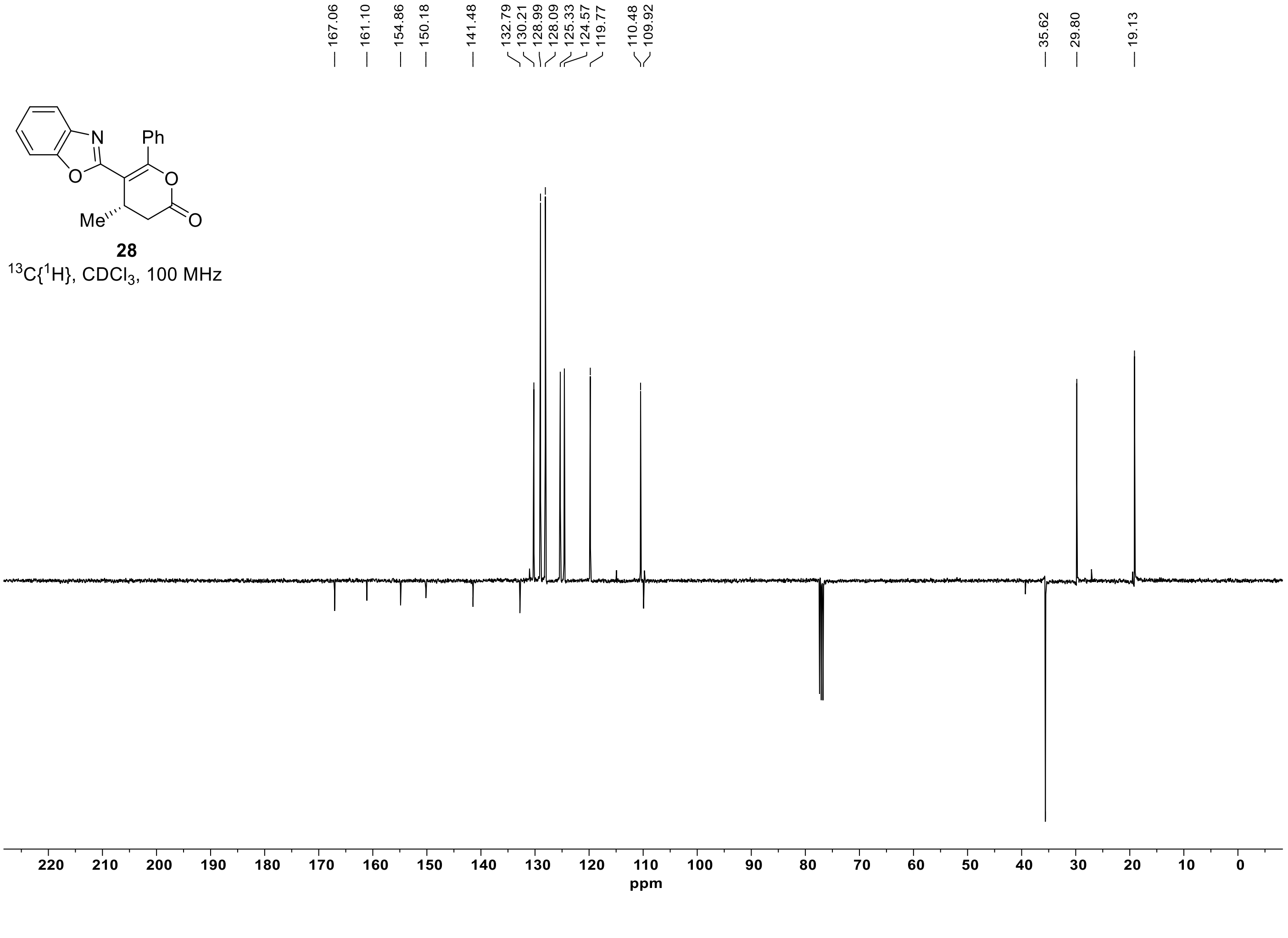

(4*R*)-2-(2-oxo-4,6-diphenyl-3,4-dihydro-2*H*-pyran-5-yl)-1*H*-benzo[d]imidazole-1-carboxylic acid 29

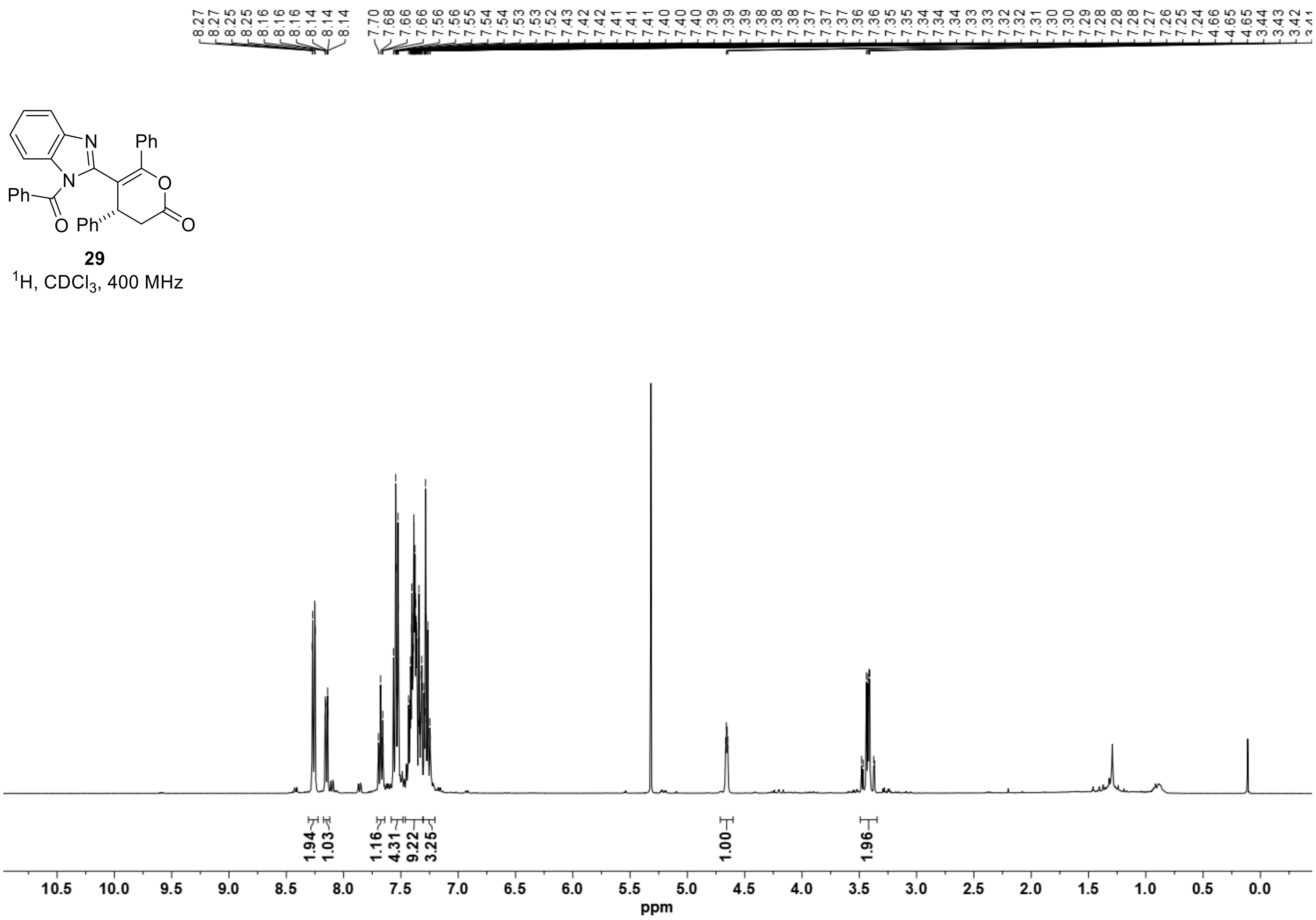

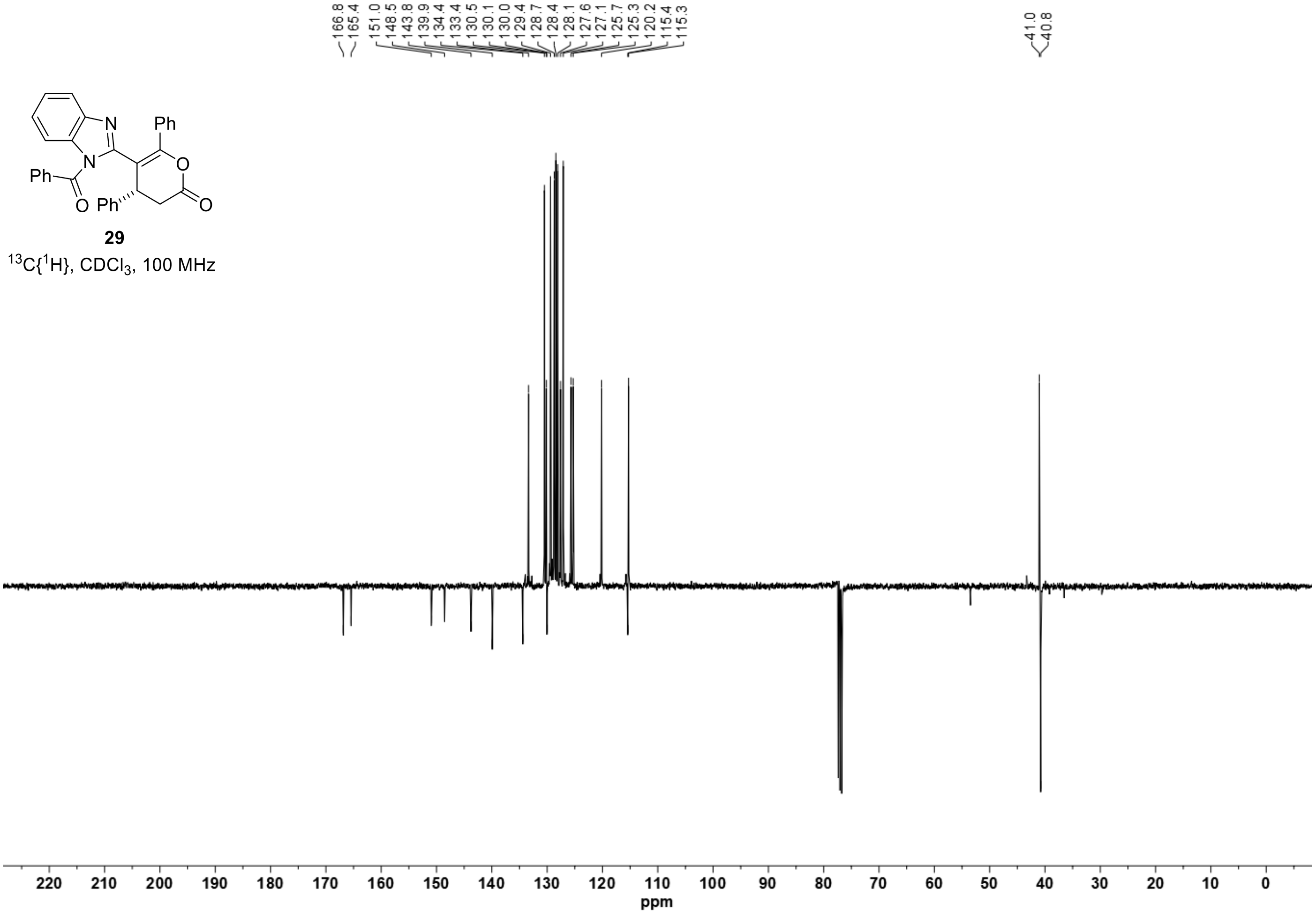

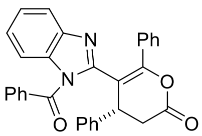

29

2D, ^{1}H-^{1}H, COSY, $CDCl_3$

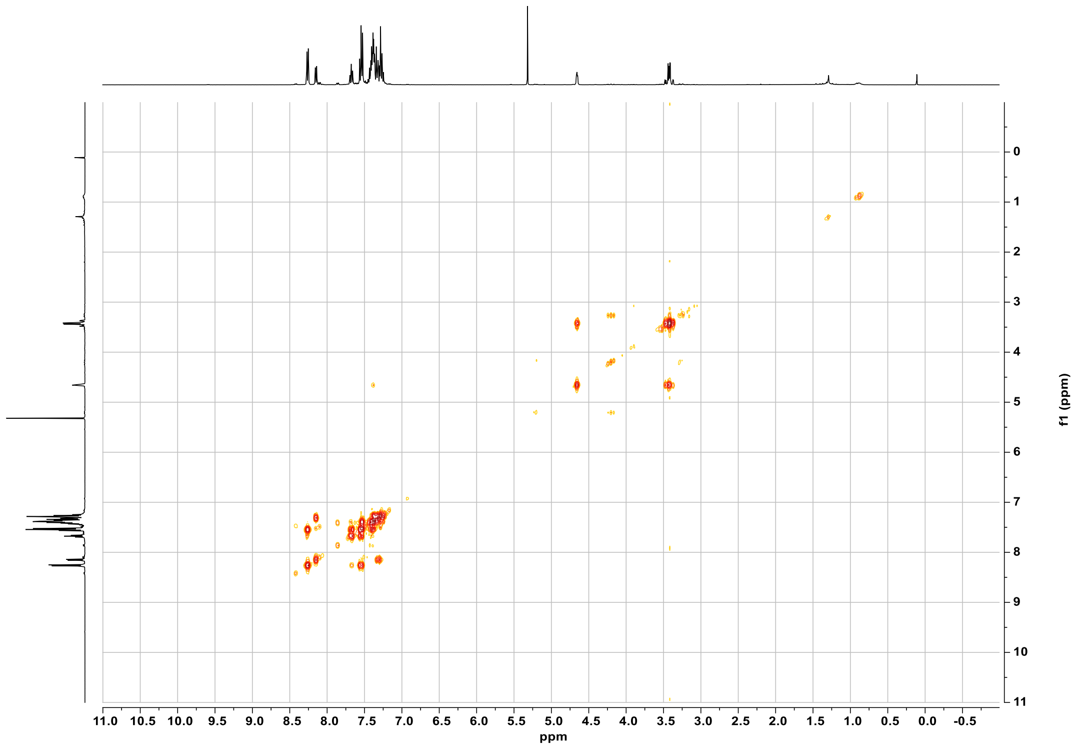

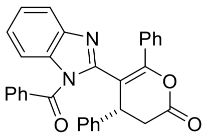

29

2D, ^{1}H-^{13}C, HSQC, $CDCl_3$

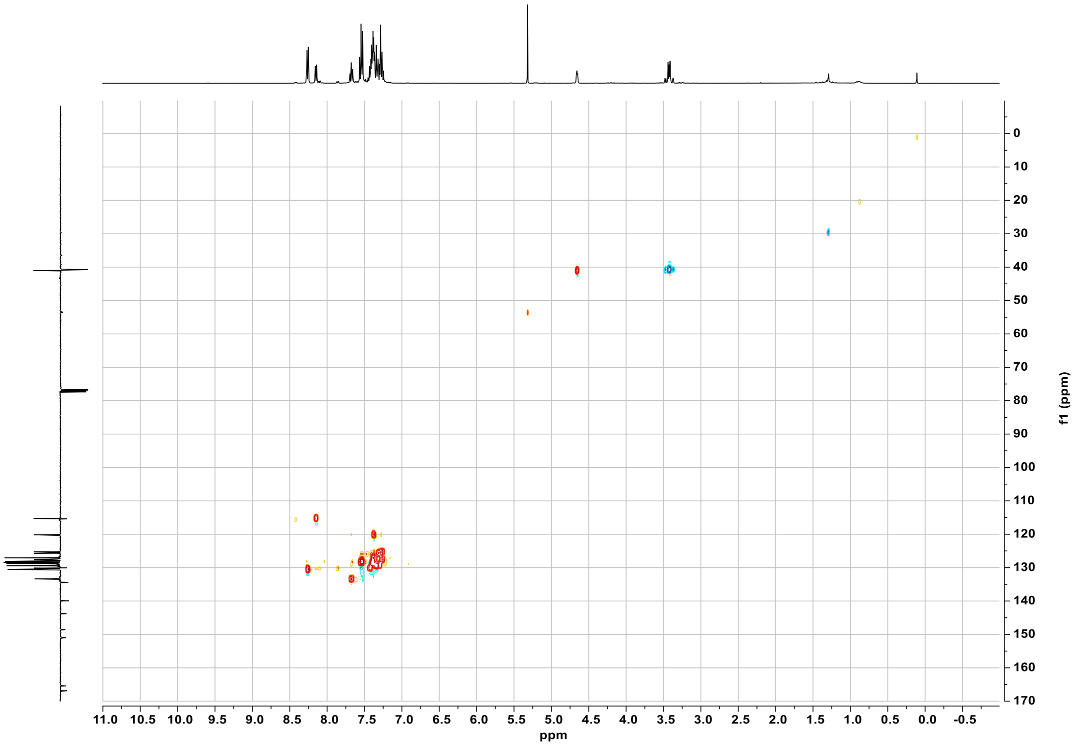

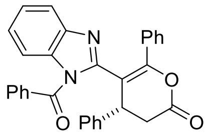

29

2D, ^{1}H-^{13}C, HMBC, $CDCl_3$

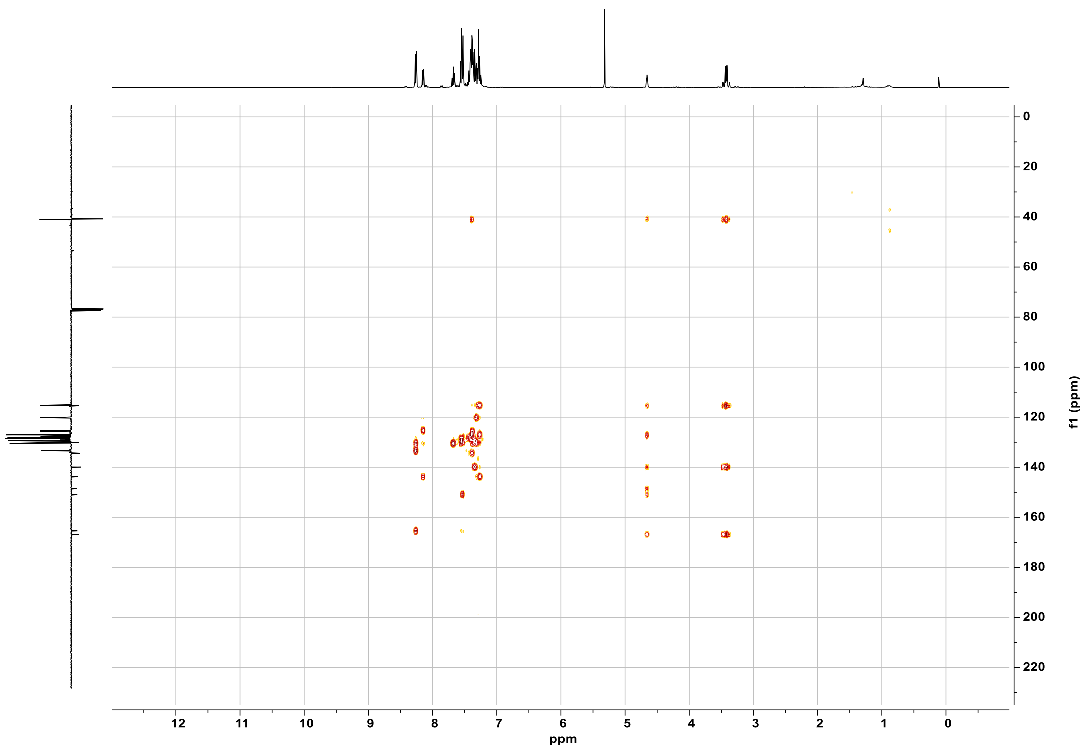

(4*S*)-5-(1-benzoyl-1*H*-benzo[d]imidazol-2-yl)-4-methyl-6-phenyl-3,4-dihydro-2*H*-pyran-2-one 30

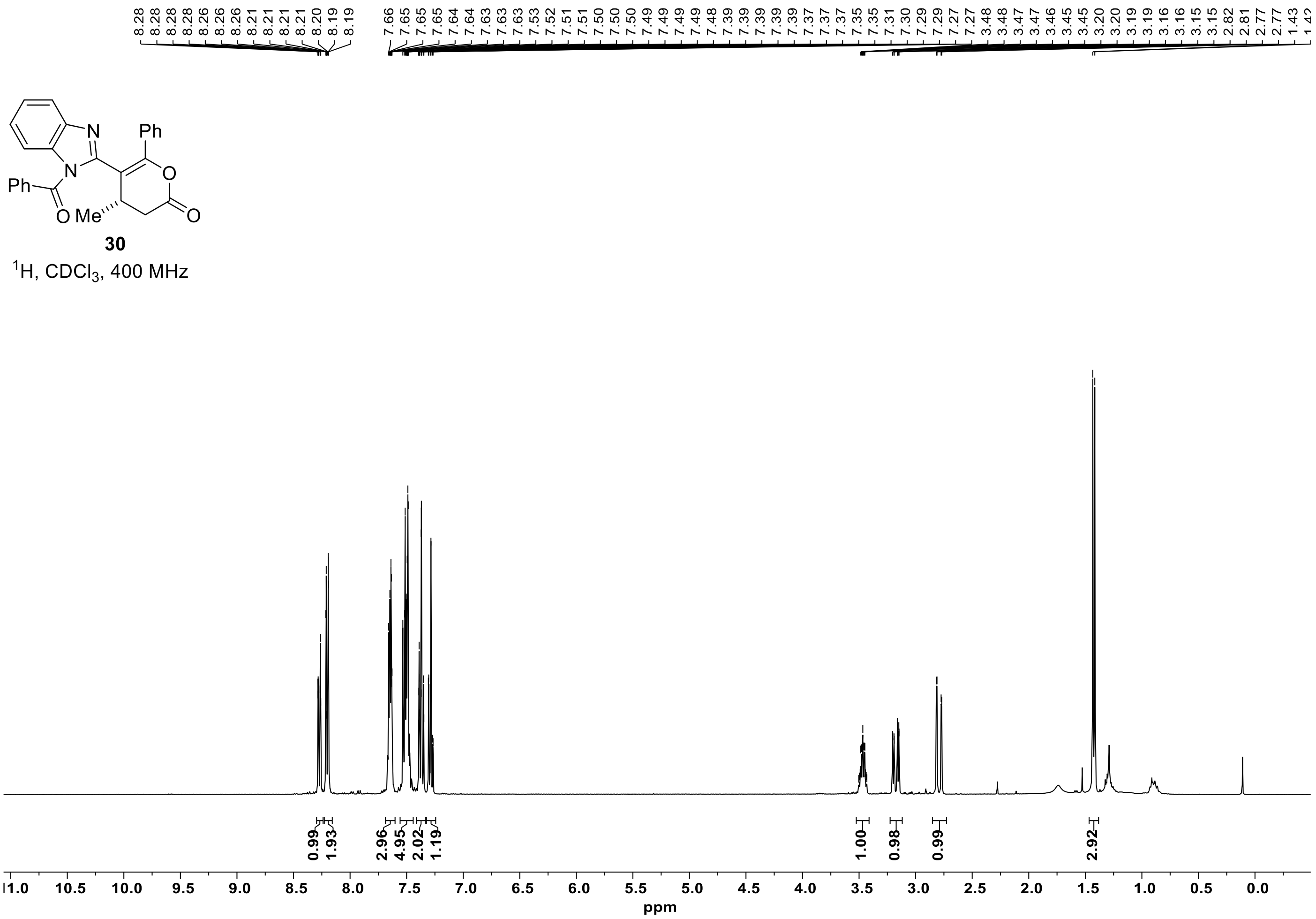

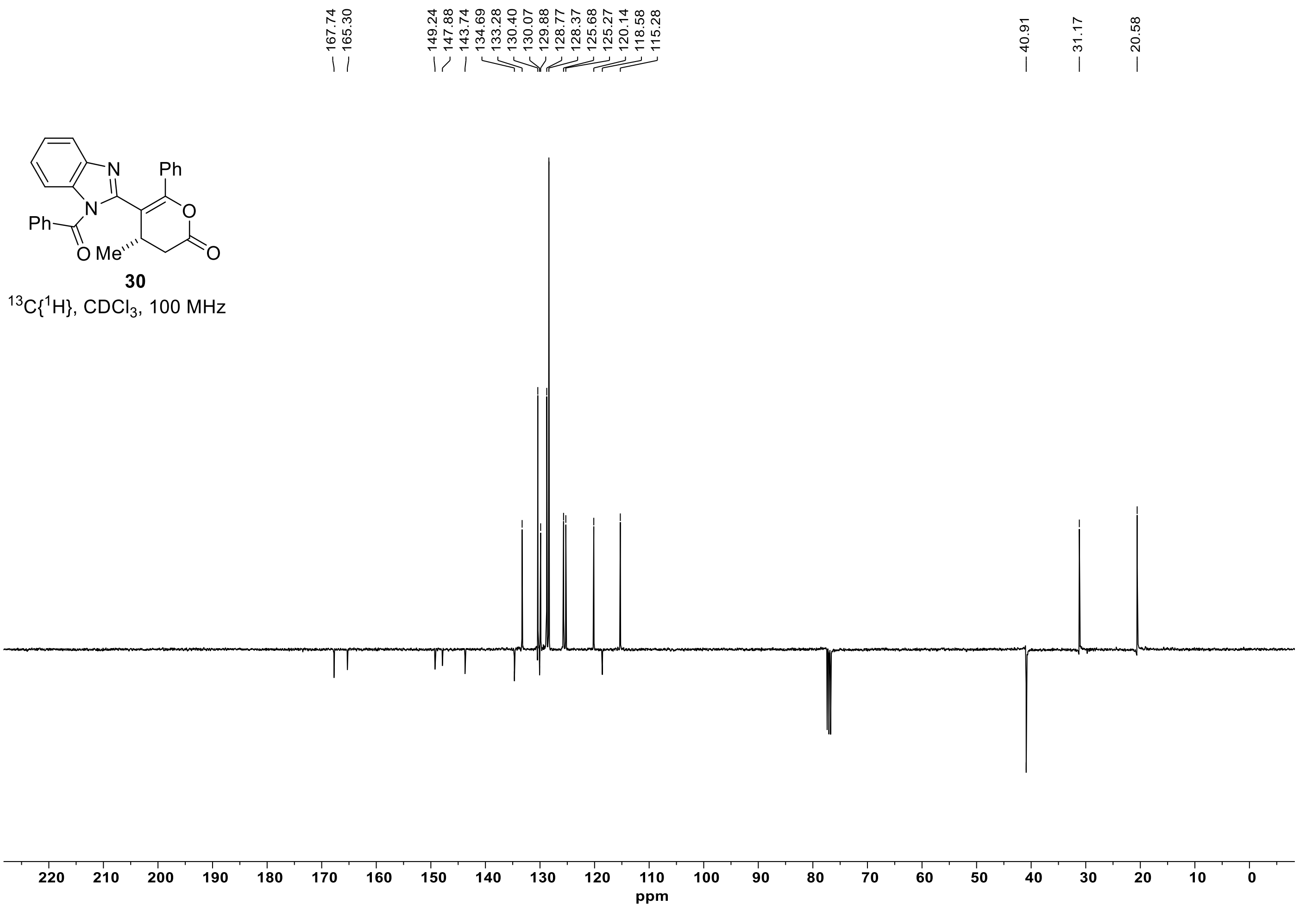

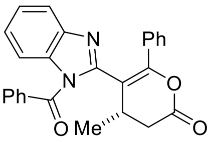

30

2D, ^{1}H-^{1}H, COSY, $CDCl_3$

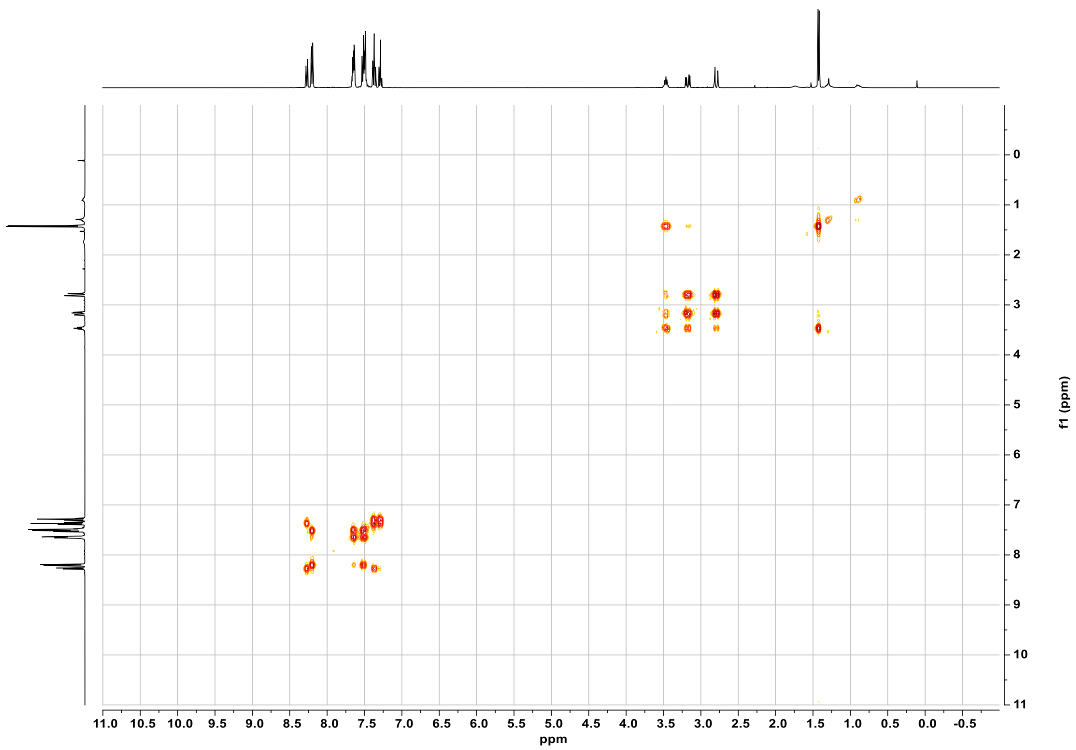

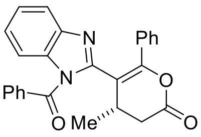

30

2D, ^{1}H-^{13}C, HSQC, $CDCl_3$

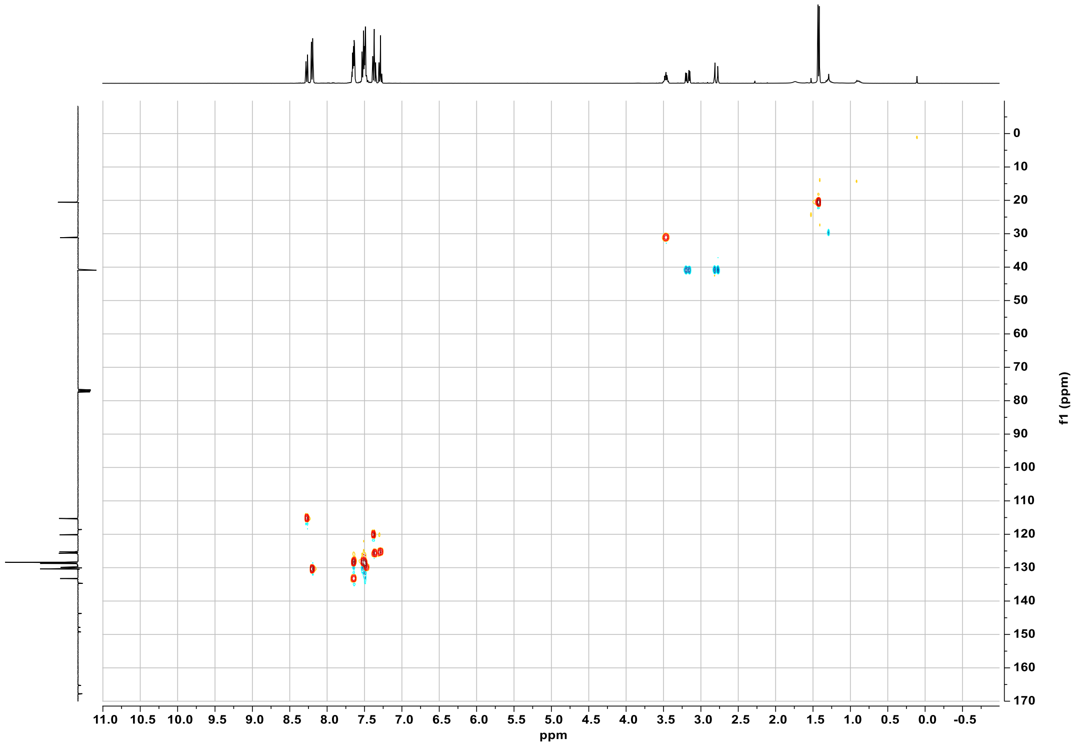

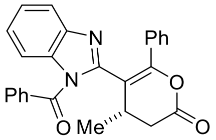

30

2D, ^{1}H-^{13}C, HMBC, $CDCl_3$

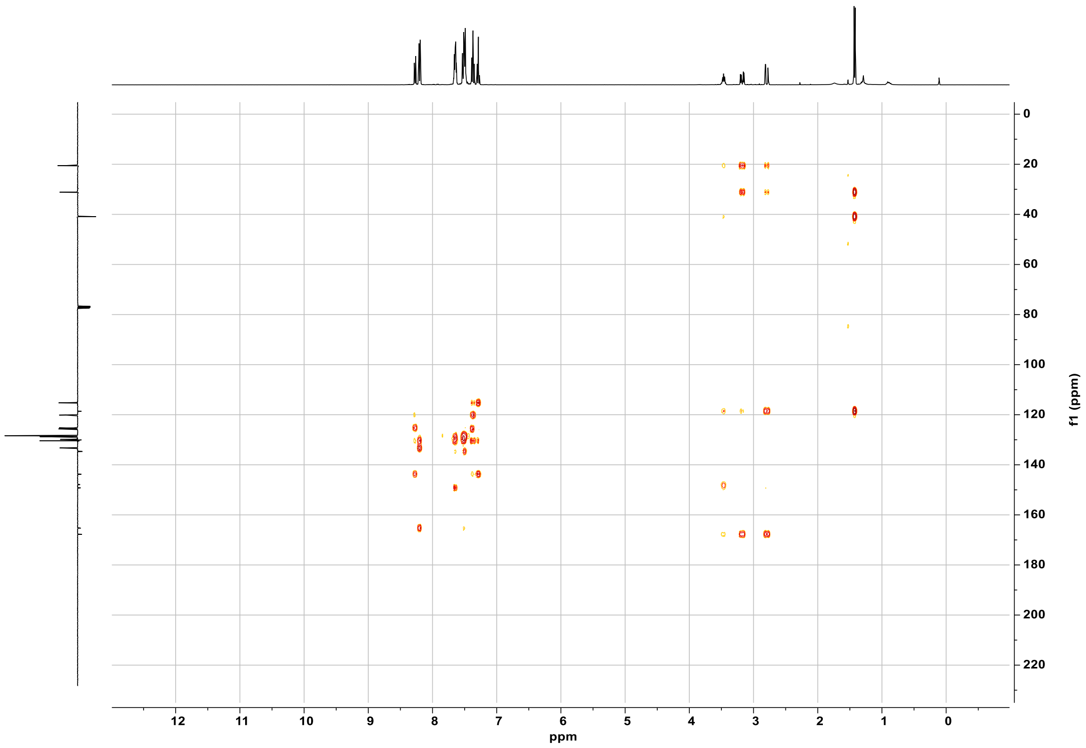

(3*S*,4*E*)-(3-methyl-1-oxo-2,3-dihydrobenzo[4,5]imidazo[1,2-*a*]pyridin-4(1*H*)-ylidene)(phenyl)methyl benzoate 31

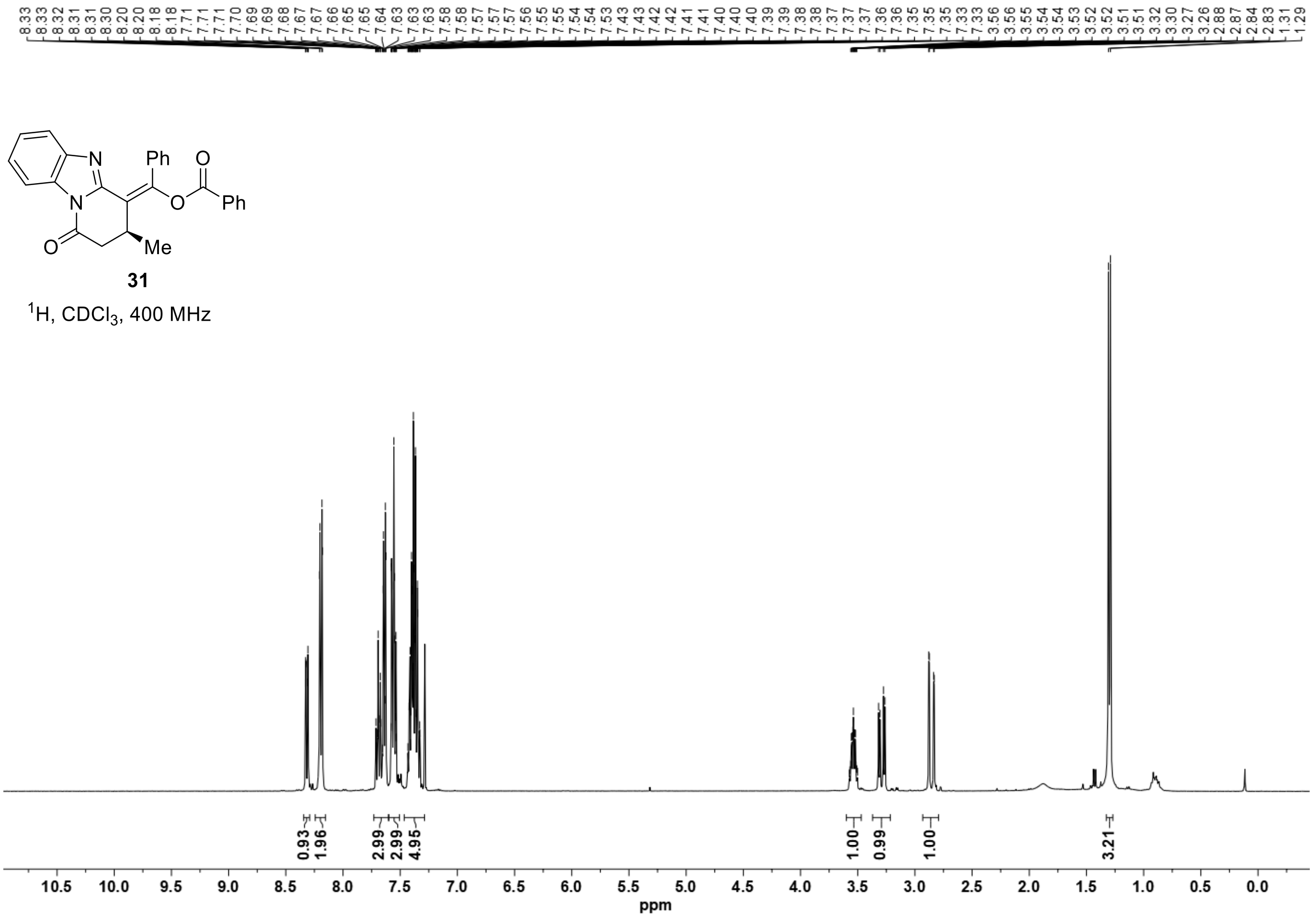

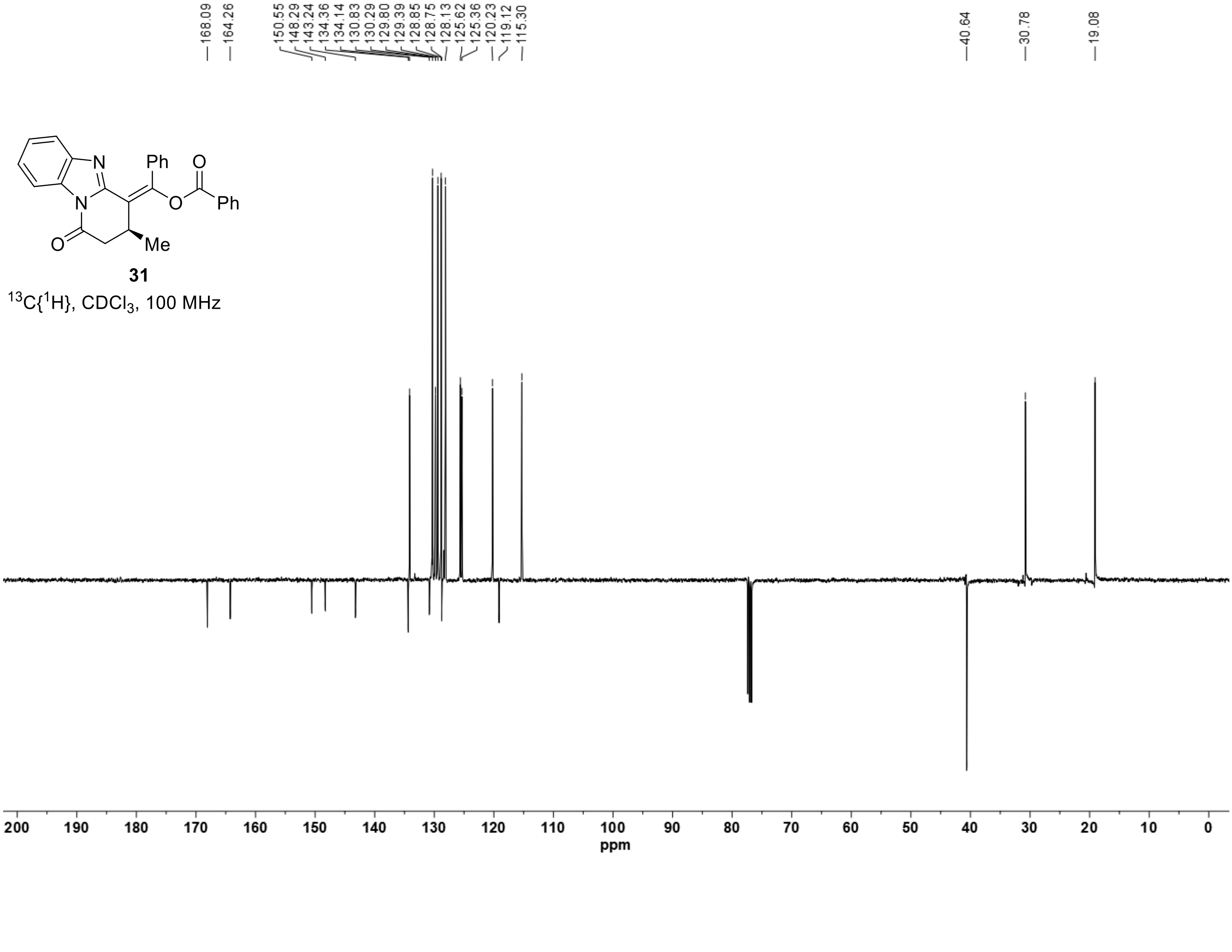

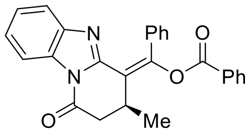

31

2D, ^{1}H-^{1}H, COSY, $CDCl_3$

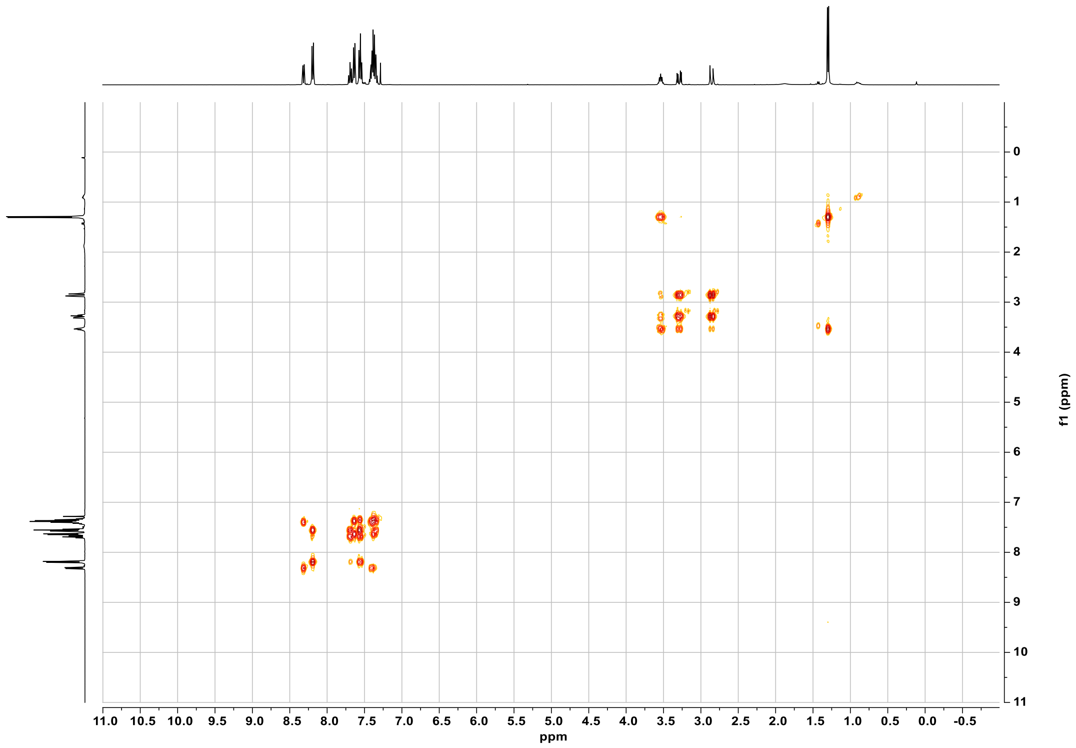

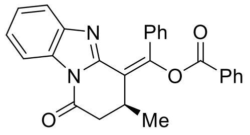

31

2D, ^{1}H-^{13}C, HSQC, $CDCl_3$

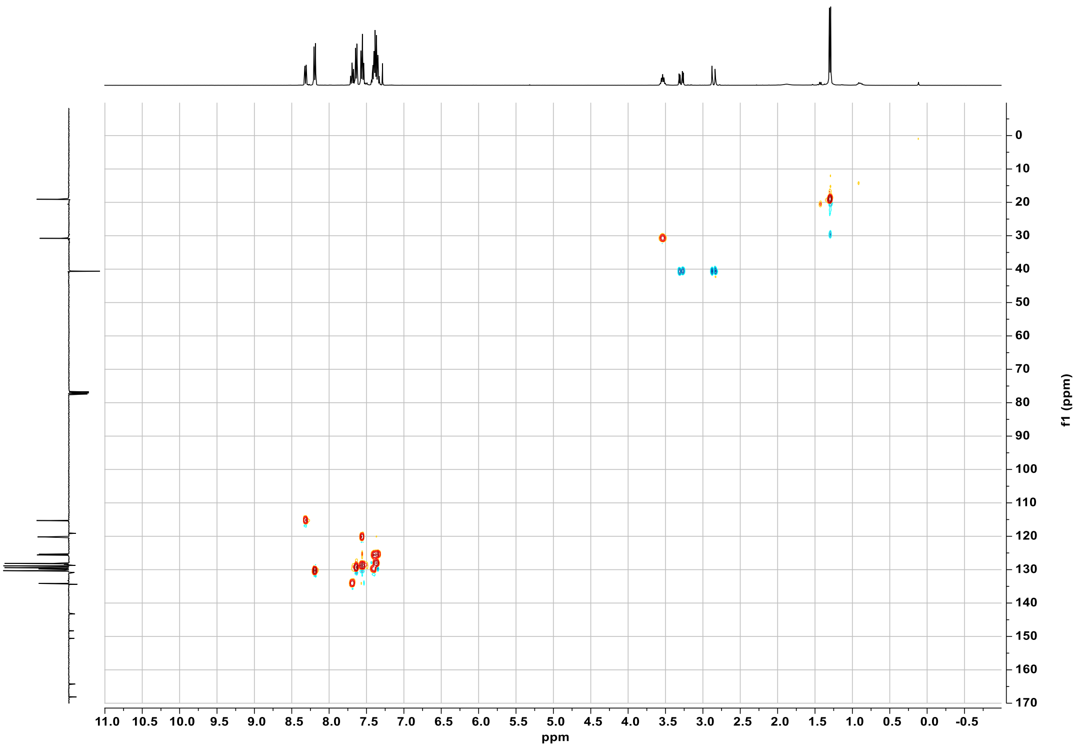

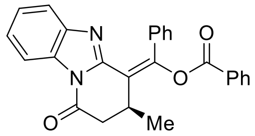

31

2D, ^{1}H-^{13}C, HMBC, $CDCl_3$

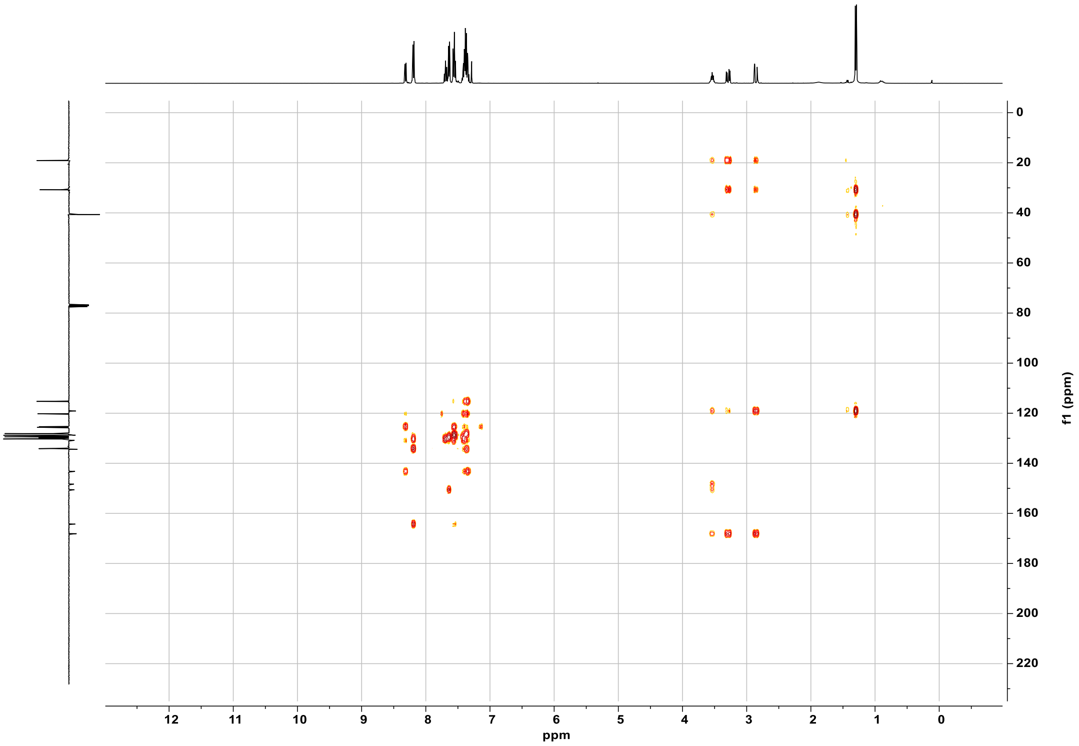

(3*R*)-*N*,*N*-dimethyl-1-oxo-3-phenyl-2,3-dihydro-1*H*-benzo[4,5]thiazolo[3,2-*a*]pyridine-4-carboxamide 32

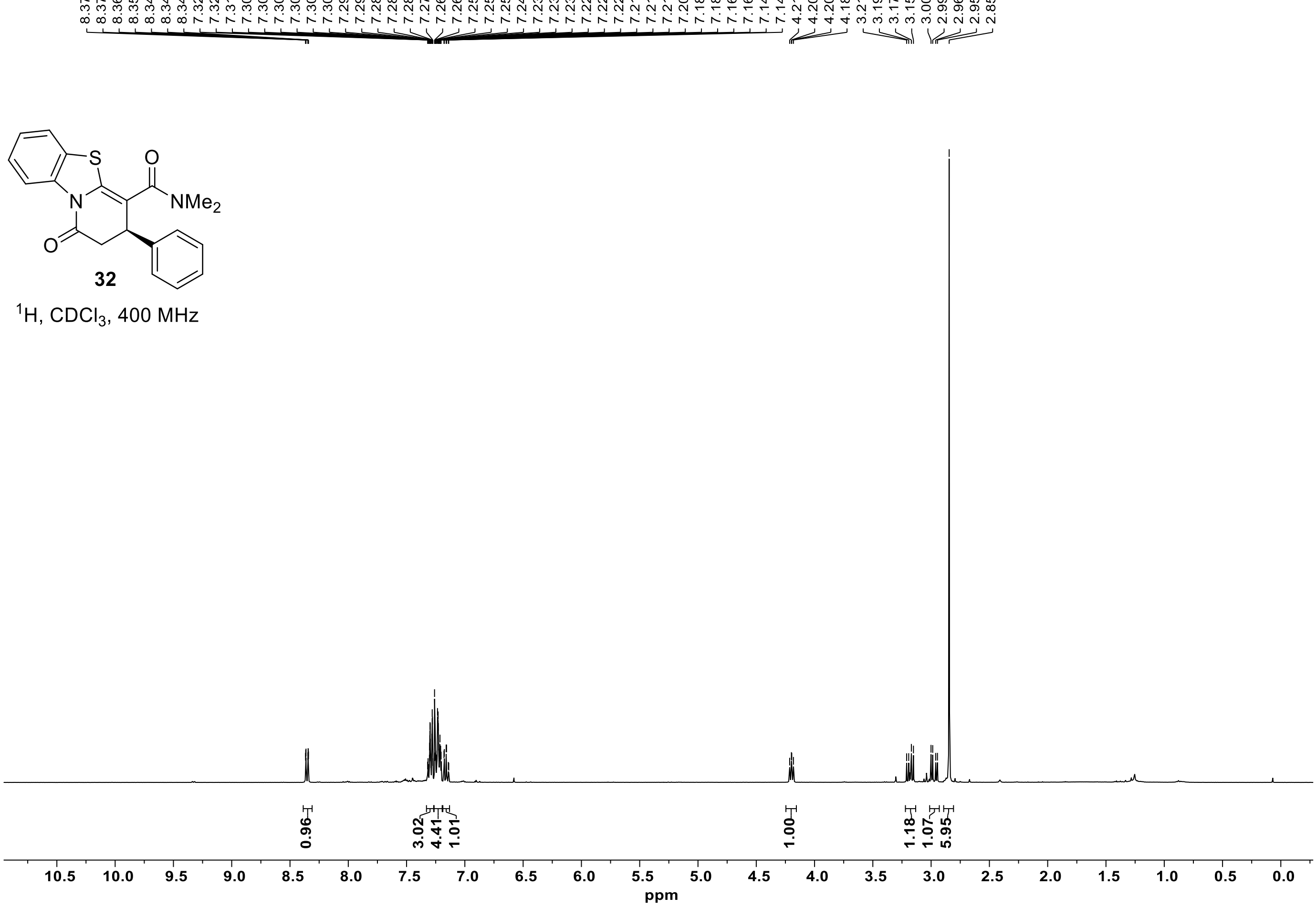

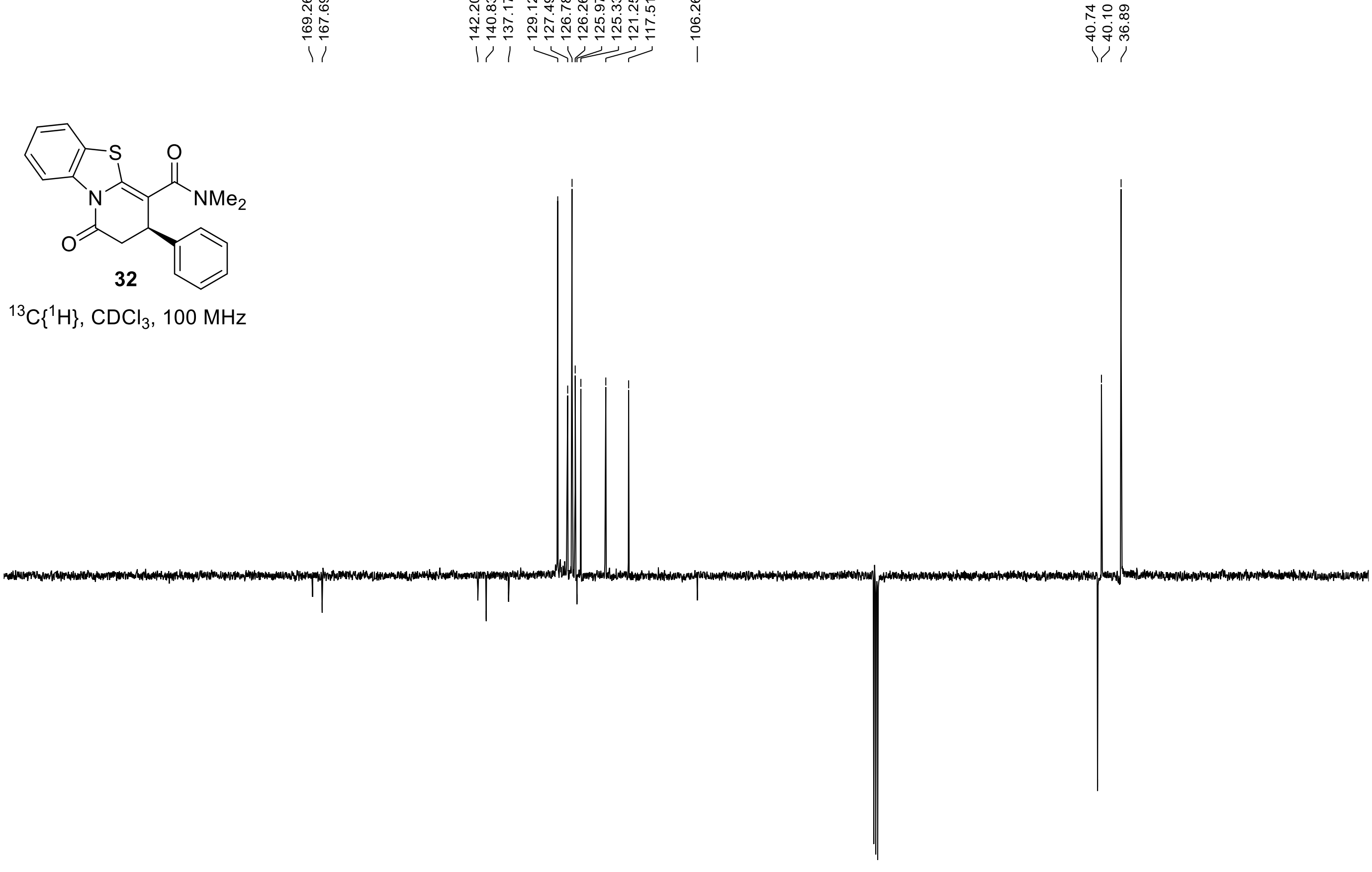

(3*S*)-*N*,*N*,3-trimethyl-1-oxo-2,3-dihydro-1*H*-benzo[4,5]thiazolo[3,2-*a*]pyridine-4-carboxamide 33

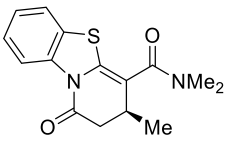

33

^{1}H, $CDCl_3$, 400 MHz

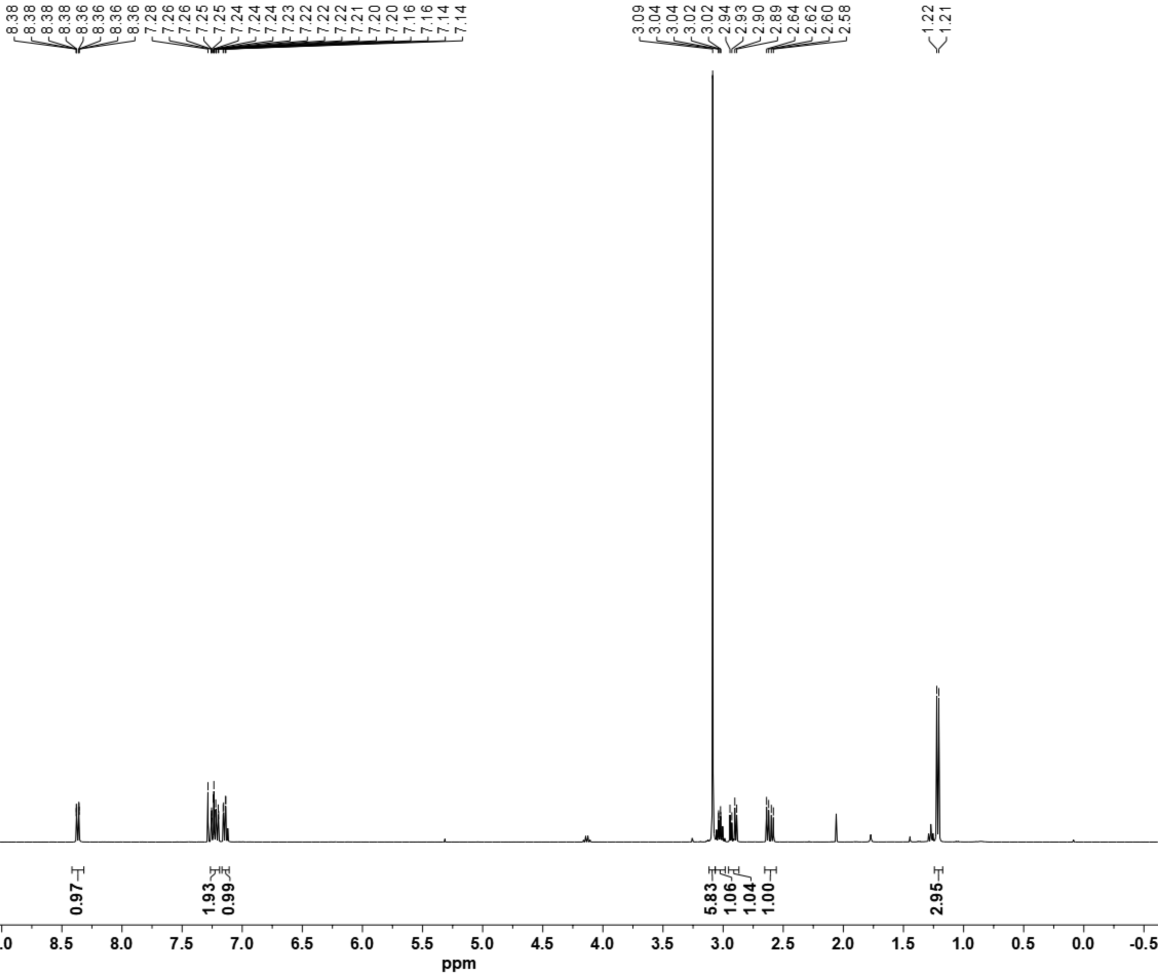

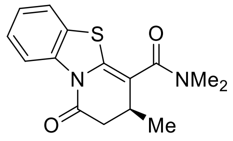

33

$^{13}C\{^{1}H\}$, $CDCl_3$, 100 MHz

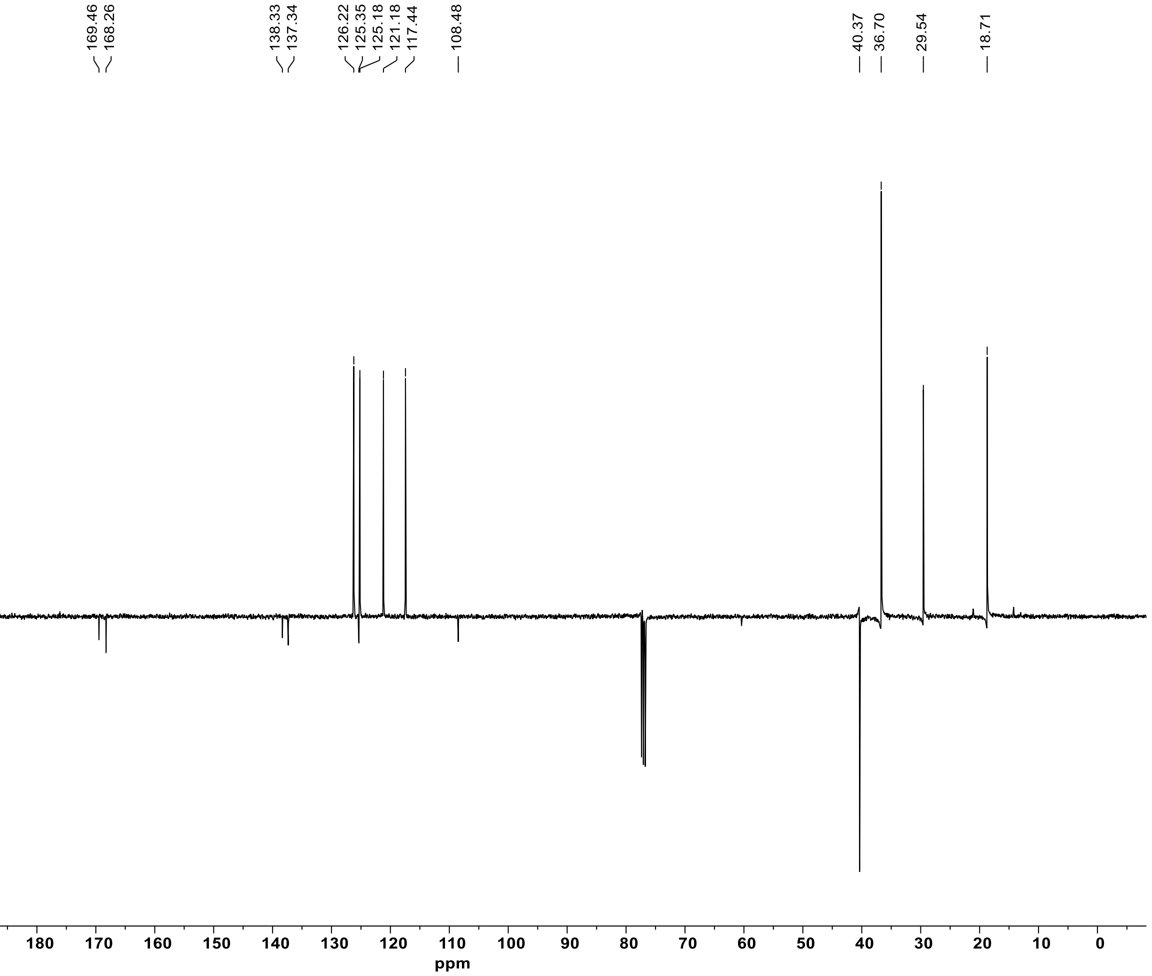

Appendix II: HPLC traces of compounds

HPLC Data for 2: ChiralPak AD-H (20% *i*-PrOH : hexane, flow rate 1 mL·min^{-1}, 254 nm, 30 °C), t_R (*S*)-**2**: 12.9 min, t_R (*R*)-**2**: 21.8 min, 94:6 er.

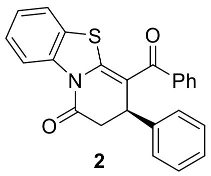

2

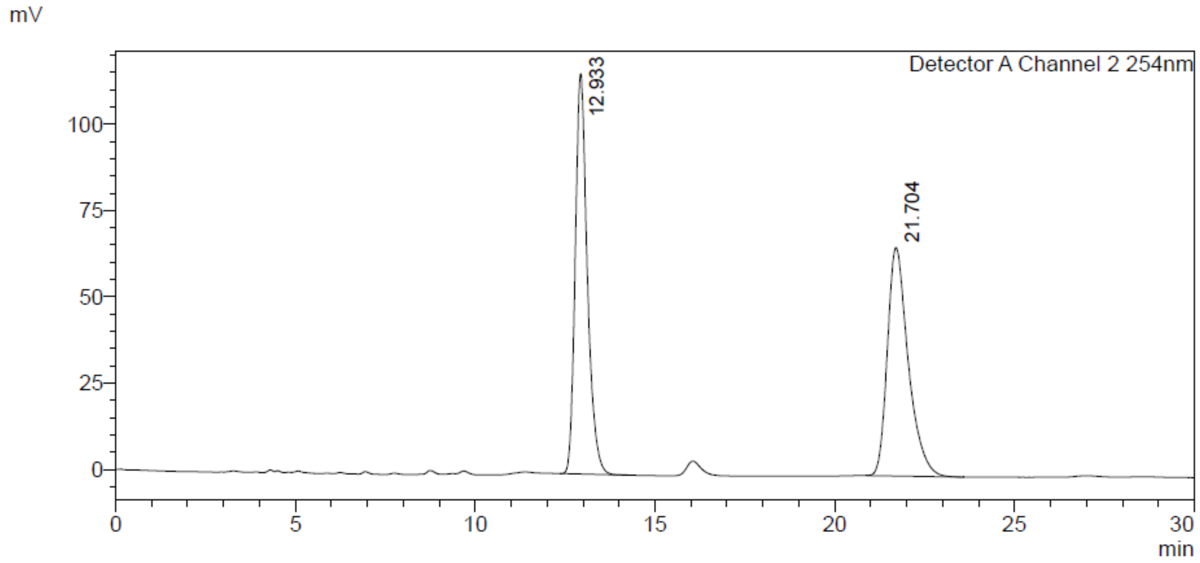

<Peak Table>

Detector A Channel 2 254nm

Peak#	Ret. Time	Area%
1	12.933	50.332
2	21.704	49.668
Total		100.000

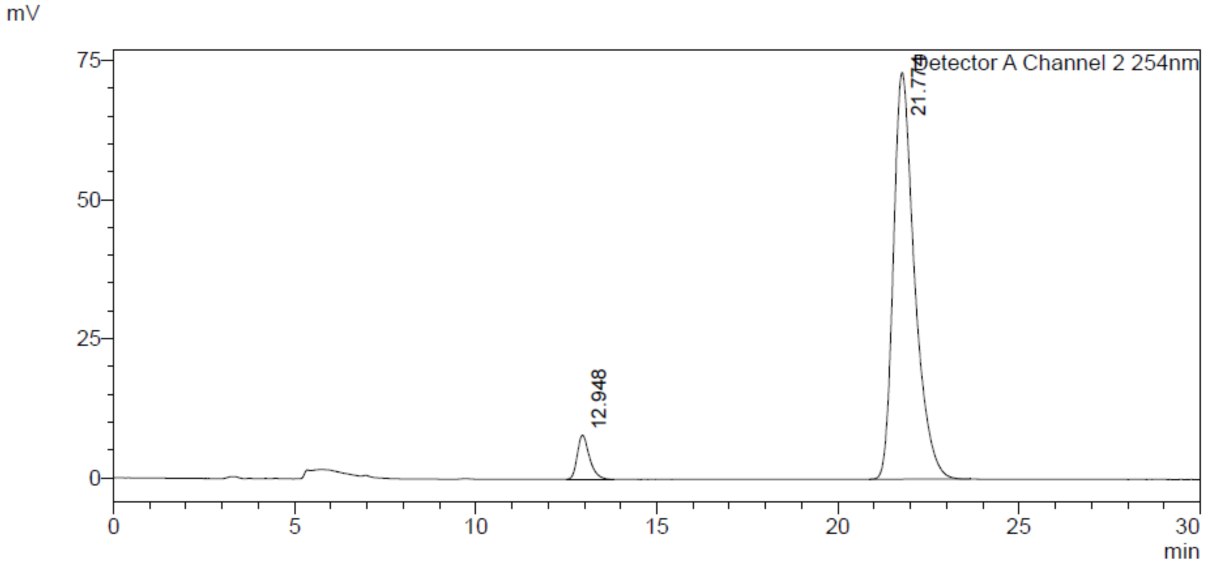

<Peak Table>

Detector A Channel 2 254nm

Peak#	Ret. Time	Area%
1	12.948	5.844
2	21.774	94.156
Total		100.000

HPLC Data for 3: ChiralPak AD-H (20% *i*-PrOH : hexane, flow rate 1 mL·min^{-1}, 254 nm, 30 °C), t_R (*S*)-**3**: 7.7 min, t_R (*R*)-**3**: 10.7 min, 91:9 er.

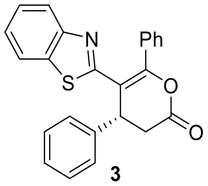

3

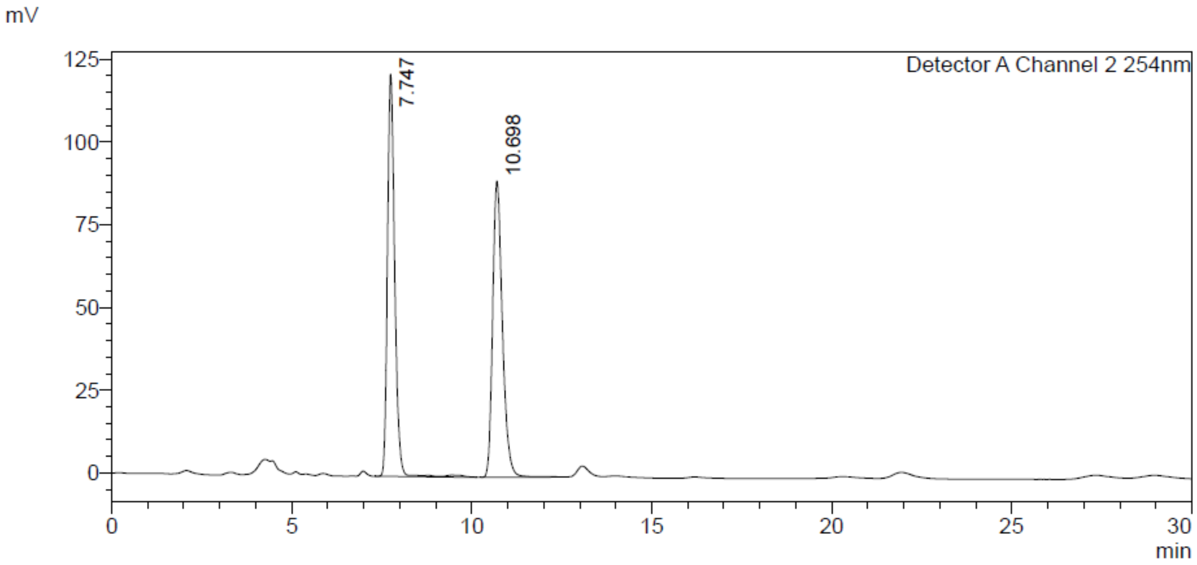

<Peak Table>

Detector A Channel 1 211nm

Peak#	Ret. Time	Area%
1	7.745	49.896
2	10.696	50.104
Total		100.000

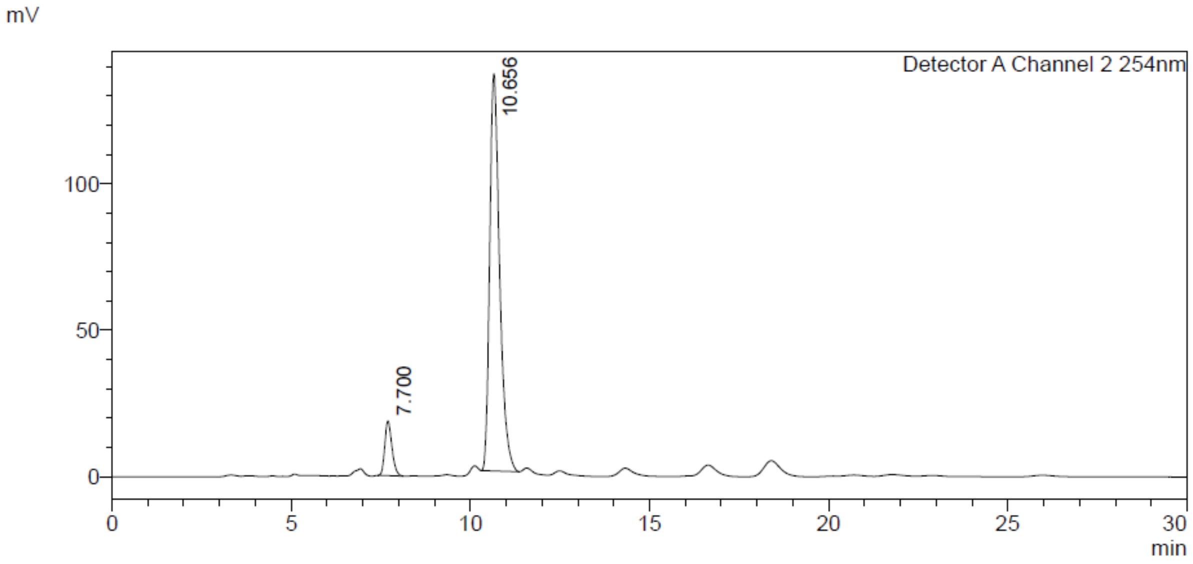

<Peak Table>

Detector A Channel 2 254nm

Peak#	Ret. Time	Area%
1	7.700	9.001
2	10.656	90.999
Total		100.000

HPLC Data for 5: ChiralPak AD-H (20% *i*-PrOH : hexane, flow rate 1 mL·min^{-1}, 254 nm, 30 °C), t_R (*S*)-**5**: 14.2 min, t_R (*R*)-**5**: 22.2 min, 94:6 er.

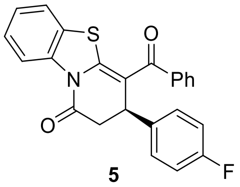

5

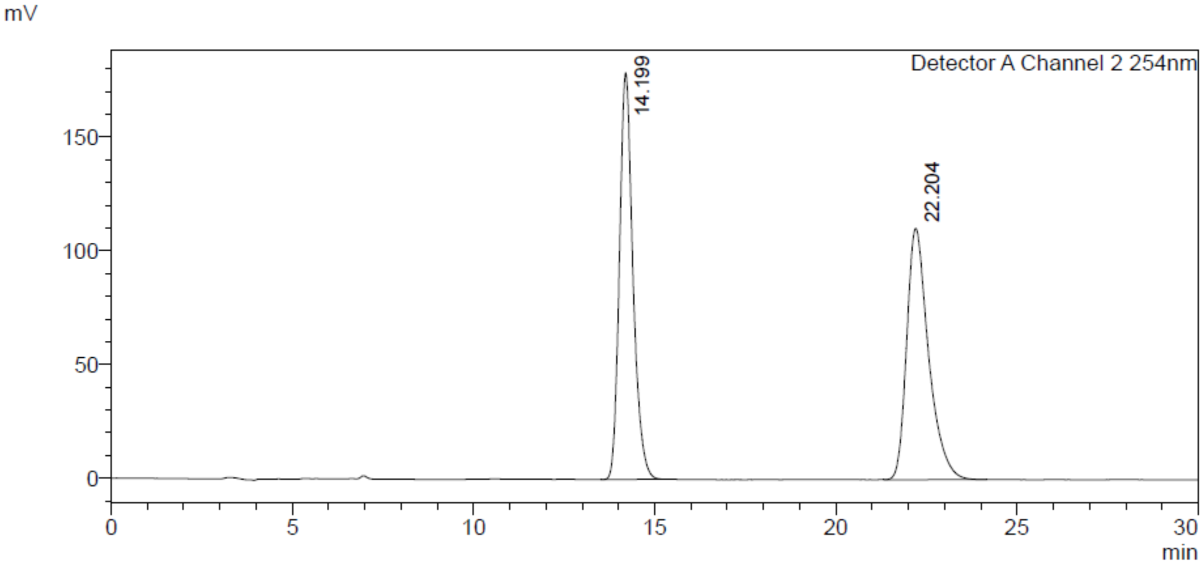

<Peak Table>

Detector A Channel 2 254nm

Peak#	Ret. Time	Area%
1	14.199	49.989
2	22.204	50.011
Total		100.000

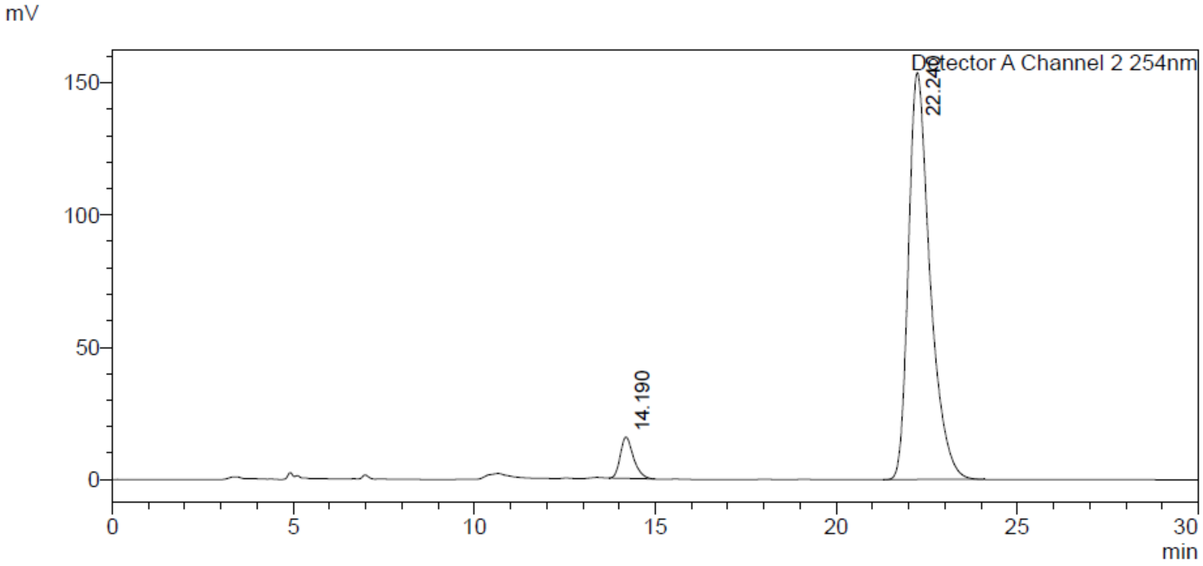

<Peak Table>

Detector A Channel 2 254nm

Peak#	Ret. Time	Area%
1	14.190	5.856
2	22.240	94.144
Total		100.000

HPLC Data for 6: ChiralPak AD-H (20% *i*-PrOH : hexane, flow rate 1 mL·min^{-1}, 254 nm, 30 °C), t_R (*S*)-**6**: 7.8 min, t_R (*R*)-**6**: 9.3 min, 91:9 er.

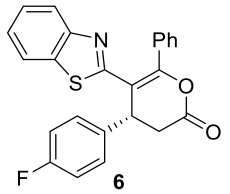

6

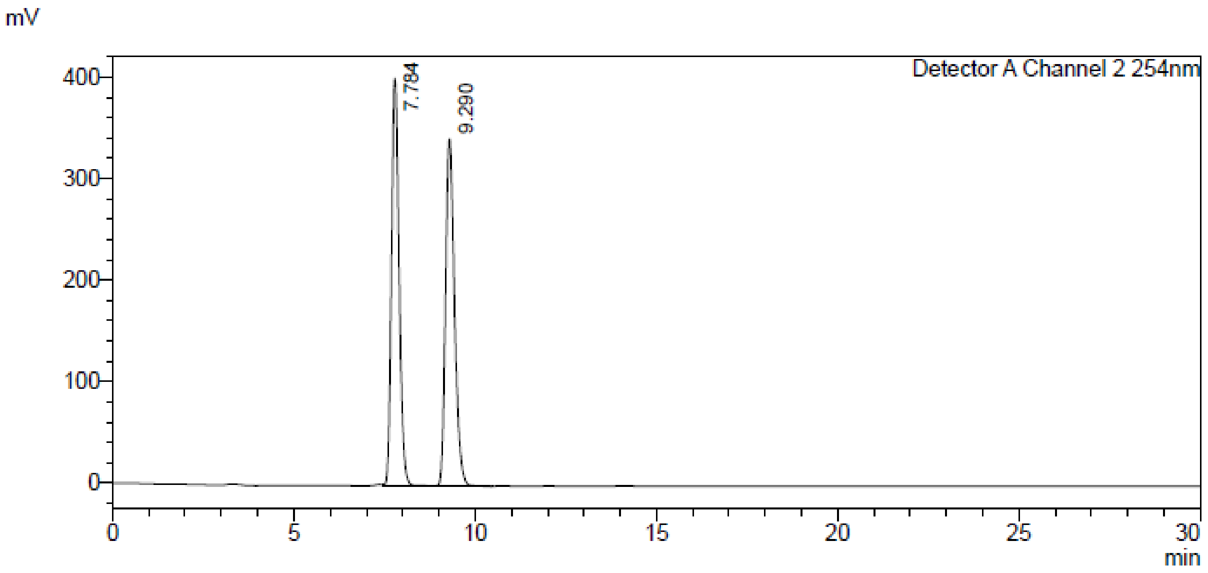

<Peak Table>

Detector A Channel 2 254nm

Peak#	Ret. Time	Area%
1	7.784	50.020
2	9.290	49.980
Total		100.000

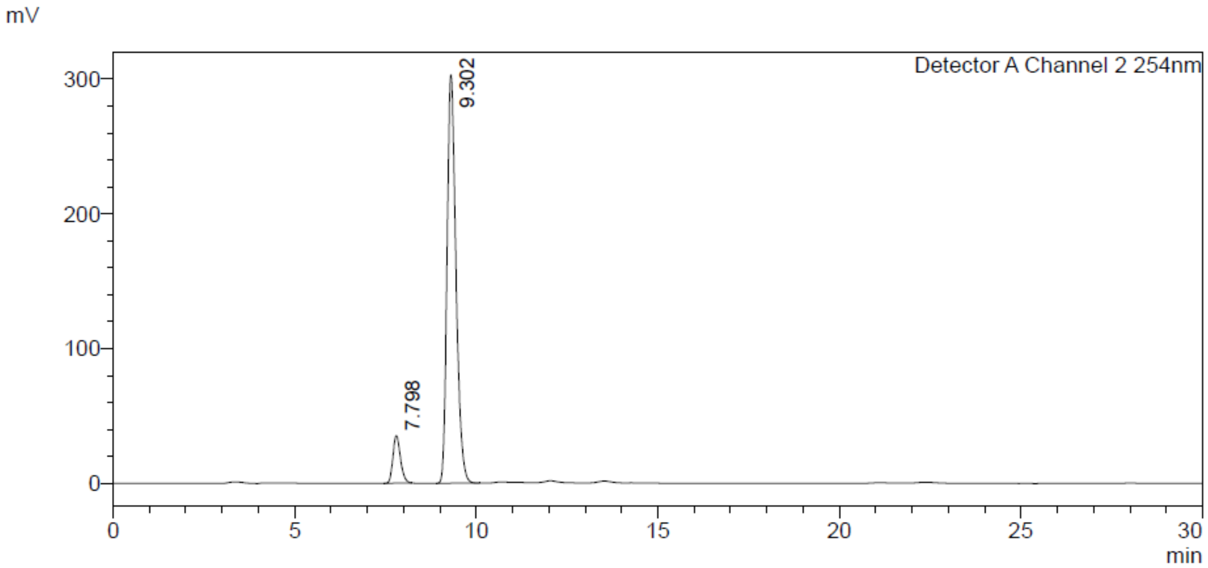

<Peak Table>

Detector A Channel 2 254nm

Peak#	Ret. Time	Area%
1	7.798	8.933
2	9.302	91.067
Total		100.000

HPLC Data for 7: ChiralPak AD-H (20% *i*-PrOH : hexane, flow rate 1 mL·min^{-1}, 254 nm, 30 °C), t_R (*S*)-**7**: 10.8 min, t_R (*R*)-**7**: 17.4 min, 94:6 er.

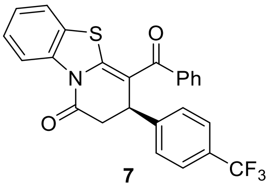

7

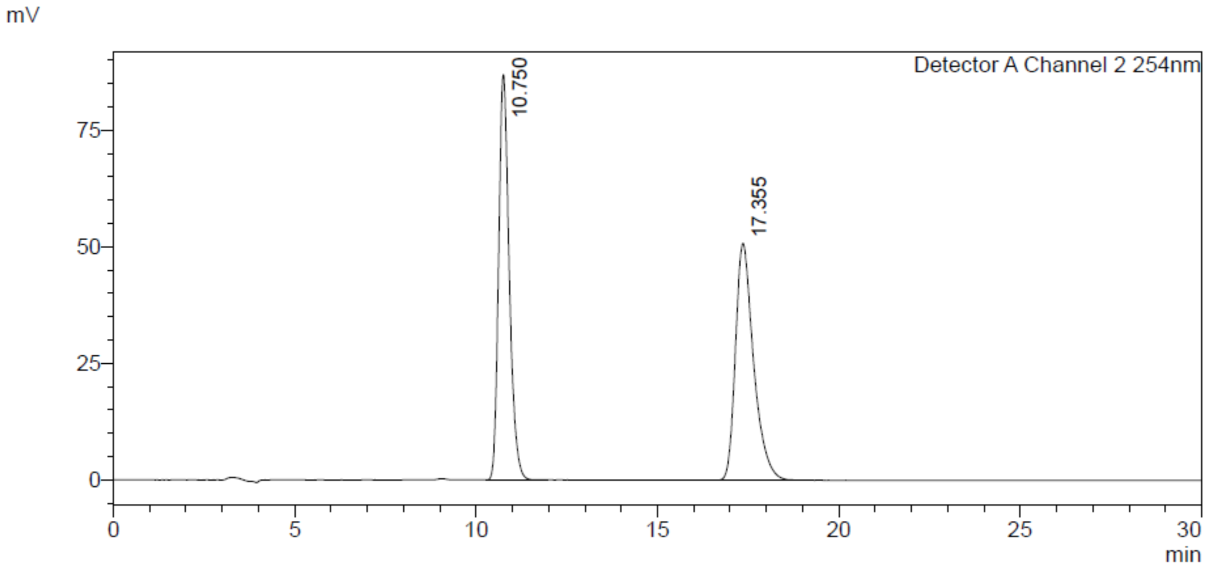

<Peak Table>

Detector A Channel 2 254nm

Peak#	Ret. Time	Area%
1	10.750	50.032
2	17.355	49.968
Total		100.000

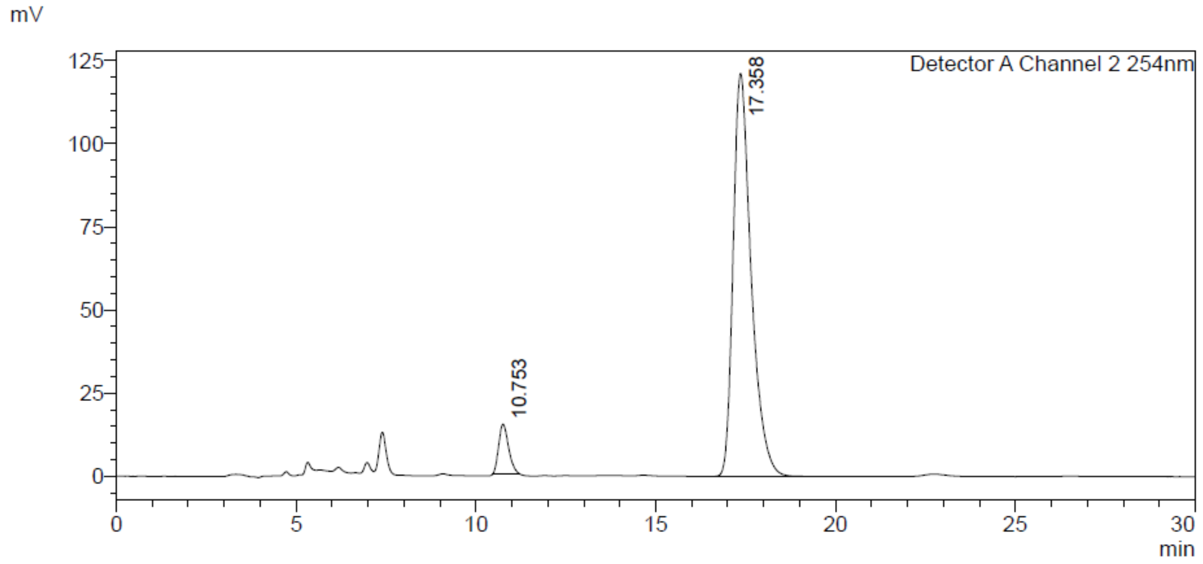

<Peak Table>

Detector A Channel 2 254nm

Peak#	Ret. Time	Area%
1	10.753	6.308
2	17.358	93.692
Total		100.000

HPLC Data for 8: ChiralPak AD-H (20% *i*-PrOH : hexane, flow rate 1 mL·min^{-1}, 254 nm, 30 °C), t_R (*S*)-**8**: 6.2 min, t_R (*R*)-**8**: 7.5 min, 93:7 er.

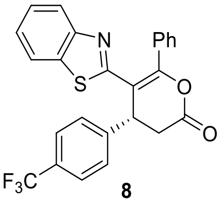

8

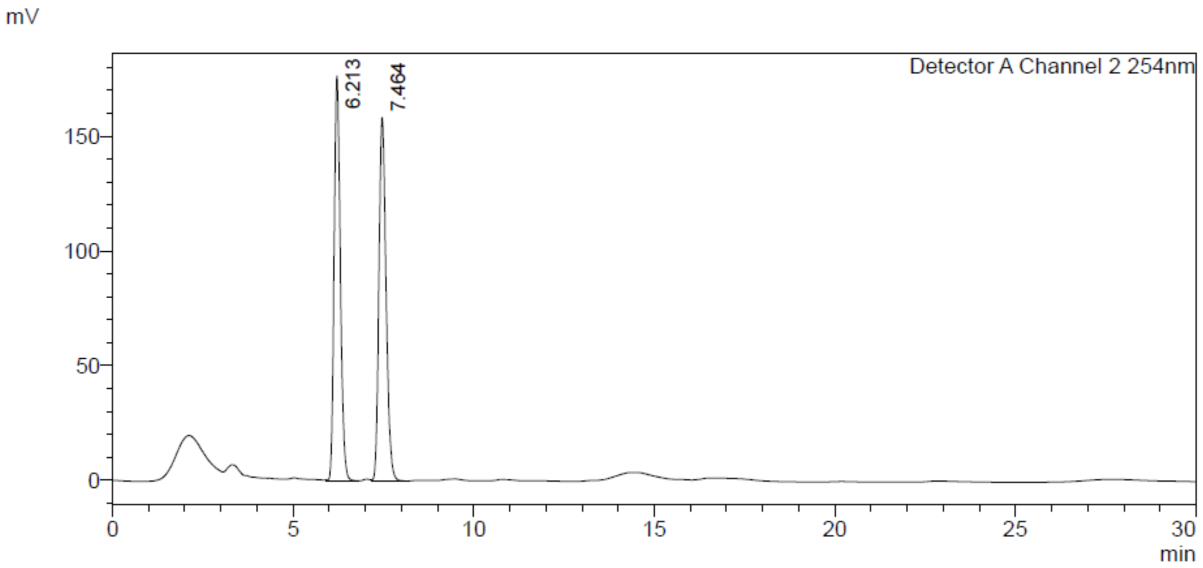

<Peak Table>

Detector A Channel 2 254nm

Peak#	Ret. Time	Area%
1	6.213	49.290
2	7.464	50.710
Total		100.000

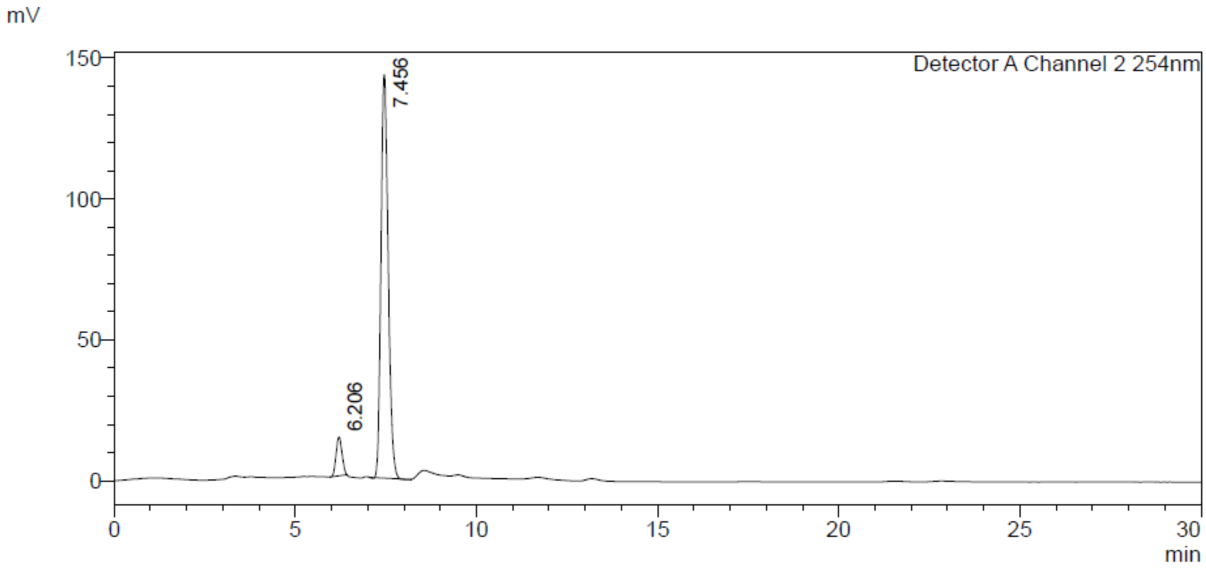

<Peak Table>

Detector A Channel 2 254nm

Peak#	Ret. Time	Area%
1	6.206	7.345
2	7.456	92.655
Total		100.000

HPLC Data for 9: ChiralPak AD-H (20% *i*-PrOH : hexane, flow rate 1 mL·min^{-1}, 254 nm, 30 °C), t_R (*S*)-**9**: 12.9 min, t_R (*R*)-**9**: 18.0 min, 79:21 er.

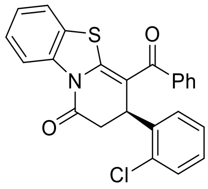

9

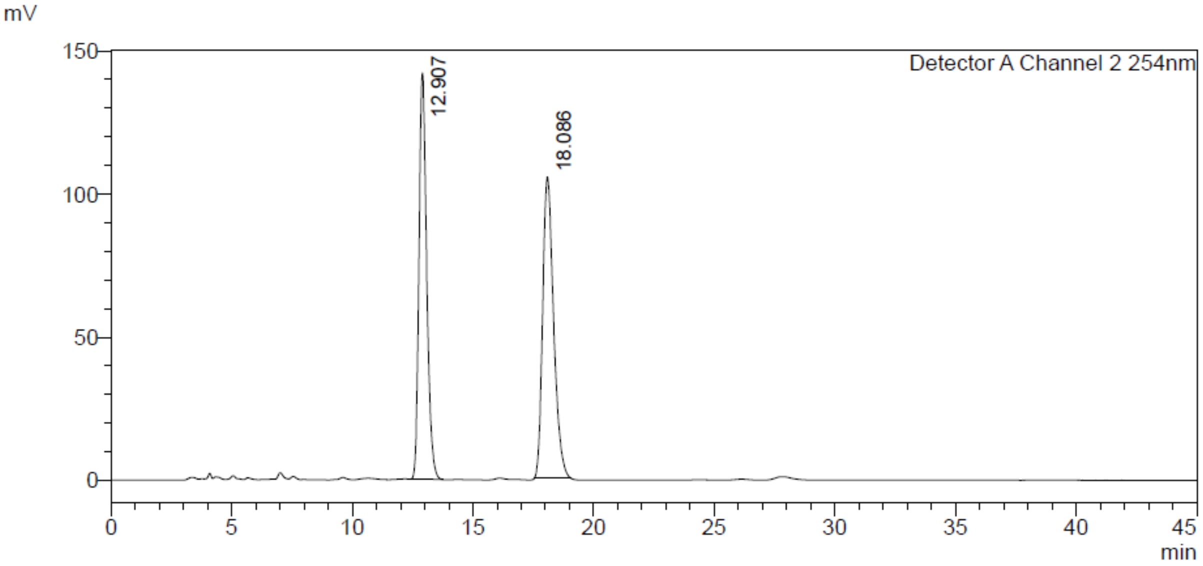

<Peak Table>

Detector A Channel 2 254nm

Peak#	Ret. Time	Area%
1	12.907	48.701
2	18.086	51.299
Total		100.000

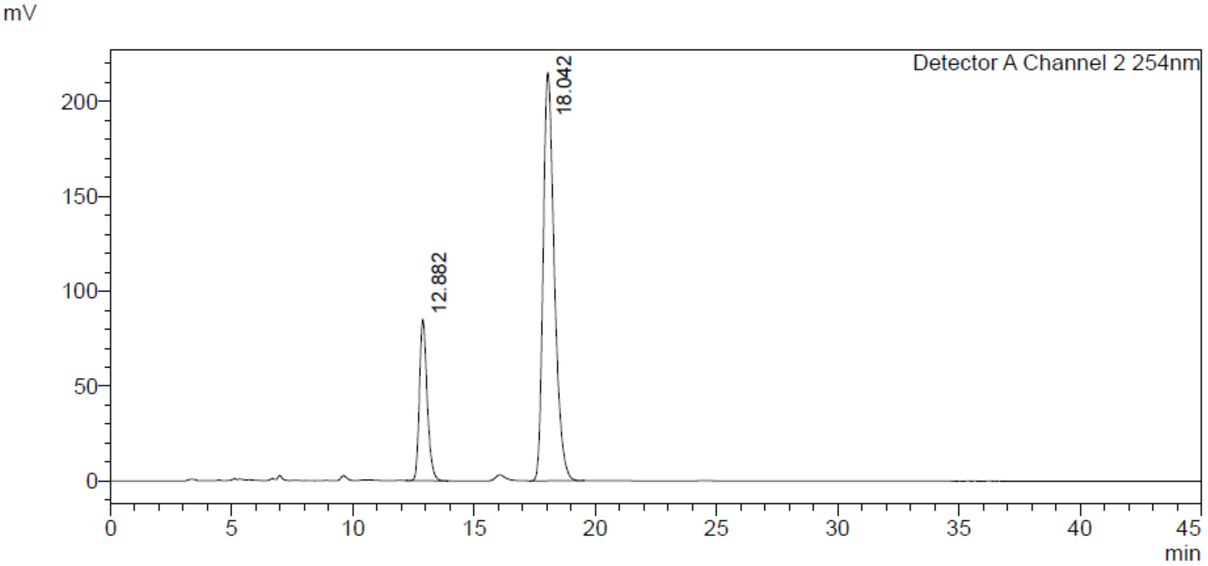

<Peak Table>

Detector A Channel 2 254nm

Peak#	Ret. Time	Area%
1	12.882	21.485
2	18.042	78.515
Total		100.000

HPLC Data for 10: ChiralPak AD-H (15% *i*-PrOH : hexane, flow rate 1 mL·min^{-1}, 254 nm, 30 °C), t_R (*S*)-**10**: 7.9 min, t_R (*R*)-**10**: 8.6 min, 62:38 er.

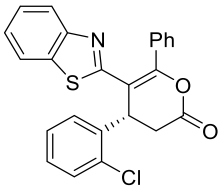

10

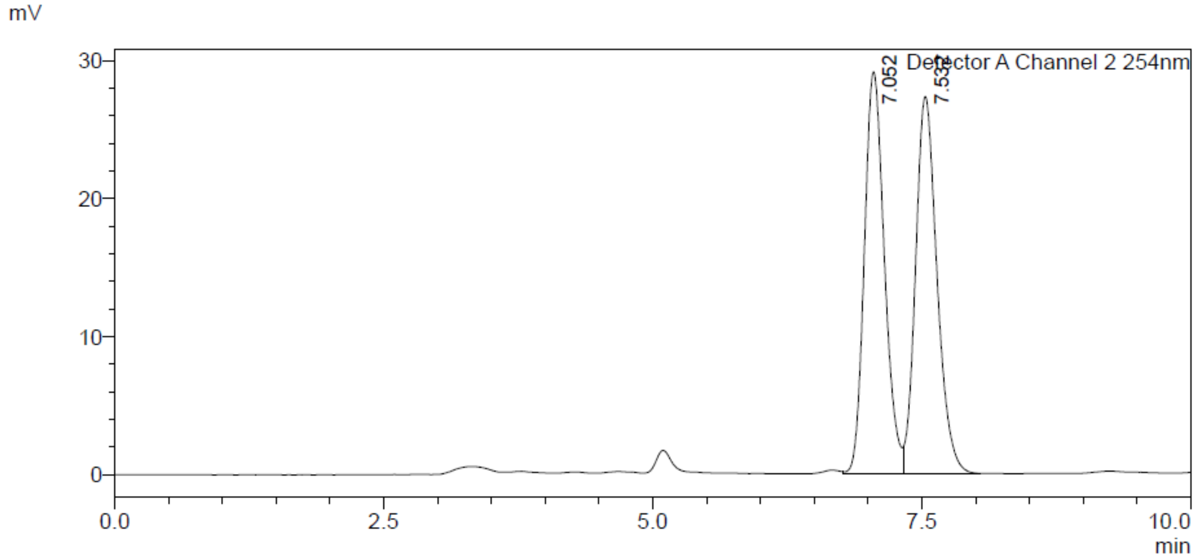

<Peak Table>

Detector A Channel 2 254nm

Peak#	Ret. Time	Area%
1	7.052	49.908
2	7.532	50.092
Total		100.000

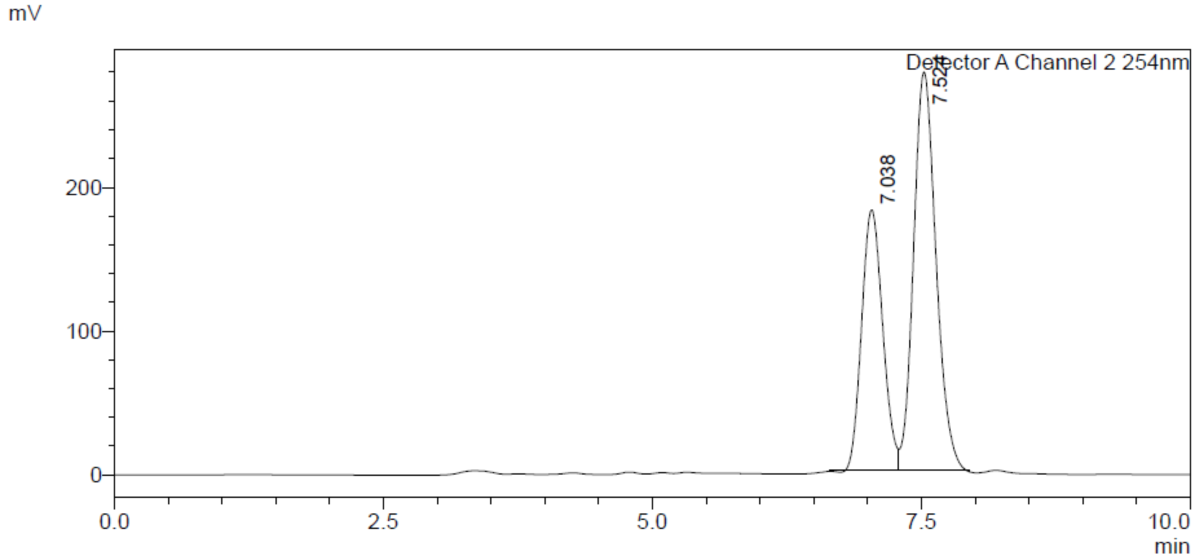

<Peak Table>

Detector A Channel 2 254nm

Peak#	Ret. Time	Area%
1	7.038	37.922
2	7.524	62.078
Total		100.000

HPLC Data for 11: ChiralPak AD-H (20% *i*-PrOH : hexane, flow rate 1 mL·min^{-1}, 254 nm, 30 °C), t_R (*S*)-**11**: 17.9 min, t_R (*R*)-**11**: 29.9 min, 90:10 er.

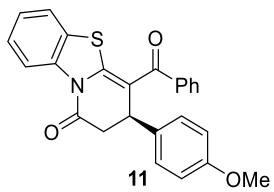

11

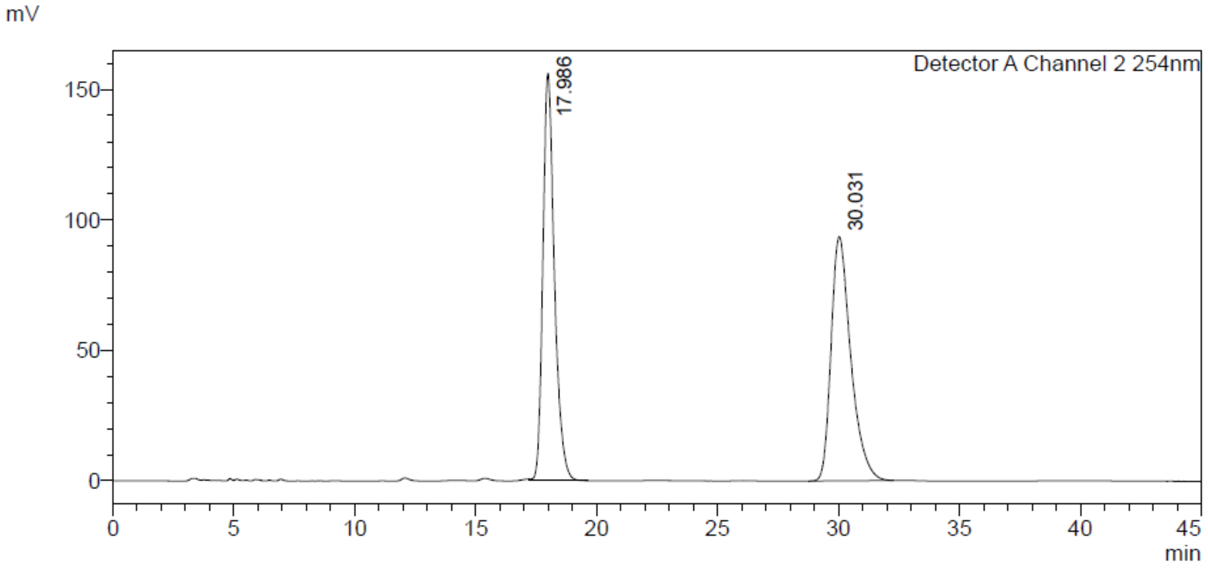

<Peak Table>

Detector A Channel 2 254nm

Peak#	Ret. Time	Area%
1	17.986	49.406
2	30.031	50.594
Total		100.000

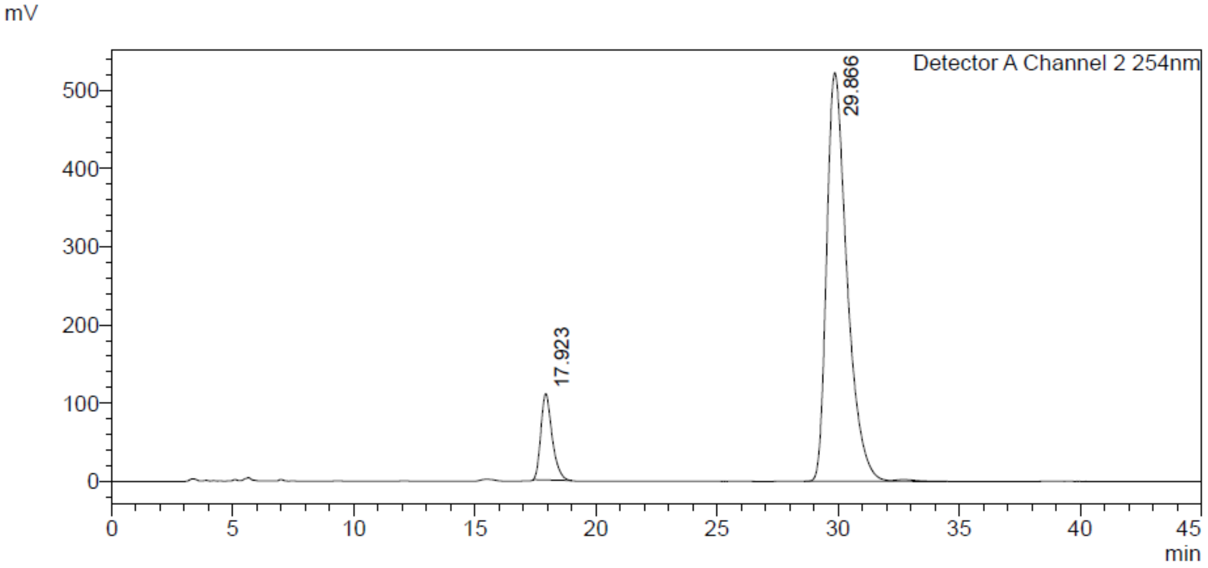

<Peak Table>

Detector A Channel 2 254nm

Peak#	Ret. Time	Area%
1	17.923	10.480
2	29.866	89.520
Total		100.000

HPLC Data for 12: ChiralPak AD-H (20% *i*-PrOH : hexane, flow rate 1 mL·min^{-1}, 254 nm, 30 °C), t_R (*S*)-**12**: 9.6 min, t_R (*R*)-**12**: 14.1 min, 88:12 er.

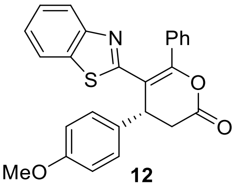

12

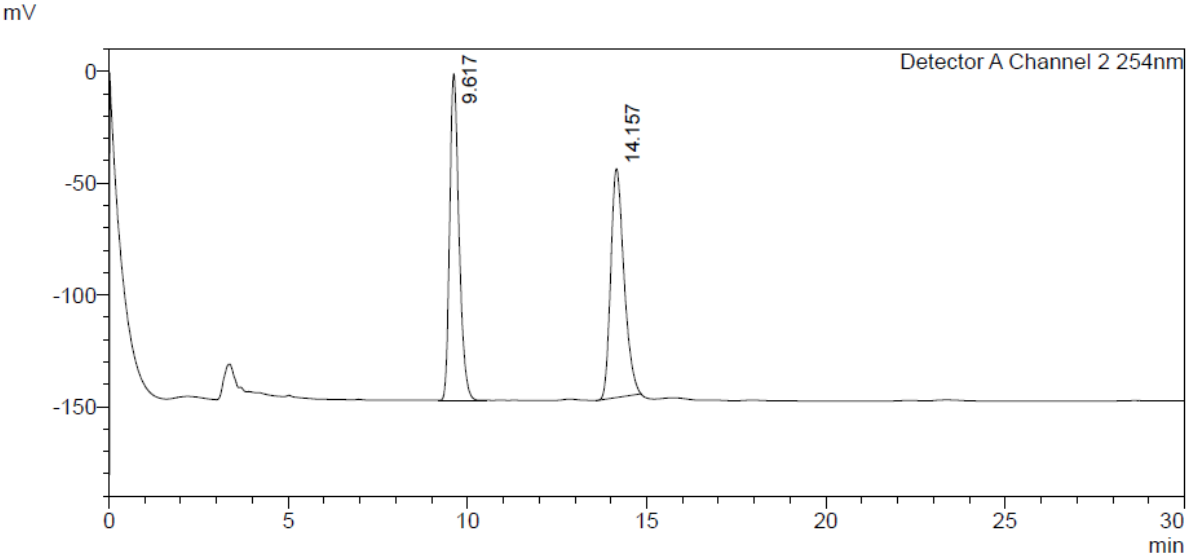

<Peak Table>

Detector A Channel 2 254nm

Peak#	Ret. Time	Area%
1	9.617	50.017
2	14.157	49.983
Total		100.000

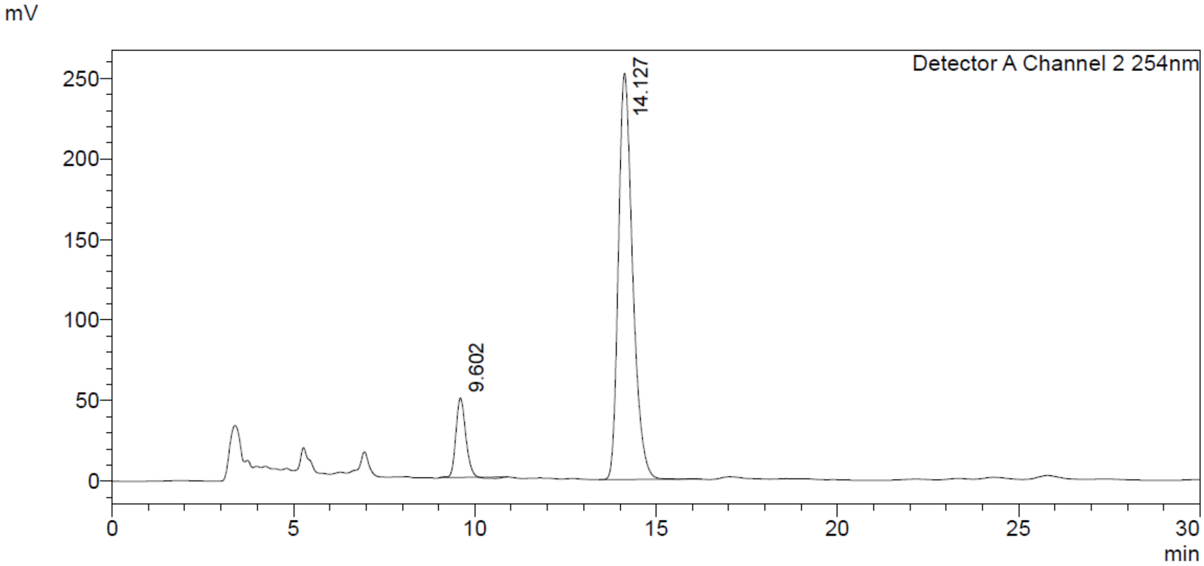

<Peak Table>

Detector A Channel 2 254nm

Peak#	Ret. Time	Area%
1	9.602	11.613
2	14.127	88.387
Total		100.000

HPLC Data for 13: ChiralPak AD-H (20% *i*-PrOH : hexane, flow rate 1 mL·min^{-1}, 254 nm, 30 °C), t_R (*S*)-**13**: 11.7 min, t_R (*R*)-**13**: 23.1 min, 93:7 er.

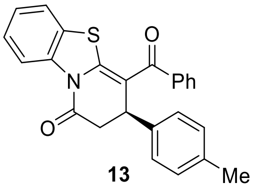

13

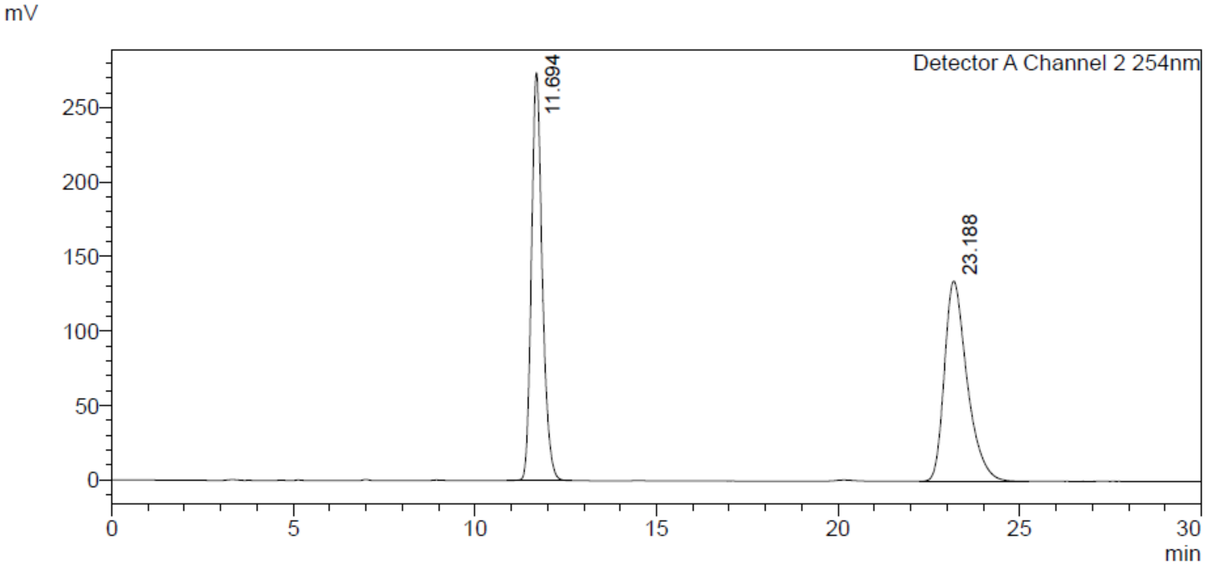

<Peak Table>

Detector A Channel 2 254nm

Peak#	Ret. Time	Area%
1	11.694	49.340
2	23.188	50.660
Total		100.000

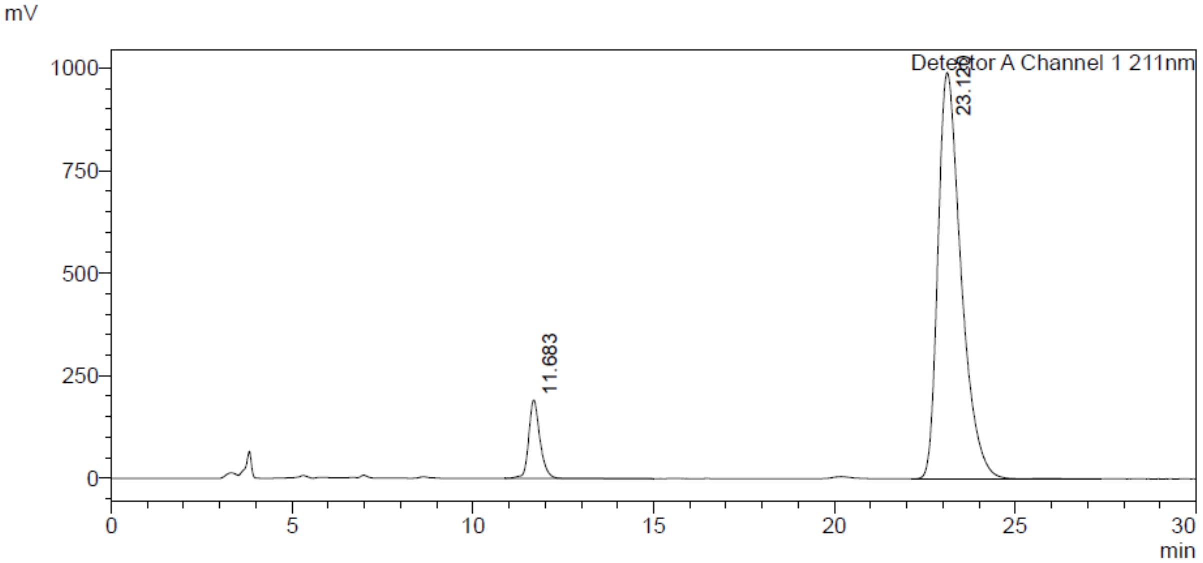

<Peak Table>

Detector A Channel 2 254nm

Peak#	Ret. Time	Area%
1	11.685	7.003
2	23.122	92.997
Total		100.000

HPLC Data for 14: ChiralPak AD-H (20% *i*-PrOH : hexane, flow rate 1 mL·min^{-1}, 254 nm, 30 °C), t_R (*S*)-**14**: 7.7 min, t_R (*R*)-**14**: 10.5 min, 89:11 er.

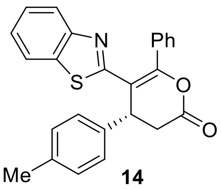

14

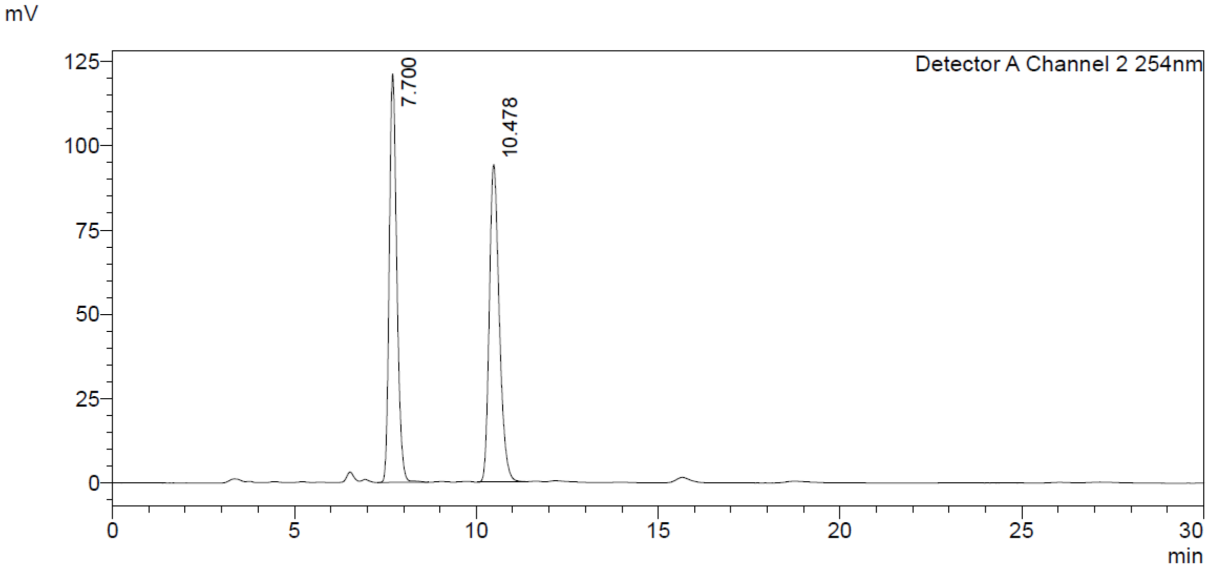

<Peak Table>

Detector A Channel 2 254nm

Peak#	Ret. Time	Area%
1	7.700	49.217
2	10.478	50.783
Total		100.000

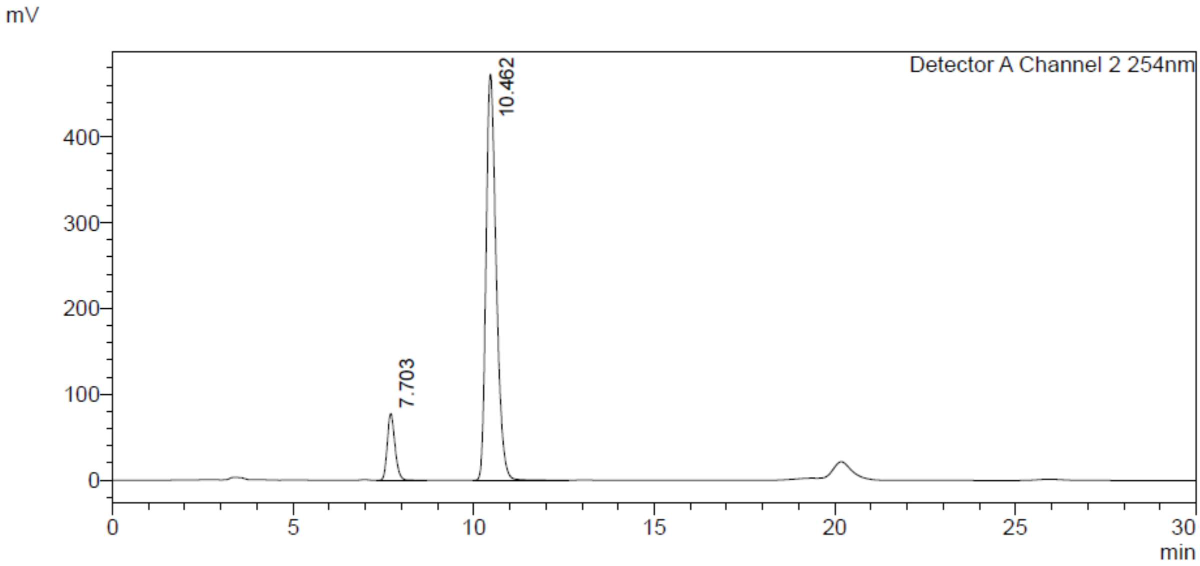

<Peak Table>

Detector A Channel 2 254nm

Peak#	Ret. Time	Area%
1	7.703	11.027
2	10.462	88.973
Total		100.000

HPLC Data for 15: ChiralPak AD-H (20% *i*-PrOH : hexane, flow rate 1 mL·min^{-1}, 254 nm, 30 °C), t_R (*S*)-**15**: 10.7 min, t_R (*R*)-**15**: 15.8 min, 96:4 er.

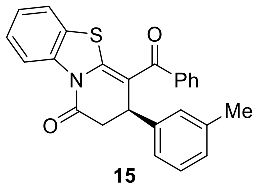

15

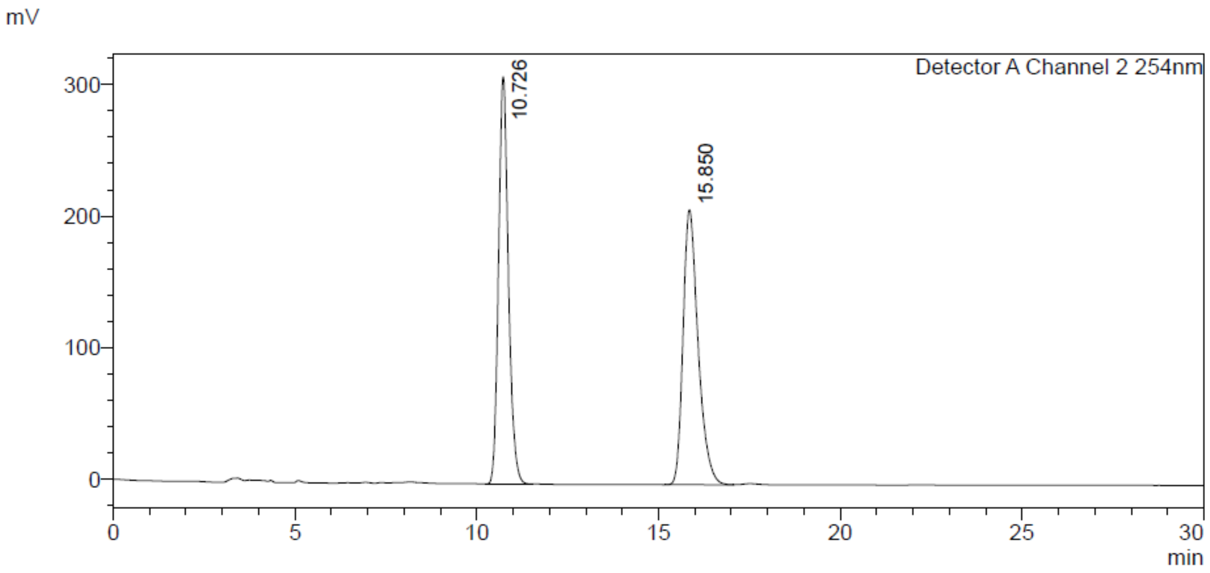

<Peak Table>

Detector A Channel 2 254nm

Peak#	Ret. Time	Area%
1	10.726	49.354
2	15.850	50.646
Total		100.000

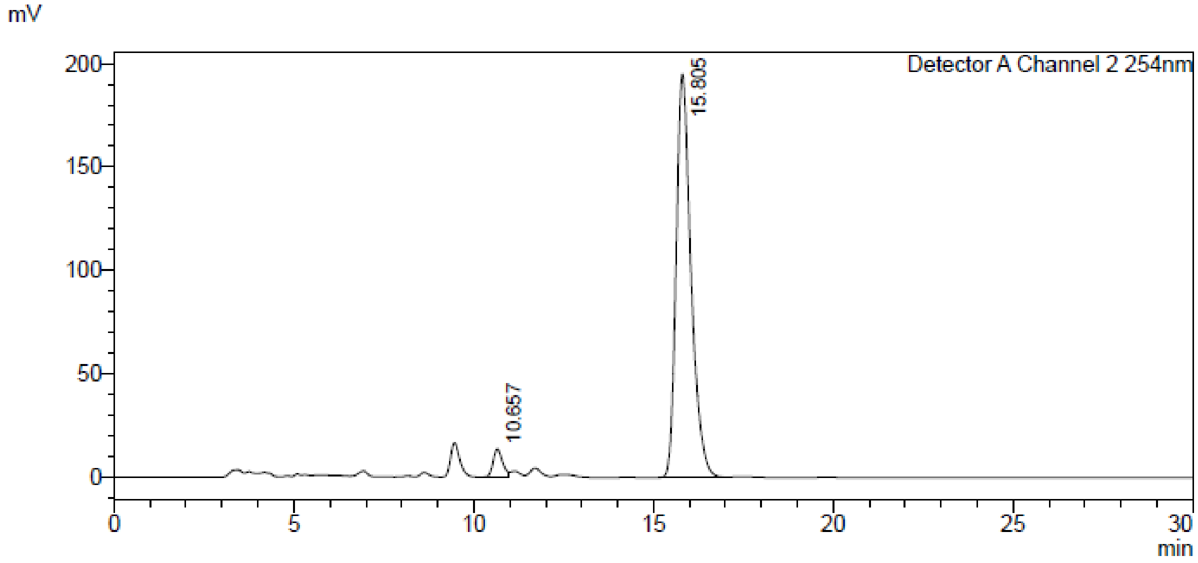

<Peak Table>

Detector A Channel 2 254nm

Peak#	Ret. Time	Area%
1	10.657	4.359
2	15.805	95.641
Total		100.000

HPLC Data for 16: ChiralPak AD-H (20% *i*-PrOH : hexane, flow rate 1 mL·min^{-1}, 254 nm, 30 °C), t_R (*S*)-**16**: 6.8 min, t_R (*R*)-**16**: 9.4 min, 92:8 er.

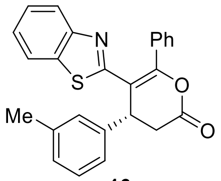

16

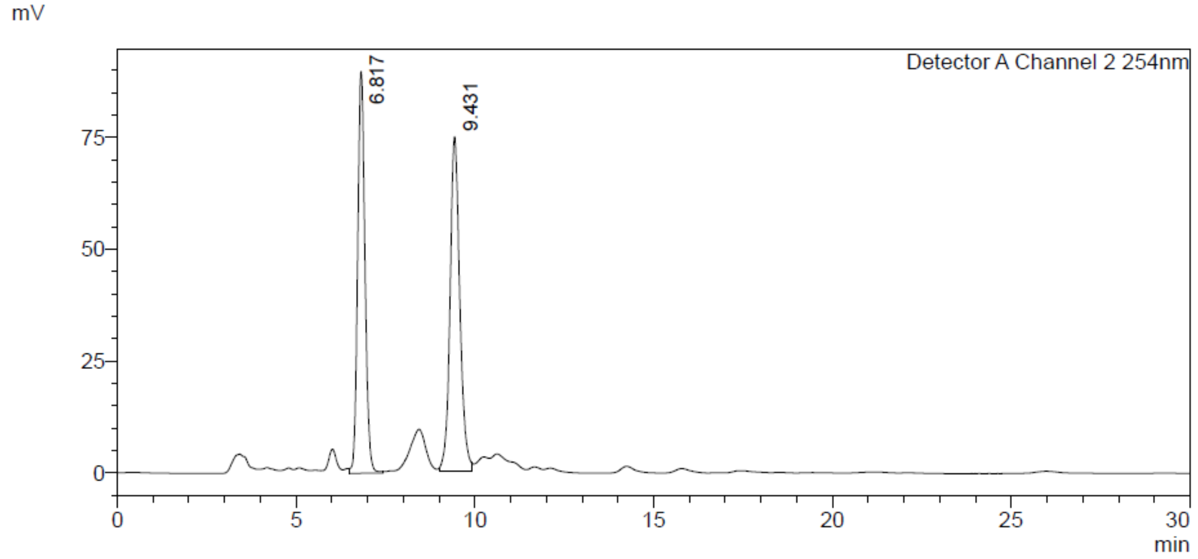

<Peak Table>

Detector A Channel 2 254nm

Peak#	Ret. Time	Area%
1	6.817	47.534
2	9.431	52.466
Total		100.000

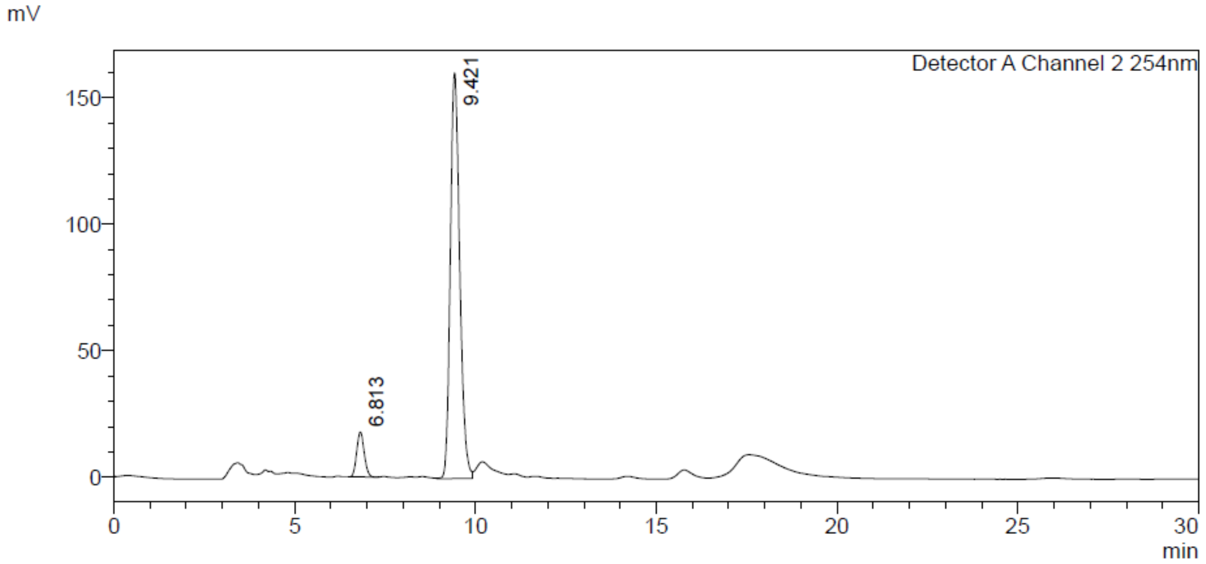

<Peak Table>

Detector A Channel 2 254nm

Peak#	Ret. Time	Area%
1	6.813	8.121
2	9.421	91.879
Total		100.000

HPLC Data for 17: ChiralPak AD-H (20% *i*-PrOH : hexane, flow rate 1 mL·min^{-1}, 254 nm, 30 °C), t_R (*S*)-**17**: 6.8 min 16.2 min, t_R (*R*)-**17**: 25.6 min, 96:4 er.

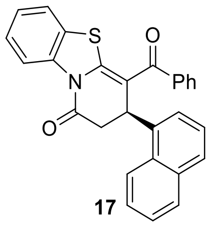

17

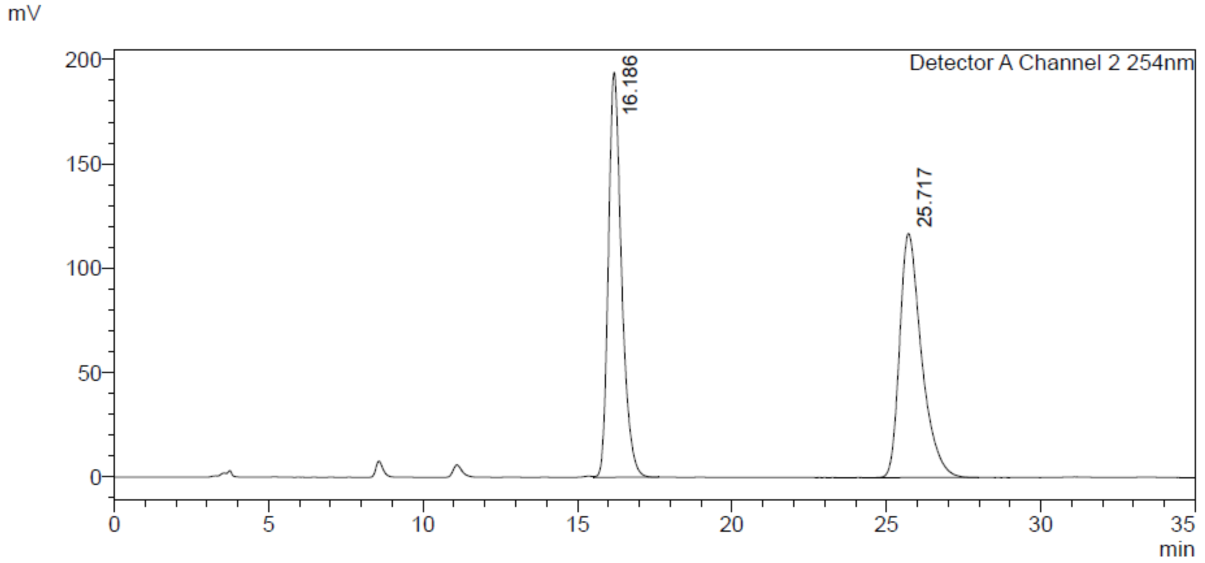

<Peak Table>

Detector A Channel 2 254nm

Peak#	Ret. Time	Area%
1	16.186	49.985
2	25.717	50.015
Total		100.000

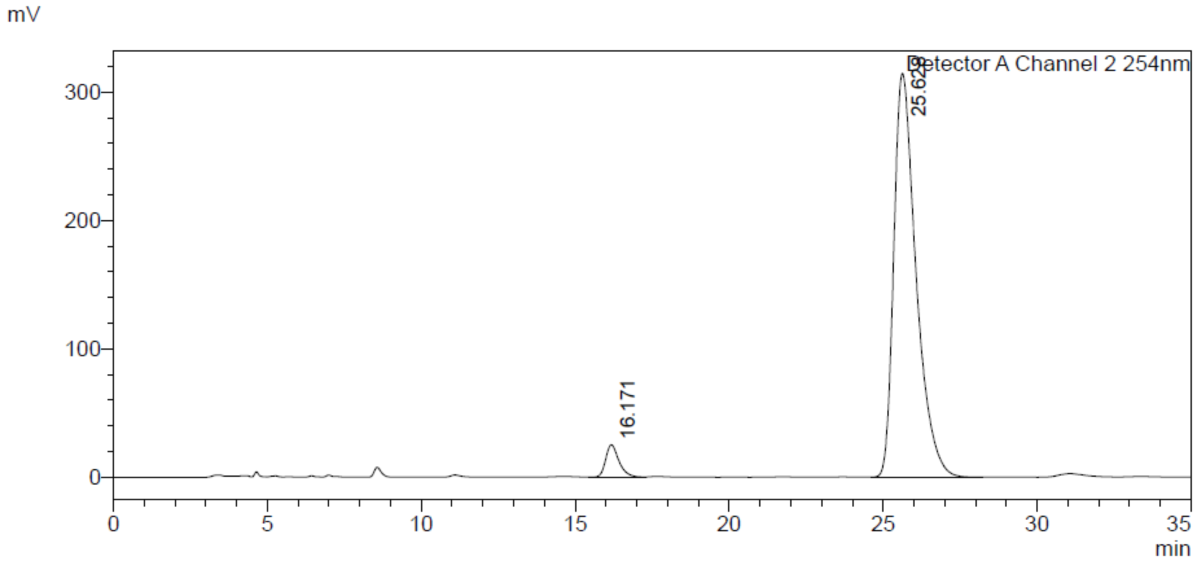

<Peak Table>

Detector A Channel 2 254nm

Peak#	Ret. Time	Area%
1	16.171	4.480
2	25.628	95.520
Total		100.000

HPLC Data for 18: ChiralPak AD-H (20% i-PrOH : hexane, flow rate 1 mL min^{-1}, 254 nm, 30 °C), t_R (*R*)-**18**: 8.5 min, t_R (*S*)-**18**: 11.1 min, 80:20 er.

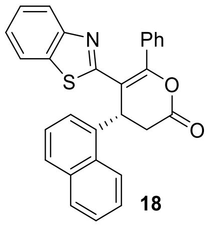

18

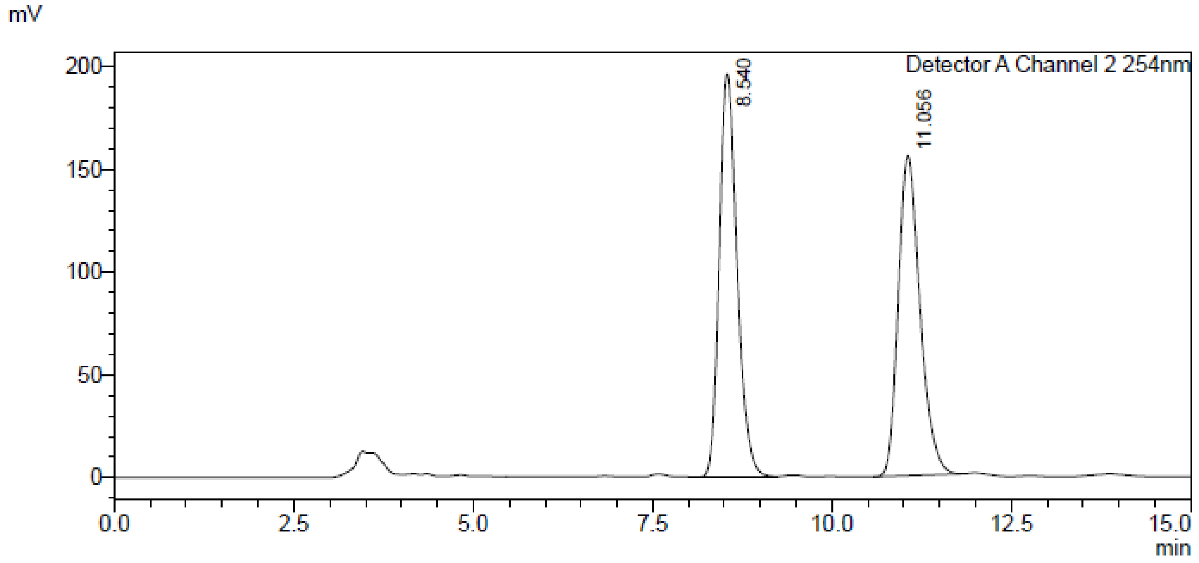

<Peak Table>

Detector A Channel 2 254nm

Peak#	Ret. Time	Area%
1	8.540	50.737
2	11.056	49.263
Total		100.000

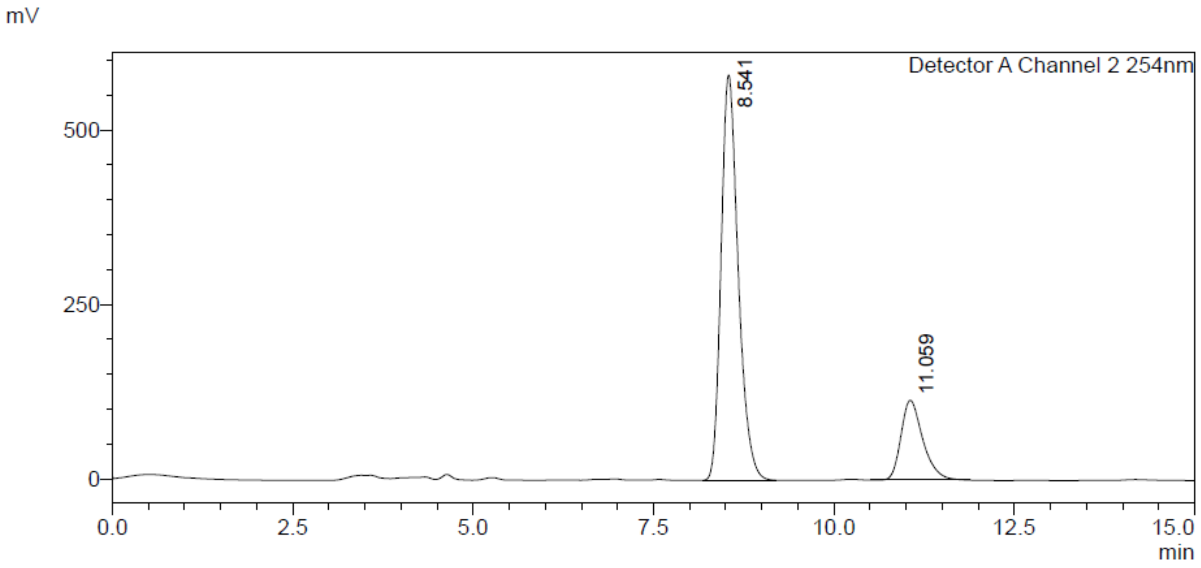

<Peak Table>

Detector A Channel 2 254nm

Peak#	Ret. Time	Area%
1	8.541	79.884
2	11.059	20.116
Total		100.000

HPLC Data for 19: ChiralPak AD-H (20% *i*-PrOH : hexane, flow rate 1 mL·min^{-1}, 254 nm, 30 °C), t_R (*R*)-**19**: 14.9 min, t_R (*S*)-**19**: 19.7 min, 93:7 er.

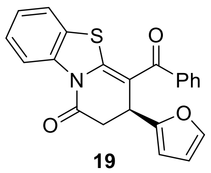

19

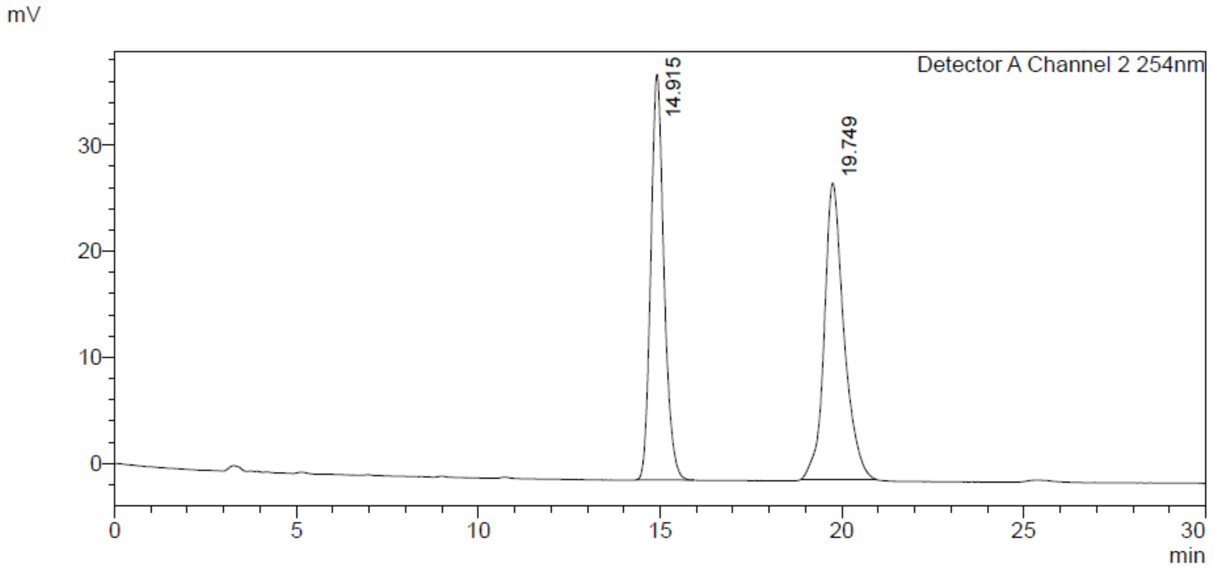

<Peak Table>

Detector A Channel 2 254nm

Peak#	Ret. Time	Area%
1	14.915	47.909
2	19.749	52.091
Total		100.000

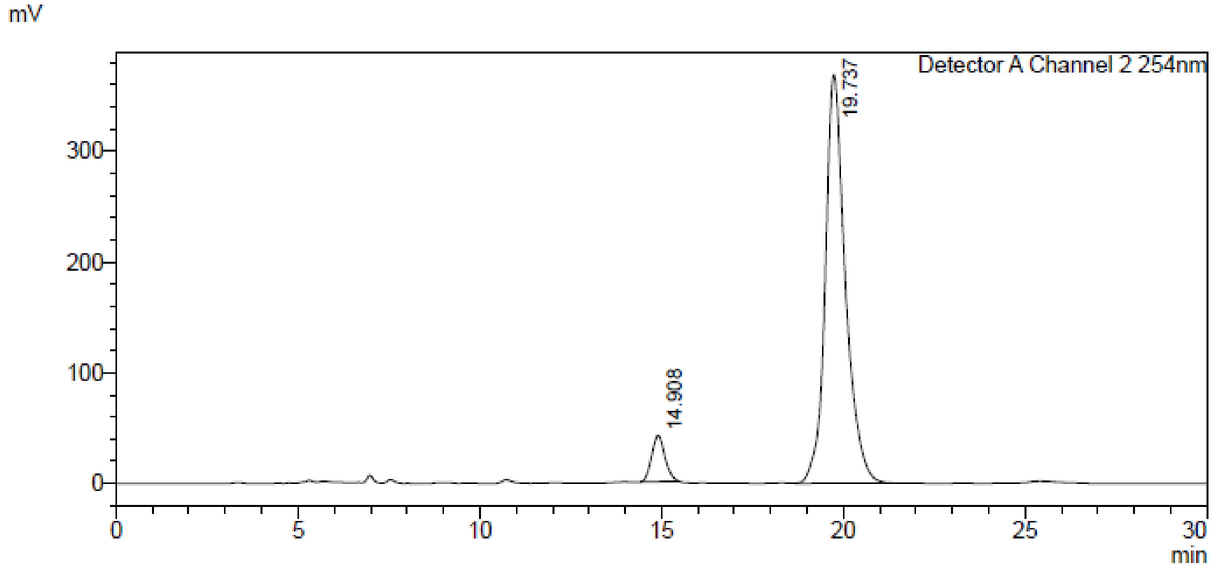

<Peak Table>

Detector A Channel 2 254nm

Peak#	Ret. Time	Area%
1	14.908	6.939
2	19.737	93.061
Total		100.000

HPLC Data for 20: ChiralPak AD-H (20% *i*-PrOH : hexane, flow rate 1 mL·min^{-1}, 254 nm, 30 °C), t_R (*R*)-**20**: 7.6 min, t_R (*S*)-**20**: 14.8 min, 79:21 er.

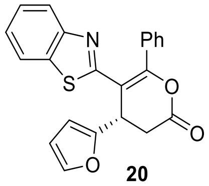

20

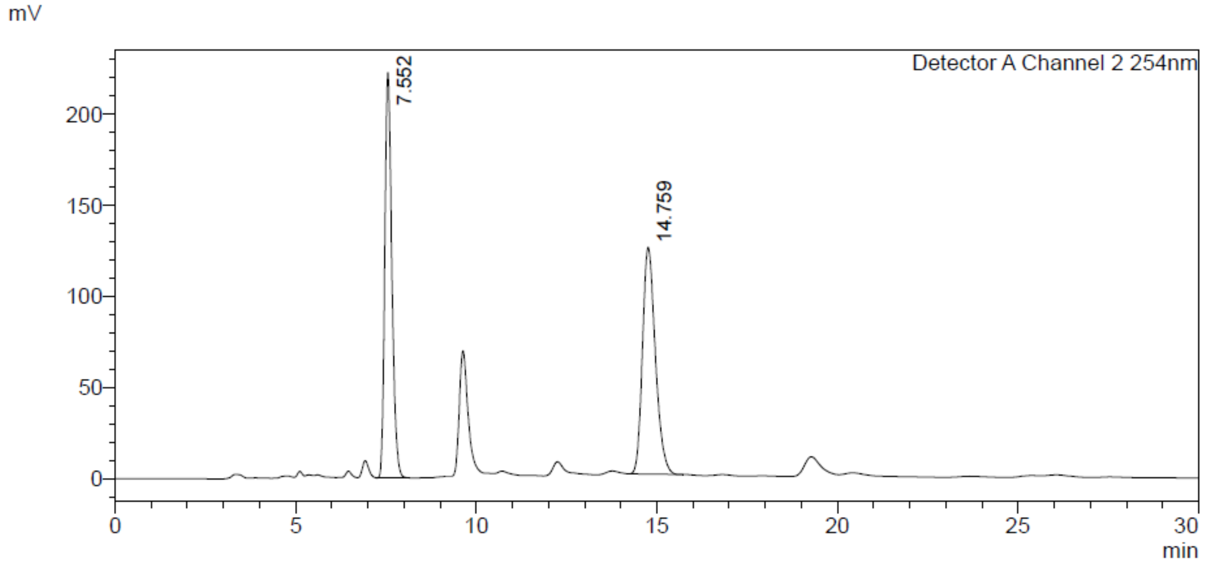

<Peak Table>

Detector A Channel 2 254nm

Peak#	Ret. Time	Area%
1	7.552	49.461
2	14.759	50.539
Total		100.000

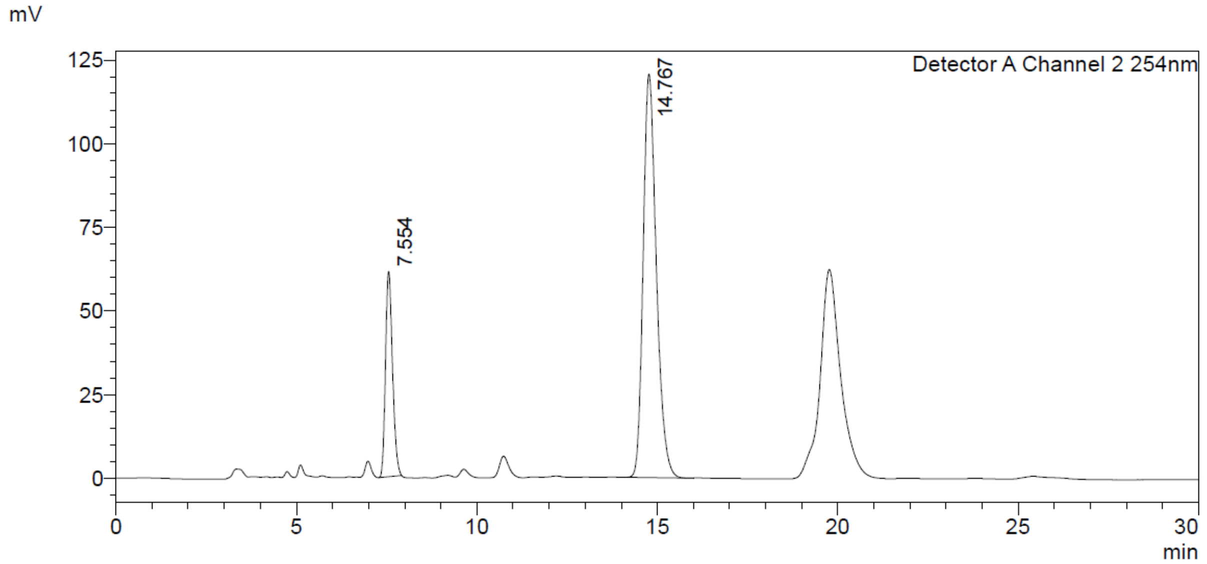

<Peak Table>

Detector A Channel 2 254nm

Peak#	Ret. Time	Area%
1	7.554	21.244
2	14.767	78.756
Total		100.000

HPLC Data for 21: ChiralPak AD-H (20% *i*-PrOH : hexane, flow rate 1 mL·min^{-1}, 254 nm, 30 °C), t_R (*R*)-**21**: 18.0 min, t_R (*S*)-**21**: 28.2 min, 97:3 er.

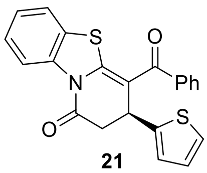

21

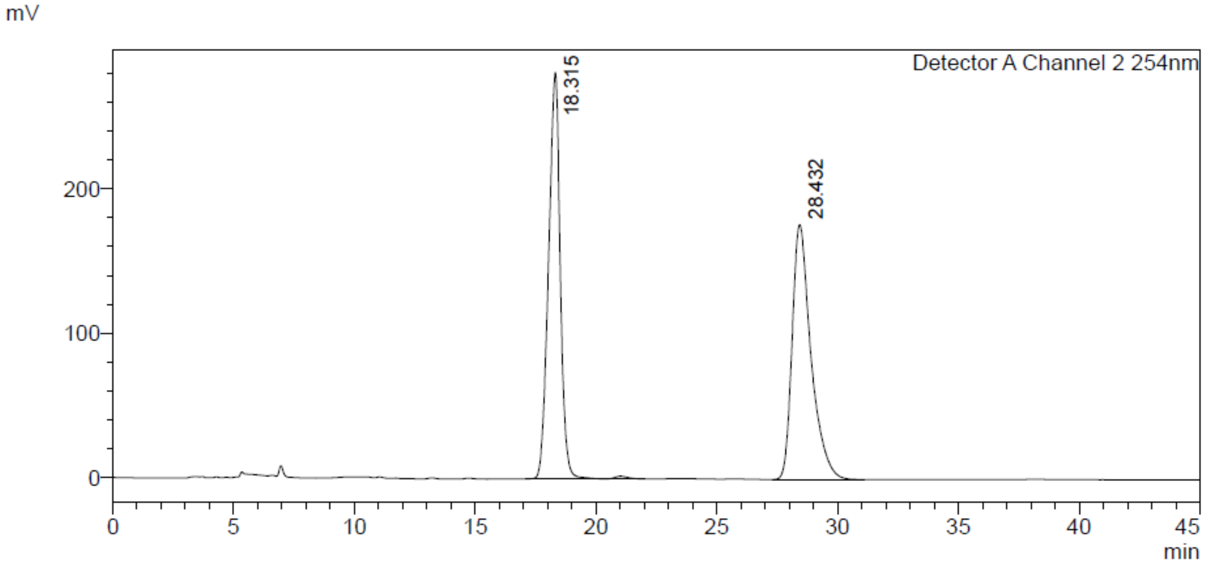

<Peak Table>

Detector A Channel 2 254nm

Peak#	Ret. Time	Area%
1	18.315	49.756
2	28.432	50.244
Total		100.000

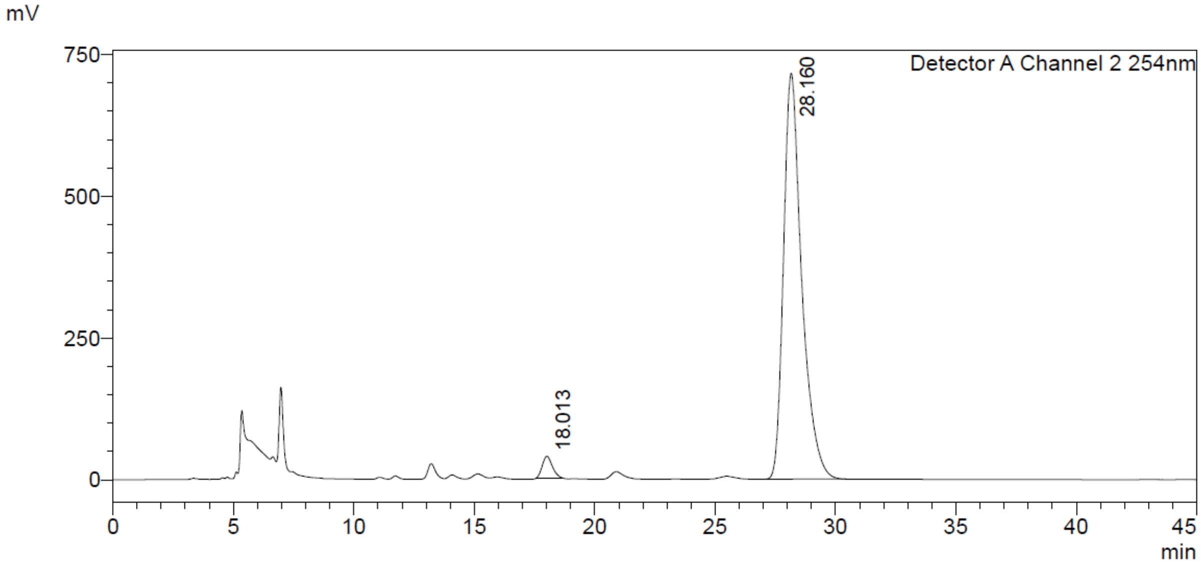

<Peak Table>

Detector A Channel 2 254nm

Peak#	Ret. Time	Area%
1	18.013	2.815
2	28.160	97.185
Total		100.000

HPLC Data for 22: ChiralPak AD-H (20% *i*-PrOH : hexane, flow rate 1 mL·min^{-1}, 254 nm, 30 °C), t_R (*R*)-**22**: 8.6 min, t_R (*S*)-**22**: 17.9 min, 87:13 er.

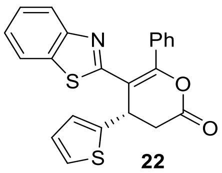

22

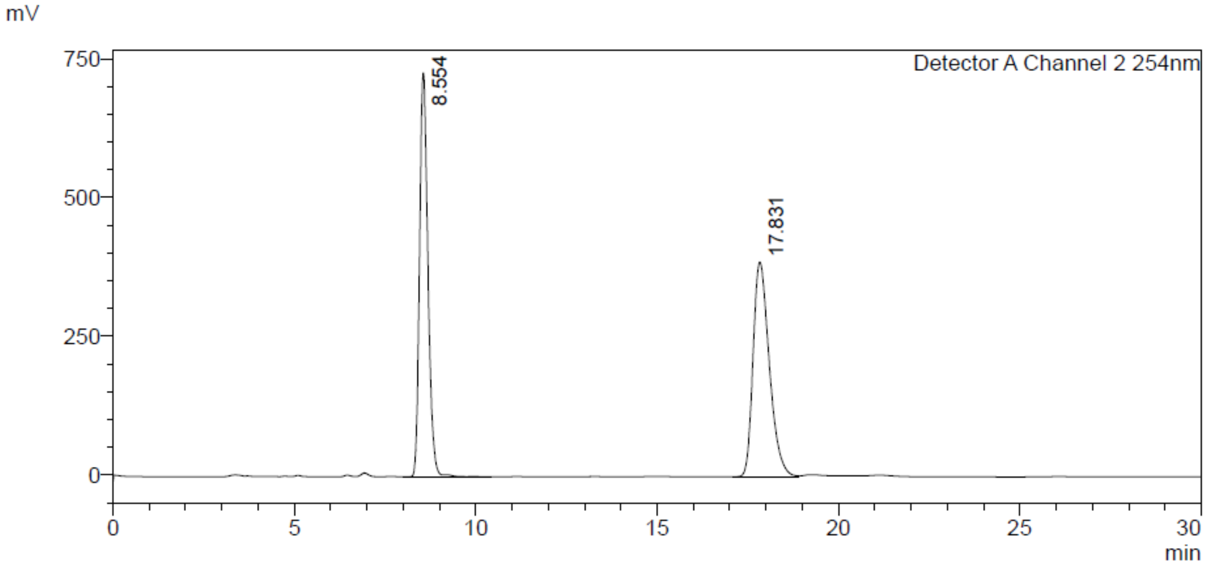

<Peak Table>

Detector A Channel 2 254nm

Peak#	Ret. Time	Area%
1	8.554	49.208
2	17.831	50.792
Total		100.000

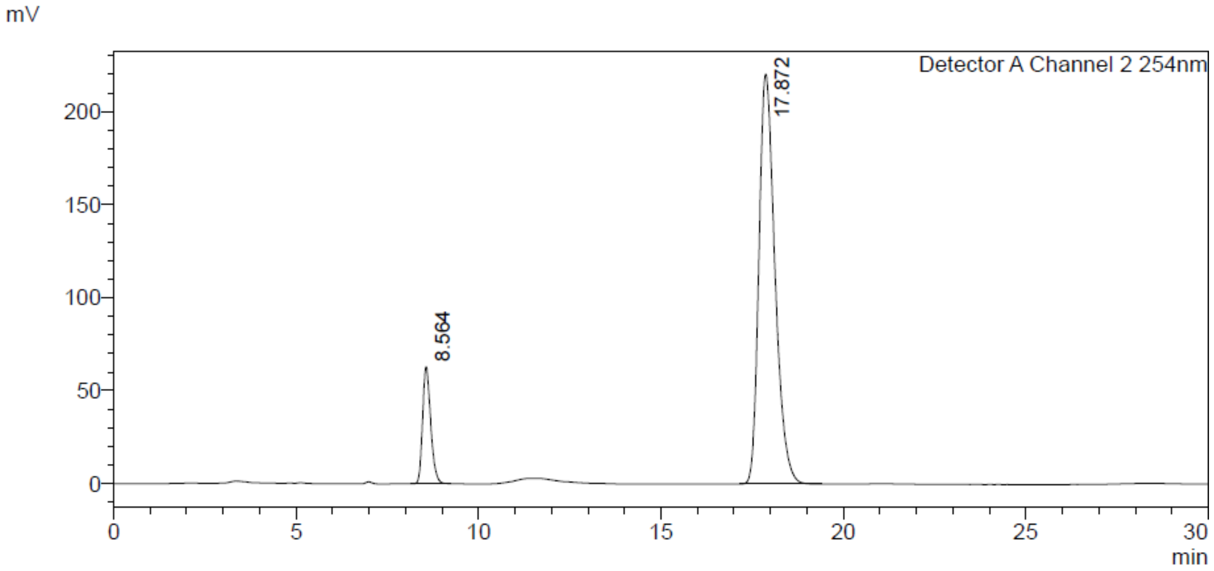

<Peak Table>

Detector A Channel 2 254nm

Peak#	Ret. Time	Area%
1	8.564	12.588
2	17.872	87.412
Total		100.000

HPLC Data for 23: ChiralPak AD-H (20% *i*-PrOH : hexane, flow rate 1 mL·min^{-1}, 254 nm, 30 °C), t_R (*R*)-**23**: 11.5 min, t_R (*S*)-**23**: 14.4 min, 96:4 er.

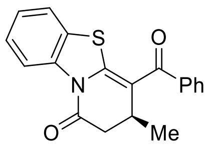

23

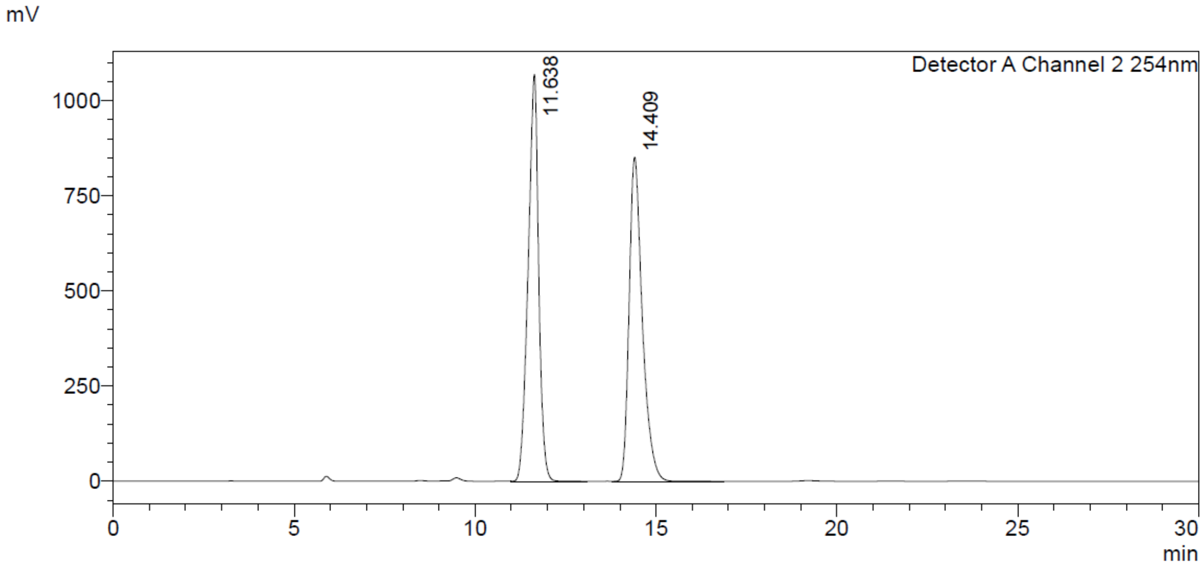

<Peak Table>

Detector A Channel 2 254nm

Peak#	Ret. Time	Area%
1	11.638	49.973
2	14.409	50.027
Total		100.000

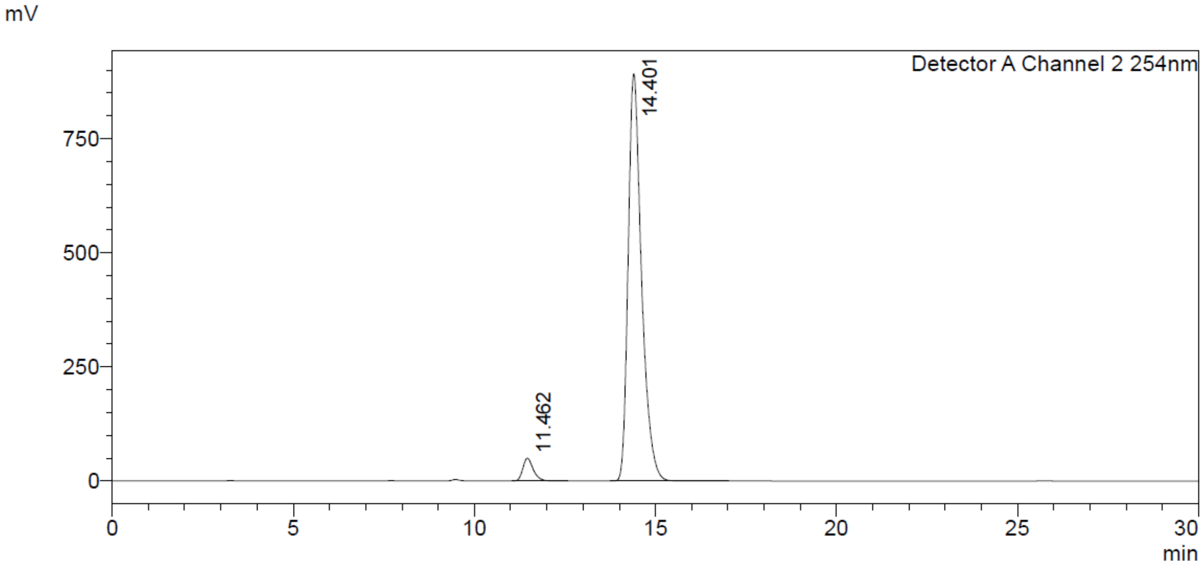

<Peak Table>

Detector A Channel 2 254nm

Peak#	Ret. Time	Area%
1	11.462	4.198
2	14.401	95.802
Total		100.000

HPLC Data for 24: ChiralPak AD-H (20% *i*-PrOH : hexane, flow rate 1 mL·min^{-1}, 254 nm, 30 °C), t_R (*R*)-**24**: 5.9 min, t_R (*S*)-**24**: 9.5 min, 95:5 er.

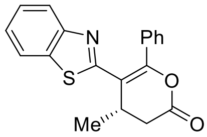

24

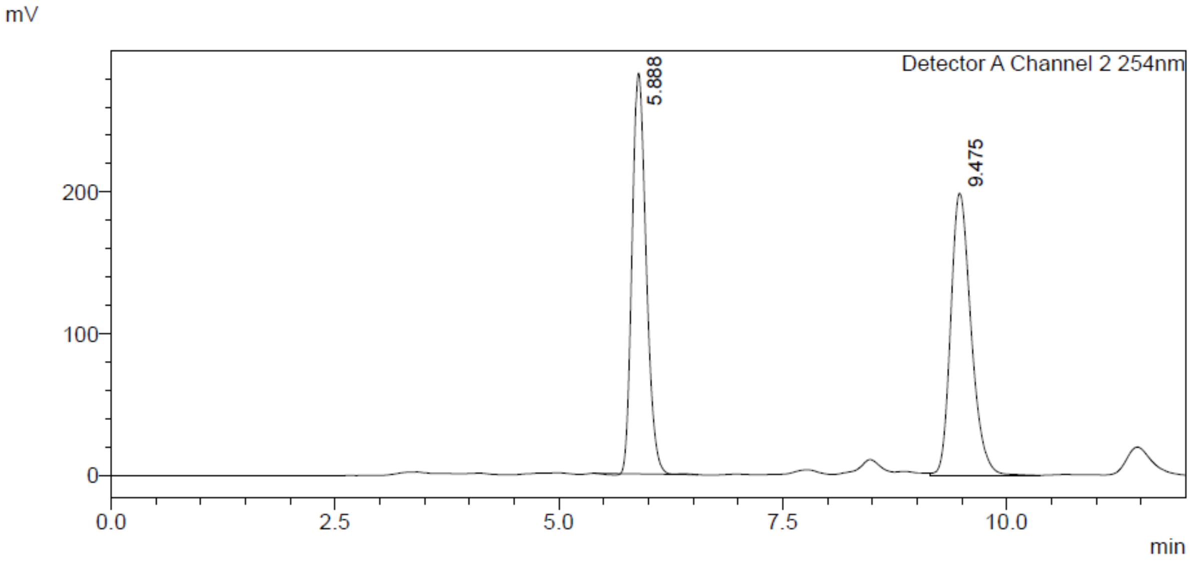

<Peak Table>

Detector A Channel 2 254nm

Peak#	Ret. Time	Area%
1	5.888	49.483
2	9.475	50.517
Total		100.000

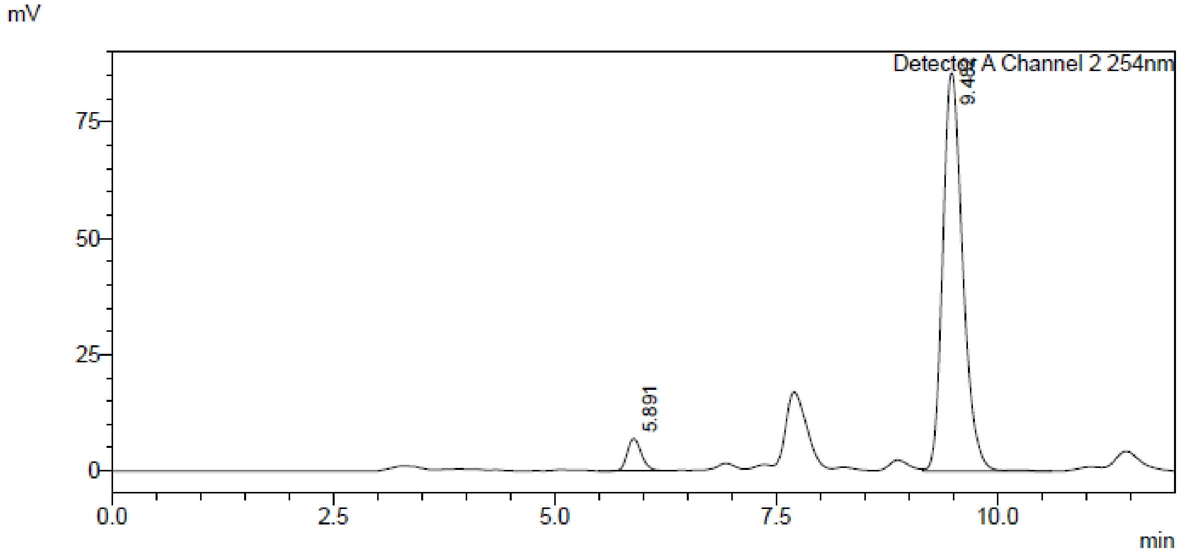

<Peak Table>

Detector A Channel 2 254nm

Peak#	Ret. Time	Area%
1	5.891	5.323
2	9.482	94.677
Total		100.000

HPLC Data for 25: ChiralPak AD-H (5% *i*-PrOH : hexane, flow rate 1 mL·min^{-1}, 254 nm, 30 °C), t_R (*S*)-**25**: 31.9 min, t_R (*R*)-**25**: 37.5 min, 94:6 er.

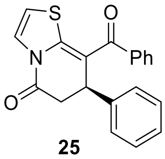

25

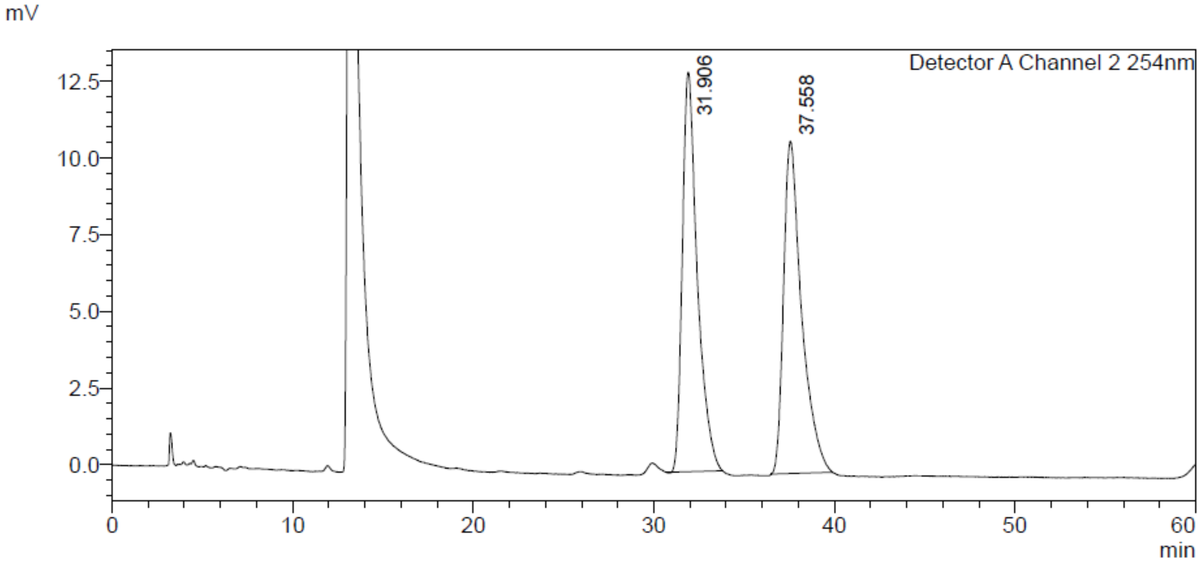

<Peak Table>

Detector A Channel 2 254nm

Peak#	Ret. Time	Area%
1	31.906	49.920
2	37.558	50.080
Total		100.000

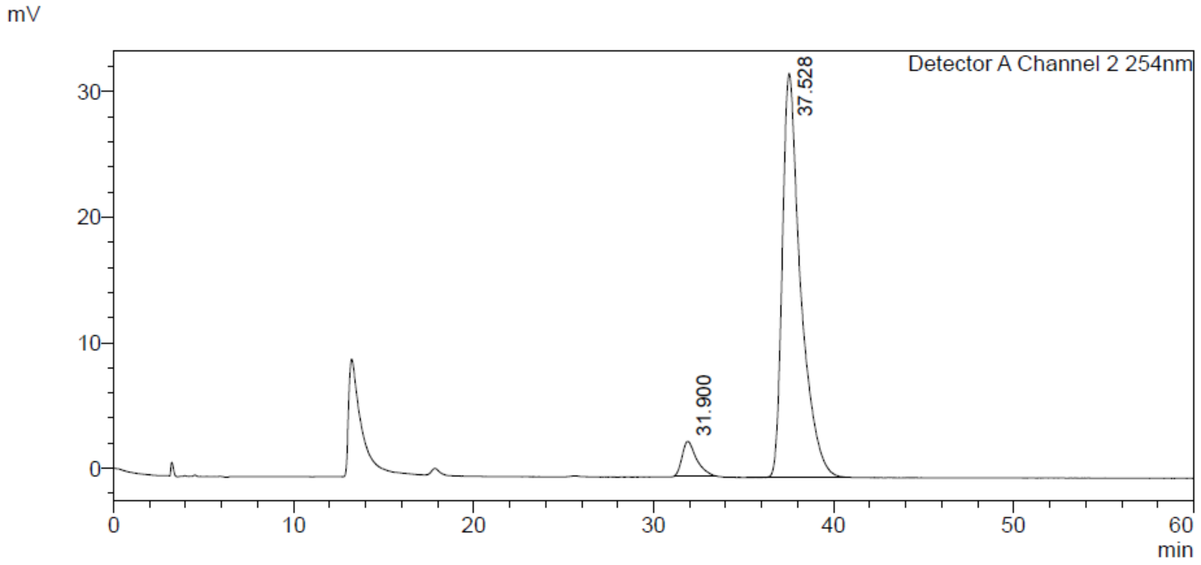

<Peak Table>

Detector A Channel 2 254nm

Peak#	Ret. Time	Area%
1	31.900	6.303
2	37.528	93.697
Total		100.000

HPLC Data for 25 (15 mmol scale-up): ChiralPak AD-H (5% *i*-PrOH : hexane, flow rate 1 mL·min^{-1}, 254 nm, 30 °C), t_R (*S*)-**25**: 32.2 min, t_R (*R*)-**25**: 37.8 min, 92:8 er.

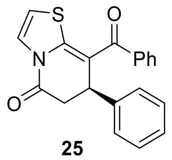

25

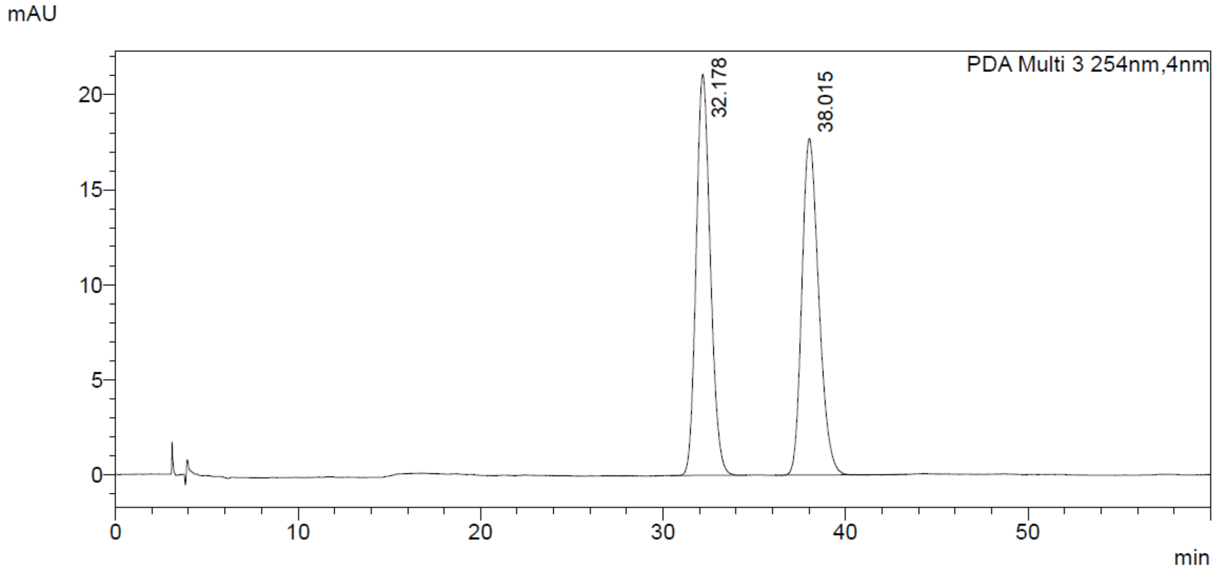

<Peak Table>

PDA Ch3 254nm

Peak#	Ret. Time	Area%
1	32.178	50.098
2	38.015	49.902
Total		100.000

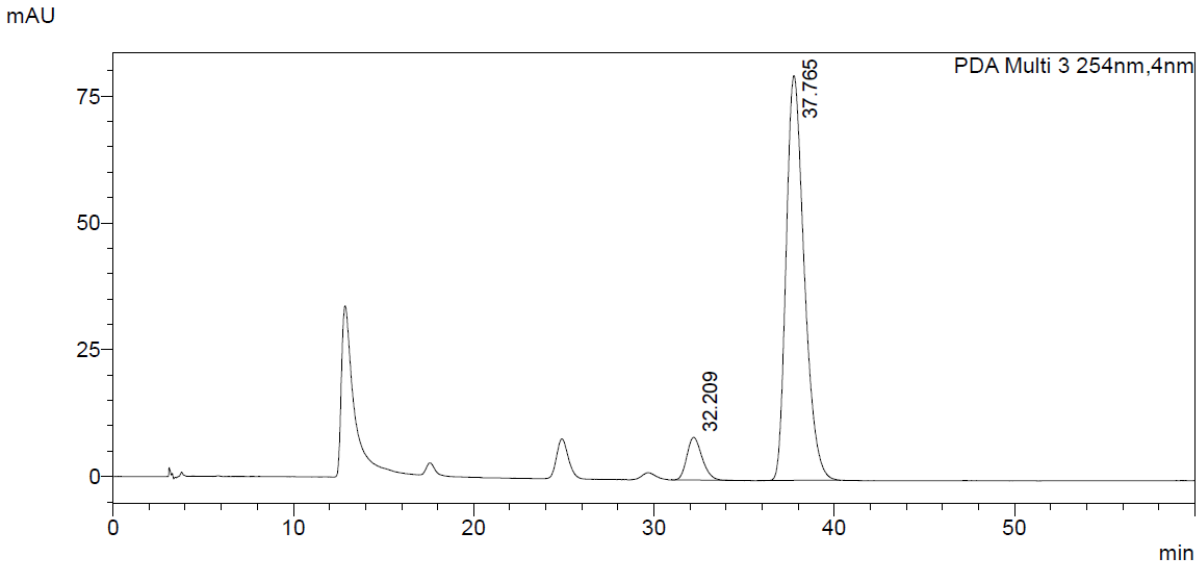

<Peak Table>

PDA Ch3 254nm

Peak#	Ret. Time	Area%
1	32.209	8.146
2	37.765	91.854
Total		100.000

HPLC Data for 25 (recrystallization): ChiralPak AD-H (5% *i*-PrOH : hexane, flow rate 1 mL·min^{-1}, 254 nm, 30 °C), t_R (*S*)-**25**: 32.3 min, t_R (*R*)-**25**: 38.1 min, 99:1 er.

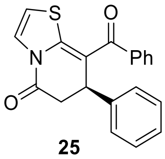

25

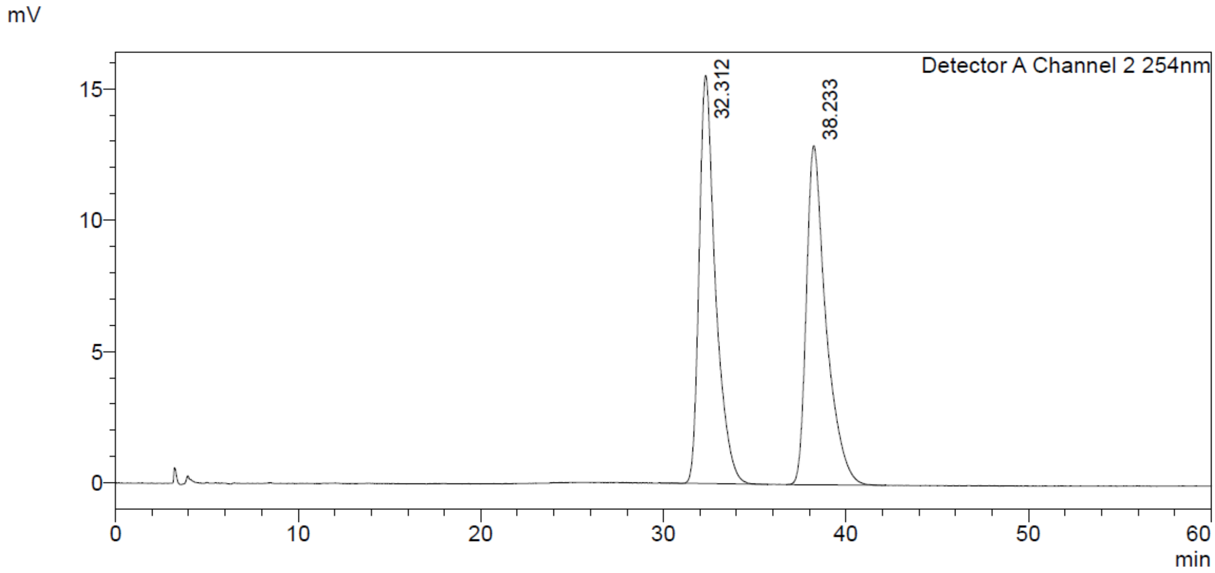

<Peak Table>

Detector A Channel 2 254nm

Peak#	Ret. Time	Area%
1	32.312	50.037
2	38.233	49.963
Total		100.000

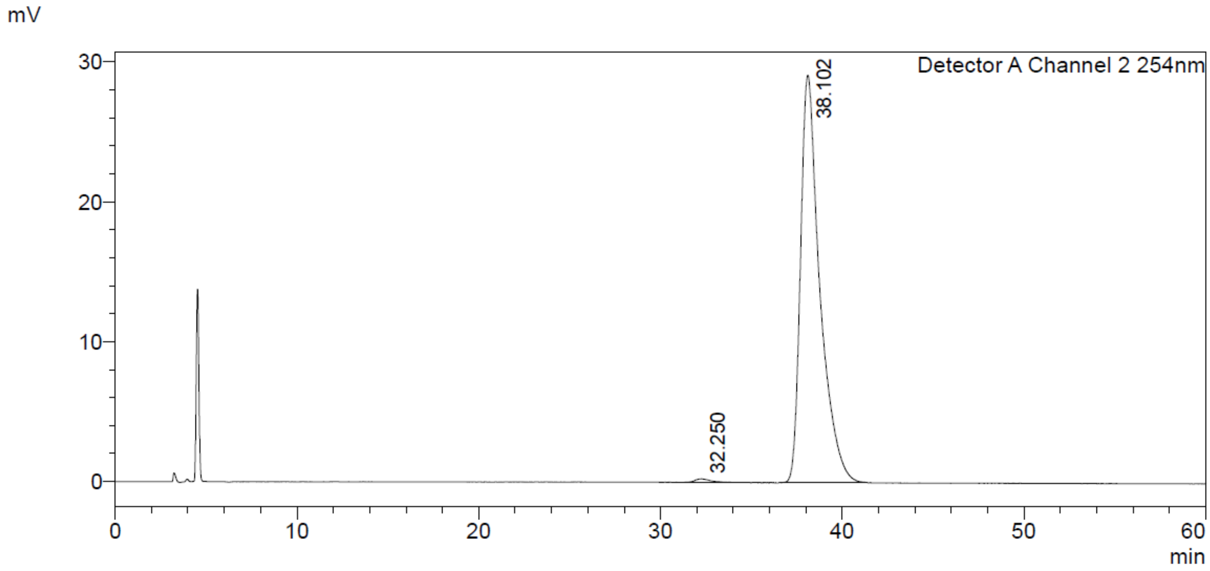

<Peak Table>

Detector A Channel 2 254nm

Peak#	Ret. Time	Area%
1	32.250	0.739
2	38.102	99.261
Total		100.000

HPLC Data for 26: ChiralPak AD-H (5% *i*-PrOH : hexane, flow rate 1 mL·min^{-1}, 254 nm, 30 °C), t$_R$ (*R*)-**26**: 24.1 min, t$_R$ (*S*)-**26**: 28.7 min, 91:9 er.

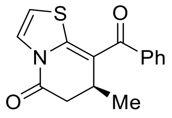

26

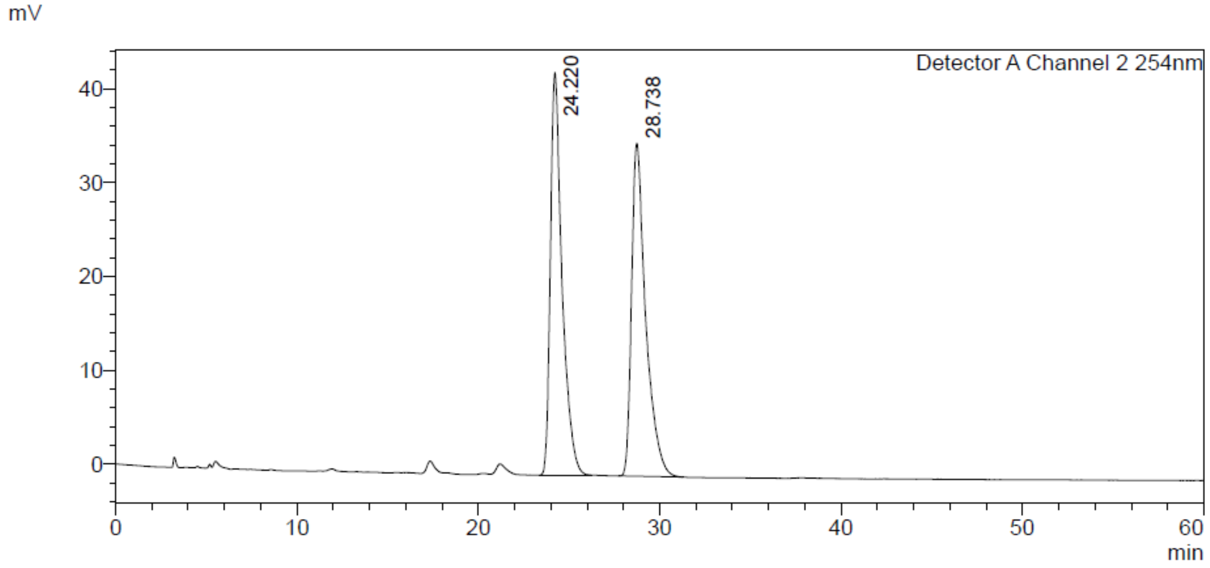

<Peak Table>

Detector A Channel 2 254nm

Peak#	Ret. Time	Area%
1	24.220	49.953
2	28.738	50.047
Total		100.000

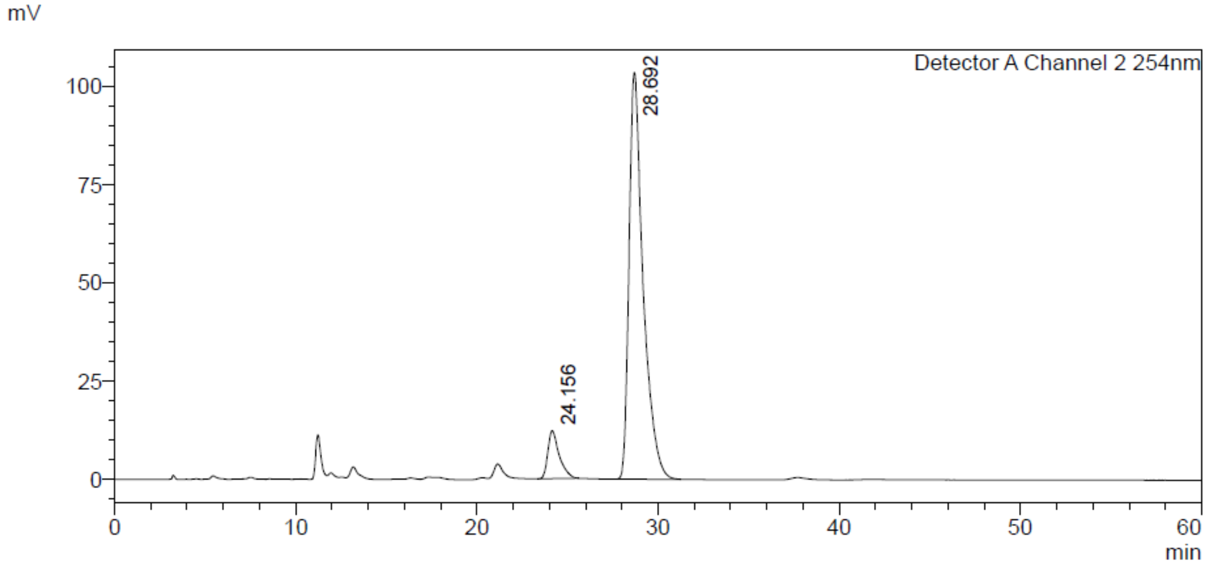

<Peak Table>

Detector A Channel 2 254nm

Peak#	Ret. Time	Area%
1	24.156	8.745
2	28.692	91.255
Total		100.000

HPLC Data for 27: ChiralPak OJ-H (10% *i*-PrOH : hexane, flow rate 1 mL·min^{-1}, 254 nm, 30 °C), t_R (*R*)-**27**: 19.6 min, t_R (*S*)-**27**: 25.2 min, 98:2 er.

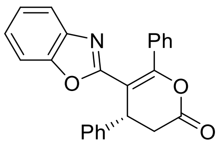

27

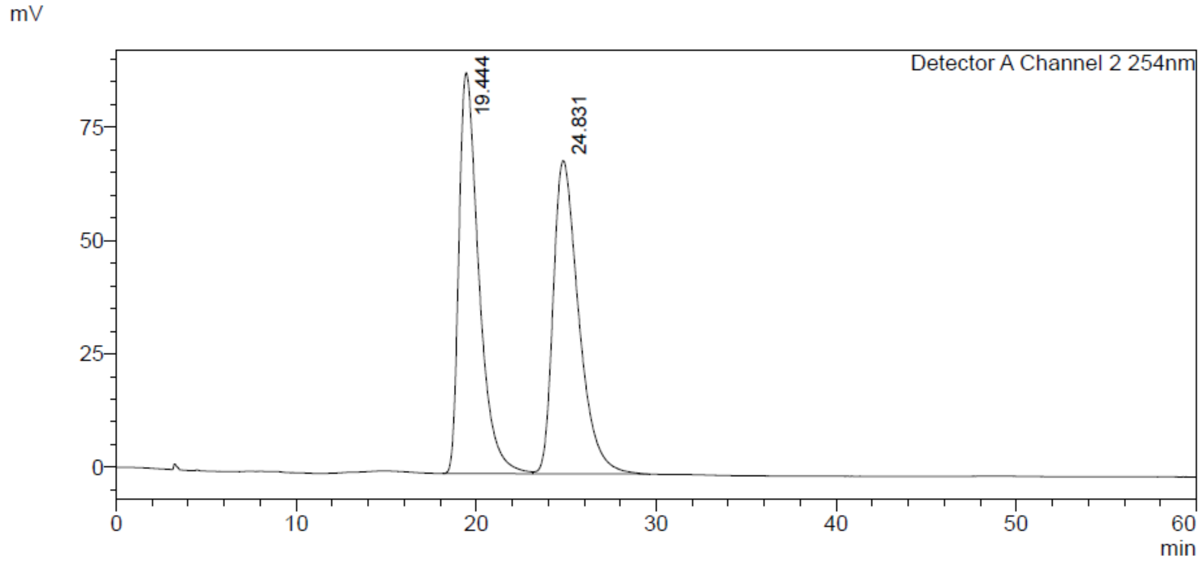

<Peak Table>

Detector A Channel 2 254nm

Peak#	Ret. Time	Area%
1	19.444	50.040
2	24.831	49.960
Total		100.000

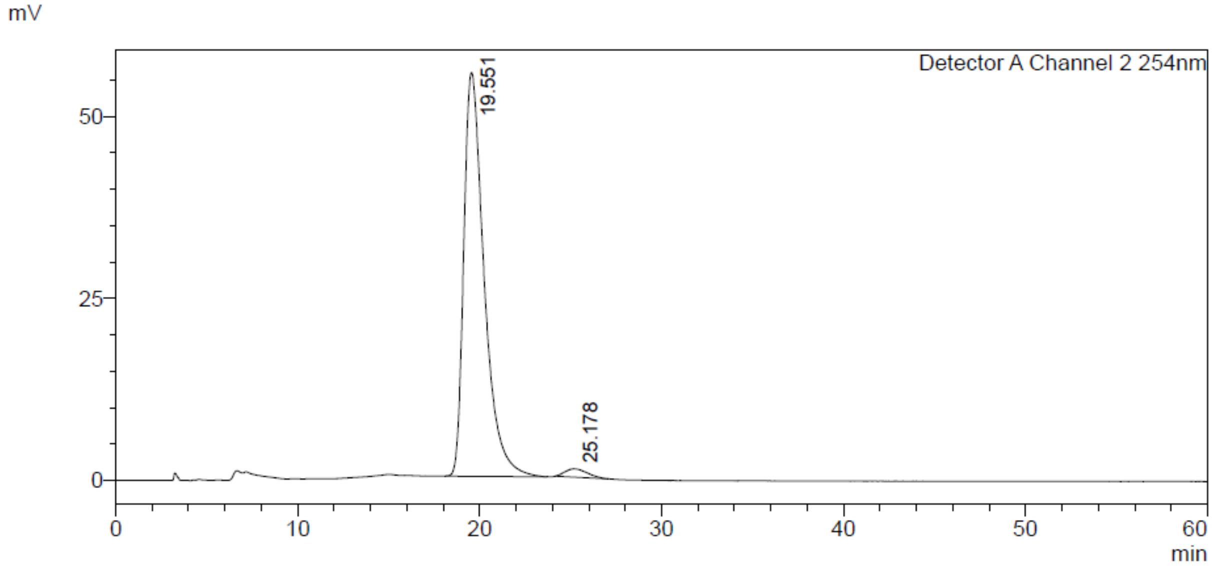

<Peak Table>

Detector A Channel 2 254nm

Peak#	Ret. Time	Area%
1	19.551	97.830
2	25.178	2.170
Total		100.000

HPLC Data for 28: ChiralPak AD-H (5% *i*-PrOH : hexane, flow rate 1 mL·min^{-1}, 254 nm, 30 °C), t_R (*R*)-**28**: 9.1 min, t_R (*S*)-**28**: 15.3 min, 92:8 er.

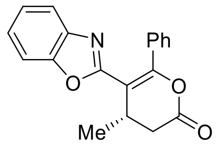

28

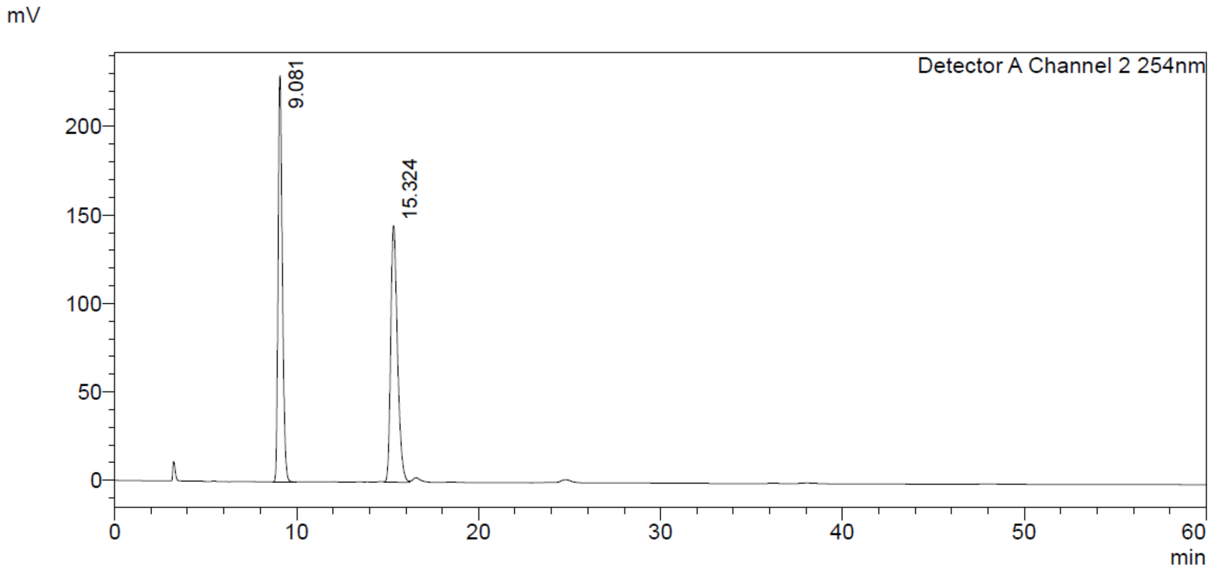

<Peak Table>

Detector A Channel 2 254nm

Peak#	Ret. Time	Area%
1	9.081	49.954
2	15.324	50.046
Total		100.000

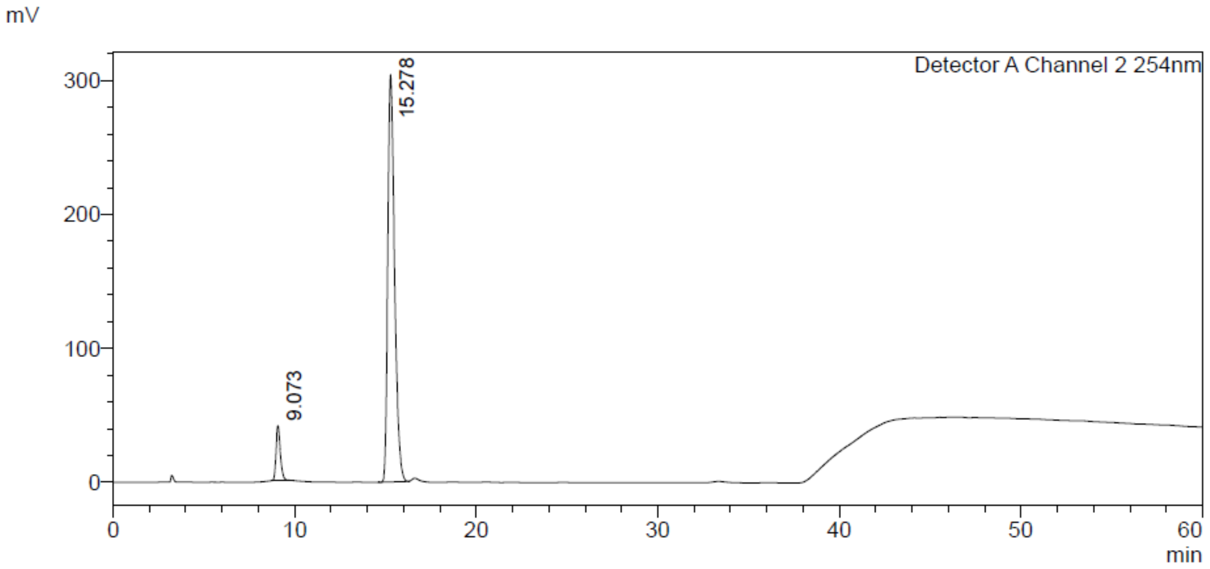

<Peak Table>

Detector A Channel 2 254nm

Peak#	Ret. Time	Area%
1	9.073	7.967
2	15.278	92.033
Total		100.000

HPLC Data for 29: ChiralPak AD-H (20% *i*-PrOH : hexane, flow rate 1 mL·min^{-1}, 254 nm, 30 °C), t_R (*S*)-**29**: 6.0 min, t_R (*R*)-**29**: 34.1 min, 90:10 er.

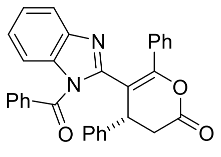

29

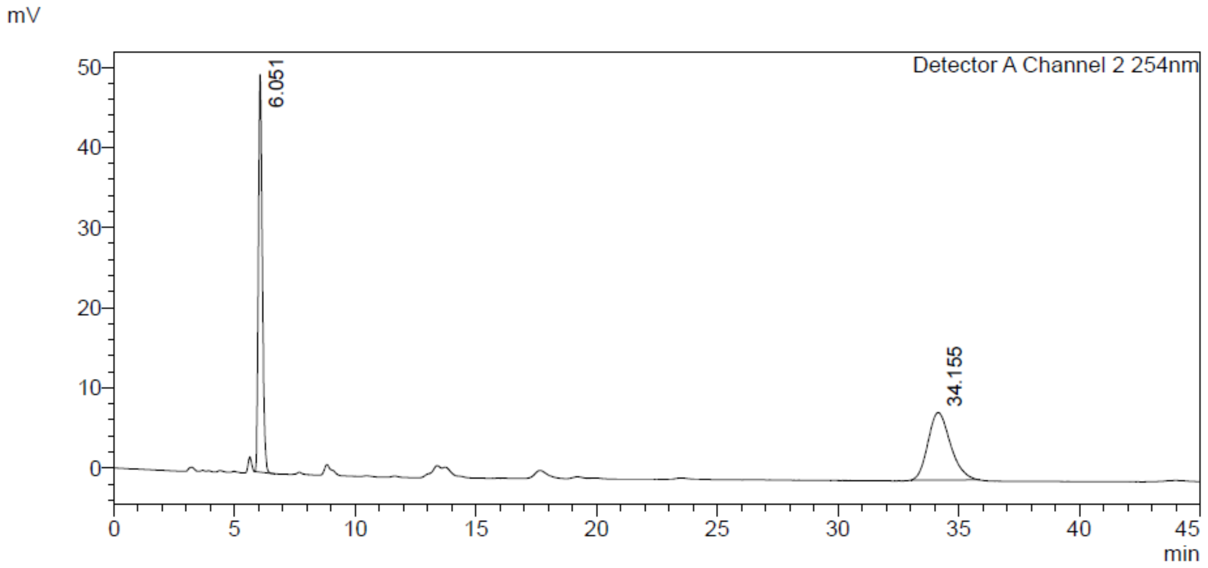

<Peak Table>

Detector A Channel 2 254nm

Peak#	Ret. Time	Area%
1	6.051	50.893
2	34.155	49.107
Total		100.000

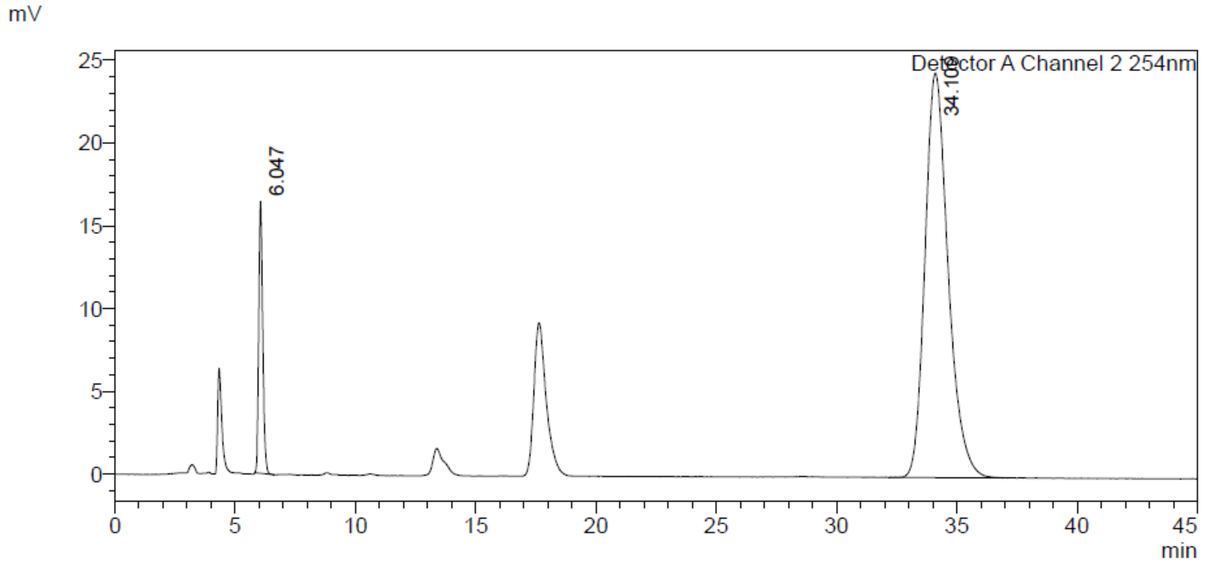

<Peak Table>

Detector A Channel 2 254nm

Peak#	Ret. Time	Area%
1	6.047	10.405
2	34.109	89.595
Total		100.000

HPLC Data for 30: ChiralPak AD-H (20% *i*-PrOH : hexane, flow rate 1 mL·min^{-1}, 254 nm, 30 °C), t_R (*R*)-**30**: 7.9 min, t_R (*S*)-**30**: 46.8 min, 86:14 er.

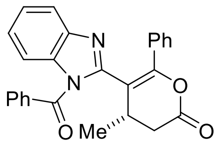

30

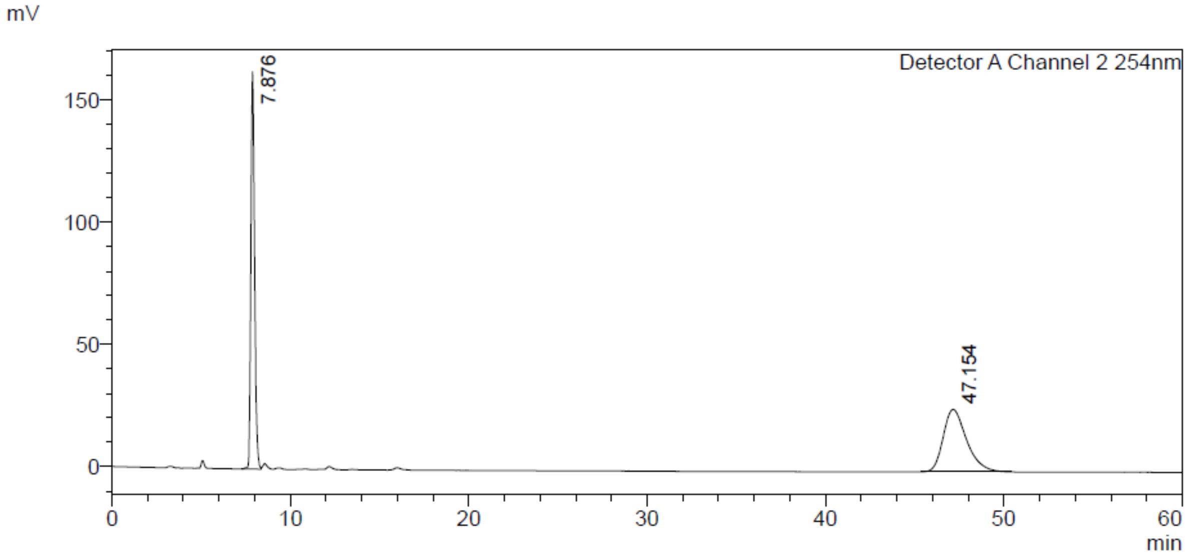

<Peak Table>

Detector A Channel 2 254nm

Peak#	Ret. Time	Area%
1	7.876	50.121
2	47.154	49.879
Total		100.000

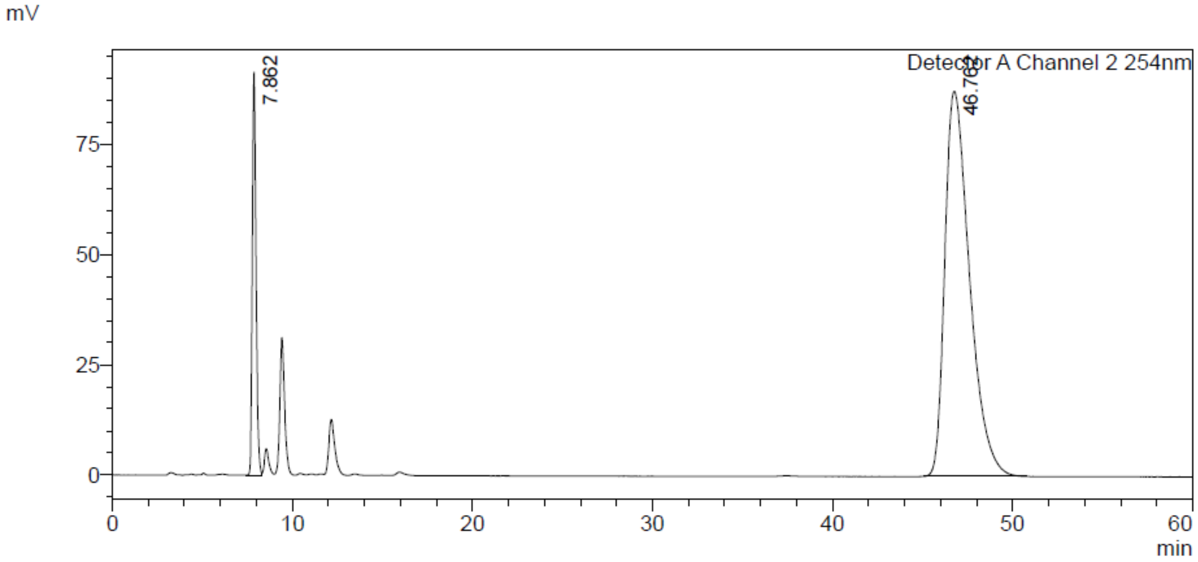

<Peak Table>

Detector A Channel 2 254nm

Peak#	Ret. Time	Area%
1	7.862	13.604
2	46.762	86.396
Total		100.000

HPLC Data for 31: ChiralPak AD-H (20% *i*-PrOH : hexane, flow rate 1 mL·min^{-1}, 254 nm, 30 °C), t_R (*R*)-**31**: 5.1 min, t_R (*S*)-**31**: 8.5 min, 86:14 er.

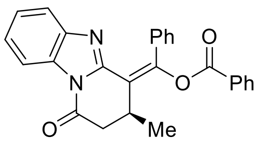

31

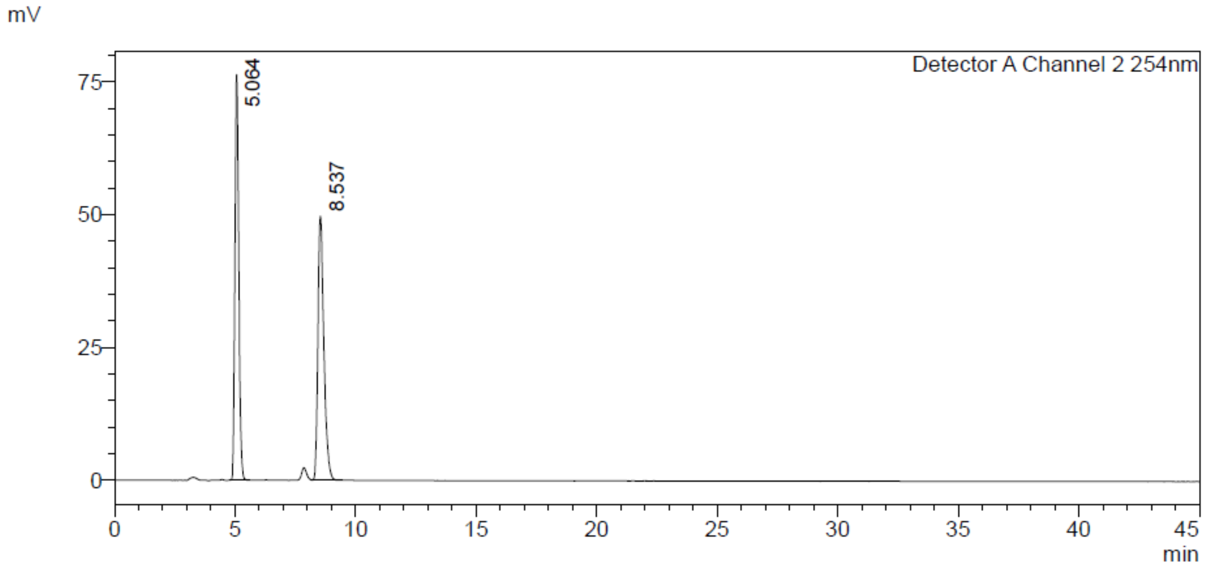

<Peak Table>

Detector A Channel 2 254nm

Peak#	Ret. Time	Area%
1	5.064	50.007
2	8.537	49.993
Total		100.000

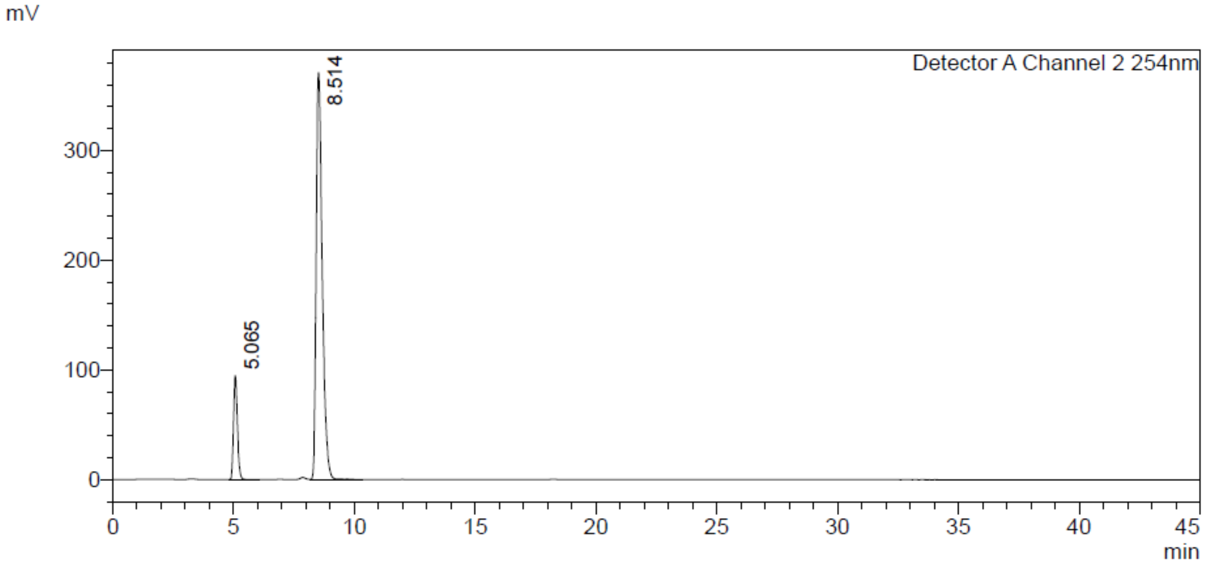

<Peak Table>

Detector A Channel 2 254nm

Peak#	Ret. Time	Area%
1	5.065	13.931
2	8.514	86.069
Total		100.000

HPLC Data for 32: ChiralPak AD-H (20% *i*-PrOH : hexane, flow rate 1 mL·min^{-1}, 254 nm, 30 °C), t_R (*R*)-**32**: 16.8 min, t_R (*S*)-**32**: 26.9 min, 98:2 er.

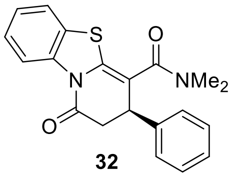

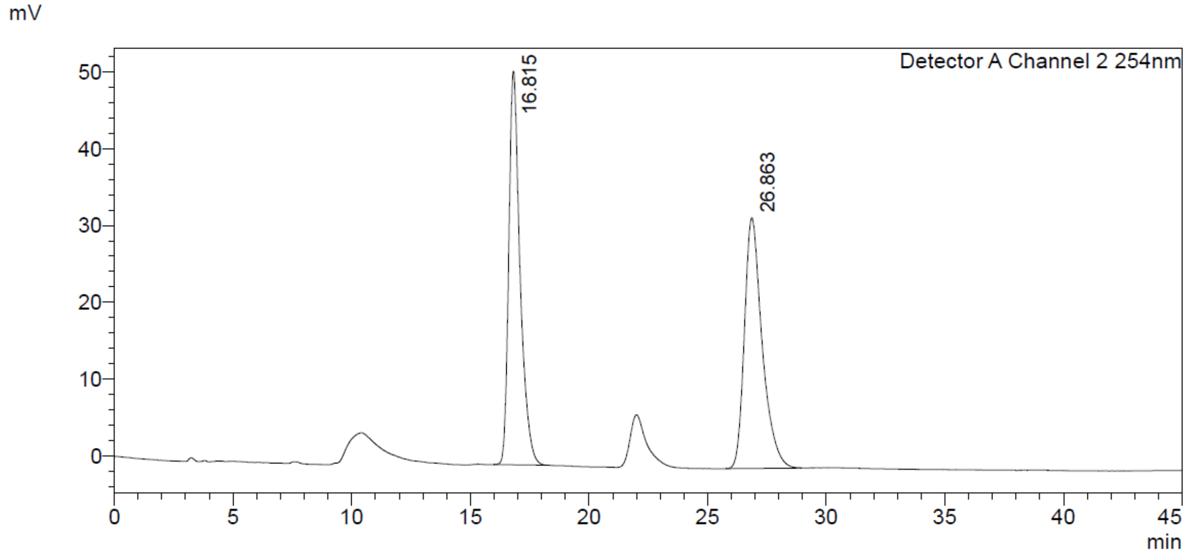

<Peak Table>

Detector A Channel 2 254nm

Peak#	Ret. Time	Area%
1	16.815	49.893
2	26.863	50.107
Total		100.000

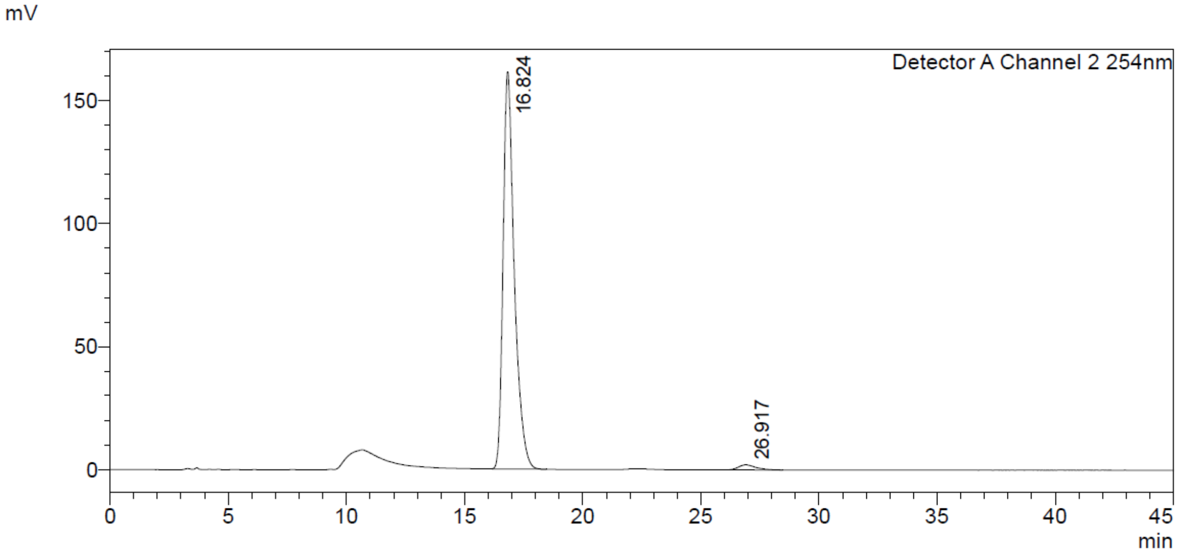

<Peak Table>

Detector A Channel 2 254nm

Peak#	Ret. Time	Area%
1	16.824	98.048
2	26.917	1.952
Total		100.000

HPLC Data for 33: ChiralPak AD-H (20% *i*-PrOH : hexane, flow rate 1 mL·min^{-1}, 254 nm, 30 °C), t_R (*S*)-**33**: 9.6 min, t_R (*R*)-**33**: 13.6 min, 80:20 er.

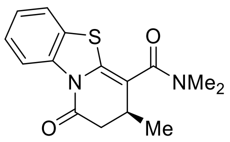

33

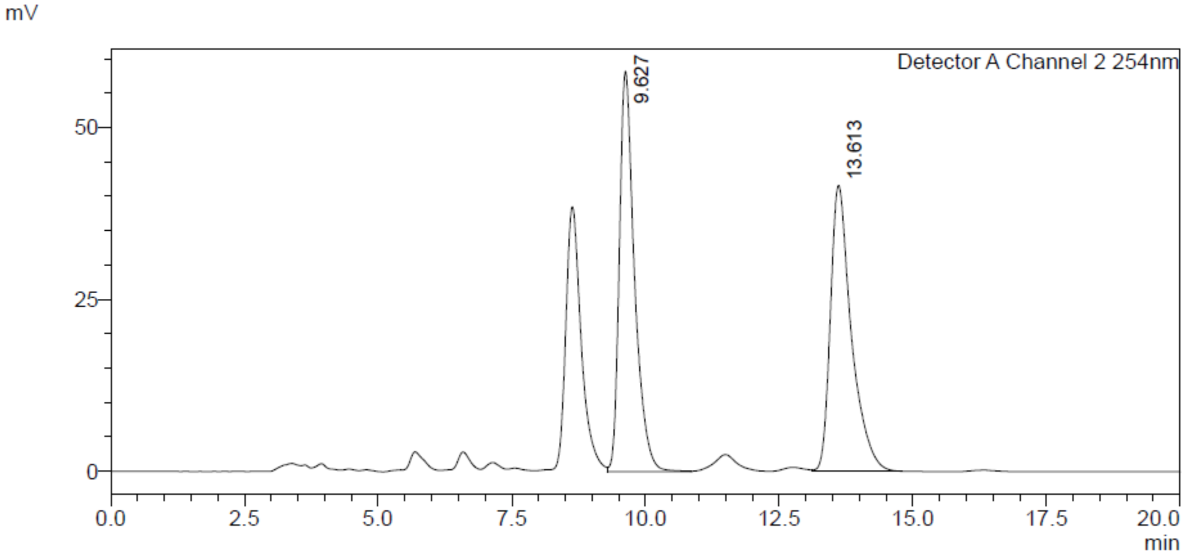

<Peak Table>

Detector A Channel 2 254nm

Peak#	Ret. Time	Area%
1	9.627	50.320
2	13.613	49.680
Total		100.000

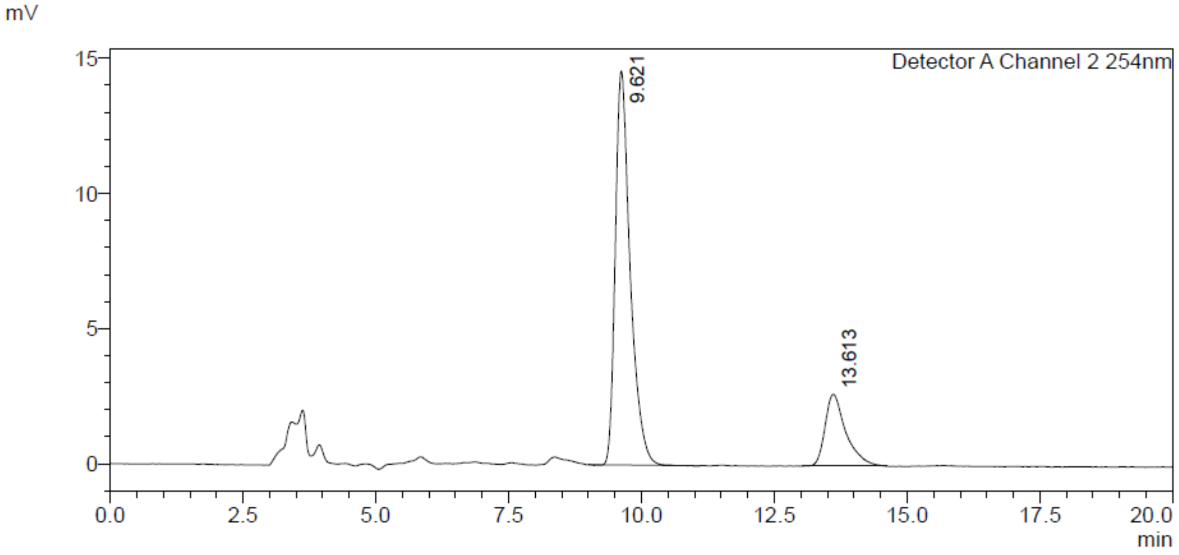

<Peak Table>

Detector A Channel 2 254nm

Peak#	Ret. Time	Area%
1	9.621	79.718
2	13.613	20.282
Total		100.000
